# Geminal group-directed olefinic C-H functionalization via four- to eight-membered *exo*-metallocycles

**DOI:** 10.1038/s41467-019-13098-1

**Published:** 2019-11-08

**Authors:** Keke Meng, Tingyan Li, Chunbing Yu, Cong Shen, Jian Zhang, Guofu Zhong

**Affiliations:** College of Materials, Chemistry and Chemical Engineering, Hangzhou Normal University, Hangzhou, 311121 China

**Keywords:** Catalytic mechanisms, Homogeneous catalysis, Synthetic chemistry methodology

## Abstract

Great efforts have been made in the activation of a C(alkenyl)-H bond vicinal to the directing group to proceed *via* five- or six-membered *endo*-metallocycles. In stark contrast, functionalization of a C(alkenyl)-H bond geminal to the directing group via *exo*-metallocycle pathway continued to be elusive. Here we report the selective transformation of an olefinic C-H bond that is geminal to the directing group bearing valuable hydroxyl, carbamate or amide into a C-C bond, which proceeds through four- to eight-membered *exo*-palladacycles. Compared to the reported mechanisms proceeding only through six-membered *exo*-palladacycles via *N,N*-bidentate chelation, our weak and *O*-monodentate chelation-assisted C(alkenyl)-H activations tolerate longer or shorter distances between the olefinic C-H bonds and the coordinating groups, allowing for the functionalizations of many olefinic C-H bonds in alkenyl alcohols, carbamates and amides. The synthetic applicability has been demonstrated by the preparative scale and late-stage C-H functionalization of steroid and ricinoleate derivatives.

## Abstract (web summary)

Identifying distinct cyclometallation pathways is essential to new types of C-H activation and functionalization in organic synthesis. Here, the authors show the activation of an olefinic C-H bond geminal to the directing group via four- to eight-membered *exo*-palladacycles with broad substrate scope.

## Introduction

Alkenes are commonly present structural motifs and are versatile building blocks in organic synthesis^1^. Direct functionalization of unactivated alkenyl C-H bonds represents the most straightforward way to valuable alkenes from simpler ones^2–8^. A fundamental transformation for direct olefinic C-H functionalization is the Heck reaction, which proceeds by olefin insertion followed by *β*-hydride elimination^2–4^. In recent years, radical C-H alkenylations have also been developed with a variety of carbon/heteroatom-centered radicals, proceeding through radical addition to alkenes and following single-electron-transfer (SET) oxidation/elimination^5^. However, the site- and stereo-selectivity of these methods are largely governed by intrinsic steric and electronically biased properties of the alkene substrates due to the addition-elimination mechanisms. Actually, controlling the site- and stereo-selectivity of C(alkenyl)-H cleavage still remains a formidable challenge due to very subtle differences in terms of bond strength and electronic properties.

Pioneered by Murai´s work on Ru-catalyzed carbonyl-directed *ortho*-C–H activation, broadly defined directing groups (DGs) have served as highly effective tools for controlling C–H bond activations via cyclometallations^6–8^. Most of the directed aromatic- and aliphatic C-H activations proceeded through a normal five- or six-membered cyclometallated intermediate, however, there are very limited reports on protocols by distinct cyclometallations. The Yu group previously disclosed a Pd(II)-catalyzed aromatic C-H functionalizations directed by distal weakly coordinating functional groups via six- or seven-membered cyclopalladation^9^. The same group also reported a nitrile-based template directed *meta*-selective C–H alkenylation, proceeding by a macrocyclic pre-transition state^10,11^. Functionaliztion of *para* C–H bonds was addressed as well using the similar template-based strategy by the Maiti group^12^. Chelation-assisted strategy was also widely used in aliphatic C–H activations^13^. The Gaunt group reported a palladium-catalyzed C–H bond activation through a four-membered cyclopalladation pathway, leading to the selective synthesis of nitrogen heterocycles^14^. Sanford and co-workers described a nitrogen-directed transannular C(alkyl)-H activation of alicyclic amine cores^15^. Very recently, the Yu group disclosed an aliphatic γ-C–H arylation protocol to be proceeded by conventionally disfavored six-membered cyclopalladation, using a strained directing group derived from pyruvic acid^16^. Considering the number of novel reactions that have arisen from metallacycle intermediates, identification of distinct cyclometallation pathways would lead to novel C–H bond transformations.

**Fig. 1:**
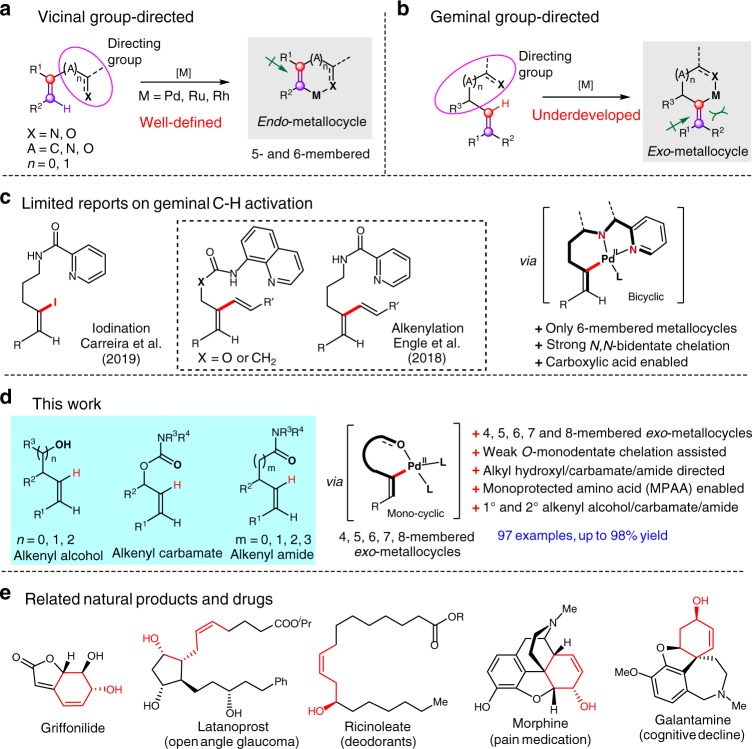
Chelation-assisted olefinic C-H functionalization. **a**, **b** Type of group-directed olefinic C–H activation. **c** Reports on geminal olefinic C–H activation by bicyclic palladacycles. **d** Geminal olefinic C–H functionalization of alkenyl alcohols, carbamates and amides (this work). **e** Structurally related bioactive and natural molecules

Remarkable efforts have been made in the vicinal group-directed olefinic C-H bond functionalization such as alkenylation^17–19^, alkynylation^20–22^, alkylation^23,24^, and others^25–34^. However, their applicability can be severely curtailed by vicinal selectivity displayed through the only 5- or 6-membered *endo*-metallocycles, and by the biased electronic and steric properties of the alkene substrates (Fig. 1a). Moreover, the cyclometallation concept has been remarkably expanded to positional selective C-H bond activation in arenes and alkanes^6–16^, but its utilization in site-selective C(alkenyl)-H bond functionalization still remains elusive^17–35^.

Formation of *exo*-metallocycle is rare in catalytic reactions^36–39^ and is often less competitive when forming an *endo*-metallocycle is possible, presumably due to added conformational degrees of freedom and increased energy (Fig. 1b)^41^. Dong and co-workers demonstrated an *exo*-directing-group-enabled functionalization of sp^3^ C–H bonds via five-membered *exo*-palladacycles to provide 1,2-diols^38^. Recently, Engle’s group reported a carboxylic acid-enabled C(alkenyl)-H functionalization via a characterized six-membered palladacycle, using strong *N,N*-bidentate auxiliary which was removable under nickel-catalyzed methanolysis conditions^39^. Carreira and co-workers also demonstrated an alkenyl C–H iodination via six-membered alkenyl palladacycle intermediate, using picolinamide as the bidentate-chelation directing group (Fig. 1c)^40^. However, the major limitation is that the exquisite selectivity is strictly restricted to the cleavage of the C(alkenyl)–H bond that will result in six-membered *exo*-cyclopalladation. Moreover, the installation and removal of strongly coordinating directing group may impede the widespread application of these transformations.

Hydroxyl, carbamate and amide are valuable and widely occurring functionalities which have been used in C–H functionalizaiton^6–8^, and we envisioned that these weak and *mono*-dentate directing group could also enable the distal geminal C(alkenyl)-H activation by *mono*-cyclic *exo*-cyclometallation. Comparing to thermodynamically more stable and bicyclic palladacycles by *N,N*-bidentate chelation, *mono*-dentate-chelation protocol may functionalize widespread geminal carbon centers that are one or more bonds further away from functional groups, by the formation of small- to medium-sized *mono*-cyclic metallocycle intermediate, which are transient but less strained. While the formation of four-membered metallocycle is highly challenging, seven- and eight-membered cyclometallation are also much less favored in general as the aliphatic carbon between the alkenyl motif and the coordinating group rotates freely and increases the entropic barrier significantly^9,14^. In line with our ongoing interest in olefinic C–H activation^19,34^, herein, we focus on *gem*-olefinic C-H activation via four- to eight-membered *exo*-cyclometallation, and the utility is characterized by the C–H functionalization of a wide range of functionalized alkenes, including synthetically valuable homoallyl-, bishomoallyl- and allyl alcohols/carbamates/amides that constitute integral structural motifs in natural products and drug design (Fig. 1d, e).

## Results

### Development of *gem*-group-directed alkenyl C-H alkenylation

As the alcohol can be naturally incorporated into the product without installation and removal steps in C–H functionalization, we started investigation by employing previously reported Ru-, Rh- or Pd-catalyzed systems for hydroxyl-chelation-assisted C–H alkenylation reactions^42–44^. Unfortunately, (*E*)-3-hexen-1-ol with acrylate led to trace alkenylated/alkylation product and/or poor regioselectivity under a variety of catalytic conditions. In this context, we envisioned that (*Z*)-alkenyl alcohol could be suitable due to the following two inherent effects. First, incorporation of R^1^ group would make target olefinic C–H bond more reactive (Fig. 1b) due to increased nucleophilicity toward transition metal and block or obviate competitive vicinal C-H activation sites. Second, absence of R^2^ group would avoid steric repulsion and facilitate subsequent insertion step. Although various of ligands were inefficient, *mono*-*N*-protected amino acid (MPAA) combined with palladium was found to promote the alkenylation reaction^45,46^, with Ac-Phe-OH emerging as the best one (see Supplementary Table 1). Fine-tuning the chain length between the hydroxyl and olefinic carbon greatly influenced such C-H functionalization. While the reactions of (*Z*)-3-hexen-1-ol (**1a**) (5-*exo*) reacted smoothly with acrylate to provide diene **3** in 69% yield, *cis*-2-hexen-1-ol (4-*exo*) and *cis*-4-decen-1-ol (6-*exo*) led to desired products in only 30% and 15% yields respectively (Table 1, entries 1–3). However, *cis*-5-octen-1-ol (7-*exo*) failed to react (Table 1, entry 4). Reactions of differently substituted alkenyl alcohols were performed to investigate the steric and electronic influences on geminal C-H activation. Unfortunately, (*E*)-3-hexen-1-ol and 4-methyl-3-penten-1-ol led to trace product due to the difficulty in formation of *exo*-palladacycle and/or alkenylation by steric repulsion (Table 1, entries 5 and 6). Meanwhile, terminal alkenyl alcohol showed very limited reactivity presumably due to the decreased nucleophilicity of C-H bond toward transition metal (Table 1, entry 7). Carbamate derived from (*Z*)-allylic alcohol also led to satisfactory results with the help of Ac-Ile-OH ligand instead via 6-membered *exo*-palladacycle (Table 1, entry 8). However, homoallyl carbamate did not react (7-*exo*) (Table 1, entry 9). Similarly, other differently substituted alkenyl carbamate exhibited no reactivity toward acrylate due to disfavored steric and/or electronic influences (Table 1, entries 10–12). Although acrylamides were commonly used in alkenyl C-H fucntionalization, there is still no report on C_sp2_-H transformation of nonconjugated alkenyl amides^17–35^. Herein, amide was successfully demonstrated to be effective in geminal C-H functionalization. Both secondary and tertiary amides provided corresponding geminal products in good yields, which proceeded by 6-membered palladacycles (Table 1, entries 13 and 14). Notably, this protocol also allowed palladium catalyst to functionalize alkenes bearing longer or shorter chain lengths between the amide and olefinic carbon, which proceeded by 5- and even 7-membered palladacycles, albeit with moderate yields (Table 1, entries 15 and 16). Interestingly, a weak coordinating of amide to form 8-membered palladacycle was also observed, albeit with most of the amide substrate recovered (Table 1, entry 17). Moreover, steric and electronic influences on geminal C-H activation were consistent with the reactions of alcohols and carbamates (Table 1, entries 18–20). All of these results showed a precise recognition of distance and geometry in these alkenyl C-H functionalizations. Other alcohol-derived functional groups (FGs), such as ester (FG^5^), ether (FG^6^), urea (FG^7^), acetone oxime ether (FG^8^) and amides with acidic N-H bonds (FG^9^ and FG^10^), were also tested, but none of them could furnish desired C-H functionalization (Table 1, entries 21–26).

**Table 1:** Development of *gem*-group-directed olefinic C–H alkenylation


entry	FG	R^1^	R^2^	*n*	conditions	yield (%)^a^	metallocycle^b^
1	FG^1^	Pr	H	0	A	30 (3va)	4
2	FG^1^	Et	H	1	A	69 (3aa)	5
3	FG^1^	pentyl	H	2	A	15 (3za)	6
4	FG^1^	Et	H	3	A	<5	7
5	FG^1^	Me	Me	1	A	<5	5
6	FG^1^	H	Et	1	A	5	5
7	FG^1^	H	H	1	A	<5	5
8	FG^2^	Pr	H	0	B	56 (5aa)	6
9	FG^2^	Et	H	1	B	<5	7
10	FG^2^	Me	Me	0	B	0	6
11	FG^2^	H	H	0	B	0	6
12	FG^2^	H	Pr	0	B	0	6
13	FG^3^	pentyl	H	1	C	74 (7aa)	6
14	FG^4^	pentyl	H	1	C	60 (7ha)	6
15	FG^4^	pentyl	H	0	C	40 (7qa)	5
16	FG^4^	Et	H	2	C	37 (7ra)	7
17	FG^4^	Et	H	3	C	18 (7ta)	8
18	FG^4^	Me	Me	1	C	0	6
19	FG^4^	H	H	1	C	0	6
20	FG^4^	H	Me	1	C	0	6
21	FG^5^	Et	H	0,1	A,B,C	0	–
22	FG^6^	Et	H	0,1	A,B,C	<5	–
23	FG^7^	Pr	H	0,1	A,B,C	0	–
24	FG^8^	Pr	H	0,1	A,B,C	0	–
25	FG^9^	pentyl	H	0,1	A,B,C	0	–
26	FG^10^	pentyl	H	0,1	A,B,C	0	–

^a^The yields are isolated yields

^b^The sizes of exo-metallocycles

### Substrate scope

With the optimized reaction conditions in hand, we firstly examined the scope and limitation of alkenes **2** by employing alcohol **1a** as the substrate (Table 2). It was found that a wide variety of acrylates **2** could provide the alkenylated products in 39–81% yields (**3aa-3ai**). Remarkably, vinyl ketones were also suitable coupling partners (**3aj** and **3ak**). Moreover, both vinyl phosphonate and styrene were reacted smoothly, affording products **3al** and **3am** in 75% and 54% yields, respectively. Even acrylamides reacted well, though amides are usually prone to coordination (**3an** and **3ao**). Thereafter, we turned our attention to expand the scope of the reaction to other representative homoallylic alcohols **1**. Alcohols bearing longer alkyl chain also reacted well (**3ba** and **3ca**). Introducing of alkyl group at the allylic position showed moderate yield (**3da**). The versatile catalytic system was not limited to primary alcohols. Indeed, we were pleased to identify easily oxidized secondary alcohols as viable substrates in this protocol likewise^47^. Incorporation of alkyl, aryl and even alkenyl groups at the carbon adjacent to hydroxyl group showed good results (**3ea-3sa**). In particular, aryl ring bearing substituents such as F and Cl can be well tolerated in this protocol (**3na** and **3oa**), which is synthetically useful for further elaborations of the products. Unfortunately, alkenyl alcohol bearing sensitive *para*-Br led to decreased yield due to undesired side reactions (**3qa**). Interestingly, easily decomposed tertiary alcohol also reacted, but leading to cyclization products **3A** (33%) and **3A**′ (17%) due to restricted conformational freedom and bond angle compression. Herein, the *Z*-configuration of product **3A**′ implied the involvement of the *anti*-alkoxypalladation process in the cyclization^41,42^. Moreover, 4-phenyl-3-buten-1-ol converted well (**3ua**). Cyclic alcohol such as 2-cyclohexene-1-methanol also reacted, albeit with reduced efficiency (**3ta**).

**Table 2:** Olefinic C–H alkenylation of homoallylic alcohols^a^

Notably, simple allyl alcohols, widely used as allylation reagent^48,49^, are seldom C-H functionalized due to competitive C–H cleavage sites and easy decomposition under catalytic conditions. Meanwhile, four-membered metallocycle is very rare in catalytic reactions^14^, whose utilization in olefinic C–H activation has not been reported^17–35^. To our satisfaction, (*Z*)-allyl alcohols could be alkenylated in moderate yields, using *N*-acetyl-L-isoleucine (Ac-Ile-OH) as the ligand (**3va-3ya**). Moreover, *cis*-4-decen-1-ol was proved to be reactive, albeit with reduced efficiency (**3za**). The synthetic flexibility afforded by allyl- and bishomoallyl alcohols greatly expands the range of core structures that can subsequently be accessed.

The carbamate was usually employed as both a DG and an alcohol surrogate in C–H activation. Herein, allylic alcohol masked as its carbamate could be well C–H functionalized with the help of Ac-Gly-OH ligand instead (Table 3). Various acrylates could be served as coupling partners (**5aa-5ah**). Different dialkyl carbamates were examined to optimize the C–H activation, and installation of bulky isopropyl group led to alkenylated product in 62% yield (**5da-5fa**). Carbamate derived from secondary alcohol also converted without any decrease in efficiency (**5ga**). 3-Hydroxyl cyclohexene is a widely occurring skeleton in bioactive molecules and is highly attractive substrate for C–H functionalization (Fig. 1e). To our delight, carbamate masked 3-hydroxyl cyclohexene provided 62–70% yields (**5ha** and **5ia**). Notably, cyclohexene bearing quaternary carbon, which can be found in medical molecules such as galantamine, led to 95% yield (**5ja**). Finally, this protocol also highlighted tolerance of sensitive benzyl and even cinnamyl moieties, producing products **5ka** and **5la** in 98% and 63% yields respectively.

**Table 3:** Olefinic C–H alkenylation of alkenyl carbamates^a^

^a^ The yields are isolated yields based on carbamate **4**.

Next, we focused on substrate scope of alkyl amide directed geminal C(alkenyl)-H fucntionalization by 5- to 8-membered palladacycles (Table 4). The C-H alkenylation proceeded smoothly with various olefinic coupling partners such as acrylates, vinyl ketone and even styrene, providing 43–74% yields (**7aa-7ah**). Differently *N*-substituted secondary amides also reacted well with acrylates (**7ba-7ea**). Although primary amide led to trace product, tertiary amides led to good yields (**7ha** and **7ia**). Incorporation of alkyl and benzyl groups at the carbon adjacent to amide group showed good results (**7ja**, **7ka** and **7la**). Differently *N*-substituted alkenyl amides **6m-6q** also led to moderate yields proceeded by 5-membered *exo*-palladacycles. Notably, although alkenyl **6r** exhibited decreased reactivity due to the difficulty in the formation of 7-membered *exo*-palladacycle, incorporation of methyl group greatly facilitate the C-H alkenylation (**7sa**, 60% yield). Interestingly, amide **6t** bearing longer alkyl chain still showed limited reactivity toward acrylate (**7ta**).

**Table 4:** Olefinic C–H alkenylation of alkenyl amides^a^

^a^ The yields are isolated yields based on amide **6**.

### Competition experiments and mechanistic considerations

**Fig. 2:**
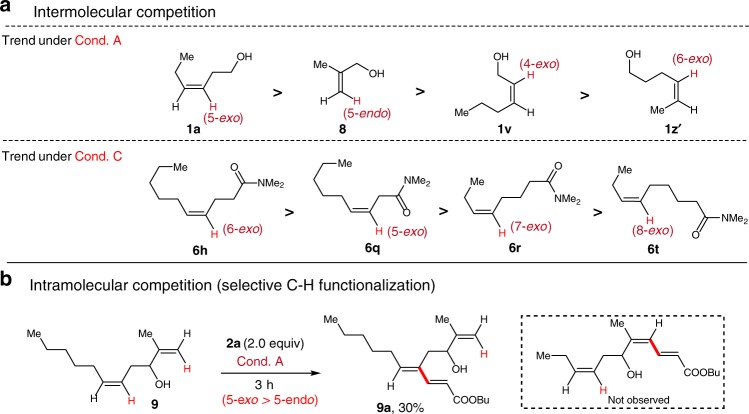
Competition experiments. **a** Intermolecular competition experiments using alkenyl alcohols or alkenyl amides to rank the reaction trend under Cond. A or Cond C. **b** Intramolecular competition experiment using compound **9**

Next, competition experiments were performed for acrylate **2a** and alcohols **1** or amides **6** under optimal conditions to rank the relative reactivities, thus demonstrating the preferential formation of the related alkenyl metallocycle intermediates (see Fig. 2 and Supplementary Tables 4-5).

On the basis of the data compiled from intermolecular competition experiments, the relative reactivities of alcohols **1a**, **1v**, **8** and **1z´** were ranked to be **1a** > **8** > **1v** > **1z´** (Fig. 2a, trend under Cond. A). These results exhibited that alkenyl C-H activation by 5-membered *exo*-palladacycle (**1a**) occurred preferentially over 4-membered *exo*-palladacycle (**1v**), and the C-H activation by 6-membered *exo*-palladacycle (**1z’**) was the most disfavored because the added freely rotating sp^3^ carbon center significantly increases the entropic barrier for assembling the desired transition state, being consistent with our observation in substrate scoping (Table 2). Notably, vicinal C_sp2_-H functionalization by 5-membered *endo*-palladacycle (**8**) was also efficient under Cond. A. However, formation of 5-*exo*-metallocycle was found to be more competitive than the formation of 5-*endo*-metallocycle (**1a** > **8**). An alcohol **9** bearing two alkenyl moieties was synthesized and reacted under Cond. A, and the C-H alkenylation only occurred on *Z*-alkenyl moiety to provide product **9a** (Fig. 2b). Both of the inter- and intra-molecular competition experiments showed the preferential formation of 5-*exo*-palladacycle over 5-*endo*-palladacycle, being inconsistent with previous reports^38,41^. Similarly, while 4-methyl-4-pentenamide exhibited limited reactivity (14% yield) and poor selectivity under conditions C, **7ra** was obtained in 37% yield, suggesting the preferential formation of *exo*-palladacycle. Also, competition studies were performed for acrylate **2a** with amide **6h**, **6q**, **6r** or **6t** under conditions C, and the relative reactivities of amides were ranked to be **6** **h** > **6q** > **6r** > **6t**. These results were generally consistent with prior substrate scoping (Table 4) and the kinetically and thermodynamically favored *exo*-cyclopalladation dominating the geminal C_sp2_-H activation (Fig. 2a, trend under Cond. C). Notably, both substrates **1z’** and **6h** converted by the formation of 6-membered *exo*-palladacycles, but alkenyl amide **6h** reacted efficiently because the carbonyl group reduced conformational degrees of freedom. Moreover, while amide **6r** did react via 7-*exo*-cyclopalladation, homoallyl carbamate did not convert even at elevated temperatures (Table 1, entry 9), exhibiting the great influence of coordinating effect. All of these primary results exhibited that the *exo*- and *endo*-cyclometallations were governed by coordination strength and conformation effects on the C-H activation step^9,16^, and the detailed mechanistic studies will be discussed in a later report. Deuterium incorporation experiments supported the irreversibility of the C-H activation steps, and no *Z/E* isomerization excluded other possible mechanistic pathway such as π-allylpalladium(II) formation or nucleometalation/*β*-X elimination^39,50^. These results combined with kinetic isotope effect (KIE) experiments exhibited that the irreversible C-H bond cleavage step originated the site selectivity, thus possible mechanisms based on *exo*-palladacycles are proposed (see Supplementary Figs. 1–4)^51^.

### Preparative scale synthesis and coordinating group removal

**Fig. 3:**
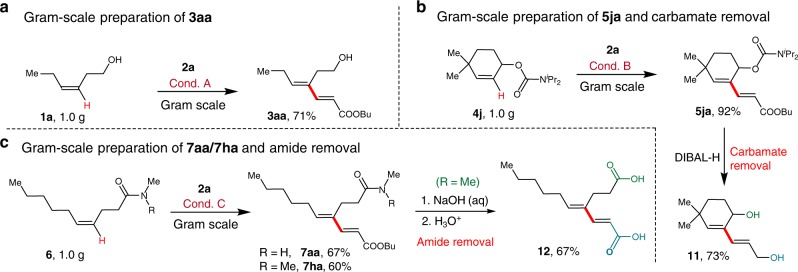
Gram-scale synthesis and directing group removal. **a** Gram-scale synthesis of compound **3aa**. **b** Gram-scale synthesis of compound **5ja** and carbamate removal. **c** Gram-scale synthesis of compound **7aa/7ha** and amide removal

To establish scalability, the conversion of **1a** was run at a gram scale to give **3aa** in 71% yield (Fig. 3a). Olefinic C-H alkenylation of **4j** at a preparative scale was also successful. The removal of the carbamate in **5ja** was readily accomplished by reduction at room temperature to give diol **11** in 73% yield (Fig. 3b). Moreover, geminal C-H olefination of alkenyl amides reacted smoothly at gram-scale, providing **7aa** and **7ha** in good yields. Notably, amide removal was realized by simple hydrolysis, yielding dienoic acid **12** in good yield (Fig. 3c).

### Late-stage C-H functionalization

**Fig. 4:**
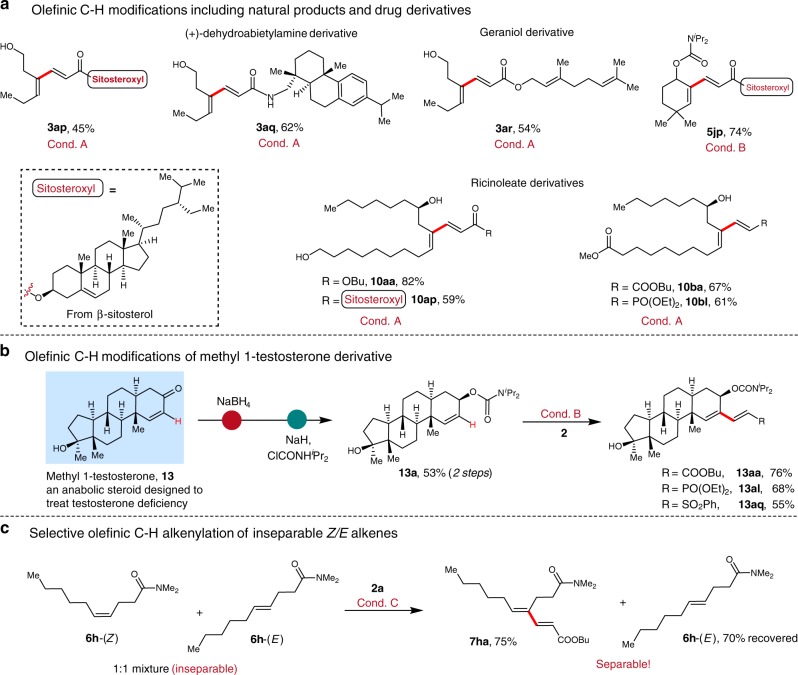
Late-stage C–H functionalization of natural and pharmaceutical compounds. **a** Olefinic C–H modifications including natural products and drug derivatives. **b** Olefinic C–H alkenylation of methyl 1-testosterone derivative. **c** Selective conversion of inseparable *Z/E* alkenyl amides

To further demonstrate the utility of our methods, we attempted late-stage C–H functionalization of natural and medicinal compounds (Fig. 4). Gratifyingly, these protocols were characterized by high chemoselectivity, highlighting the successful conversion of sensitive geraniol and (+)-dehydroabietylamine as well as the cholesteryl derivatives (**3ap**, **3aq**, **3ar** and **5jp**). Ricinoleate derivatives were smoothly reacted, delivering the targets in good to excellent yields (**10aa-10bl**, 59–82% yields) (Fig. 4a). Methyl-1-testosterone is an anabolic steroid derivative to treat male testosterone deficiency. Herein, using steroid-derived carbamate **13a** as a representative drug molecule for diversification, three different alkenylation analogs were readily accessed (**13aa-13aq**, 55–76% yields, Fig. 4b). Given that many therapeutic agents contain alkenyl alcohol moieties, we expect our methodology to provide valuable opportunities for streamlining analog generation and thereby accelerating structure-activity relationship studies in drug discovery. Finally, if an inseparable mixture of *Z*- and *E*-alkenyl amides **6h** was subjected to Cond. C, *Z*-alkene reacted smoothly to provide diene **7ha** in 75% yield, with the unreacted *E*-isomer successfully recovered (Fig. 4c).

## Discussion

In summary, we have presented a chelation-assisted geminal C(alkenyl)-H functionalization of alkenyl alcohols, carbamates and amides by palladium catalysis. Such olefinic C-H functionalization proceeds via unconventional four- to eight-membered *exo*-palladacycles and allows the alkenylation of (*Z*)-configurated and cyclic homoallyl-, bishomoallyl-, and allyl alcohols/carbamates/amides, which are particularly important features of numerous natural products and pharmaceutical agents. The protocols tolerate a wide variety of distances between the olefinic C-H bonds and the coordinating groups, enable the gram-scale preparation and modification of ricinoleate and even steroid derivatives, demonstrating the practicality and versatility. Furthermore, the carbamate and amide auxiliary are smoothly removed under mild reduction or hydrolysis conditions. This work greatly expands the utility of current C(alkenyl)-H activation reactions that are based on five- and six-membered *endo*-/*exo*-cyclometallation, and we anticipate that this method will find broad applicability in multifarious synthetic endeavors.

## Methods

### General procedure for C–H alkenylation of alcohols

An oven-dried vial was charged with Pd(OAc)_2_ (10 mol%, 0.03 mmol), Ac-Phe-OH (50 mol%, 0.15 mmol), Ag_2_CO_3_ (1.5 equiv, 0.45 mmol), Cs_2_CO_3_ (30 mol%, 0.09 mmol), CF_3_CH_2_OH (10 equiv, 3.0 mmol) and 1,4-dioxane (0.6 mL). Then, alcohol **1** (1.0 equiv, 0.3 mmol) and alkene **2** (2.0 equiv, 0.6 mmol) were added into the solution in sequence. The vial was sealed under argon and heated to 70 °C with stirring for 16–24 h. After cooling down, the mixture was concentrated to give the crude product which was directly applied to a flash column chromatography for separation (EtOAc/petroleum ether mixtures).

### General procedure for C-H alkenylation of carbamates

An oven-dried vial was charged with Pd(OAc)_2_ (10 mol%, 0.02 mmol), Ac-Gly-OH (20 mol%, 0.04 mmol), Ag_2_CO_3_ (3.0 equiv, 0.60 mmol) and CF_3_CH_2_OH (1.0 mL). Then, carbamate **4** (1.0 equiv, 0.20 mmol) and alkene **2** (2.0 equiv, 0.40 mmol) were added into the solution in sequence. The vial was sealed under argon and heated to 80 °C with stirring for 16–24 h. After cooling down, the mixture was filtered and concentrated to give the crude product which was directly applied to a flash column chromatography for separation (EtOAc/petroleum ether mixtures).

### General procedure for C-H alkenylation of amides

An oven-dried vial was charged with Pd(OAc)_2_ (10 mol%, 0.02 mmol), Ac-phe-OH (50 mol%, 0.1 mmol), Ag_2_CO_3_ (1.5 equiv, 0.3 mmol), LiOH (30 mol%, 0.06 mmol), CF_3_CH_2_OH (5.0 equiv, 1.0 mmol) and MeCN (1.0 mL). Then, amide **6** (1.0 equiv, 0.20 mmol) and alkene **2** (2.0 equiv, 0.4 mmol) were added into the solution in sequence. The vial was sealed under argon and heated to 70 °C with stirring for 16 h. After cooling down, the mixture was concentrated to give the crude product which was directly applied to a flash column chromatography for separation (EtOAc/Petroleum ether mixtures).

## Supplementary information

Supplementary Information

## Data Availability

The authors declare that all data supporting the findings of this study are available within the article and its Supplementary Information files. All data are also available from the corresponding authors upon reasonable request.

## References

[1] Wang J, Chataigner I (2012). Stereoselective alkene synthesis.

[2] Sigman MS, Werner EW (2012). Imparting catalyst control upon classical palladium-catalyzed alkenyl C-H bond functionalization reactions. Acc. Chem. Res..

[3] Shang X, Liu Z-Q (2013). Transition metal-catalyzed C_vinyl_-C_vinyl_ bond formation *via* double C_vinyl_-H bond activation. Chem. Soc. Rev..

[4] Deb A, Maiti D (2017). Emergence of unactivated olefins for the synthesis of olefinated arenes. Eur. J. Org. Chem..

[5] Tang S, Liu K, Liu C, Lei A (2015). Olefinic C–H functionalization through radical alkenylation. Chem. Soc. Rev..

[6] Engle KM, Mei TS, Wasa M, Yu JQ (2012). Weak coordination as a powerful means for developing broadly useful C-H functionalization reactions. Acc. Chem. Res..

[7] Sambiagio C (2018). Comprehensive overview of directing groups applied in metal-catalysed C-H functionalisation chemistry. Chem. Soc. Rev..

[8] Gensch T, Hopkinson MN, Glorius F, Wencel-Delord J (2016). Mild metal-catalyzed C-H activation: examples and concepts. Chem. Soc. Rev..

[9] Li G (2015). Pd(II)-catalyzed C−H functionalizations directed by distal weakly coordinating functional groups. J. Am. Chem. Soc..

[10] Leow D, Li G, Mei T-S, Yu J-Q (2012). Activation of remote meta-C-H bonds assisted by an end-on template. Nature.

[11] Xu J (2019). Sequential functionalization of *meta*-C–H and ipso-C–O bonds of phenols. J. Am. Chem. Soc..

[12] Bag S (2015). Remote para-C-H functionalization of arenes by a D-shaped biphenyl template-based assembly. J. Am. Chem. Soc..

[13] He J, Wasa M, Chan KSL, Shao Q, Yu J-Q (2017). Palladium-catalyzed transformations of alkyl C–H bonds. Chem. Rev..

[14] McNally A, Haffemayer B, Collins BSL, Gaunt MJ (2014). Palladium-catalysed C-H activation of aliphatic amines to give strained nitrogen heterocycles. Nature.

[15] Topczewski JJ, Cabrera PJ, Saper NI, Sanford MS (2016). Palladium-catalysed transannular C-H functionalization of alicyclic amines. Nature.

[16] Xia G (2019). Reversing conventional site-selectivity in C(sp^3^)–H bond activation. Nat. Chem..

[17] Liang Q-J (2017). Chelation versus non-chelation control in the stereoselective alkenyl sp^2^ C-H bond functionalization reaction. Angew. Chem. Int. Ed..

[18] Jiang B, Zhao M, Li S-S, Xu Y-H, Loh T-P (2018). Macrolide synthesis through intramolecular oxidative cross-coupling of alkenes. Angew. Chem. Int. Ed..

[19] Sun Y (2019). Additive- and ligand-free cross-coupling reactions between alkenes and alkynes by iridium catalysis. Org. Lett..

[20] Feng C, Feng D, Loh T-P (2014). Rhodium(III)-catalyzed olefinic C-H alkynylation of enamides at room temperature. Chem. Commun..

[21] Xie F, Qi Z, Yu S, Li X (2014). Rh(III)- and Ir(III)-catalyzed C-H alkynylation of arenes under chelation assistance. J. Am. Chem. Soc..

[22] Tan E, Quinonero O, de Orbe ME, Echavarren AM (2018). Broad-scope Rh-catalyzed inverse-Sonogashira reaction directed by weakly coordinating groups. ACS Catal..

[23] Aihara Y, Chatani N (2013). Nickel-catalyzed direct alkylation of C-H bonds in benzamides and acrylamides with functionalized alkyl halides via bidentate-chelation assistance. J. Am. Chem. Soc..

[24] Zhou B, Hu Y, Wang C (2015). Manganese-catalyzed direct nucleophilic C(sp^2^)-H addition to aldehydes and nitriles. Angew. Chem. Int. Ed..

[25] Yu D-G, Gensch T, Azambuja F, Vásquez-Céspedes S, Glorius F (2014). Co(III)-catalyzed C-H activation/formal SN-type reactions: selective and efficient cyanation, halogenation, and allylation. J. Am. Chem. Soc..

[26] Feng C, Loh T-P (2013). Directing-group-assisted copper-catalyzed olefinic trifluoromethylation of electron-deficient alkenes. Angew. Chem. Int. Ed..

[27] Ilies L, Asako S, Nakamura E (2011). Iron-catalyzed stereospecific activation of olefinic C-H bonds with Grignard reagent for synthesis of substituted olefins. J. Am. Chem. Soc..

[28] Zhao Y (2017). Rh/Cu-catalyzed cascade [4+2] vinylic C-H O-annulation and ring contraction of α-aryl enones with alkynes in air. Angew. Chem. Int. Ed..

[29] Shi Z, Koester DC, Boultadakis-Arapinis M, Glorius F (2013). Rh(III)-catalyzed synthesis of multisubstituted Isoquinoline and pyridine N-oxides from oximes and diazo compounds. J. Am. Chem. Soc..

[30] Piou T, Rovis T (2014). Rh(III)-catalyzed cyclopropanation initiated by C-H activation: ligand development enables a diastereoselective [2+1] annulation of N-noxyphthalimides and alkenes. J. Am. Chem. Soc..

[31] Wang D, Wang F, Song G, Li X (2012). Diverse reactivity in a rhodium(III)-catalyzed oxidative coupling of N-allyl arenesulfonamides with alkynes. Angew. Chem., Int. Ed..

[32] Morimoto M, Miura T, Murakami M (2015). Rhodium-catalyzed dehydrogenative borylation of aliphatic terminal alkenes with pinacolborane. Angew. Chem., Int. Ed..

[33] Kuppusamy R, Muralirajan K, Cheng C-H (2016). Cobalt(III)-catalyzed [5+1] annulation for 2H-chromenes synthesis via vinylic C-H activation and intramolecular nucleophilic Addition. ACS Catal..

[34] Yu C, Zhang J, Zhong G (2017). One step synthesis of γ-alkylidenebutenolides from simple vinyl carboxylic acids and alkenes. Chem. Commun..

[35] Wang K, Hu F, Zhang Y, Wang J (2015). Directing group-assisted transition-metal-catalyzed vinylic C-H bond functionalization. Sci. Chin. Chem..

[36] Kong W-J (2016). Pd-catalyzed α-selective C-H functionalization of olefins: en route to 4-imino-β-lactams. J. Am. Chem. Soc..

[37] Tsai H-C, Huang Y-H, Chou C-M (2018). Rapid access to ortho-alkylated vinylarenes from aromatic acids by dearomatization and tandem decarboxylative C-H olefination/ rearomatization. Org. Lett..

[38] Ren Z, Mo F, Dong G (2012). Catalytic functionalization of unactivated sp^3^ C-H bonds *via* exo-directing groups: synthesis of chemically differentiated 1,2-diols. J. Am. Chem. Soc..

[39] Liu M (2018). C(alkenyl)-H activation via six-membered palladacycles: catalytic 1,3-diene synthesis. J. Am. Chem. Soc..

[40] Schreib BS, Carreira EM (2019). Palladium-catalyzed regioselective C−H iodination of unactivated alkenes. J. Am. Chem. Soc..

[41] Mawo RY, Mustakim S, Young VG, Hoffmann MR, Smoliakova IP (2007). Endo-effect-driven regioselectivity in the cyclopalladation of (S)-2-tert-butyl-4-phenyl-2-oxazoline. Organometallics.

[42] Lu Y, Wang D-H, Engle KM, Yu J-Q (2010). Pd(II)-catalyzed hydroxyl-directed C-H olefination enabled by monoprotected amino acid ligands. J. Am. Chem. Soc..

[43] Nakanowatari S, Ackermann L (2014). Ruthenium(II)-catalyzed synthesis of isochromenes by C-H activation with weakly coordinating aliphatic hydroxyl groups. Chem. Eur. J..

[44] Morimoto K, Hirano K, Satoh T, Miura M (2011). Synthesis of isochromene and related derivatives by rhodium-catalyzed oxidative coupling of benzyl and allyl alcohols with alkynes. J. Org. Chem..

[45] Wang D-H, Engle KM, Shi B-F, Yu J-Q (2010). Ligand-enabled reactivity and selectivity in a synthetically versatile aryl C-H olefination. Science.

[46] Engle KM, Yu J-Q (2013). Developing ligands for palladium(II)-catalyzed C–H functionalization: intimate dialogue between ligand and substrate. J. Org. Chem..

[47] Ferreira EM, Stoltz BM (2001). The palladium-catalyzed oxidative kinetic resolution of secondary alcohols with molecular oxygen. J. Am. Chem. Soc..

[48] Mishra NK, Sharma S, Park J, Han S, Kim IS (2017). Recent advances in catalytic C(sp^2^)-H allylation reactions. ACS Catal..

[49] Shen D, Chen Q, Yan P, Zeng X, Zhong G (2017). Enantioselective dearomatization of naphthol derivatives with allylic alcohols by cooperative iridium and brønsted acid catalysis. Angew. Chem. Int. Ed..

[50] Yu J-Q, Gaunt MJ, Spencer JB (2002). Convenient preparation of trans-arylalkenes via palladium(II)-catalyzed isomerization of cis-arylalkenes. J. Org. Chem..

[51] Simmons EM, Hartwig JF (2012). On the interpretation of deuterium kinetic isotope effects in C-H bond functionalizations by transition metal complexes. Angew. Chem., Int. Ed..

