# Supplementary material for: Geminal group-directed olefinic C-H functionalization via four- to eight-membered exo-metallocycles
Source: Nat Commun. 2019 Nov 8;10:5109. doi: 10.1038/s41467-019-13098-1 (PMC6841671; doi:10.1038/s41467-019-13098-1)
Supplement: Supplementary file 1 — Supplementary Information [file 41467_2019_13098_MOESM1_ESM.pdf]

## Supplementary Information

# Geminal group-directed olefinic C-H functionalizations *via* four- to eight-membered *exo*-metallocycles

Meng *et al.*

## Supplementary Methods

Analytical thin layer chromatography (TLC) was performed using Merck 60 F254 precoated silica gel plate (0.2 mm thickness). Subsequent to elution, plates were visualized using UV radiation (254 nm) on Spectroline Model ENF-24061/F 254nm. Further visualization was possible by staining with basic solution of potassium permanganate or acidic solution of ceric molybdate. Flash column chromatography was performed using Merck aluminium oxide 90 active neutral with freshly distilled solvents. Columns were typically packed as slurry and equilibrated with the appropriate solvent system prior to use. Proton nuclear magnetic resonance ($^1H$ NMR) was recorded on Bruker AMX 500 spectrophotometer ($CDCl_3$). Chemical shifts for $^1H$ NMR spectra are reported as δ in units of parts per million (ppm) downfield from $SiMe_4$ (δ = 0.0) and relative to the signal of chloroform-*d* (δ = 7.26, singlet). Multiplicities were given as: s (singlet), d (doublet), t (triplet), dd (doublets of doublet) or m (multiplets). The number of protons (n) for a given resonance is indicated by nH. Coupling constants are reported as a *J* value in Hz. Carbon nuclear magnetic resonance spectra ($^{13}C$ NMR) are reported as δ in units of parts per million (ppm) downfield from $SiMe_4$ (δ = 0.0) and relative to the signal of chloroform-*d* (δ = 77.0, triplet). High resolution mass spectra (HRMS) was performed on Waters Q-Tof Permies Mass Spectrometer, using Electro Spray Ionization (ESI) mode. IR spectra were recorded as thin films on KBr or NaCl plates on a Bio-Rad FTS 165 FTIR spectrometer and are reported in frequency of absorption ($cm^{-1}$). $Pd(OAc)_2$ and $CF_3CH_2OH$ were purchased from TCI. Mono-*N*-protected amino acid, $Ag_2CO_3$, $Cs_2CO_3$ were purchased from Energy Chemical and they were used directly. Other reagents, unless otherwise noted below, are commercially available from Alfa Aesar (China) Chemical Co, Ltd. and used without further purification.

**Supplementary Table 1.** Optimization of olefinic C-H alkenylation of homoallyl alcohol [a]

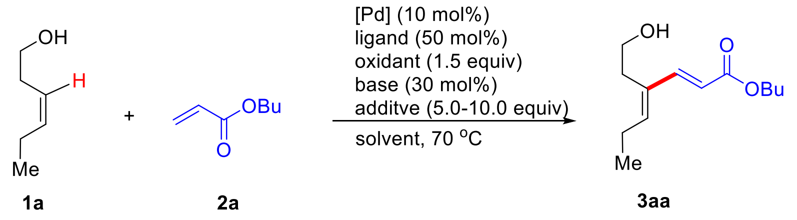


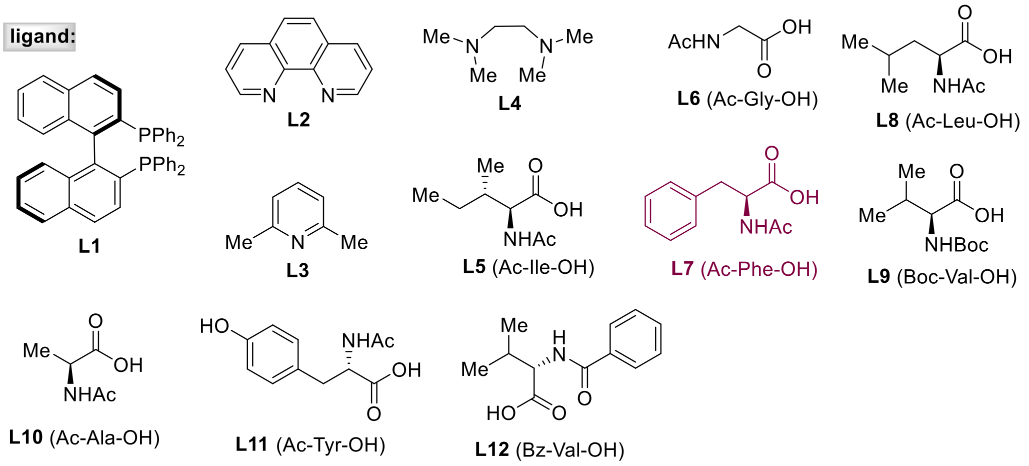


| entry | catalyst | ligand | oxidant | base | additive (eq.) | solvent | yield (%)[b] |
|---|---|---|---|---|---|---|---|
| 1 | $Pd(OAc)_2$ | - | $Ag_2CO_3$ | $Cs_2CO_3$ | $CF_3CH_2OH$ (5) | dioxane | 0 |
| 2 | $Pd(OAc)_2$ | **L1** | $Ag_2CO_3$ | $Cs_2CO_3$ | $CF_3CH_2OH$ (5) | dioxane | 0 |
| 3 | $Pd(OAc)_2$ | **L2** | $Ag_2CO_3$ | $Cs_2CO_3$ | $CF_3CH_2OH$ (5) | dioxane | 0 |
| 4 | $Pd(OAc)_2$ | **L3** | $Ag_2CO_3$ | $Cs_2CO_3$ | $CF_3CH_2OH$ (5) | dioxane | 0 |
| 5 | $Pd(OAc)_2$ | **L4** | $Ag_2CO_3$ | $Cs_2CO_3$ | $CF_3CH_2OH$ (5) | dioxane | 0 |
| 6 | $Pd(OAc)_2$ | **L5** | $Ag_2CO_3$ | $Cs_2CO_3$ | $CF_3CH_2OH$ (5) | dioxane | 34 |
| 7 | $Pd(OAc)_2$ | **L6** | $Ag_2CO_3$ | $Cs_2CO_3$ | $CF_3CH_2OH$ (5) | dioxane | 0 |
| 8 | $Pd(OAc)_2$ | **L7** | $Ag_2CO_3$ | $Cs_2CO_3$ | $CF_3CH_2OH$ (5) | dioxane | 56 |
| 9 | $Pd(OAc)_2$ | **L8** | $Ag_2CO_3$ | $Cs_2CO_3$ | $CF_3CH_2OH$ (5) | dioxane | 20 |
| 10 | $Pd(OAc)_2$ | **L9** | $Ag_2CO_3$ | $Cs_2CO_3$ | $CF_3CH_2OH$ (5) | dioxane | 6 |
| 11 | $Pd(OAc)_2$ | **L10** | $Ag_2CO_3$ | $Cs_2CO_3$ | $CF_3CH_2OH$ (5) | dioxane | 10 |
| 12 | $Pd(OAc)_2$ | **L11** | $Ag_2CO_3$ | $Cs_2CO_3$ | $CF_3CH_2OH$ (5) | dioxane | 5 |
| 13 | $Pd(OAc)_2$ | **L12** | $Ag_2CO_3$ | $Cs_2CO_3$ | $CF_3CH_2OH$ (5) | THF | 54 |
| 14 | $Pd(OAc)_2$ | **L7** | $Ag_2CO_3$ | $Cs_2CO_3$ | $CF_3CH_2OH$ (5) | DME | 46 |
| 15 | $Pd(OAc)_2$ | **L7** | $Ag_2CO_3$ | $Cs_2CO_3$ | $CF_3CH_2OH$ (5) | MeCN | 42 |
| 16 | $Pd(OAc)_2$ | **L7** | $Ag_2CO_3$ | $Cs_2CO_3$ | $CF_3CH_2OH$ (5) | DCE | 56 |
| 17 | $Pd(OAc)_2$ | **L7** | $Ag_2CO_3$ | $Cs_2CO_3$ | $CF_3CH_2OH$ (5) | DMSO | 11 |
| 18 | $Pd(OAc)_2$ | **L7** | $Ag_2CO_3$ | $Cs_2CO_3$ | $CF_3CH_2OH$ (5) | toluene | 51 |
| 19 | $Pd(OAc)_2$ | **L7** | $Ag_2CO_3$ | $Cs_2CO_3$ | $CF_3CH_2OH$ (5) | MeOH | 20 |
| 20 | $Pd(OAc)_2$ | **L7** | $Ag_2CO_3$ | $Cs_2CO_3$ | $CF_3CH_2OH$ (5) | DMF | 13 |
| 21 | $Pd(OAc)_2$ | **L7** | $Ag_2CO_3$ | $K_2CO_3$ | $CF_3CH_2OH$ (5) | dioxane | 48 |
| 22 | $Pd(OAc)_2$ | **L7** | $Ag_2CO_3$ | $Na_2CO_3$ | $CF_3CH_2OH$ (5) | dioxane | 40 |

| | | | | | | | |
|---|---|---|---|---|---|---|---|
| 23 | $Pd(OAc)_2$ | **L7** | $Ag_2CO_3$ | NaOH | $CF_3CH_2OH$ (5) | dioxane | 46 |
| 24 | $Pd(OAc)_2$ | **L7** | $Ag_2CO_3$ | $K_3PO_4$ | $CF_3CH_2OH$ (5) | dioxane | 47 |
| 25 | $Pd(OAc)_2$ | **L7** | $Ag_2CO_3$ | $K_2HPO_4$ | $CF_3CH_2OH$ (5) | dioxane | 44 |
| 26 | $Pd(OAc)_2$ | **L7** | $Ag_2CO_3$ | $KH_2PO_4$ | $CF_3CH_2OH$ (5) | dioxane | 27 |
| 27 | $Pd(OAc)_2$ | **L7** | $Ag_2CO_3$ | $Cs_2CO_3$ | $CF_3CH_2OH$ (2) | dioxane | 43 |
| 28 | $Pd(OAc)_2$ | **L7** | $Ag_2CO_3$ | $Cs_2CO_3$ | $CF_3CH_2OH$ (8) | dioxane | 57 |
| 29 | $Pd(OAc)_2$ | **L7** | $Ag_2CO_3$ | $Cs_2CO_3$ | $CF_3CH_2OH$ (10) | dioxane | 69 |
| 30 | $Pd(OAc)_2$ | **L7** | $Ag_2CO_3$ | $Cs_2CO_3$ | $CF_3CH_2OH$ (15) | dioxane | 60 |
| 31 | $Pd(OAc)_2$ | **L7** | $Ag_2CO_3$ | $Cs_2CO_3$ | HFIP (5) | dioxane | 62 |
| 32 | $Pd(OAc)_2$ | **L7** | AgOAc | $Cs_2CO_3$ | $CF_3CH_2OH$ (10) | dioxane | 31 |
| 33 | $Pd(OAc)_2$ | **L7** | $Ag_2O$ | $Cs_2CO_3$ | $CF_3CH_2OH$ (10) | dioxane | 26 |
| 34 | $Pd(OAc)_2$ | **L7** | $Cu(OAc)_2$ | $Cs_2CO_3$ | $CF_3CH_2OH$ (10) | dioxane | 15 |
| 35 | $Pd(OAc)_2$ | **L7** | $MnO_2$ | $Cs_2CO_3$ | $CF_3CH_2OH$ (10) | dioxane | 5 |
| 36 | $Pd(OAc)_2$ | **L7** | $O_2$ (1 atm) | $Cs_2CO_3$ | $CF_3CH_2OH$ (10) | dioxane | 10 |
| 37 | $PdCl_2$ | **L7** | $Ag_2CO_3$ | $Cs_2CO_3$ | $CF_3CH_2OH$ (10) | dioxane | 32 |
| 38 | $Pd(TFA)_2$ | **L7** | $Ag_2CO_3$ | $Cs_2CO_3$ | $CF_3CH_2OH$ (10) | dioxane | 35 |
| 39[c] | $Pd(OAc)_2$ | **L7** | $Ag_2CO_3$ | $Cs_2CO_3$ | $CF_3CH_2OH$ (10) | dioxane | 58 |
| 40[d] | $Pd(OAc)_2$ | **L7** | $Ag_2CO_3$ | $Cs_2CO_3$ | $CF_3CH_2OH$ (10) | dioxane | 65 |
| 41[e] | $Pd(OAc)_2$ | **L7** | $Ag_2CO_3$ | $Cs_2CO_3$ | $CF_3CH_2OH$ (10) | dioxane | 62 |
| 42[f] | $Pd(OAc)_2$ | **L7** | $Ag_2CO_3$ | $Cs_2CO_3$ | $CF_3CH_2OH$ (10) | dioxane | 61 |
| 43 | - | **L7** | $Ag_2CO_3$ | $Cs_2CO_3$ | $CF_3CH_2OH$ (10) | dioxane | 0 |
| 44 | $Pd(OAc)_2$ | **L7** | - | $Cs_2CO_3$ | $CF_3CH_2OH$ (10) | dioxane | 0 |

[a] Conditions: **1a** (0.2 mmol, 1.0 equiv), **2a** (0.4 mmol, 2.0 equiv), $Pd(OAc)_2$ (10 mmol %), ligand (50 mol%), $Ag_2CO_3$ (1.5 equiv), $CF_3CH_2OH$ (5-20 equiv) in a solvent (0.6 mL) at 70 °C for 16 h. [b] Isolated yield. [c] at 60 °C. [d] at 80 °C. [e] 24 h. [f] 48 h. DCE = 1,2-dichloroethane; EA = ethyl acetate; DMSO = dimethyl sulphoxide; DME = 1,2-dimethoxyethane; DMF = *N*,*N*-dimethylformamide.

## General procedure for olefinic C-H alkenylation of alkenyl alcohols (*Cond. A*)

An oven-dried vial was charged with $Pd(OAc)_2$ (6.7 mg, 0.03 mmol, 10 mol%), Ac-Phe-OH (31.0 mg, 0.15 mmol, 50 mol%), $Ag_2CO_3$ (124.1 mg, 0.45 mmol, 1.5 equiv), $Cs_2CO_3$ (29.3 mg, 0.09 mmol, 30 mol%,), $CF_3CH_2OH$ (217 µL, 3.0 mmol, 10.0 equiv) and 1,4-dioxane (0.6 mL). Then, alcohol **1** (0.3 mmol, 1.0 equiv) and alkene **2** (0.6 mmol, 2.0 equiv) were added into the solution in sequence. The vial was sealed under argon and heated to 70 °C with stirring for 16 hours. After cooling down, the mixture was concentrated to give the crude product which was directly applied to a flash column chromatography for purification (ethyl acetate/petroleum ether mixtures).

**Supplementary Table 2.** Optimization of olefinic C-H alkenylation of allyl carbamate [a]

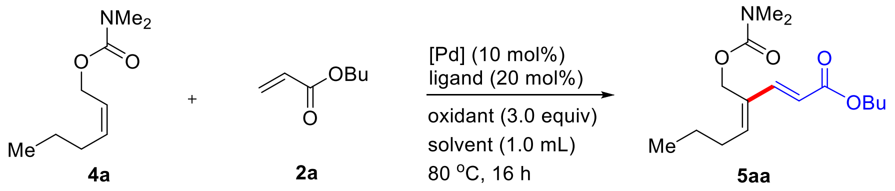


| Entry | catalyst | ligand | oxidant | solvent | yield (%) |
|---|---|---|---|---|---|
| 1 | $Pd(OAc)_2$ | Ac-Gly-OH | $Ag_2CO_3$ | DCE | 17 |
| 2 | $Pd(OAc)_2$ | Ac-Gly-OH | $Ag_2CO_3$ | DMF | 28 |
| 3 | $Pd(OAc)_2$ | Ac-Gly-OH | $Ag_2CO_3$ | MeCN | 14 |
| 4 | $Pd(OAc)_2$ | Ac-Gly-OH | $Ag_2CO_3$ | DMSO | 15 |
| 5 | $Pd(OAc)_2$ | Ac-Gly-OH | $Ag_2CO_3$ | $CH_3CH_2OH$ | 56 |
| 6 | $Pd(OAc)_2$ | Ac-Gly-OH | $Ag_2CO_3$ | HFIP | 24 |
| 7 | $Pd(OAc)_2$ | Ac-Gly-OH | $Ag_2CO_3$ | THF | 10 |
| 8 | $Pd(OAc)_2$ | Ac-Gly-OH | $Ag_2CO_3$ | $CF_3COCH_3$ | 17 |
| 9 | $Pd(OAc)_2$ | Ac-Gly-OH | $Ag_2CO_3$ | DME | < 5 |
| 10 | $Pd(OAc)_2$ | Ac-Gly-OH | $Ag_2CO_3$ | 1,4-dioxane | 13 |
| 11[b] | $Pd(OAc)_2$ | Ac-Gly-OH | $Ag_2CO_3$ | $CH_3CH_2OH$ | 16 |
| 12[c] | $Pd(OAc)_2$ | Ac-Gly-OH | $Ag_2CO_3$ | $CH_3CH_2OH$ | 23 |
| 13[d] | $Pd(OAc)_2$ | Ac-Gly-OH | $Ag_2CO_3$ | $CH_3CH_2OH$ | 37 |
| 14 | $Pd(OAc)_2$ | Ac-Gly-OH | AgOAc | $CH_3CH_2OH$ | 33 |
| 15 | $Pd(OAc)_2$ | Ac-Gly-OH | $O_2$ (1 atm) | $CH_3CH_2OH$ | 10 |
| 16 | $Pd(OAc)_2$ | Ac-Gly-OH | $Cu(OAc)_2$ | $CH_3CH_2OH$ | < 5 |
| 17 | $Pd(OAc)_2$ | Ac-Gly-OH | $CuCl_2 \cdot H_2O$ | $CH_3CH_2OH$ | 0 |
| 18 | $PdCl_2$ | Ac-Gly-OH | $Ag_2CO_3$ | $CH_3CH_2OH$ | 13 |
| 19 | $Pd(TFA)_2$ | Ac-Gly-OH | $Ag_2CO_3$ | $CH_3CH_2OH$ | 20 |
| 20 | $Pd(OAc)_2$ | Ac-Leu-OH | $Ag_2CO_3$ | $CH_3CH_2OH$ | 17 |
| 21 | $Pd(OAc)_2$ | Ac-Ala-OH | $Ag_2CO_3$ | $CH_3CH_2OH$ | 32 |
| 22 | $Pd(OAc)_2$ | Ac-Ile-OH | $Ag_2CO_3$ | $CH_3CH_2OH$ | 7 |
| 23 | $Pd(OAc)_2$ | Ac-Phe-OH | $Ag_2CO_3$ | $CH_3CH_2OH$ | 13 |
| 24 | $Pd(OAc)_2$ | Boc-Val-OH | $Ag_2CO_3$ | $CH_3CH_2OH$ | < 5 |
| 25[e] | $Pd(OAc)_2$ | Ac-Gly-OH | $Ag_2CO_3$ | $CH_3CH_2OH$ | 17 |
| 26[f] | $Pd(OAc)_2$ | Ac-Gly-OH | $Ag_2CO_3$ | $CH_3CH_2OH$ | 32 |

| | | | | | |
|---|---|---|---|---|---|
| 27[g] | $Pd(OAc)_2$ | Ac-Gly-OH | $Ag_2CO_3$ | $CH_3CH_2OH$ | 47 |

[a] Unless otherwise noted, the reactions were carried out using alcohol **4a** (0.2 mmol), acrylate **2a** (0.4 mmol), $Pd(OAc)_2$ (10.0 mol%), ligand (20 mmol%), oxidant (3.0 equiv) in a solvent (1.0 mL) at 80 °C for 16 h under an argon atmosphere (1 atm). The yields indicated in the table are isolated yields. [b] At 40 °C. [c] At 60 °C. [d] At 100 °C. [e] 6 h. [f] 8 h. [g] 10 h. DCE = 1,2-dichloroethane; EA = ethyl acetate; DMSO = dimethyl sulphoxide; DME = 1,2-dimethoxyethane; DMF = *N*,*N*-dimethylformamide.

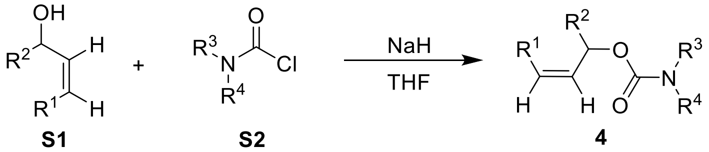


A solution of alcohol (1.0 mmol, 1.0 equiv) in THF (2.0 mL) was added dropwisly to a suspension of sodium hydride (60% in mineral oil) (1.2 mmol, 1.2 equiv) in THF (0.5 mL) at 0 °C. After stirring at r.t. for 1 h, carbamoyl chloride (1.4 mmol, 1.4 equiv) was added over a period of 15 min. The mixture was heated to refluxed for 3 h and cooled down to r.t. before it was poured into a 1 mol/L HCl solution (aq., 2 mL). After separation of the organic phase, the aqueous layer was extracted three times with ethyl acetate (2 mL) and the combined organic phases were washed with a saturated aqueous $NaHCO_3$ solution (2 mL). After drying over $Na_2SO_4$ and evaporation of the solvent under vacuum, the crude product was purified by column chromatography. [1]

## General procedure for Olefinic C-H Alkenylation of Alkenyl Carbamates (*Cond. B*)

An oven-dried screw-cap vial was charged with $Pd(OAc)_2$ (4.5 mg, 10 mol%, 0.02 mmol), Ac-Gly-OH (4.7 mg, 20 mol%, 0.04 mmol), $Ag_2CO_3$ (165.5 mg, 3.0 equiv, 0.6 mmol) and $CF_3CH_2OH$ (1.0 mL). Then, carbamate **4** (0.2 mmol, 1.0 equiv) and alkene **2** (0.40 mmol, 2.0 equiv) were added into the solution in sequence. The vial was sealed under nitrogen and heated to 80 °C with stirring for 16 hours. After cooling down, the mixture was filtered and concentrated to give the crude product which was directly applied to a flash column chromatography (ethyl acetate/petroleum ether mixtures).

**Supplementary Table 3.** Optimization of olefinic C-H alkenylation of alkenyl amides [a]

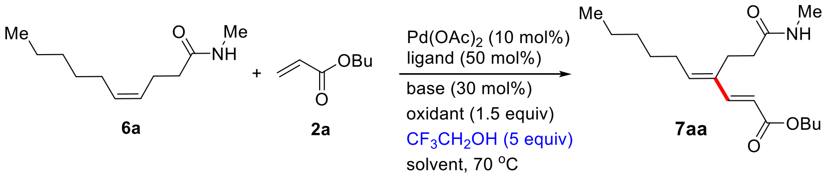


| entry | ligand | base (mol%) | additive (equiv) | oxidant (equiv) | solvent | yield (%)[b] |
|---|---|---|---|---|---|---|
| 1 | Ac-Phe-OH | $Cs_2CO_3$ (30) | $CF_3CH_2OH$ (10) | $Ag_2CO_3$ (1.5) | dioxane | 37 |
| 2 | Ac-Phe-OH | $Cs_2CO_3$ (50) | $CF_3CH_2OH$ (10) | $Ag_2CO_3$ (1.5) | dioxane | 8 |
| 3 | Ac-Phe-OH | $K_2CO_3$ (30) | $CF_3CH_2OH$ (10) | $Ag_2CO_3$ (1.5) | dioxane | 13 |
| 4 | Ac-Phe-OH | $Na_2CO_3$ (30) | $CF_3CH_2OH$ (10) | $Ag_2CO_3$ (1.5) | dioxane | 18 |
| 5 | Ac-Phe-OH | NaOH (30) | $CF_3CH_2OH$ (10) | $Ag_2CO_3$ (1.5) | dioxane | 50 |
| 6 | Ac-Phe-OH | LiOH (30) | $CF_3CH_2OH$ (10) | $Ag_2CO_3$ (1.5) | dioxane | 51 |
| 7 | Ac-Phe-OH | CsOH (30) | $CF_3CH_2OH$ (10) | $Ag_2CO_3$ (1.5) | dioxane | 40 |
| 8 | Ac-Phe-OH | LiOH (30) | $CF_3CH_2OH$ (5) | $Ag_2CO_3$ (1.5) | MeCN | 74 |
| 9 | Ac-Phe-OH | LiOH (30) | $CF_3CH_2OH$ (5) | $Ag_2CO_3$ (1.5) | MeOH | 43 |
| 10 | Ac-Phe-OH | LiOH (30) | $CF_3CH_2OH$ (5) | $Ag_2CO_3$ (1.5) | DMF | 10 |
| 11 | Ac-Phe-OH | LiOH (30) | $CF_3CH_2OH$ (5) | $Ag_2CO_3$ (1.5) | acetone | 17 |
| 12 | Ac-Ile-OH | LiOH (30) | $CF_3CH_2OH$ (5) | $Ag_2CO_3$ (1.5) | MeCN | 46 |
| 13 | Ac-Leu-OH | LiOH (30) | $CF_3CH_2OH$ (5) | $Ag_2CO_3$ (1.5) | MeCN | 47 |
| 14 | Ac-Val-OH | LiOH (30) | $CF_3CH_2OH$ (5) | $Ag_2CO_3$ (1.5) | MeCN | 52 |
| 15 | Ac-Gly-OH | LiOH (30) | $CF_3CH_2OH$ (5) | $Ag_2CO_3$ (1.5) | MeCN | 0 |
| 16 | PivOH | LiOH (30) | $CF_3CH_2OH$ (5) | $Ag_2CO_3$ (1.5) | MeCN | 0 |
| 17 | 2,2'-bipyridyl | LiOH (30) | $CF_3CH_2OH$ (5) | $Ag_2CO_3$ (1.5) | MeCN | 0 |
| 18 | TMEDA | LiOH (30) | $CF_3CH_2OH$ (5) | $Ag_2CO_3$ (1.5) | MeCN | 0 |
| 19 | 1,10-phen | LiOH (30) | $CF_3CH_2OH$ (5) | $Ag_2CO_3$ (1.5) | MeCN | 0 |
| 20 | Ac-Phe-OH | LiOH (30) | $CF_3CH_2OH$ (5) | AgTFA (1.5) | MeCN | 20 |

[a] Reaction conditions: **6a** (0.2 mmol), **2a** (0.4 mmol), $Pd(OAc)_2$ (10 mol%), ligand (50 mol%), base (30 mol%), oxidant (1.5 equiv), $CF_3CH_2OH$ (5 equiv), in MeCN (1 mL) at 70 °C for 16 h. [b] Isolated yields. [c] PivOH (4.0 equiv) was added. [d] AcOH (4.0 equiv) was added. [e] AcOH (0.5 equiv) was added.

## General Procedure for Olefinic C-H Alkenylation of Alkenyl Amides (*Cond. C*)

An oven-dried screw-cap vial was charged with $Pd(OAc)_2$ (10.0 mol%, 0.02 mmol), Ac-phe-OH (50.0 mol%, 0.1 mmol), $Ag_2CO_3$ (1.5 eq., 0.3 mmol), LiOH (30 mol%, 0.06 mmol), $CF_3CH_2OH$ (5.0 equiv, 1.0 mmol) and MeCN (1.0 mL). Then, amide **6** (1.0 equiv, 0.20 mmol) and alkene **2** (2.0 equiv, 0.4 mmol) were added into the solution in sequence. The vial was sealed under argon and heated to 70 ℃ with stirring for 16 hours. After cooling down, the mixture was concentrated to give the crude product

which was directly applied to a flash column chromatography (EtOAc/Petroleum ether mixtures) for separation.

## Characterization of products

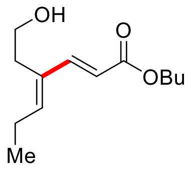


**Butyl (2E, 4E)-4-(2-hydroxyethyl) hepta-2,4-dienoate (3aa)**. Yellow oil, yield = 69%. Reaction time: 16 h. $^{1}H$ NMR (500 MHz, $CDCl_3$): δ = 7.26 (d, *J* = 16.5 Hz, 1H), 6.01 (t, *J* = 7.5 Hz, 1H), 5.85 (d, *J* = 16.0 Hz, 1H ), 4.15 (t, *J* = 7.0 Hz, 2H), 3.65 (t, *J* = 7.0 Hz, 2H), 2.55 (t, *J* = 7.0 Hz, 2H), 2.23-2.29 (m, 2H), 1.62-1.68 (m, 2H), 1.37-1.44 (m, 2H), 1.03-1.06 (m, 3H), 0.93-0.96 (m, 3H). $^{13}C$ NMR (125 MHz, $CDCl_3$): δ = 167.61, 148.45, 146.27, 132.64, 115.66, 64.23, 61.16, 30.77, 29.97, 22.24, 19.18, 13.72, 13.69. HR-MS (ESI): m/z calculated for $C_{13}H_{22}O_3$ $[M+H]^+$: 227.1642, found: 227.1642. FTIR (KBr, $cm^{-1}$): 3786.32, 1731.91, 1703.94, 1519.16, 1514.97, 1486.51, 1416.54, 1385.07, 1172.81, 982.04.

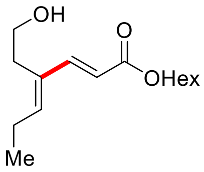


**Hexyl (2E, 4E)-4-(2-hydroxyethyl) hepta-2,4-dienoate (3ab)**. Yellow oil, yield = 75%. Reaction time: 16 h. $^{1}H$ NMR (500 MHz, $CDCl_3$): δ = 7.19 (d, *J* = 16.0 Hz, 1H), 5.94 (t, *J* = 7.5 Hz, 1H), 5.79 (d, *J* = 16.0 Hz, 1H), 4.06 (t, *J* = 7.0 Hz, 2H), 3.59 (t, *J* = 7.0 Hz, 2H), 2.49 (t, *J* = 7.0 Hz, 2H), 2.16-2.21 (m, 2H), 1.56-1.61 (m, 2H), 1.22-1.32 (m, 6H), 0.97 (t, *J* = 7.5 Hz, 3H), 0.83 (t, *J* = 7.0 Hz, 3H). $^{13}C$ NMR (125 MHz, $CDCl_3$): δ = 166.65, 147.49, 145.24, 131.67, 114.62, 63.55, 60.12, 30.46, 28.98, 27.68, 24.63, 21.53, 21.23, 12.99, 12.68. HR-MS (ESI): m/z calculated for $C_{15}H_{26}O_3$ $[M+H]^+$: 255.1965, found: 255.1983. FTIR (KBr, $cm^{-1}$): 3478.88, 3444.52, 1789.89, 1633.80, 2022.38, 1841.99, 1455.27, 1621.43, 1416.18, 986.64.

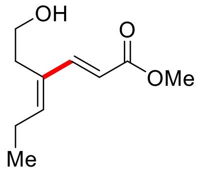


**Methyl (2E, 4E)-4-(2-hydroxyethyl) hepta-2,4-dienoate (3ac)**. Yellow oil, yield = 73%. Reaction time: 16 h. $^{1}H$ NMR (500 MHz, $CDCl_3$): δ = 7.26 (d, *J* = 15.5 Hz, 1H), 5.99 (t, *J* = 7.5 Hz, 1H), 5.84 (d, *J* = 15.5 Hz, 1H), 3.73 (s, 3H), 3.62-3.65 (m, 2H), 2.54 (t, *J* = 7.0 Hz, 2H), 2.21-2.27 (m, 2H), 1.01-1.04 (m, 3H). $^{13}C$ NMR (125 MHz, $CDCl_3$): δ = 166.94, 147.78, 145.51, 131.63, 114.18, 60.14, 50.52, 28.95, 21.25, 12.67. HR-MS (ESI): m/z calculated for $C_{10}H_{16}O_3$ $[M+H]^+$: 185.1172, found: 185.1167. FTIR (KBr, $cm^{-1}$): 3550.18, 3417.84, 1760.01, 1714.31, 1416.76, 1392.94, 1296.18, 1172.03, 983.17, 846.40.

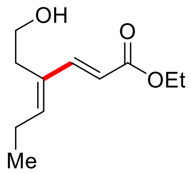


**Ethyl (2E, 4E)-4-(2-hydroxyethyl) hepta-2,4-dienoate (3ad)**. Yellow oil, yield = 81%. Reaction time: 16 h. $^{1}H$ NMR (500 MHz, $CDCl_3$): δ = 7.19 (d, *J* = 16.0 Hz, 1H), 5.93 (t, *J* = 7.0 Hz, 1H), 5.79 (d, *J* = 16.0 Hz, 1H), 4.11-4.15 (m, 2H), 3.58 (t, *J* = 7.0 Hz, 2H), 2.48-2.51 (t, *J* = 7.0 Hz, 2H), 2.15-2.21 (m, 2H), 1.21-1.24 (m, 3H), 0.95-0.99 (m, 3H). $^{13}C$ NMR (125 MHz, $CDCl_3$): δ = 166.57, 147.55, 145.29, 131.67, 114.59, 60.11, 59.30, 28.98, 21.23, 13.28, 12.68. HR-MS (ESI): m/z calculated for $C_{11}H_{18}O_3$ $[M+H]^+$: 199.1329, found: 199.1320. FTIR (KBr, $cm^{-1}$): 3542.50, 3533.73, 3479.47, 2357.86, 2335.82, 1505.22, 1445.94, 1171.15, 667.65.

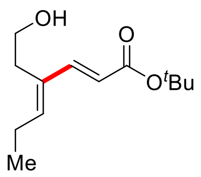


**tert-Butyl (2E,4E)-4-(2-hydroxyethyl) hepta-2,4-dienoate (3ae)**. Yellow oil, yield = 67%. Reaction time: 16 h. $^{1}$H NMR (500 MHz, $CDCl_3$): δ = 7.16 (d, *J* = 16.0 Hz, 1H), 5.97 (t, *J* = 7.5 Hz, 1H), 5.76 (d, *J* = 15.5 Hz, 1H), 3.64 (t, *J* = 7.0 Hz, 2H), 2.54 (t, *J* = 7.0 Hz, 2H), 2.20-2.56 (m, 2H), 1.48 (s, 9H), 1.00-1.03 (m, 3H). $^{13}$C NMR (125 MHz, $CDCl_3$): δ = 165.83, 146.41, 144.6, 131.55, 116.54, 79.18, 60.20, 28.99, 27.18, 21.18, 12.73. HR-MS (ESI): m/z calculated for $C_{13}H_{22}O_3$ $[M+H]^+$: 227.1642, found: 227.1646. FTIR (KBr, $cm^{-1}$): 3542.63, 3500.34, 3417.81, 1714.26, 1704.10, 1673.67, 1667.58, 1506.29, 1416.77, 1403.21, 1295.36, 1152.29.

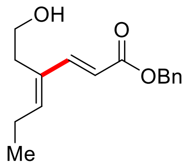


**Benzyl (2E, 4E)-4-(2-hydroxyethyl) hepta-2, 4-dienoate (3af)**. Yellow oil, yield = 39%. Reaction time: 16 h. $^{1}$H NMR (500 MHz, $CDCl_3$): δ = 7.19-7.31 (m, 6H), 5.95 (t, *J* = 7.5 Hz, 1H), 5.84 (d, *J* = 16.0 Hz, 1H), 5.12 (s, 2H), 3.58 (t, *J* = 7.0 Hz, 2H), 2.48 (t, *J* = 7.0 Hz, 2H), 2.15-2.21 (m, 2H), 0.96 (t, *J* = 7.5 Hz, 3H). $^{13}$C NMR (125 MHz, $CDCl_3$): δ = 166.23, 148.03, 145.74, 135.12, 131.61, 127.55, 127.24, 127.18, 114.28, 65.17, 60.14, 28.91, 21.27, 12.66. HR-MS (ESI): m/z calculated for $C_{16}H_{20}O_3$ $[M+H]^+$: 261.1485, found: 261.1474. FTIR (KBr, $cm^{-1}$): 3507.51, 3417.34, 1770.32, 1714.67, 1621.60, 1385.53, 1258.80, 1020.55, 802.74.

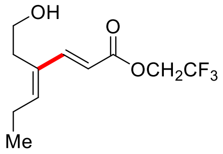


**2, 2, 2-Trifluoroethyl (2E,4E)-4-(2-hydroxyethyl) hepta-2,4-dienoate (3ag)**. Yellow oil, yield = 44%. Reaction time: 16 h. $^{1}$H NMR (500 MHz, $CDCl_3$): δ = 7.36 (d, *J* = 16.0 Hz, 1H), 6.08 (t, *J* = 7.5 Hz, 1H), 5.90 (d, *J* = 16.0 Hz, 1H), 4.53 (q, *J* = 8.5 Hz, 2H), 3.67 (t, *J* = 7.0 Hz, 2H), 2.57 (t, *J* = 7.0 Hz, 2H), 2.25-2.31 (m, 2H), 1.05 (t, *J* = 7.5 Hz, 3H). $^{13}$C NMR (125 MHz, $CDCl_3$): δ = 164.62, 150.06, 147.28, 131.66, 122.12 (q, $J_{C-F}$ = 275.6 Hz), 112.35, 60.10, 59.24 (q, $J_{C-F}$ = 36.3 Hz), 28.83, 21.39, 12.56. HR-MS (ESI): m/z calculated for $C_{11}H_{15}F_3O_3$ $[M+H]^+$: 253.1046, found: 253.1041. FTIR (KBr, $cm^{-1}$): 3654.05, 3500.11, 3417.70, 1841.92, 1698.41, 1360.26, 1257.70, 987.79.

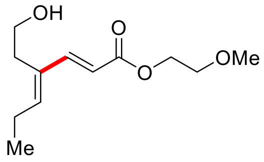


**2-Methoxyethyl (2E,4E)-4-(2-hydroxyethyl) hepta-2,4-dienoate (3ah)**. Yellow oil, yield = 78%. Reaction time: 16 h. $^{1}$H NMR (500 MHz, $CDCl_3$): δ = 7.23 (d, *J* = 16.0 Hz, 1H), 5.96 (t, *J* = 7.5 Hz, 1H), 5.84 (d, *J* = 16.0 Hz, 1H), 4.23 (t, *J* = 4.0 Hz, 2H), 3.56-3.59 (m, 4H), 3.34 (s, 3H), 2.48 (t, *J* = 7.0 Hz, 2H), 2.16-2.22 (m, 2H), 0.97 (t, *J* = 7.5 Hz, 3H). $^{13}$C NMR (125 MHz, $CDCl_3$): δ = 166.43, 148.05, 145.68, 131.65, 114.18, 69.58, 62.34, 60.10, 57.99, 28.95, 21.26, 12.66. HR-MS (ESI): m/z calculated for $C_{12}H_{20}O_4$ $[M+H]^+$: 229.1434, found: 229.1430. FTIR (KBr, $cm^{-1}$): 3507.52, 3479.32, 3459.67, 2395.83, 1661.57, 1372.53, 1337.55, 985.06.

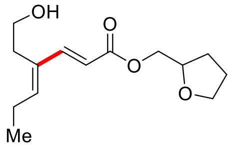


**(Tetrahydrofuran-2-yl) methyl (2E,4E)-4-(2-hydroxyethyl) hepta-2,4-dienoate (3ai)**. Yellow oil, yield = 73%. Reaction time: 16 h. $^{1}$H NMR (500 MHz, $CDCl_3$): δ = 7.26 (d, *J* = 15.5 Hz, 1H), 5.99 (t, *J* = 7.5 Hz, 1H), 5.87 (d, *J* = 16.0 Hz, 1H), 4.20 (dd, *J* = 3.5 Hz, *J* = 11.5 Hz, 1H), 4.12-4.14 (m, 1H), 4.04 (dd, *J* = 7.0 Hz, *J* = 11.5 Hz, 1H), 3.87 (dd, *J* = 7.0 Hz, *J* = 15.0 Hz, 1H), 3.76-3.80 (m, 1H), 3.60 (t, *J* = 7.0 Hz, 2H), 2.52 (t, *J* = 7.0 Hz , 2H), 2.22 (t, *J* = 7.5 Hz, 2H), 1.85-2.01 (m, 4H), 0.99-1.03 (m, 3H). $^{13}$C NMR (125 MHz, $CDCl_3$): δ = 166.42, 148.03, 145.62, 131.68, 114.19, 75.65, 67.43, 65.39, 60.07, 28.97, 26.99, 24.64, 21.25, 12.67. HR-MS (ESI): m/z calculated for

$C_{14}H_{22}O_4$ $[M+H]^+$: 255.1591, found: 255.1581. FTIR (KBr, $cm^{-1}$): 3654.35, 1714.54, 1682.50, 1505.51, 1482.85, 1372.85, 1295.26, 1169.90.

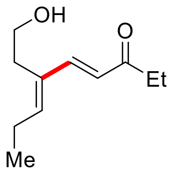


**(4E,6E)-6-(2-hydroxyethyl) nona-4,6-dien-3-one (3aj)**. Yellow oil, yield = 55%. Reaction time: 16 h. $^1$H NMR (500 MHz, $CDCl_3$): δ = 7.14 (d, *J* = 16.5 Hz, 1H), 6.15 (d, *J* = 16.0 Hz, 1H), 6.02-6.06 (m, 1H), 3.64 (t, *J* = 7.0 Hz, 2H), 2.55-2.61 (m, 4H), 2.23-2.28 (m, 2H), 1.02-1.12 (m, 6H). $^{13}$C NMR (125 MHz, $CDCl_3$): δ = 201.54, 147.15, 146.44, 132.99, 123.89, 61.20, 33.73, 29.94, 22.40, 13.68, 8.32. HR-MS (ESI): m/z calculated for $C_{11}H_{18}O_2$ $[M+H]^+$: 183.1380, found: 183.1374. FTIR (KBr, $cm^{-1}$): 3828.35, 2434.28, 2398.23, 1770.44, 1577.34, 1520.03, 1417.34, 1428.12, 1403.60, 985.49.

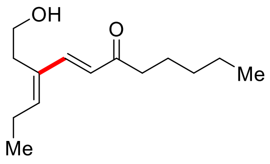


**(3E,5E)-4-(2-hydroxyethyl) dodeca-3,5-dien-7-one (3ak)**. Yellow oil, yield = 54%. Reaction time: 16 h. $^1$H NMR (500 MHz, $CDCl_3$): δ = 7.11-7.14 (m, 1H), 6.15 (d, *J* = 16.0 Hz, 1H), 6.03 (t, *J* = 7.0 Hz, 1H), 3.63 (t, *J* = 7.0 Hz, 2H), 2.52-2.58 (m, 4H), 2.24-2.27 (m, 2H), 1.61 (t, *J* = 7.0 Hz, 2H), 1.29-1.30 (m, 4H), 1.02-1.05 (m, 3H), 0.86-0.89 (m, 3H). $^{13}$C NMR (125 MHz, $CDCl_3$): δ = 200.30, 146.19, 145.53, 132.01, 123.15, 60.19, 39.63, 30.51, 28.96, 23.16, 21.47, 21.40, 12.93, 12.68. HR-MS (ESI): m/z calculated for $C_{14}H_{24}O_2$ $[M+H]^+$: 225.1849, found: 225.1847. FTIR (KBr, $cm^{-1}$): 3654.28, 3354.70, 2020.28, 1557.40, 1505.35, 1393.07, 1360.46, 987.93.

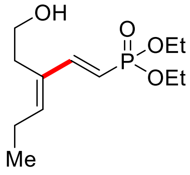


**Diethyl ((1E, 3E)-3-(2-hydroxyethyl) hexa-1,3-dien-1-yl) phosphonate (3al)** Yellow oil, yield = 75%. Reaction time: 16 h. $^1$H NMR (500 MHz, $CDCl_3$): δ = 6.97-7.07 (m, 1H), 5.91 (t, *J* = 7.0 Hz, 1H), 5.56 (td, *J* = 3.5 Hz, *J* = 18.0 Hz, 1H), 4.03-4.07 (m, 4H), 3.57-3.63 (m, 2H), 2.51-2-55 (m, 2H), 2.19-2.24 (m, 2H), 1.28-1.32 (m, 6H), 0.98-1.93 (m, 3H). $^{13}$C NMR (125 MHz, $CDCl_3$): δ = 151.54 (d, $J_{C\text{-}P}$ = 5.0 Hz), 144.56, 132.12 (d, $J_{C\text{-}P}$ = 22.5 Hz), 109.57 (d, $J_{C\text{-}P}$ = 191.3 Hz), 60.73, 60.69, 59.99, 28.75, 21.15, 15.39, 15.34, 12.68. HR-MS (ESI): m/z calculated for $C_{12}H_{23}O_4P$ $[M+H]^+$: 263.1407, found: 263.1403. FTIR (KBr, $cm^{-1}$): 3654.34, 3444.53, 3418.18, 2225.34, 1770.27, 1455.31, 1332.28, 1232.70, 1026.55.

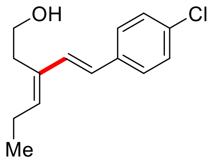


**(E)-3-((E)-2-(4-Cl-phenylethenyl)) hex-3-en-1-ol (3am)** Yellow oil, yield = 54%. Reaction time: 16 h. $^1$H NMR (500 MHz, $CDCl_3$): δ = 7.18-7.24 (m, 4H), 6.62 (d, *J* = 16.0 Hz, 1H), 6.38 (d, *J* = 16.5 Hz, 1H), 5.69 (t, *J* = 7.5 Hz, 1H), 3.65 (t, *J* = 7.0 Hz, 2H), 2.58 (t, *J* = 7.0 Hz, 2H), 2.14-2.20 (m, 2H), 0.96 (t, *J* = 7.5 Hz, 3H). $^{13}$C NMR (125 MHz, $CDCl_3$): δ = 138.03, 135.20, 132.28, 132.18, 131.53, 127.70, 126.69, 123.47, 60.60, 29.00, 20.89, 13.17. HR-MS (ESI): m/z calculated for $C_{14}H_{17}ClO$ $[M-H_2O]+H]^+$: 219.0914, found: 219.0928. FTIR (KBr, $cm^{-1}$): 3417.81, 3331.79, 1732.22, 1661.67, 1621.68, 1557.31, 1505.40, 1258.25, 1088.67, 1012.38, 805.60, 422.27.

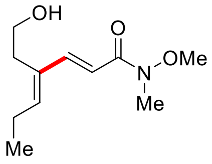


**(2E,4E)-4-(2-Hydroxyethyl)-*N*-methoxy-*N*-methyl hepta-2,4-dienamide (3an)**. Yellow oil, yield = 65%. Reaction time: 16 h. $^1$H NMR (500 MHz, $CDCl_3$): δ = 7.29 (d, *J* = 15.5 Hz, 1H), 6.43 (d, *J* = 15.5 Hz, 1H), 6.00 (t, *J* = 7.5 Hz, 1H), 3.69 (s, 3H), 3.66 (t, *J* = 7.0 Hz, 2H), 3.25 (s, 3H), 2.59 (t, *J* = 7.0 Hz, 2H), 2.21-2.27 (m, 2H), 1.01 (t, *J* = 7.5 Hz, 3H). $^{13}$C NMR (125 MHz, $CDCl_3$): δ = 166.54, 146.36, 144.58, 131.92, 112.12, 60.71, 60.34, 31.50, 29.21, 21.19, 12.75. HR-MS (ESI): m/z

calculated for $C_{11}H_{19}NO_3$ $[M+H]^+$: 214.1438, found: 214.1440. FTIR (KBr, $cm^{-1}$): 3654.39, 3472.87, 1704.16, 1698.63, 1694.38, 1557.41, 1442.85, 1403.31, 1392.46, 1179.40, 1002.51.

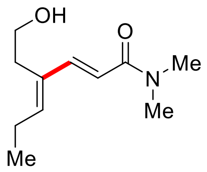


**(2E,4E)-4-(2-Hydroxyethyl)-*N*,*N*-dimethyl hepta-2,4-dienamide (3ao)**. Yellow oil, yield = 71%. Reaction time: 16 h. $^1$H NMR (500 MHz, $CDCl_3$): δ = 7.20 (d, *J* = 15.0 Hz, 1H), 6.28 (d, *J* = 15.0 Hz, 1H), 5.93 (t, *J* = 7.5 Hz, 1H), 3.63 (t, *J* = 7.0 Hz, 2H), 3.07 (s, 3H), 2.99 (s, 3H), 2.57 (t, *J* = 7.0 Hz, 2H), 2.18-2.24 (m, 2H), 1.00 (t, *J* = 7.5 Hz, 3H). $^{13}$C NMR (125 MHz, $CDCl_3$): δ = 166.43, 145.25, 143.46, 131.86, 113.76, 60.37, 36.42, 34.85, 29.27, 21.09, 12.79. HR-MS (ESI): m/z calculated for $C_{11}H_{19}NO_2$ $[M+H]^+$: 198.1489, found: 198.1492. FTIR (KBr, $cm^{-1}$): 3654.23, 3550.31, 3500.31, 3417.93, 1842.04, 1557.30, 1428.03, 1385.30, 1360.47, 979.54.

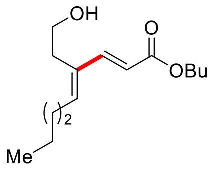


**Butyl (2E,4E)-4-(2-hydroxyethyl) nona-2,4-dienoate (3ba)** Yellow oil, yield = 73%. Reaction time: 16 h. $^1$H NMR (500 MHz, $CDCl_3$): δ = 7.19 (d, *J* = 16.5 Hz, 1H), 5.94 (t, *J* = 7.5 Hz, 1H), 5.78 (d, *J* = 16.0 Hz, 1H), 4.06-4.09 (m, 2H), 3.58 (t, *J* = 7.0 Hz, 2H), 2.50 (t, *J* = 7.0 Hz, 2H), 2.17 (q, *J* = 7.5 Hz, 2H), 1.55-1.61 (m, 2H), 1.26-1.36 (m, 6H), 0.82-0.89 (m, 6H). $^{13}$C NMR (125 MHz, $CDCl_3$): δ = 166.67, 147.51, 143.97, 132.10, 114.50, 63.23, 60.14, 30.35, 29.77, 29.05, 27.66, 21.43, 18.18, 12.91, 12.72. HR-MS (ESI): m/z calculated for $C_{15}H_{26}O_3$ $[M+H]^+$: 255.1955, found: 255.1958. FTIR (KBr, $cm^{-1}$): 3830.09, 3654.80, 3573.47, 3380.88, 1770.25, 1663.87, 1416.16, 1403.23.

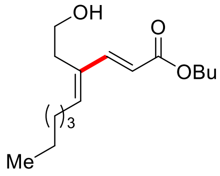


**Butyl (2E,4E)-4-(2-hydroxyethyl) deca-2,4-dienoate (3ca)**. Yellow oil, yield = 69%. Reaction time: 16 h. $^1$H NMR (500 MHz, $CDCl_3$): δ = 7.18-7.22 (m, 1H), 5.95 (q, *J* = 7.0 Hz, 1H), 5.79 (dd, *J* = 3.0 Hz, *J* = 15.5 Hz, 1H), 4.06-4.10 (m, 2H), 3.59 (q, *J* = 7.0 Hz, 2H), 2.48-2.52 (m, 2H), 2.14-2.19 (m, 2H), 1.55-1.60 (m, 2H), 1.33-1.37 (m, 4H), 1.23-1.25 (m, 4H), 0.86-0.90 (m, 3H), 0.80-0.83 (m, 3H). $^{13}$C NMR (125 MHz, $CDCl_3$): δ = 166.62, 147.46, 144.04, 132.05, 114.54, 63.22, 60.17, 30.53, 29.77, 29.05, 27.92, 27.88, 21.50, 18.18, 12.97, 12.72. HR-MS (ESI): m/z calculated for $C_{16}H_{28}O_3$ $[M+H]^+$: 269.2111, found: 269.2117. FTIR (KBr, $cm^{-1}$): 3382.82, 2352.16, 1774.04, 1515.34, 1416.73, 1403.21, 1393.03, 1385.23, 1360.28, 1172.67, 1038.14.

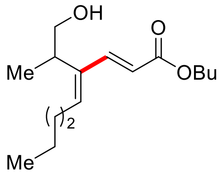


**Butyl (2E,4Z)-4-(1-hydroxypropan-2-yl) nona-2,4-dienoate (3da)**. Yellow oil, yield = 48%. Reaction time: 16 h. $^1$H NMR (500 MHz, $CDCl_3$): δ = 7.24 (d, *J* = 17.0 Hz, 1H), 6.03 (t, *J* = 7.5 Hz, 1H), 5.99 (d, *J* = 16.0 Hz, 1H), 4.15 (t, *J* = 6.7 Hz, 2H), 3.64-3.69 (m, 2H), 2.99-3.05 (m, 1H), 2.22 (q, *J* = 7.0 Hz, 2H), 1.64-1.67 (m, 2H), 1.37-1.43 (m, 4H), 1.33-1.37 (m, 2H), 1.13 (d, *J* = 7.2 Hz, 3H), 0.95 (t, *J* = 7.5 Hz, 3H), 0.91 (t, *J* = 7.0 Hz, 3H). $^{13}$C NMR (125 MHz, $CDCl_3$): δ = 166.33, 145.15, 139.50, 136.45, 116.10, 64.81, 63.22, 35.16, 30.60, 29.77, 27.30, 21.42, 18.17, 14.40, 12.90, 12.73. HR-MS (ESI): m/z calculated for $C_{16}H_{28}O_3$ $[M+H]^+$: 269.2111, found: 269.2112. FTIR (KBr, $cm^{-1}$): 3132.60, 2919.35, 1694.17, 1667.46, 1633.77, 1505.04, 1455.17, 1400.20.

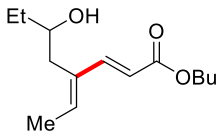


**Butyl (2E,4E)-4-(2-hydroxybutyl) hexa-2,4-dienoate (3ea)**. Alcohol **1e** was prepared according to reported method.[2] Yellow oil, yield = 52%. Reaction time: 16 h. $^1$H NMR (500 MHz, $CDCl_3$): δ = 7.28 (d, *J* = 16.0 Hz, 1H), 6.13-6.17 (m, 1H), 5.82 (d, *J* = 16.0 Hz, 1H), 4.14 (td, *J* = 1.5 Hz, *J* = 7.0 Hz, 2H), 3.63-3.68 (m, 1H), 2.38-2.49 (m, 2H), 1.85 (d, *J* = 7.0 Hz, 3H), 1.61-1.67 (m, 2H), 1.48-1.55 (m, 2H), 1.38-1.42

(m, 2H), 0.97-1.00 (m, 3H), 0.92-0.96 (m, 3H). $^{13}$C NMR (125 MHz, $CDCl_3$): δ = 167.59, 148.62, 139.22, 134.94, 115.70, 71.91, 64.24, 34.12, 30.78, 30.10, 19.19, 15.08, 13.74, 10.15. HR-MS (ESI): m/z calculated for $C_{14}H_{24}O_3$ $[M+H]^+$: 241.1798, found: 241.1795. FTIR (KBr, $cm^{-1}$): 3473.28, 1790.01, 1747.00, 1651.65, 1557.38, 1633.28, 1403.25, 1385.30, 1337.13, 984.16.

**Butyl (2E,4E)-4-(2-hydroxypropyl) hepta-2,4-dienoate (3fa)**. Alcohol **1f** was prepared according to reported method.[2] Reaction time: 16 h. Yellow oil, yield = 59%. $^1$H NMR (500 MHz, $CDCl_3$): δ = 7.22 (d, $J$ = 16.0 Hz, 1H), 6.09 (q, $J$ = 7.0 Hz, 1H), 5.75 (d, $J$ = 15.5 Hz, 1H), 4.07-4.10 (m, 2H), 3.57-3.62 (m, 1H), 2.32-2.43 (m, 2H), 1.79 (d, $J$ = 7.5 Hz, 3H), 1.55-1.61 (m, 2H), 1.42-1.50 (m, 2H), 1.34 (q, $J$ = 7.5 Hz, 2H), 0.92 (t, $J$ = 7.0 Hz, 3H), 0.87 (t, $J$ = 7.5 Hz, 3H). $^{13}$C NMR (125 MHz, $CDCl_3$): δ = 166.58, 147.61, 138.21, 138.94, 114.69, 70.89, 63.23, 33.11, 19.77, 29.09, 18.17, 14.06, 12.73, 9.14. HR-MS (ESI): m/z calculated for $C_{14}H_{24}O_3$ $[M+H]^+$: 241.1798, found: 241.1793. FTIR (KBr, $cm^{-1}$): 3654.31, 3550.32, 3417.97, 1660.18, 1633.83, 1557.43, 1505.24, 1463.09, 1392.95, 1304.11, 1165.32, 780.52 .

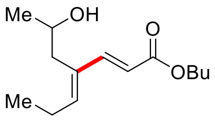


**Butyl (2E,4E)-4-(2-hydroxy-4-methylpentyl) deca-2,4-dienoate (3ga)**. Alcohol **1g** was prepared according to reported method.[3] Yellow oil, yield = 52%. Reaction time: 16 h. $^1$H NMR (500 MHz, $CDCl_3$): δ = 7.28 (d, $J$ = 16.0 Hz, 1H), 6.04 (t, $J$ = 7.5 Hz, 1H), 5.81 (d, $J$ = 16.0 Hz, 1H), 4.13-4.16 (m, 2H), 3.77-3.81 (m, 1H), 2.35-2.46 (m, 2H), 2.23 (q, $J$ = 7.5 Hz, 2H), 1.77-1.83 (m, 1H), 1.61-1.67 (m, 2H), 1.39-1.44 (m, 4H), 1.24-1.31 (m, 6H), 0.94 (t, $J$ = 7.5 Hz, 6H), 0.88 (t, $J$ = 6.5 Hz, 6H). $^{13}$C NMR (125 MHz, $CDCl_3$): δ = 166.55, 147.82, 144.38, 132.66, 114.85, 67.56, 63.22, 45.49, 34.41, 30.55, 29.78, 28.19, 27.88, 23.70, 22.54, 21.51, 20.91, 18.18, 12.97, 12.73. HR-MS (ESI): m/z calculated for $C_{20}H_{36}O_3$ $[M+H]^+$: 347.2557, found: 347.2546. FTIR (KBr, $cm^{-1}$): 3564.70, 3417.21, 2972.05, 2900.15, 1404.08, 1393.46, 1065.67, 1049.40.

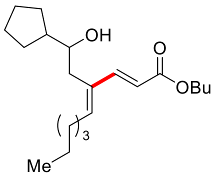


**Butyl (2E,4E)-4-(2-cyclohexyl-2-hydroxyethyl) deca-2,4-dienoate (3ha)**. Alcohol **1h** was prepared according to reported method.[3] Yellow oil, yield = 52%. Reaction time: 16 h. $^1$H NMR (500 MHz, $CDCl_3$): δ = 7.27 (d, $J$ = 15.5 Hz, 1H), 6.05 (t, $J$ = 7.5 Hz, 1H), 5.78 (d, $J$ = 16.0 Hz, 1H), 4.10-4.17 (m, 2H), 3.46 (q, $J$ = 5.5 Hz, 1H), 2.41 (d, $J$ = 7.0 Hz, 2H), 2.23 (m, 2H), 1.89 (d, $J$ = 8.0 Hz, 1H), 1.61-1.79 (m, 6H), 1.39 (q, $J$ = 7.5 Hz, 4H), 1.07-1.30 (m, 10H), 0.94 (t, $J$ = 7.5 Hz, 3H), 0.88 (t, $J$ = 7.0 Hz, 3H). $^{13}$C NMR (125 MHz, $CDCl_3$): δ = 166.53, 147.77, 144.45, 133.10, 114.78, 73.40, 63.20, 42.80, 30.55, 29.80, 28.14, 27.89, 27.08, 25.52, 25.31, 25.15, 21.51, 18.18, 12.98, 12.74. HR-MS (ESI): m/z calculated for $C_{22}H_{38}O_3$ $[M+H]^+$: 373.2713, found: 373.2703. FTIR (KBr, $cm^{-1}$): 3626.38, 3616.64, 3425.22, 2972.61, 2925.99, 1462.67, 1454.96, 1384.57, 1087.64, 800.54.

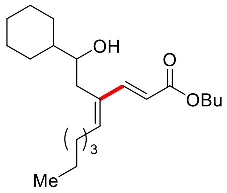


**Butyl (2E,4E)-4-(2-cyclopentyl-2-hydroxyethyl) deca-2,4-dienoate (3ia)**. Alcohol **1i** was prepared according to reported method.[3] Yellow oil, yield = 52%. Reaction time: 16 h. $^1$H NMR (500 MHz, $CDCl_3$): δ = 7.27 (d, $J$ = 16.0 Hz, 1H), 6.05 (t, $J$ = 7.5 Hz, 1H), 5.80 (d, $J$ = 16.0 Hz, 1H), 4.10-4.16 (m, 2H), 3.51 (q, $J$ = 16.0 Hz, 1H), 2.42 (t, $J$ = 4.0 Hz, 2H), 2.21-2.26 (m, 2H), 1.89-1.97 (m, 1H), 1.76-1.83 (m, 2H), 1.62-1.67 (m, 4H), 1.52-1.56 (m, 2H), 1.37-1.43 (m, 4H), 1.24-1.30 (m, 6H), 0.93 (t, $J$ = 7.5 Hz, 3H), 0.88 (t, $J$ = 7.0 Hz, 3H). $^{13}$C NMR (125 MHz, $CDCl_3$): δ = 166.53, 147.79, 144.44, 132.82, 114.84, 73.29, 63.21, 45.43, 32.71, 30.55, 29.79, 28.46, 28.14, 27.89, 27.54, 24.77, 24.62, 21.51, 18.18, 12.98, 12.73. HR-MS (ESI): m/z calculatedfor $C_{21}H_{36}O_3$ $[M+Na]^+$: 359.2557,

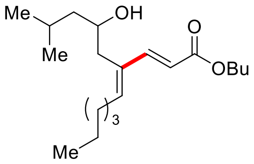

found: 359.2548. FTIR (KBr, $cm^{-1}$): 3646.08, 3626.46, 3584.87, 3417.62, 2971.15, 2899.86, 1651.40, 1633.53, 1621.40, 1065.73.

**Butyl (2E,4E)-4-hexylidene-6-hydroxydodec-2-enoate (3ja)**. Alcohol **1j** was prepared according to

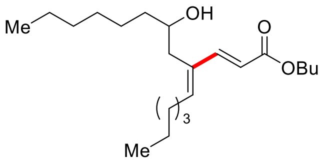


reported method.[3] Yellow oil, yield = 51%. Reaction time: 16 h. $^1$H NMR (500 MHz, $CDCl_3$): δ = 7.22 (d, *J* = 6.0 Hz, 1H), 5.98 (t, *J* = 7.5 Hz, 1H), 5.75 (d, *J* = 16.0 Hz, 1H), 4.07-4.10 (m, 2H), 3.64-3.65 (m, 1H), 2.32-2.38 (m, 2H), 2.17 (q, *J* = 7.5 Hz, 2H), 1.55-1.61 (m, 2H), 1.22-1.43 (m, 18H), 0.88 (t, *J* = 7.5 Hz, 3H), 0.82 (t, *J* = 6.0 Hz, 6H). $^{13}$C NMR (125 MHz, $CDCl_3$): δ = 166.55, 147.78, 144.40, 132.69, 114.85, 69.51, 63.21, 36.32, 33.92, 30.81, 30.55, 29.78, 28.32, 28.17, 27.88, 24.80, 21.60, 21.51, 18.18, 13.06, 12.97, 12.73. HR-MS (ESI): m/z calculated for $C_{22}H_{40}O_3$ $[M+H]^+$: 353.3050, found: 353.3039. FTIR (KBr, $cm^{-1}$): 3626.51, 3421.78, 2971.70, 2925.70, 1651.40, 1621,54, 1384.29, 1086.45.

**Butyl (2E,4E)-4-(2-hydroxy-2-phenylethyl) deca-2,4-dienoate (3ka)**. Alcohol **1k** was prepared

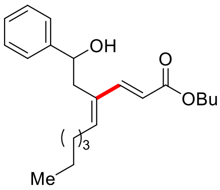


according to reported method.[3] Yellow oil, yield = 56%. Reaction time: 16 h. $^1$H NMR (500 MHz, $CDCl_3$): δ = 7.19-7.28 (m, 6H), 5.92 (t, *J* = 7.5 Hz, 1H), 5.83 (d, *J* = 16.0 Hz, 1H), 4.72 (t, *J* = 7.0 Hz, 1H), 4.08 (t, *J* = 6.5 Hz, 2H), 2.70 (dd, *J* = 7.5 Hz, *J* = 14.0 Hz, 1H), 2.56 (dd, *J* = 6.0 Hz, *J* = 13.5 Hz, 1H), 1.97-2.01 (m, 2H), 1.83-1.88 (m, 1H), 1.56-1.62 (m, 2H), 1.31-1.39 (m, 2H), 1.13-1.21 (m, 6H), 0.89 (t, *J* = 7.5 Hz, 3H), 0.79 (t, *J* = 7.0 Hz, 3H). $^{13}$C NMR (125 MHz, $CDCl_3$): δ = 166.58, 147.63, 144.96, 142.89, 131.83, 127.37, 126.68, 124.77, 114.37, 71.86, 63.22, 35.94, 30.51, 29.79, 27.91, 27.61, 21.45, 18.19, 12.97, 12.74. HR-MS (ESI): m/z calculated for $C_{22}H_{32}O_3$ $[M+H]^+$: 367.2244, found: 367.2231. FTIR (KBr, $cm^{-1}$): 3470.72, 3383.63, 2484.67, 1732.62, 1673.61, 1557.21, 1417.04, 1385.26.

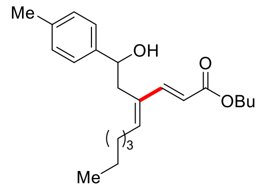


**Butyl (2E,4E)-4-(2-hydroxy-2-(*p*-tolyl) ethyl) deca-2,4- dienoate (3la)**. Alcohol **1l** was prepared according to reported method.[3] Yellow oil, yield = 48%. Reaction time: 16 h. $^1$H NMR (500 MHz, $CDCl_3$): δ = 7.23-7.30 (m, 3H), 7.14 (d, *J* = 7.5 Hz, 2H), 5.99 (t, *J* = 7.5 Hz, 1H), 5.90 (d, *J* = 16.0 Hz, 1H), 4.76 (t, *J* = 7.0 Hz, 1H), 4.16 (t, *J* = 6.5 Hz, 2H), 2.76 (dd, *J* = 8.0 Hz, *J* = 14.0 Hz, 1H), 2.61 (dd, *J* = 6.0 Hz, *J* = 14.0 Hz, 1H), 2.33 (s, 3H), 2.04-2.10 (m, 1H), 1.94-1.96 (m, 2H), 1.63-1.69 (m, 2H), 1.38-1.46 (m, 2H), 1.19-1.28 (m, 6H), 0.96 (t, *J* = 7.5 Hz, 3H), 0.87 (t, *J* = 7.0 Hz, 3H). $^{13}$C NMR (125 MHz, $CDCl_3$): δ = 166.59, 147.61, 144.88, 139.95, 136.34, 131.95, 128.03, 124.71, 114.70, 71.74, 63.20, 35.91, 30.54, 29.80, 27.94, 27.64, 21.47, 20.09, 18.19, 12.98, 12.74. HR-MS (ESI): m/z calculated for $C_{23}H_{34}O_3$ $[M+H]^+$: 359.2581, found: 359.2583. FTIR (KBr, $cm^{-1}$): 3742.37, 3444.58, 2988.72, 1621.41, 1417.21, 1304.80, 1175.05, 984.38.

**Butyl (2E,4E)-4-(2-hydroxy-2-(4-methoxyphenyl) ethyl) deca- 2,4-dienoate (3ma)**. Alcohol **1m** was

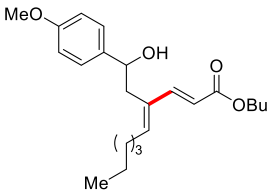


prepared according to reported method.[3] Yellow oil, yield = 55%. Reaction time: 16 h. $^1$H NMR (500 MHz, $CDCl_3$): δ = 7.26-7.29 (m, 3H), 6.86 (d, *J* = 8.7 Hz, 2H), 5.98 (t, *J* = 7.4 Hz, 1H), 5.89 (d, *J* = 16.0 Hz, 1H), 4.74 (t, *J* = 6.9 Hz, 1H), 4.15 (t, *J* = 6.7 Hz, 2H), 3.79 (s, 3H), 2.76 (dd, *J* = 13.7 Hz, *J* = 7.6 Hz, 1H), 2.60 (dd, *J* = 13.7 Hz, *J* = 6.2 Hz, 1H), 2.05-2.07 (m, 2H), 1.92-1.96 (m, 1H), 1.63-1.68 (m, 2H), 1.38-1.44 (m, 2H), 1.20-1.30 (m, 6H), 0.96 (t, *J* = 7.4 Hz, 3H), 0.87 (t, *J* = 7.1 Hz, 3H). $^{13}$C NMR (125 MHz, $CDCl_3$): δ = 166.61, 158.15, 147.70, 144.84, 135.09,

131.95, 126.00, 114.68, 112.72, 71.48, 63.21, 54.25, 35.91, 30.53, 29.79, 27.94, 27.64, 21.46, 18.19, 12.97, 12.74. HR-MS (ESI): m/z calculated for $C_{23}H_{34}O_4$ $[M+H]^+$: 375.2530, found: 375.2521. FTIR (KBr, $cm^{-1}$): 3362.10, 1698.59, 1614.59, 1434.71, 1247.38, 1178.00, 1047.83, 880.44, 832.29, 666.62.

**Butyl(2E,4E)-4-(2-(4-chlorophenyl)-2-hydroxyethyl)deca-2,4-dienoate (3na)**. Alcohol **1n** was

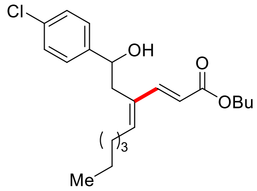


prepared according to reported method.[3] Yellow oil, yield = 48%. Reaction time: 16 h. $^1$H NMR (500 MHz, $CDCl_3$): δ = 7.34-7.26 (m, 5H), 6.00 (t, *J* = 7.5 Hz, 1H), 5.87 (d, *J* = 15.9 Hz, 1H), 4.78 (t, *J* = 6.9 Hz, 1H), 4.16 (t, *J* = 6.7 Hz, 2H), 2.74 (dd, *J* = 13.8 Hz, *J* = 7.6 Hz, 1H), 2.60 (dd, J = 13.8 Hz, *J* = 6.2 Hz, 1H), 2.06 (d, *J* = 26.1 Hz, 2H), 1.94-1.88 (m, 1H), 1.68-1.64 (m, 2H), 1.42 (dd, *J* = 15.0 Hz, 7.5 Hz, 2H), 1.32-1.22 (m, 6H), 0.96 (t, *J* = 7.4 Hz, 3H), 0.87 (t, *J* = 7.1 Hz, 3H). $^{13}$C NMR (125 MHz, $CDCl_3$): δ = 166.50. 147.46, 145.12, 141.32, 132.33, 131.46, 127.47, 126.19, 114.80, 71.74, 63.29, 35.94, 30.53, 29.78, 27.96, 27.63, 21.46, 18.19, 12.97, 12.74. HR-MS (ESI): m/z calculated for $C_{22}H_{31}ClO_3$ $[M+H]^+$: 379.2034, found: 379.2031. FTIR (KBr, $cm^{-1}$): 3417.55, 2970.43, 2924.78, 1633.28, 1311.51, 1183.92, 1090.59, 1049.75, 880.77.

**Butyl (2E,4E)-4-(2-(4-fluorophenyl)-2-hydroxyethyl) deca-2,4- dienoate (3oa)**. Alcohol **1o** was

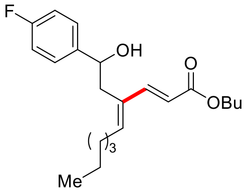


prepared according to reported method.[3] Yellow oil, yield = 53%. Reaction time: 16 h. $^1$H NMR (500 MHz, $CDCl_3$): δ = 7.26-7.33 (m, 3H), 7.01 (t, *J* = 8.7 Hz, 2H), 5.99 (t, *J* = 7.5 Hz, 1H), 5.87 (d, *J* = 16.0 Hz, 1H), 4.78 (t, *J* = 6.9 Hz, 1H), 4.15 (t, *J* = 6.7 Hz, 2H), 2.75 (dd, *J* = 13.8 Hz, *J* = 7.6 Hz, 1H), 2.60 (dd, *J* = 13.8 Hz, *J* = 6.3 Hz, 1H), 2.15 (s, 1H), 2.05 (dd, *J* = 14.3 Hz , *J* = 7.2 Hz, 1H), 1.91 (dd, *J* = 25.0 Hz, *J* = 11.6 Hz, 1H), 1.63-1.69 (m, 2H), 1.40-1.46 (m, 2H), 1.20-1.29 (m, 6H), 0.96 (t, *J* = 7.4 Hz, 3H), 0.87 (t, *J* = 7.1 Hz, 3H). $^{13}$C NMR (125 MHz, $CDCl_3$): δ = 166.53, 161.25 (d, $J_{C-F}$ = 244.1 Hz), 147.52, 145.03, 138.6 (d, $J_{C-F}$ = 3.1 Hz), 131.60, 126.4 (d, $J_{C-F}$ = 8.0 Hz), 114.76, 114.1 (d, $J_{C-F}$ = 21.3 Hz), 71.19, 63.27, 36.02, 30.52, 29.78, 27.92, 27.61, 21.45, 18.18, 12.95, 12.73. HR-MS (ESI): m/z calculated for $C_{22}H_{31}FO_3$ $[M+H]^+$: 363.2330, found: 363.2335. FTIR (KBr, $cm^{-1}$): 3667.37, 3383.12, 2970.39, 2925.21, 1452.86, 1087.60, 1049.25, 880.44.

**Butyl (2E,4E)-4-(2-([1,1'-biphenyl]-4-yl)-2-hydroxyethyl) deca-2,4-dienoate (3pa)**. Alcohol **1p** was

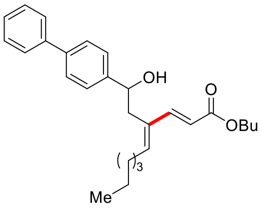


prepared according to reported method.[3] Yellow solid, yield = 60%. Melting point: 47 °C. Reaction time: 16 h. $^1$H NMR (500 MHz, $CDCl_3$): δ = 7.55-7.58 (m, 4H), 7.41-7.44 (m, 4H), 7.25-7.35 (m, 2H), 6.01 (t, *J* = 7.4 Hz, 1H), 5.92 (d, *J* = 15.9 Hz, 1H), 4.84 (t, *J* = 6.8 Hz, 1H), 4.15 (t, *J* = 6.7 Hz, 2H), 2.80 (dd, *J* = 13.8 Hz, *J* = 7.7 Hz, 1H), 2.66 (dd, *J* = 13.8 Hz, *J* = 6.1 Hz, 1H), 2.18 (s, 1H), 2.05-2.12 (m, 1H), 1.91-1.98 (m, 1H), 1.62-1.68 (m, 2H), 1.38-1.41 (m, 2H), 1.21-1.29 (m, 6H), 0.94 (t, *J* = 7.4 Hz, 3H), 0.84 (t, *J* = 6.9 Hz, 3H). $^{13}$C NMR (125 MHz, $CDCl_3$): δ = 166.59, 147.64, 145.04, 141.91, 139.77, 139.62, 131.78, 127.73, 126.27, 126.07, 126.02, 125.24, 114.73, 71.61, 63.24, 35.93, 30.52, 29.77, 27.95, 27.64, 21.44, 18.18, 12.95, 12.73. HR-MS (ESI): m/z calculated for $C_{28}H_{36}O_3$ $[M+H]^+$: 421.2737, found: 421.2732. FTIR (KBr, $cm^{-1}$): 3606.29, 3418.11, 1633.44, 1434.93, 1359.46, 1049.89, 880.53, 773.38.

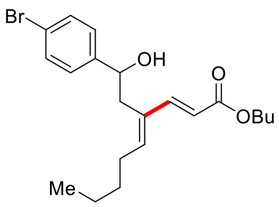


**Butyl (2E,4E)-4-(2-(4-bromophenyl)-2-hydroxyethyl) nona -2,4-dienoate (3qa)**

Alcohol **1q** was prepared according to reported method.[3] Following the general procedure, **3qa** was obtained as a yellow oil (37.6 mg, 31% yield). $^1$H NMR (500 MHz, $CDCl_3$) δ 7.46 (d, *J* = 8.5 Hz, 2H), 7.27 (d, *J* = 15.5 Hz, 1H), 7.23 (d, *J* = 8.5 Hz, 2H), 6.00 (t, *J* = 7.5 Hz, 1H), 5.87 (d, *J* = 16.0 Hz, 1H), 4.77 (t, *J* = 7.0Hz, 1H), 4.16 (t, *J* = 6.5 Hz, 2H), 2.74 (dd, *J* = 13.5, 7.5 Hz, 1H), 2.60 (dd, *J* = 13.5, 6.0 Hz, 1H), 2.07 – 2.02 (m, 2H), 1.69 – 1.64 (m, 2H), 1.46 – 1.40 (m, 2H), 1.30 – 1.23 (m, 4H), 0.96 (t, *J* = 7.5 Hz, 3H), 0.87 (t, *J* = 7.0 Hz, 3H); $^{13}$C NMR (125 MHz, $CDCl_3$) δ 166.46, 147.41, 145.09, 141.80, 131.41, 130.42, 126.51, 120.42, 114.81, 71.18, 63.30, 35.89, 30.06, 29.78, 27.72, 21.43, 18.18, 12.87, 12.74; HRMS (ESI): m/z for $C_{21}H_{30}BrO_3$ $[M+H]^+$: 409.1373, found: 409.1365; FTIR (KBr, $cm^{-1}$): 3417.26, 2926.17, 2856.07, 2354.21, 1651.63, 1644.90, 1633.89, 1615.05, 1557.38, 1538.50, 1505.23, 806.54,436.05.

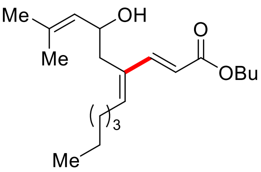


**Butyl (2E,4E)-4-(2-hydroxy-4-methylpent-3-en-1-yl) deca-2,4- dienoate (3ra)**. Alcohol **1r** was prepared according to reported method.[3] Yellow oil, yield = 47%. Reaction time: 16 h. $^1$H NMR (500 MHz, $CDCl_3$): δ = 7.26-7.30 (d, *J* = 12.5 Hz, 1H), 6.01 (t, *J* = 7.5 Hz, 1H), 5.89 (d, *J* = 15.8 Hz, 1H), 5.19 (d, *J* = 9.8 Hz, 1H), 4.49 (dd, *J* = 15.2 Hz, *J* = 7.0 Hz, 1H), 4.15 (t, *J* = 6.7 Hz, 2H), 2.56 (dd, *J* = 13.7, 7.3 Hz, 1H), 2.43 (dd, *J* = 13.7, 6.2 Hz, 1H), 2.23 (q, *J* = 7.4 Hz, 2H), 1.70 (s, 3H), 1.66 (dd, *J* = 14.4, 7.4 Hz, 2H), 1.62 (s, 3H), 1.41 (dd, *J* = 14.9, 7.5 Hz, 4H), 1.30 (d, *J* = 7.2 Hz, 4H), 0.95 (t, *J* = 7.4 Hz, 3H), 0.89 (t, *J* = 6.9 Hz, 3H). $^{13}$C NMR (125 MHz, $CDCl_3$): δ = 166.64, 147.91, 144.36, 134.51, 132.13, 126.32, 114.67, 66.51, 63.16, 33.78, 30.59, 29.77, 28.23, 27.84, 24.74, 21.52, 18.18, 17.36, 12.99, 12.73. HR-MS (ESI): m/z calculated for $C_{20}H_{34}O_3$ $[M+H]^+$: 323.2581, found: 323.2587. FTIR (KBr, $cm^{-1}$): 3851.13, 3286.23, 2970.43, 2922.78, 1629.78, 1049.76, 880.79, 781.42.

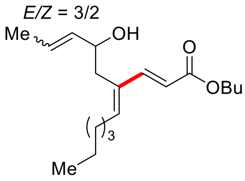


**Butyl (2E,4E)-4-((E)-2-hydroxypent-3-en-1-yl) deca-2,4-dienoate (3sa)**. Alcohol **1s** was prepared as a *E*/*Z* mixture according to reported method (2*E*/2*Z* = 3/2).[3] Yellow oil, yield = 42%. Reaction time: 16 h. According to NMR spectrum, *E*/*Z* = 3/2. $^1$H NMR (500 MHz, $CDCl_3$): δ = 7.26-7.31 (m, 1H), 6.03 (t, *J* = 7.5 Hz, 1H), 5.90 (d, *J* = 16 Hz, 0.6H), 5.85 (d, *J* = 15.5 Hz, 0.4H), 5.62-5.69 (m, 0.4H), 5.54-5.59 (m, 0.6H), 5.48-5.52 (m, 0.4H), 5.41-5.45 (m, 0.6H), 4.60 (q, *J* = 7.5 Hz, 0.7H), 4.15 (t, *J* = 6.5 Hz, 2H), 2.53-2.60 (m, 1H), 2.42-2.46 (m, 1H), 2.21-2.27 (m, 2H), 1.62-1.69 (m, 6H), 1.39-1.45 (m, 4H), 1.30 (t, *J* = 3.5 Hz, 3H), 0.95 (t, *J* = 7.5 Hz, 3H), 0.89 (t, *J* = 6.5 Hz, 3H). $^{13}$C NMR (125 MHz, $CDCl_3$): δ = 166.61, 147.85, 147.79, 144.48, 144.44, 132.27, 132.10, 131.94, 131.60, 126.09, 125.77, 114.79, 114.71, 70.57, 65.24, 63.19, 33.75, 33.52, 30.56, 29.77, 28.26, 28.20, 27.85, 27.82, 21.52, 21.50, 18.18, 16.62, 12.98, 12.73, 12.44. HR-MS (ESI): m/z calculated for $C_{19}H_{32}O_3$ $[M+H]^+$: 309.2424, found: 309.2423. FTIR (KBr, $cm^{-1}$): 3584.88, 3264.14, 2969.64, 2924.43, 1683.58, 1504.73, 1307.18, 1088.28, 1049.17, 880.56, 666.73.

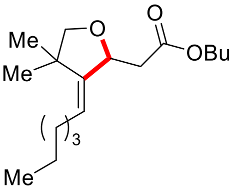


**Butyl (S,E)-2-(3-hexylidene-4,4-dimethyltetrahydrofuran-2-yl) acetate (3A)**. Tertiary alcohol was prepared according to reported method.[4] Yellow oil, yield = 33%. Reaction time: 16 h. $^1$H NMR (500 MHz, $CDCl_3$): δ = 5.06 (td, *J* = 1.5 Hz, *J* = 7.5 Hz, 1H), 4.72-4.75 (m, *J* = 6.5 Hz, 1H), 4.08-4.11 (m, 2H), 3.56 (dd, *J* = 3.5 Hz, *J* = 73 Hz, 2H), 2.55 (d, *J* = 6.5 Hz, 2H), 2.09-2.18 (m,

2H), 1.60-1.63 (m, 2H), 1.29-1.41 (m, 8H), 1.21 (s, 3H), 1.21 (s, 3H), 0.92 (t, *J* = 7.5 Hz, 3H), 0.89 (t, *J* = 7.0 Hz, 3H). $^{13}$C NMR (125 MHz, $CDCl_3$): δ = 170.44, 147.04, 120.35, 80.02, 78.49, 63.46, 40.94, 40.27, 30.53, 29.63, 28.76, 26.58, 25.19, 24.01, 21.57, 18.11, 13.02, 12.70. HR-MS (ESI): m/z calculated for $C_{18}H_{32}O_3$ $[M+H]^+$: 297.2424, found: 297.2425. FTIR (KBr, $cm^{-1}$): 3581.22, 3748.63, 3742.6, 3626.93, 1651.74, 1538.67, 1505.42, 1455.49.

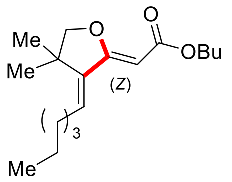


**Butyl (Z)-2-((E)-3-hexylidene-4,4-dimethyldihydrofuran-2(3H)- ylidene) acetate (3A′)**. Yellow oil, yield = 17%. Reaction time: 16 h. $^1$H NMR (500 MHz, $CDCl_3$): δ = 6.12 (t, *J* = 7.5 Hz, 1H), 5.21 (s, 1H), 4.08-4.11 (m, 4H), 2.29 (q, *J* = 7.5 Hz, 2H), 1.59-1.63 (m, 4H), 1.37-1.45 (m, 4H), 1.32 (t, *J* = 3.0 Hz, 2H), 1.29 (s, 6H), 0.89-0.94 (m, 6H). $^{13}$C NMR (125 MHz, $CDCl_3$): δ = 168.15, 165.49, 139.69, 129.12, 83.24, 82.95, 62.15, 39.02, 30.53, 29.96, 28.18, 26.66, 24.27, 21.50, 18.21, 12.96, 12.77. HR-MS (ESI): m/z calculated for $C_{18}H_{30}O_3$ $[M+H]^+$: 295.2268, found: 295.2259. FTIR (KBr, $cm^{-1}$): 3473.03, 3318.40, 1673.79, 1638.40, 1557.43, 1505.19, 1445.93, 1372.47.

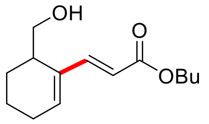


**Butyl (E)-3-(6-(hydroxymethyl) cyclohex-1-en-1-yl) acrylate (3ta)**. Alcohol **1t** was prepared according to reported method.[5] Yellow oil, yield = 33%. Reaction time: 16 h. $^1$H NMR (500 MHz, $CDCl_3$): δ = 7.23 (d, *J* = 16.0 Hz, 1H), 6.24 (t, *J* = 4.0 Hz, 1H), 5.89 (d, *J* = 16.0 Hz, 1H), 4.14 (t, *J* = 6.5 Hz, 2H), 3.72 (dd, *J* = 3.0 Hz, *J* = 10.5 Hz, 1H), 3.55 (t, *J* = 9.5 Hz, 1H), 2.63 (s, 1H), 2.21-2.23 (m, 2H), 2.02-2.05 (m, 1H), 1.63-1.67 (m, 5H), 1.37-1.44 (m, 2H), 0.94 (t, *J* = 7.5 Hz, 3H). $^{13}$C NMR (125 MHz, $CDCl_3$): δ = 167.72, 147.16, 140.99, 134.67, 115.00, 64.24, 63.65, 36.22, 30.79, 26.40, 23.59, 19.20, 17.24, 13.75. HR-MS (ESI): m/z calculated for $C_{12}H_{20}O_3$ $[M+H]^+$: 235.1305, found: 235.1321. FTIR (KBr, $cm^{-1}$): 3550.50, 3472.87, 1732.26, 1714.52, 1651.71, 1626.64, 1463.20, 1416.88, 1393.11, 1372.51, 1172.46.

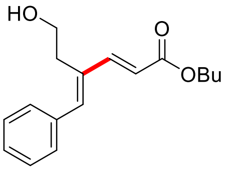


**Butyl (2E,4E)-4-(2-hydroxyethyl)-5-phenylpenta-2,4-dienoate (3ua)**. Alcohol **1u** was prepared according to reported method.[6] Yellow oil, yield = 52%. Reaction time: 16 h. $^1$H NMR (500 MHz, $CDCl_3$): δ = 7.29-7.38 (m, 5H), 7.22-7.23 (m, 1H), 6.89 (s, 1H), 5.97 (d, *J* = 15.5 Hz, 1H), 4.12 (t, *J* = 6.5 Hz, 2H), 3.77 (t, *J* = 7.0 Hz, 2H), 2.76 (t, *J* = 7.5 Hz, 2H), 1.57-1.63 (m, 2H), 1.33-1.39 (m, 2H), 0.88 (t, *J* = 7.5 Hz, 3H). $^{13}$C NMR (125 MHz, $CDCl_3$): δ = 167.35, 148.70, 141.09, 136.12, 134.49, 129.13, 128.57, 128.16, 117.62, 64.42, 61.22, 30.79, 30.51, 19.21, 13.76. HR-MS (ESI): m/z calculated for $C_{17}H_{22}O_3$ $[M+H]^+$: 275.1642, found: 275.1642. FTIR (KBr, $cm^{-1}$): 3444.75, 3418.02, 3382.09, 1747.06, 1634.03, 1403.31, 1385.36, 1337.39, 996.77.

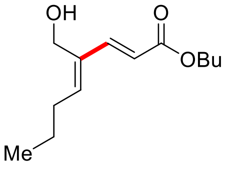


**Butyl (2E,4Z)-4-(hydroxymethyl) octa-2,4-dienoate (3va)**. Yellow oil, yield = 44%. Reaction time: 16h. $^1$H NMR (500 MHz, $CDCl_3$): δ = 7.19 (d, *J* = 13.0 Hz, 1H), 5.96-6.02 (m, 2H), 4.30 (s, 2H), 4.09 (m, 2H), 2.20 (m, 2H), 1.55-1.61 (m, 2H), 1.32-1.43 (m, 4H), 0.85-0.89 (m, 6H). $^{13}$C NMR (125 MHz, $CDCl_3$): δ = 166.55, 145.62, 143.71, 134.96, 115.85, 63.22, 55.95, 29.76, 29.55, 21.48, 18.16, 12.76, 12.71. HR-MS (ESI): m/z calculated for $C_{13}H_{22}O_3$ $[M+H]^+$: 227.1642, found: 227.1640. FTIR (KBr, $cm^{-1}$): 3419.93, 3354.12, 2200.93, 1732.19, 1714.52, 1428.03, 1385.28, 1257.61, 999.38.

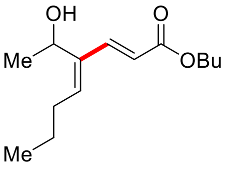


**Butyl (2E,4Z)-4-(1-hydroxyethyl) octa-2,4-dienoate (3wa)**. Yellow oil, yield = 20%. Reaction time: 16 h. $^1$H NMR (500 MHz, $CDCl_3$): δ = 7.22 (d, *J* = 16.0 Hz, 1H), 6.24 (d, *J* = 16.0 Hz, 1H), 5.89 (t, *J* = 7.5 Hz, 1H), 4.95 (q, *J* = 6.5 Hz, 1H), 4.15 (t, *J* = 7.0 Hz, 2H), 2.20-2.25 (m, 2H), 1.62-1.68 (m, 2H), 1.57-1.58

(m, 2H), 1.44-1.48 (m, 2H), 1.39-1.40 (d, *J* = 7.0 Hz, 3H), 0.92-0.96 (m, 6H). $^{13}$C NMR (125 MHz, $CDCl_3$): δ = 166.60, 143.85, 139.26, 138.66, 116.76, 64.45, 63.17, 29.78, 29.31, 21.57, 20.97, 18.17, 12.83, 12.73. HR-MS (ESI): m/z calculated for $C_{14}H_{24}O_3$ $[M+H]^+$: 241.1798, found: 241.1794. FTIR (KBr, $cm^{-1}$): 3616.60, 3550.07, 2924.32, 1682.93, 1462.87, 1385.04, 1173.40, 1089.05, 1049.32, 880.76, 666.71.

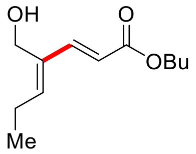


**Butyl (2E,4Z)-4-(hydroxymethyl) hepta-2,4-dienoate (3xa)**. Yellow oil, yield = 37%. Reaction time: 16 h. $^1$H NMR (500 MHz, $CDCl_3$): δ = 7.25 (d, *J* = 15.0 Hz, 1H), 6.07 (d, *J* = 15.5 Hz, 1H), 6.03-6.09 (m, 1H), 4.37 (s, 2H), 4.16 (t, *J* = 6.5 Hz, 2H), 2.28-2.34 (m, 2H), 1.62-1.68 (m, 2H), 1.37-1.45 (m, 2H), 1.07 (t, *J* = 7.5 Hz, 3H), 0.95 (t, *J* = 7.5 Hz, 3H). $^{13}$C NMR (125 MHz, $CDCl_3$): δ = 166.51, 145.61, 145.27, 134.27, 115.91, 63.22, 55.90, 29.76, 20.92, 18.16, 12.85, 12.72. HR-MS (ESI): m/z calculated for $C_{12}H_{20}O_3$ $[M+H]^+$: 213.1485, found: 213.1478. FTIR (KBr, $cm^{-1}$): 3441.21, 3417.76, 3332.43, 2966.36, 2925.54, 2358.45, 1694.28, 1682.65, 1170.32, 1047.71.

**Butyl (2E,4Z)-4-(hydroxymethyl) undeca-2,4-dienoate (3ya)**. Yellow oil, yield = 43%. Reaction

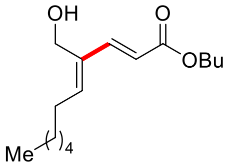


time: 16 h. $^1$H NMR (500 MHz, $CDCl_3$): δ = 7.25 (d, *J* = 16.0 Hz, 1H), 6.03-6.08 (m, 2H), 6.03 (d, *J* = 7.5 Hz, 1H), 4.37 (s, 2H), 4.16 (t, *J* = 6.5 Hz, 2H), 2.28 (m, 2H), 1.62-1.68 (m, 2H), 1.40-1.45 (m, 4H), 1.26-1.39 (m, 6H), 0.95 (t, *J* = 7.5 Hz, 3H), 0.89 (t, *J* = 6.5 Hz, 3H). $^{13}$C NMR (125 MHz, $CDCl_3$): δ = 166.53, 145.63, 144.03, 134.75, 115.80, 63.21, 55.98, 30.60, 29.76, 28.23, 27.95, 27.61, 21.54, 18.17, 13.04, 12.72. HR-MS (ESI): m/z calculated for $C_{16}H_{28}O_3$ $[M+H]^+$: 269.2111, found: 269.2105. FTIR (KBr, $cm^{-1}$): 3667.55, 3592.70, 3673.42, 2969.15, 2924.77, 2354.70, 1698.72, 1311.46, 1173.28, 1088.65, 1049.63, 880.78.

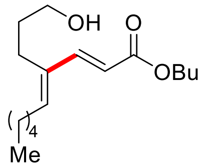


**Butyl (2E,4E)-4-(3-hydroxypropyl) deca-2,4-dienoate (3za)**. Yellow oil, yield = 22%. Reaction time: 16 h. $^1$H NMR (500 MHz, $CDCl_3$): δ = 7.24 (d, *J* = 16.5 Hz, 1H), 5.91 (t, *J* = 7.5 Hz, 1H), 5.83 (t, *J* = 15.5 Hz, 1H), 4.15 (t, *J* = 7.0 Hz, 2H), 3.66 (t, *J* = 6.5 Hz, 2H), 2.34 (t, *J* = 7.5 Hz, 2H), 2.20 (m, 2H), 1.63-1.68 (m, 4H), 1.39-1.45 (m, 4H), 1.30-1.31 (m, 4H), 0.95 (t, *J* = 7.5 Hz, 3H), 0.89 (t, *J* = 6.5 Hz, 3H). $^{13}$C NMR (125 MHz, $CDCl_3$): δ = 166.74, 147.60, 142.14, 135.59, 114.23, 63.15, 61.49, 30.55, 30.54, 29.80, 27.85, 27.68, 21.75, 21.51, 18.18, 12.99, 12.73. HR-MS (ESI): m/z calculated for $C_{17}H_{30}O_3$ $[M+H]^+$: 283.2268, found: 283.2264. FTIR (KBr, $cm^{-1}$): 3383.11, 2973.09, 2925.40, 2899.49, 1454.95, 1381.99, 1087.75, 880.42.

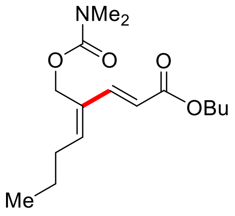


**Butyl (2E,4Z)-4-(((dimethylcarbamoyl)oxy)methyl) octa-2,4-dienoate (5aa)**. Light yellow oil, yield = 56%. Reaction time: 16 h. $^1$H NMR ($CDCl_3$): δ = 7.20 (d, *J* = 16.0 Hz, 1H), 6.05 (t, *J* = 7.5 Hz, 1H), 5.91 (d, *J* = 16.0 Hz, 1H), 4.73 (s, 2H), 4.09 (t, *J* = 7.0 Hz, 2H), 2.85 (brs, 3H), 2.78 (brs, 3H), 2.22-2.27 (m, 2H), 1.56-1.61 (m, 2H), 1.30-1.44 (m, 4H), 0.85-0.89 (m, 6H). $^{13}$C NMR ($CDCl_3$): δ = 166.52, 155.31, 145.62, 145.50, 131.45, 115.85, 63.23, 58.14, 35.46, 34.91, 29.76, 29.69, 21.38, 18.18, 12.73, 12.70. HRMS (ESI): m/z calculated for $C_{16}H_{27}NO_4$ $[M+H]^+$: 320.1832, found: 320.1825. FTIR (KBr, $cm^{-1}$): 3592.49, 3507.26, 3444.61, 3383.21, 3362.33, 3299.65, 1746.99, 1574.36, 1360.12, 1311.47, 1180.45.

**Methyl (2E,4Z)-4-(((dimethylcarbamoyl) oxy) methyl) octa-2,4- dienoate (5ab)**. Light yellow oil, yield = 45%. Reaction time: 16 h. $^1$H NMR ($CDCl_3$): δ = 7.22 (d, *J* = 16.0 Hz, 1H), 6.06 (t, *J* = 7.6 Hz, 1H), 5.92(d, *J* = 16.0 Hz, 1H), 4.73 (s, 2H), 3.68 (s, 3H), 2.85 (brs, 3H), 2.78 (brs, 3H), 2.22-2.28 (m, 2H), 1.37-1.43 (m, 2H), 0.86 (t, *J* = 7.6 Hz, 3H). $^{13}$C NMR ($CDCl_3$): δ = 167.85, 156.29, 146.96, 146.69, 132.47, 116.39, 59.11, 51.50, 36.45, 35.91, 30.70, 22.37, 13.69. HRMS (ESI): m/z calculated for $C_{13}H_{21}NO_4$ $[M+H]^+$: 256.1543, found: 256.1538. FTIR (KBr, $cm^{-1}$): 3507.38, 3383.34, 3355.55, 1713.65, 1667.47, 1644.54, 1557.19, 1538.18, 1392.89, 1180.24.

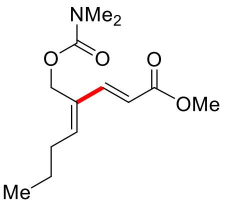


**Ethyl (2E,4Z)-4-(((dimethylcarbamoyl)oxy) methyl) octa-2,4-dienoate (5ac)**. Light yellow oil, yield = 46%. Reaction time: 16 h. $^1$H NMR ($CDCl_3$): δ = 7.21 (d, *J* = 16.0 Hz, 1H), 6.06 (t, *J* = 7.6 Hz, 1H), 5.91 (d, *J* = 16.0 Hz, 1H), 4.73 (s, 2H), 4.14 (q, *J* = 7.2 Hz, 2H), 2.85 (brs, 3H), 2.78 (brs, 3H), 2.22-2.27 (m, 2H), 1.37-1.43 (m, 2H), 1.23 (t, *J* = 7.2 Hz, 3H), 0.86 (t, *J* = 7.2 Hz, 3H). $^{13}$C NMR ($CDCl_3$): δ = 167.42, 156.32, 146.68, 146.52, 132.47, 116.84, 60.28, 59.14, 36.48, 35.92, 30.70, 22.38, 14.23, 13.70. HRMS (ESI): m/z calculated for $C_{14}H_{23}NO_4$ $[M+H]^+$: 270.17, found: 270.1694. FTIR (KBr, $cm^{-1}$): 3592.25, 3382.39, 2972.77, 2899.30, 1694.03, 1557.19, 1455.07, 1048.79, 880.70, 666.38.

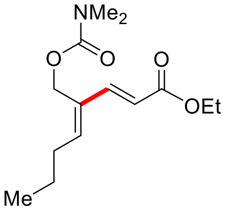


**Hexyl (2E,4Z)-4-(((dimethyl carbamoyl) oxy) methyl) octa-2,4- dienoate (5ad)**. Light yellow oil, yield = 49%. Reaction time: 16 h. $^1$H NMR ($CDCl_3$): δ = 7.20 (d, *J* = 16.0 Hz, 1H), 6.06 (t, *J* = 7.6 Hz, 1H), 5.91 (d, *J* = 16.0 Hz, 1H), 4.73 (s, 2H), 4.07 (t, *J* = 6.8 Hz, 2H), 2.85 (brs, 3H), 2.78 (brs, 3H), 2.22-2.27 (m, 2H), 1.58-1.63 (m, 2H), 1.37-1.43 (m, 2H), 1.24-1.28 (m, 6H), 0.82-0.88 (m, 6H).$^{13}$C NMR ($CDCl_3$): δ = 167.51, 156.32, 146.61, 146.44, 132.49, 116.89, 64.53, 59.16, 36.46, 35.94, 31.45, 30.70, 28.68, 25.62, 22.53, 22.38, 13.98, 13.70. HRMS (ESI): m/z calculated for $C_{18}H_{31}NO_4$ $[M+H]^+$: 326.2326, found: 326.2322. FTIR (KBr, $cm^{-1}$): 3564.40, 3417.65, 2971.38, 2926.10, 2900.03, 1731.86, 1434.87, 1304.62, 1049.47, 880.31.

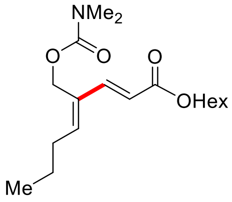


**Isobutyl (2E,4Z)-4-(((dimethylcarbamoyl)oxy)methyl) octa-2,4 -dienoate (5ae)**. Light yellow oil, yield = 55%. Reaction time: 16 h. $^1$H NMR ($CDCl_3$): δ = 7.29 (d, *J* = 15.6 Hz, 1H), 6.14 (t, *J* = 7.6 Hz, 1H), 6.00 (d, *J* = 16.0 Hz, 1H), 4.82 (s, 2H), 3.95 (d, *J* = 6.4 Hz, 2H), 2.94 (brs, 3H), 2.87 (brs, 3H), 2.30-2.36 (m, 2H), 1.97-2.01 (m, 1H), 1.46-1.51 (m, 2H), 0.95-0.98 (m, 9H). $^{13}$C NMR ($CDCl_3$): δ = 167.46, 156.31, 146.59, 146.41, 132.47, 116.87, 70.47, 59.17, 36.46, 35.92, 30.70, 27.81, 22.38, 19.13, 13.70. HRMS (ESI): m/z calculated for $C_{16}H_{27}NO_4$ $[M+H]^+$: 320.1832, found: 320.1829. FTIR (KBr, $cm^{-1}$): 3626.78, 3606.18, 3592.64, 3417.83, 2971.32, 2924.18, 2900.15, 1651.56, 1470.62, 1049.53, 880.45.

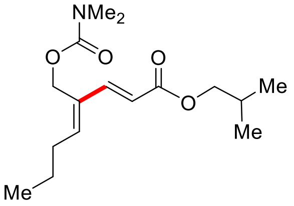


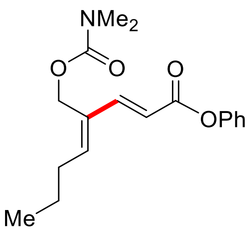


**Phenyl (2E,4Z)-4-(((dimethylcarbamoyl)oxy)methyl)octa-2,4-dienoate (5af)**. Light yellow oil, yield = 46%. Reaction time: 16 h. $^1$H NMR ($CDCl_3$): δ = 7.30-7.41 (m, 3H), 7.14-7.18 (m, 1H), 7.05-7.07 (m, 2H), 6.09-6.17 (m, 2H), 4.79 (s, 2H), 2.87 (brs, 3H), 2.82 (brs, 3H), 2.26-2.31 (m, 2H), 1.38-1.47 (m, 2H), 0.88 (t, *J* = 7.2 Hz, 3H). $^{13}$C NMR ($CDCl_3$): δ = 165.76, 156.29, 150.88, 148.67, 147.85, 132.59, 129.37, 125.64, 121.63, 115.94, 59.10, 36.53, 35.98, 30.83, 22.35, 13.72. HRMS (ESI): m/z calculated for $C_{18}H_{23}NO_4$ $[M+H]^+$:

318.1700, found: 318.1697. FTIR (KBr, $cm^{-1}$): 3850.93, 3584.92, 3564.47, 3444.74, 1704.39, 1673.50, 1574.39, 1367.49, 1182.05, 1152.61.

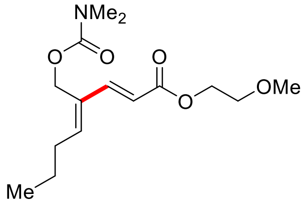


**2-Methoxyethyl (2E,4Z)-4-(((dimethylcarbamoyl)oxy)methyl) octa-2,4-dienoate (5ag)**. Light yellow oil, yield = 50%. Reaction time: 16 h.$^{1}$H NMR ($CDCl_3$): δ = 7.32 (d, *J* = 15.6 Hz, 1H), 6.15 (t, *J* = 7.6 Hz, 1H), 6.04 (d, *J* = 16.0 Hz, 1H), 4.80 (s, 2H), 4.33 (t, *J* = 4.8 Hz, 2H), 3.65 (t, *J* = 4.8 Hz, 2H), 3.42 (s, 3H), 2.93 (brs, 3H), 2.86 (brs, 3H), 2.30-2.35 (m, 2H), 1.45-1.51 (m, 2H), 0.94 (t, *J* = 7.6 Hz, 3H). $^{13}C$ NMR ($CDCl_3$): δ = 167.34, 156.28, 147.24, 146.86, 132.46, 116.37, 70.59, 63.39, 59.09, 58.99, 36.47, 35.95, 30.71, 22.36, 13.69. HRMS (ESI): m/z calculated for $C_{15}H_{25}NO_5$ $[M+H]^+$: 300.1805, found: 300.1803. FTIR (KBr, $cm^{-1}$): 3646.14, 3472.72, 3383.40, 3299.68, 1732.14, 1633.34, 1515.13, 1455.13, 1278.23, 981.76.

**(Tetrahydrofuran-2-yl) methyl (2E,4Z)-4-(((dimethylcarbamoyl) oxy) methyl) octa-2,4-dienoate (5ah)**. Light yellow oil, yield = 47%. Reaction time: 16 h. $^1H$ NMR ($CDCl_3$): δ = 7.31 (d, *J* = 16.0 Hz, 1H), 6.14 (t, *J* = 7.6 Hz, 1H), 6.03 (d, *J* = 16.0 Hz, 1H), 4.79 (s, 2H), 4.24-4.28 (m, 1H), 4.16-4.19 (m, 1H), 4.07-4.11 (m, 1H), 3.89-3.94 (m, 1H), 3.80-3.85 (m, 1H), 2.93 (brs, 3H), 2.85 (brs, 3H), 2.29-2.35 (m, 2H), 2.02-2.06 (m, 1H), 1.91-1.94 (m, 2H), 1.62-1.67 (m, 1H), 1.45-1.50 (m, 2H), 0.94 (t, *J* = 7.2 Hz, 3H). $^{13}C$ NMR ($CDCl_3$): δ = 167.32, 156.28, 147.21, 146.82, 132.46, 116.40, 76.64, 68.44, 66.43, 59.10, 36.47, 35.93, 30.70, 28.03, 25.66, 22.36, 13.68. HRMS (ESI): m/z calculated for $C_{17}H_{27}NO_5$ $[M+H]^+$: 348.1781, found: 348.1775. FTIR (KBr, $cm^{-1}$): 3673.50, 3592.46, 3584.99, 2971.27, 2899.98, 1704.33, 1651.49, 1434.83, 1078.21, 1049.63, 880.16.

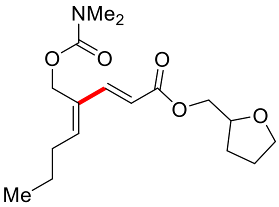


**Butyl (2E,4Z)-4-(((dimethyl carbamoyl) oxy) methyl) hepta-2,4-dienoate (5ba)**. Light yellow oil, yield = 48%. Reaction time: 16 h.$^{1}$H NMR ($CDCl_3$): δ = 7.27 (d, *J* = 16.0 Hz, 1H), 6.13 (t, *J* = 7.6 Hz, 1H), 6.00 (d, *J* = 15.6 Hz, 1H), 4.81 (s, 2H), 4.17 (t, *J* = 6.8 Hz, 2H), 2.93 (brs, 3H), 2.87 (brs, 3H), 2.33-2.40 (m, 2H), 1.63-1.70 (m, 2H),1.37-1.47 (m, 2H), 1.07 (t, *J* = 7.6 Hz, 3H), 0.96 (t, *J* = 7.2 Hz, 3H). $^{13}C$ NMR ($CDCl_3$): δ = 167.46, 156.28, 147.98, 146.58, 131.80, 116.92, 64.21, 59.07, 36.46, 35.90, 30.75, 22.11, 19.17, 13.72, 13.70. HRMS (ESI): m/z calculated for $C_{15}H_{25}NO_4$ $[M+H]^+$: 306.1676, found: 306.1671. FTIR (KBr, $cm^{-1}$): 3585.17, 3417.35, 2971.68, 2933.13, 2898.94, 1644.75, 1462.90, 1181.76, 1088.25, 880.64.

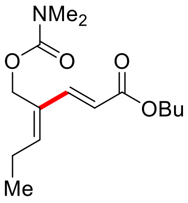


**Butyl (2E,4Z)-4-(((dimethylcarbamoyl) oxy) methyl) undeca-2,4- dienoate (5ca)**. Light yellow oil, yield = 51%. Reaction time: 16 h. $^1H$ NMR ($CDCl_3$): δ = 7.28 (d, *J* = 16.0 Hz, 1H),6.14 (t, *J* = 8.0 Hz, 1H), 5.98 (d,*J* = 15.6 Hz, 1H), 4.80 (s, 2H), 4.16 (t, *J* = 6.8 Hz, 2H), 2.93 (brs, 3H), 2.86(brs, 3H), 2.31-2.36 (m, 2H), 1.63-1.70 (m, 2H), 1.39-1.45 (m, 4H), 1.27-1.34 (m, 6H), 0.96 (t, *J* = 7.2 Hz, 3H), 0.89 (t, *J* = 6.8 Hz, 3H). $^{13}C$ NMR ($CDCl_3$): δ = 167.49, 156.29, 146.77, 146.62, 132.26, 116.79, 64.20, 59.12, 36.43, 35.90, 31.61, 30.76, 29.12, 28.88, 28.75, 22.54, 19.17, 14.10, 13.71. HRMS (ESI): m/z calculated for $C_{19}H_{33}NO_4$ $[M+H]^+$: 362.2302, found: 362.2294. FTIR (KBr, $cm^{-1}$): 3606.03, 3585.04, 3572.95, 3444.54, 2970.12, 2924.79, 2900.35, 1712.83, 1393.29, 1180.11, 1049.91.

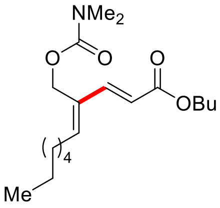

**Butyl (2E, 4Z)-4-(((diethylcarbamoyl) oxy) methyl) octa-2,4-dienoate (5da)**. Light yellow oil, yield = 54%. Reaction time: 16 h. $^1$H NMR (CDCl$_3$): δ = 7.20 (d, *J* = 16.0 Hz, 1H), 6.05 (t, *J* = 8.0 Hz, 1H), 5.90 (d, *J* = 16.0 Hz, 1H), 4.74 (s, 2H), 4.08 (t, *J* = 6.5 Hz, 2H), 3.17 (brs, 4H), 2.23-2.27 (m, 2H), 1.55-1.61 (m, 2H), 1.38-1.42 (m, 2H), 1.31-1.34 (m, 2H), 1.02 (brs, 6H), 0.84-0.89 (m, 6H). $^{13}$C NMR (CDCl$_3$): δ = 166.51, 154.62, 145.68, 145.35, 131.58, 115.87, 63.17, 57.79, 40.85, 40.27, 29.75, 29.66, 21.38, 18.16, 12.98, 12.71, 12.68, 12.49. HRMS (ESI): m/z calculated for $C_{18}H_{31}NO_4$ $[M+H]^+$: 326.2326, found: 326.233. FTIR (KBr, cm$^{-1}$): 3673.61, 3646.21, 3416.88, 2973.21, 2925.18, 2899.75, 1651.52, 1633.71, 1393.27, 1086.07, 1049.25.

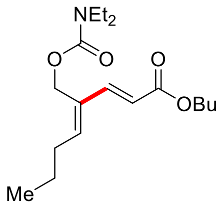


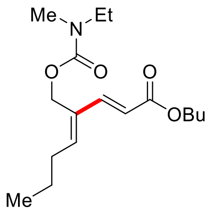


**Butyl (2E,4Z)-4-(((ethyl (methyl) carbamoyl) oxy) methyl) octa- 2,4-dienoate (5ea)**. Light yellow oil, yield = 53%. Reaction time: 16 h. $^1$H NMR (CDCl$_3$): δ = 7.20 (d, *J* = 16.0 Hz, 1H), 6.05 (t, *J* = 7.5 Hz, 1H), 5.90 (d, *J* = 15.5 Hz, 1H), 4.73 (s, 2H), 4.08 (t, *J* = 6.5 Hz, 2H), 3.20 (brs, 2H), 2.79 (brs, 3H), 2.22-2.27 (m, 2H), 1.55-1.61 (m, 2H), 1.31-1.42 (m, 4H), 1.01 (brs, 3H), 0.84-0.89 (m, 6H). $^{13}$C NMR (CDCl$_3$): δ = 166.51, 154.96, 145.64, 145.42, 131.52, 115.85, 63.19, 57.98, 42.74, 42.49, 32.99, 32.23, 29.75, 21.38, 18.17, 12.72, 12.69. HRMS (ESI): m/z calculated for $C_{17}H_{29}NO_4$ $[M+H]^+$: 312.2169, found: 312.2175. FTIR (KBr, cm$^{-1}$): 3626.50, 3564.47, 3472.80, 3444.45, 3417.50, 1713.58, 1698.70, 1651.39, 1393.06, 1385.11, 1049.09.

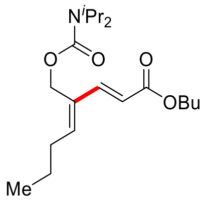


**Butyl (2E,4Z)-4-(((diisopropyl carbamoyl) oxy) methyl) octa-2,4- dienoate (5fa)**. Light yellow oil, yield = 62%. Reaction time: 16 h. $^1$H NMR (CDCl$_3$): δ = 7.22 (d, *J* = 15.5 Hz, 1H), 6.06 (t, *J* = 7.5 Hz, 1H), 5.89 (d, *J* = 16.0 Hz, 1H), 4.73 (s, 2H), 4.08 (t, *J* = 6.5 Hz, 2H), 3.64 (brs, 2H), 2.22-2.26 (m, 2H), 1.54-1.59 (m, 2H), 1.38-1.42 (m, 2H), 1.31-1.34 (m, 2H), 1.10 (brs, 12H), 0.84-0.88 (m, 6H). $^{13}$C NMR (CDCl$_3$): δ = 166.52, 154.44, 145.73, 145.32, 131.63, 116.01, 63.14, 57.24, 45.63, 44.39, 29.75, 29.64, 21.37, 20.31, 19.62, 18.14, 12.70, 12.68. HRMS (ESI): m/z calculated for $C_{20}H_{35}NO_4$ $[M+H]^+$: 354.2639, found: 354.2642. FTIR (KBr, cm$^{-1}$): 3606.11, 3592.56, 3417.83, 2971.03, 2923.44, 2900.30, 1644.79, 1633.73, 1470.64, 1065.76, 879.66.

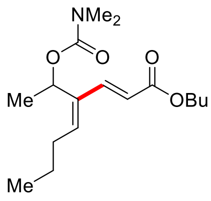


**Butyl (2E,4Z)-4-(1-((dimethyl carbamoyl) oxy) ethyl) octa-2,4- dienoate (5ga)**. Light yellow oil, yield = 48%. Reaction time: 16 h. $^1$H NMR (CDCl$_3$): δ = 7.11 (d, *J* = 16.0 Hz, 1H), 6.09 (d, *J* = 16.4 Hz, 1H), 5.87 (t, *J* = 7.6 Hz, 1H), 5.71 (q, *J* = 6.8 Hz, 1H), 4.08 (t, *J* = 6.8 Hz, 2H), 2.83 (s, 6H), 2.19-2.28 (m, 2H), 1.56-1.60 (m, 2H), 1.34-1.38 (m, 7H), 0.87 (m, *J* = 7.2 Hz, 6H). $^{13}$C NMR (CDCl$_3$): δ = 167.51, 155.80, 144.88, 142.02, 136.98, 117.41, 68.26, 64.15, 36.39, 35.95, 30.78, 30.55, 22.40, 19.83, 19.19, 13.87, 13.72. HRMS (ESI): m/z calculated for $C_{17}H_{29}NO_4$ $[M+H]^+$: 334.1989, found: 334.1983. FTIR (KBr, cm$^{-1}$): 3585.04, 3573.13, 3444.61, 2965.31, 2933.28, 1712.27, 1644.69, 1470.49, 1173.59, 1066.03, 1051.30.

**Butyl (E)-3-(6-((dimethyl carbamoyl) oxy) cyclohex-1-en-1-yl) acrylate (5ha)**. Light yellow oil, yield = 62%. Reaction time: 16 h. $^1$H NMR (CDCl$_3$): δ = 7.22 (d, *J* = 16.0 Hz, 1H), 6.37-6.39 (m, 1H), 5.88 (d, *J* = 16.0 Hz, 1H), 5.48 (s, 1H), 4.09-4.20 (m, 2H), 2.94 (brs, 3H), 2.85 (brs, 3H),2.30-2.37 (m, 1H), 2.14-2.21 (m, 1H), 1.99-2.03 (m, 1H),1.63-1.68 (m, 5H), 1.37-1.43 (m, 2H), 0.94 (t, *J* = 7.2 Hz, 3H). $^{13}$C NMR (CDCl$_3$): δ = 167.62, 156.04, 145.28, 142.24, 133.56, 116.20, 65.86,

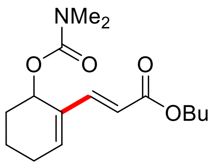

64.16, 36.39, 35.81, 30.72, 28.61, 26.44, 19.15, 16.91, 13.70. HRMS (ESI): m/z calculated for $C_{16}H_{25}NO_4$ $[M+H]^+$: 318.1676, found: 318.1666. FTIR (KBr, $cm^{-1}$): 3646.03, 3585.01, 3444.46, 3417.92, 3362.55, 3299.79, 1704.15, 1633.74, 1557.21, 1183.34.

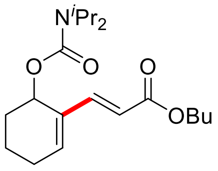


**Butyl (E)-3-(6-((diisopropyl carbamoyl) oxy) cyclohex-1-en-1-yl) acrylate (5ia)**. Light yellow solid, yield = 70%. Melting point: 60 °C. Reaction time: 16 h. $^1H$ NMR (500 MHz, $CDCl_3$): δ = 7.21 (d, $J$ = 16.0 Hz, 1H), 6.38 (dd, $J$ = 5.1 Hz, $J$ = 3.0 Hz, 1H), 5.85 (d, $J$ = 16.0 Hz, 1H), 5.56 (s, 1H), 4.08-4.17 (m, 3H), 3.64 (s, 1H), 2.32-2.37 (m, 1H), 2.13-2.22 (m, 1H), 2.04-2.06 (m, 1H), 1.69-1.80 (m, 3H), 1.59-1.64 (m, 2H), 1.36-1.40 (m, 2H), 1.19-1.26 (m, 12H), 0.92 (t, $J$ = 7.4 Hz, 3H). $^{13}C$ NMR (125 MHz, $CDCl_3$): δ = 166.70, 154.15, 144.29, 141.00, 132.92, 115.54, 64.14, 63.02, 45.50, 44.04, 29.71, 27.77, 25.43, 20.48, 19.55, 18.12, 16.38, 12.67. HR-MS (ESI): m/z calculated for $C_{20}H_{33}NO_4$ $[M+H]^+$: 352.2482, found: 352.2474. FTIR (KBr, $cm^{-1}$): 3626.64, 3585.12, 3132.84, 2594.87, 1694.13, 1667.52, 1644.79, 1492.63, 1393.90.

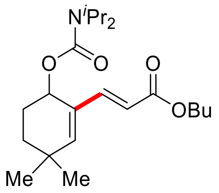


**Butyl (E)-3-(6-((diisopropyl carbamoyl) oxy)-3,3-dimethyl cyclohex-1-en-1-yl) acrylate (5ja)**. Yellow oil, yield = 95%. Reaction time: 16 h. $^1H$ NMR (500 MHz, $CDCl_3$): δ = 7.19 (d, $J$ = 16.0 Hz, 1H), 6.05 (s, 1H), 5.87 (d, $J$ = 16.0 Hz, 1H), 5.49 (t, $J$ = 3.4 Hz, 1H), 4.08-4.19 (m, 3H), 3.59 (s, 1H), 1.95-2.00 (m, 1H), 1.81-1.88 (m, 1H), 1.59-1.66 (m, 3H), 1.47 (dt, $J$ = 13.5 Hz, $J$ = 3.6 Hz, 1H), 1.36-1.42 (m, 2H), 1.18-1.20 (m, 15H), 1.02 (s, 3H), 0.92 (t, $J$ = 7.4 Hz, 3H). $^{13}C$ NMR (125 MHz, $CDCl_3$): δ = 166.54, 154.25, 150.44, 144.43, 130.39, 116.14, 64.07, 63.01, 45.04, 43.94, 32.08, 30.87, 29.72, 29.13, 25.75, 24.87, 20.63, 19.52, 18.12, 12.68. HR-MS (ESI): m/z calculated for $C_{22}H_{37}NO_4$ $[M+H]^+$: 380.2795, found: 380.2794. FTIR (KBr, $cm^{-1}$): 3564.62, 3132.68, 2498.35, 1694.03, 1644.77, 1463.00, 1400.02.

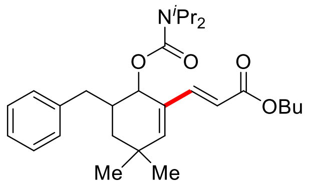


**Butyl (E)-3-(5-benzyl-6-((diisopropyl carbamoyl) oxy)-3,3-dimethyl cyclohex-1-en-1-yl) acrylate (5ka)**. Yellow solid, yield = 98%. Melting point: 78 °C. Reaction time: 16 h. $^1H$ NMR (500 MHz, $CDCl_3$): δ = 7.28 (t, $J$ = 7.5 Hz, 2H), 7.14-7.21 (m, 4H), 5.98 (s, 1H), 5.87 (d, $J$ = 16.0 Hz, 1H), 5.60 (d, $J$ = 8.5 Hz, 1H), 4.07-4.17 (m, 3H), 3.77 (brs, 1H), 3.03 (dd, $J$ = 14.0 Hz, $J$ = 3.5 Hz, 1H), 2.38 (dd, $J$ = 14.0 Hz, $J$ = 10.0 Hz, 1H), 2.20-2.27 (m, 1H), 1.58-1.63 (m, 3H), 1.46 (d, $J$ = 13.5 Hz, 1H), 1.36-1.42 (m, 2H), 1.24-1.26 (m, 9H), 1.15 (d, $J$ = 6.0 Hz, 3H), 1.00 (s, 3H), 0.98 (s, 3H), 0.92 (t, $J$ = 7.5 Hz, 3H). $^{13}C$ NMR (125 MHz, $CDCl_3$): δ = 166.53, 154.70, 147.77, 143.60, 139.16, 132.14, 128.02, 127.29, 124.90, 116.75, 71.48, 62.97, 45.68, 44.39, 38.63, 38.26, 37.38, 32.20, 29.76, 29.72, 26.62, 19.60, 19.44, 18.12, 12.66. HR-MS (ESI): m/z calculated for $C_{29}H_{43}NO_4$ $[M+H]^+$:470.3265, found: 470.3265. FTIR (KBr, $cm^{-1}$): 2956.58, 2923.91, 2852.94, 1715.52, 1694.06, 1633.89, 1462.95, 1383.23, 1174.07, 1049.07.

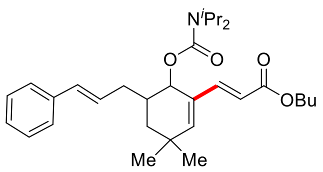


**Butyl (E)-3-(5-cinnamyl-6-((diisopropyl carbamoyl) oxy)-3,3-dimethyl cyclohex-1-en-1-yl) acrylate (5la)**. Yellow oil, yield = 63%. Reaction time: 16 h. $^1H$ NMR (500 MHz, $CDCl_3$): δ = 7.29 (d, $J$ = 7.0 Hz, 2H), 7.19-7.23 (m, 2H), 7.06-7.13 (m, 2H), 6.33 (d, $J$ = 17.5 Hz, 1H), 6.12-6.18 (m, 1H), 5.92 (s, 1H), 5.78 (d, $J$ = 16.0 Hz, 1H), 5.48 (d, $J$ = 8.0 Hz, 1H), 3.99-4.09 (m, 3H), 3.66 (brs, 1H), 2.37-2.42 (m, 1H), 2.01-2.13 (m, 2H),

1.49-1.58 (m, 3H), 1.25-1.32 (m, 3H), 1.11-1.18 (m, 12H), 1.03 (s, 3H), 0.98 (s, 3H), 0.84 (t, *J* = 7.5 Hz, 3H). $^{13}$C NMR (125 MHz, $CDCl_3$): δ = 166.52, 154.61, 147.82, 143.69, 136.60, 132.01, 130.93, 127.44, 127.08, 125.95, 125.06, 116.69, 70.75, 62.95, 45.62, 44.33, 38.62, 37.06, 34.69, 32.26, 29.75, 29.70, 27.10, 19.59, 19.42, 18.11, 12.66. HR-MS (ESI): m/z calculated for $C_{31}H_{45}NO_4$ $[M+H]^+$: 496.3421, found: 496.3420. FTIR (KBr, $cm^{-1}$): 2956.42, 2923.47, 2852.74, 1704.11, 1487.24, 1173.83, 1026.58.

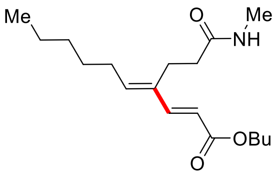


**Butyl (2E, 4E)-4-(3-(methylamino)-3-oxopropyl) deca-2, 4-dienoate (7aa)**

Yellow oil, yield = 74%, $^1$H NMR ($CDCl_3$): δ = 7.20 (d, 1H, *J* = 16.0 Hz), 5.91 (t, 1H, *J* = 7.5 Hz), 5.80 (d, 1H, *J* = 15.5 Hz), 5.77 (s, 1H), 4.17 (t, 2H, *J* = 6.5 Hz), 2.77 (d, 3H, *J* = 4.5 Hz), 2.60 (t, 2H, *J* = 8.0 Hz), 2.23-2.16 (m, 4H), 1.66-1.60 (m, 2H), 1.30-1.24 (m, 6H), 0.94 (t, 3H, *J* = 7.5 Hz), 0.87 (t, 3H, *J* = 6.5 Hz). $^{13}$C NMR ($CDCl_3$): δ = 171.64, 166.66, 147.10, 143.00, 134.71, 114.25, 63.20, 34.12, 30.50, 29.77, 27.84, 27.66, 25.31, 21.52, 18.17, 12.98, 12.72. HRMS (ESI): m/z calculated for $C_{18}H_{31}NO_3$ $[M+H]^+$: 310.2377, found: 310.2376. FTIR (KBr, $cm^{-1}$): 3444.64, 3209.23, 2359.11, 2340.91, 1732.46, 1715.48, 1634.20, 1455.61, 1398.46, 668.11.

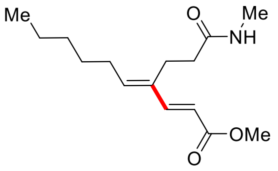


**Methyl (2E, 4E)-4-(3-(methylamino)-3-oxopropyl) deca-2, 4-dienoate (7ab)**

Yellow oil, yield = 70%, $^1$H NMR ($CDCl_3$): δ = 7.16 (d, 1H, *J* = 16.0 Hz), 5.86 (t, 1H, *J* = 7.5 Hz), 5.81 (s, 1H), 5.76 (d, 1H, *J* = 16.0 Hz), 3.67 (s, 3H), 2.72 (d, 3H, *J* = 5.0 Hz), 2.54 (t, 2H, *J* = 7.5 Hz), 2.17-2.11 (m, 4H), 1.36-1.30 (m, 2H), 1.23-1.20 (m, 4H), 0.82 (t, 3H, *J* = 7.0 Hz). $^{13}$C NMR ($CDCl_3$): δ = 171.65, 166.99, 147.42, 143.22, 134.70, 113.80, 50.47, 34.10, 30.51, 27.82, 27.67, 25.32, 21.52, 12.98. HRMS (ESI): m/z calculated for $C_{15}H_{25}NO_3$ $[M+H]^+$: 268.1907, found: 268.1907. FTIR (KBr, $cm^{-1}$): 3617.09, 3573.51, 3473.23, 3281.49, 3174.81, 2359.14, 2340.40, 1633.99, 1505.21, 1398.40.

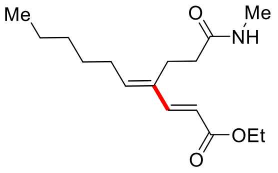


**Ethyl (2E, 4E)-4-(3-(methylamino)-3-oxopropyl) deca-2, 4-dienoate (7ac)**

Yellow solid, m.p.: 54.2 - 58.1 °C, yield = 64%, $^1$H NMR ($CDCl_3$): δ = 7.16 (d, 1H, *J* = 16.0 Hz), 5.86 (t, 1H, *J* = 7.5 Hz), 5.75 (d, 1H, *J* = 16.0 Hz), 5.64 (s, 1H), 4.14 (q, 2H, *J* = 7.0 Hz), 2.72 (d, 3H, *J* = 5.0 Hz), 2.54 (t, 2H, *J* = 7.5 Hz), 2.17-2.11 (m, 4H), 1.36-1.30 (m, 2H), 1.24-1.21 (m, 4H), 0.82 (t, 3H, *J* = 7.0 Hz). ($CDCl_3$): δ = 171.60, 166.55, 147.12, 143.05, 134.67, 114.26, 59.27, 34.14, 30.50, 27.83, 27.86, 25.32, 21.52, 13.30, 12.98. HRMS (ESI): m/z calculated for $C_{16}H_{27}NO_3$ $[M+H]^+$: 282.2064, found: 282.2075. FTIR (KBr, $cm^{-1}$): 3667.40, 3500.22, 3132.38, 3111.38, 1738.21, 1698.60, 1416.32, 1402.81, 1385.46, 1372.51.

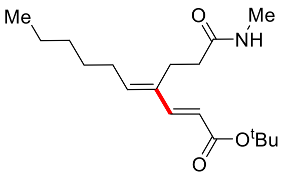


**tert-Butyl (2E, 4E)-4-(3-(methylamino)-3-oxopropyl) deca-2, 4-dienoate (7ad)**

Yellow oil, yield = 72%, $^1$H NMR ($CDCl_3$): δ = 7.06 (d, 1H, *J* = 16.0 Hz), 5.82 (t, 1H, *J* = 7.5 Hz), 5.67 (d, 2H, *J* = 15.5 Hz), 2.72 (d, 3H, *J* = 5.0 Hz),

2.53 (t, 2H, *J* = 7.5 Hz), 2.17-2.10 (m, 4H), 1.42 (s, 9H), 1.26-1.20 (m, 6H), 0.82 (t, 3H, *J* = 7.0 Hz). ($CDCl_3$): δ = 171.68, 165.93, 146.09, 142.32, 134.65, 116.10, 79.17, 34.17, 30.49, 27.87, 27.70, 27.18, 25.30, 21.56, 21.52, 12.99. HRMS (ESI): m/z calculated for $C_{18}H_{31}NO_3$ $[M+H]^+$: 310.2377, found: 310.2381. FTIR (KBr, $cm^{-1}$): 3708.66, 3573.42, 3472.97, 3238.43, 3175.40, 1770.36, 1621.52, 1445.93, 1402.75, 1385.94.

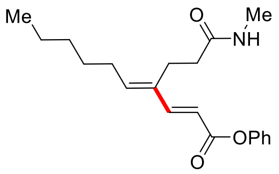


**Phenyl (2E, 4E)-4-(3-(methylamino)-3-oxopropyl) deca-2, 4-dienoate (7ae)**

Yellow oil, yield = 61%, $^1H$ NMR ($CDCl_3$): δ = 7.34 (d, 1H, *J* =16.0 Hz), 7.30 (d, 2H, *J* = 8.5 Hz), 7.16 (t, 1H, *J* = 7.0 Hz), 7.04 (d, 2H, *J* = 7.5 Hz), 5.94 (d, 1H, *J* = 9.0 Hz), 5.93 (d, 1H, *J* = 6.5 Hz), 5.72 (s, 1H), 2.70 (d, 3H, *J* = 4.5 Hz), 2.59 (t, 2H, *J* = 7.5 Hz), 1.38-1.32 (m, 2H), 1.24-1.21 (m, 4H), 0.83 (t, 3H, *J* = 7.0 Hz). $^{13}C$ NMR ($CDCl_3$): δ = 171.54, 164.97, 149.81, 149.21, 144.51, 134.82, 128.38, 124.67, 120.59, 113.26, 34.07, 30.51, 27.78, 25.32, 21.52, 12.99. HRMS (ESI): m/z calculated for $C_{20}H_{27}NO_3$ $[M+H]^+$: 330.2064, found: 330.2068. FTIR (KBr, $cm^{-1}$): 3592.68, 3507.55, 3355.15, 3281.14, 3168.14, 1715.07, 1470.72, 1463.13, 1402.57, 1193.55.

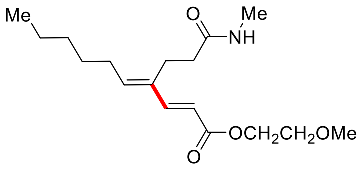


**2-Methoxyethyl (2E, 4E)-4-(3-(methylamino)-3-oxopropyl) deca-2, 4-dienoate (7af)**

Yellow oil, yield = 49%, $^1H$ NMR ($CDCl_3$): δ = 7.19 (d, 1H, *J* = 17 Hz), 5.88 (t, 1H, *J* = 7.5 Hz), 5.79 (d, 1H, *J* = 16.0 Hz), 5.55 (s, 1H), 4.24 (t, 2H, *J* = 4.5 Hz), 3.58 (t, 2H, *J* = 5.0 Hz), 3.34 (s, 3H), 2.73 (d, 3H, *J* = 4.5 Hz), 2.54 (t, 2H, *J* = 7.5 Hz), 2.16 (q, 4H, *J* = 7.5 Hz), 1.35-1.32 (m, 2H), 1.26-1.18 (m, 4H), 0.82 (t, 3H, *J* = 6.5 Hz). $^{13}C$ NMR ($CDCl_3$): δ = 171.54, 166.45, 147.74, 143.52, 134.63, 113.73, 63.59, 62.32, 57.97, 34.10, 30.50, 28.67, 27.81, 27.68, 25.32, 21.52, 12.98. HRMS (ESI): m/z calculated for $C_{17}H_{29}NO_4$ $[M+H]^+$: 312.2169, found: 312.2159. FTIR (KBr, $cm^{-1}$): 3345.97, 3299.45, 3238.58, 3133.20, 2355.83, 1770.39, 1567.89, 1445.97, 1487.11, 1402.59.

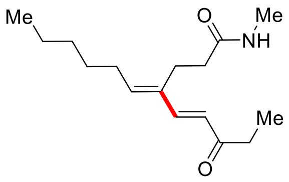


**(E)-N-methyl-4-((E)-3-oxopent-1-en-1-yl) dec-4-enamide (7ag)**

Yellow oil, yield = 48%, $^1H$ NMR ($CDCl_3$): δ = 7.04 (d, 1H, *J* = 16.0 Hz), 6.07 (d, 1H, *J* = 16.5 Hz), 5.90 (t, 1H, *J* = 7.5 Hz), 5.82 (s, 1H), 2.72 (d, 3H, *J* = 5.0 Hz), 2.56 (m, 4H), 2.17-2.13 (m, 4H), 1.36-1.31 (m, 2H), 1.26-1.21 (m, 4H), 1.05 (t, 3H, *J* = 7.5 Hz), 0.82 (t, 3H, *J* = 6.5 Hz). $^{13}C$ NMR ($CDCl_3$): δ = 200.59, 171.66, 145.02, 143.89, 135.07, 122.48, 34.19, 32.85, 30.51, 27.83, 27.82, 25.32, 21.52, 12.98, 7.32. HRMS (ESI): m/z calculated for $C_{16}H_{27}NO_2$ $[M+H]^+$: 266.2115, found: 266.2122. FTIR (KBr, $cm^{-1}$): 3667.72, 3554.76, 3473.21, 3332.30, 3281.56, 3175.09, 1682.38, 1633.83, 1538.24, 1402.19.

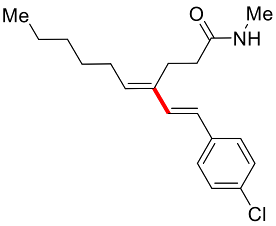


**(E)-4-((E)-4-chlorostyryl)-N-methyldec-4-enamide (7ah)**

Yellow oil, yield = 43%, $^1$H NMR ($CDCl_3$): δ = 7.26-7.24 (m, 2H), 7.20-7.18 (m, 2H), 6.59 (d, 1H, *J* =16.5 Hz), 6.36 (d, 1H, *J* = 16.5 Hz), 5.61 (t, 1H, *J* = 7.5 Hz), 5.33 (s, 1H), 2.73 (d, 3H, *J* = 4.5 Hz), 2.63 (t, 2H, *J* = 8.0 Hz), 2.23 (t, 2H, *J* = 8.0 Hz), 2.12 (q, 2H, *J* = 7.0 Hz), 1.36-1.31 (m, 2H), 1.25-1.21 (m, 4H), 0.84 (t, 3H, *J* = 6.5 Hz). $^{13}$C NMR ($CDCl_3$): δ = 171.98, 135.56, 135.36, 135.25, 131.68, 131.48, 127.70, 126.27, 123.18, 34.65, 30.56, 28.30, 27.35, 25.37, 21.58, 13.03. HRMS (ESI): m/z calculated for $C_{19}$ $H_{26}ClNO$ [M+H]$^+$: 320.1776, found: 320.1772. FTIR (KBr, cm$^{-1}$): 3520.26, 3444.39, 3299.62, 3172.49, 1698.63, 1682.35, 1574.34, 1455.19, 1422.87, 1402.54.

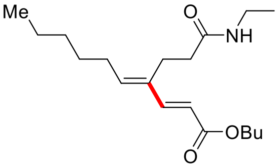


**Butyl (2E, 4E)-4-(3-(ethylamino)-3-oxopropyl) deca-2, 4-dienoate (7ba)**

Yellow oil, yield = 67%, $^1$H NMR ($CDCl_3$): δ = 7.16 (d, 1H，*J* = 15.5 Hz), 5.86 (t, 1H, *J* = 7.5 Hz), 5.76 (d, 1H, *J* = 16.0 Hz), 5.63 (s, 1H), 4.08 (t, 2H, *J* = 7.0 Hz), 3.21-2.19 (m, 2H), 2.54 (t, 2H, *J* = 7.5 Hz), 2.16-2.12 (m, 4H), 1.61-1.55 (m, 2H), 1.36-1.31 (m, 4H), 1.24-1.21 (m, 4H), 1.07 (t, 3H, *J* = 7.5 Hz), 0.89 (t, 3H, *J* = 7.5 Hz), 0.82 (t, 3H, *J* = 7.0 Hz). $^{13}$C NMR ($CDCl_3$): δ = 170.78, 166.67, 147.14, 142.98, 134.74, 114.26, 63.20, 34.23, 33.42, 30.50, 29.77, 27.83, 27.67, 21.52, 21.47, 18.17, 13.82, 12.98, 12.72. HRMS (ESI): m/z calculated for $C_{19}H_{33}NO_3$ [M+H]$^+$: 324.2533, found: 324.2525. FTIR (KBr, cm$^{-1}$): 3870.11, 3730.81, 3667.40, 3500.30, 3444.61, 3175.40, 1842.14, 1614.89, 1402.80, 1385.52.

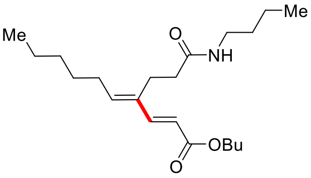


**Butyl (2E, 4E)-4-(3-(butylamino)-3-oxopropyl) deca-2, 4-dienoate (7ca)**

Yellow oil, yield = 64%, $^1$H NMR ($CDCl_3$): δ = 7.15 (d, 1H, *J* = 15.5 Hz), 5.86 (t, 1H, *J* = 7.5 Hz), 5.76 (d, 1H, *J* = 15.5 Hz), 5.74 (s, 1H), 4.08 (t, 2H, *J* = 7.0 Hz), 3.17-3.13 (m, 2H), 2.54 (t, 2H, *J* = 7.5 Hz), 2.17-2.12 (m, 4H), 1.59-1.55 (m, 2H), 1.40-1.21 (m, 12H), 0.89-0.80 (m, 9H). $^{13}$C NMR ($CDCl_3$): δ = 170.94, 166.69, 147.17, 143.00, 134.78, 114.23, 63.20, 38.55, 34.24, 30.65, 30.50, 29.76, 27.84, 27.66, 21.53, 21.51, 19.08, 18.17, 12.99, 12.74, 12.73. HRMS (ESI): m/z calculated for $C_{21}H_{37}NO_3$ [M+H]$^+$: 352.2846, found: 352.2856. FTIR (KBr, cm$^{-1}$): 3877.99, 3794.03, 3564.36, 3417.96, 3175.62, 1738.22, 1422.90, 1402.88, 1175.80, 984.53.

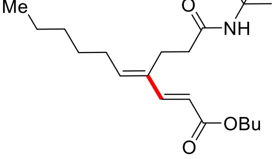


**Butyl (2E, 4E)-4-(3-(tert-butylamino)-3-oxopropyl) deca-2, 4-dienoate (7da)**

Yellow oil, yield = 41%, $^1$H NMR ($CDCl_3$): δ = 7.16 (d, 1H, *J* = 16.0 Hz), 5.86 (t, 1H, *J* = 7.5 Hz), 5.76 (d, 1H, *J* = 15.0 Hz), 5.15 (s, 1H), 4.09 (t, 2H, *J* = 7.0 Hz), 2.52 (t, 2H, *J* = 7.5 Hz), 2.17 (q, 2H, *J* = 7.5 Hz), 2.08 (t, 2H, *J* = 8.5 Hz), 1.61-1.55 (m, 2H), 1.38-1.32 (m, 4H), 1.27 (s, 9H), 1.24-1.22 (m, 4H), 0.89 (t, 3H, *J* = 7.5 Hz), 0.83 (t, 3H, *J* = 7.0 Hz). ($CDCl_3$): δ = 170.16, 166.68, 147.21, 142.83, 134.91, 114.32, 63.19, 50.21, 35.14, 30.50, 29.79,

27.83, 27.81, 27.67, 21.52, 21.39, 18.18, 12.98, 12.72. HRMS (ESI): m/z calculated for $C_{21}H_{37}NO_3$ $[M+H]^+$: 352.2846, found: 352.2853. FTIR (KBr, $cm^{-1}$): 3851.14, 3748.45, 3667.76, 3585.32, 3444.81, 3175.94, 2359.02, 2340.97, 1398.09, 668.17.

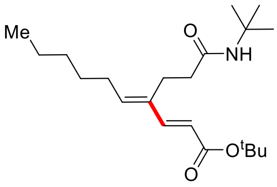


***tert*-Butyl (2E, 4E)-4-(3-(tert-butylamino)-3-oxopropyl) deca-2, 4-dienoate (7dd)**

Yellow oil, yield = 63%, $^1$H NMR ($CDCl_3$): δ = 7.07 (d, 1H, *J* = 16.0 Hz), 5.81 (t, 1H, *J* = 7.5 Hz), 5.68 (d, 1H, *J* = 16.0 Hz), 5.20 (s, 1H), 2.50 (t, 2H, *J* = 8.0 Hz), 2.15 (q, 2H, *J* = 7.5 Hz), 2.08 (t, 2H, *J* = 8.0 Hz), 1.42 (s, 9H), 1.26 (s, 9H), 1.23-1.21 (m, 2H), 0.82 (t, 3H, *J* = 6.0 Hz). ($CDCl_3$): δ = 170.27, 165.96, 146.23, 142.12, 134.90, 116.16, 79.13, 50.19, 35.21, 30.48, 27.86, 27.81, 27.62, 27.19, 21.52, 21.44, 12.98. HRMS (ESI): m/z calculated for $C_{21}H_{37}NO_3$ $[M+H]^+$: 352.2846, found: 352.2838. FTIR (KBr, $cm^{-1}$): 3850.99, 3708.58, 3626.61, 3444.68, 2359.12, 2340.84, 1651.65, 1557.38, 1397.78, 668.21.

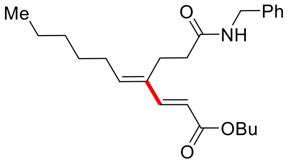


**Butyl (2E, 4E)-4-(3-(benzylamino)-3-oxopropyl) deca-2, 4-dienoate (7ea)**

White solid, m.p.: 65.0 – 66.1 °C, yield = 43%, $^1$H NMR ($CDCl_3$): δ = 7.27-7.24 (m, 2H), 7.21-7.18 (m, 3H), 7.14 (d, 1H, *J* = 16 Hz), 5.85 (t, 1H, *J* = 7.5 Hz), 5.79 (s, 1H), 5.75 (d, 1H, *J* = 15.5 Hz), 4.34 (d, 2H, *J* = 5.5 Hz), 4.06 (t, 2H, *J* = 7.0 Hz), 2.57 (t, 2H, *J* = 7.5 Hz), 2.20 (t, 2H, *J* = 8.0 Hz), 2.14 (q, 2H, *J* = 7.5 Hz), 1.59-1.53 (m, 2H), 1.36-1.29 (m, 4H), 1.24-1.20 (m, 4H), 0.87 (t, 3H, *J* = 7.0 Hz), 0.82 (t, 3H, *J* = 6.5 Hz). $^{13}$C NMR ($CDCl_3$): δ = 170.74, 166.61, 147.05, 143.02, 137.16, 134.63, 127.68, 126.85, 126.52, 114.32, 63.20, 42.71, 34.19, 30.50, 29.77, 28.88, 27.82, 27.67, 21.52, 18.17, 12.98, 12.72. HRMS (ESI): m/z calculated for $C_{24}H_{35}NO_3$ $[M+H]^+$: 386.2690, found: 386.2684. FTIR (KBr, $cm^{-1}$): 3851.06, 3742.30, 3626.03, 3564.61, 1732.18, 1698.67, 1519.89, 1455.29, 1402.61, 1385.43.

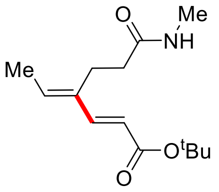


**tert-Butyl (2E, 4E)-4-ethylidene-7-(methylamino)-7-oxohept-2-enoate (7fd).** Yellow oil, yield = 46%, $^1$H NMR ($CDCl_3$): δ = 7.06 (d, 1H, *J* = 16.0 Hz), 5.93 (q, 1H, *J* = 7.0 Hz), 5.71 (s, 1H), 5.67 (d, 1H, *J* = 16.0 Hz), 2.72 (d, 3H, *J* = 4.5 Hz), 2.54 (t, 2H, *J* = 7.5 Hz), 2.17 (t, 2H, *J* = 8.5 Hz), 1.75 (d, 3H, *J* = 7.0 Hz), 1.42 (s, 9H). ($CDCl_3$): δ = 171.75, 165.96, 145.89, 136.19, 135.78, 115.97, 79.21, 33.87., 27.18, 25.33, 21.23, 13.42. HRMS (ESI): m/z calculated for $C_{14}H_{23}NO_3$ $[M+H]^+$: 254.1751, found: 254.1744. FTIR (KBr, $cm^{-1}$): 3850.74, 3620.39, 3564.35, 3444.58, 3175.64, 1732.09, 1698.52, 1574.26, 1402.63, 1256.88.

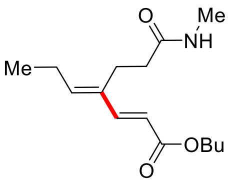


**Butyl (2E, 4E)-4-(3-(methylamino)-3-oxopropyl) hepta-2, 4-dienoate (7ga).** Yellow oil, yield = 63%, $^1$H NMR ($CDCl_3$): δ = 7.15 (d, 1H, *J* = 16.0 Hz), 5.85 (t, 1H, *J* = 7.5 Hz), 5.76 (d, 1H, *J* = 16.0 Hz), 5.58 (s, 1H), 4.08 (t, 2H, *J* = 6.5 Hz), 2.72 (d, 3H, *J* = 5.0 Hz), 2.55 (t, 2H, *J* = 7.5 Hz), 2.18-2.14 (m, 4H),

1.38-1.30 (m, 2H), 0.96 (t, 3H, *J* = 7.5 Hz), 0.89 (t, 3H, *J* = 7.5 Hz). $^{13}$C NMR ($CDCl_3$): δ = 171.62, 166.63, 147.06, 144.30, 134.21, 114.40, 63.22, 34.21, 29.76, 25.32, 21.47, 21.02, 18.17, 12.72, 12.64. HRMS (ESI): m/z calculated for $C_{15}H_{25}NO_3$ $[M+H]^+$: 268.1907, found: 268.1914. FTIR (KBr, $cm^{-1}$): 3851.03, 3742.29, 3626.61, 3585.11, 3132.41, 2339.53, 1747.68, 1651.61, 1402.85, 871.70.

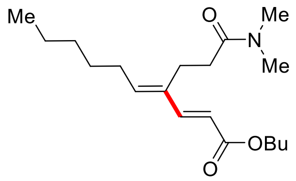


**Butyl (2E, 4E)-4-(3-(dimethylamino)-3-oxopropyl) deca-2, 4-dienoate (7ha)**

Yellow oil, yield = 60%, $^1$H NMR ($CDCl_3$): δ = 7.18 (d, 1H, *J* = 16 Hz), 5.86 (t, 1H, *J* = 7.5 Hz), 5.76 (d, 1H, *J* = 15.5 Hz), 4.09 (t, 2H, *J* = 6.5 Hz), 2.89 (d, 6H, *J* = 1.5 Hz), 2.54 (t, 2H, *J* = 8.0 Hz), 2.29 (t, 2H, *J* = 8.5 Hz), 2.15 (q, 2H, *J* = 7.5 Hz), 1.61-1.55 (m, 2H), 1.36-1.32 (m, 4H), 1.25-1.21 (m, 4H), 0.89 (t, 3H, *J* = 7.5 Hz), 0.82 (t, 3H, *J* = 7.0 Hz). $^{13}$C NMR ($CDCl_3$): δ = 171.04, 166.64, 147.22, 142.59, 135.21, 114.27, 63.16, 36.16, 34.43, 30.98, 30.54, 29.78, 27.84, 27.67, 21.51, 21.07, 18.18, 12.99, 12.73. HRMS (ESI): m/z calculated for $C_{19}H_{33}NO_3$ $[M+H]^+$: 324.2543, found: 324.2553. FTIR (KBr, $cm^{-1}$): 3584.18, 3507.21, 3458.58, 3175.58, 1841.97, 1770.21, 1445.91, 1422.86, 1402.65, 1360.55.

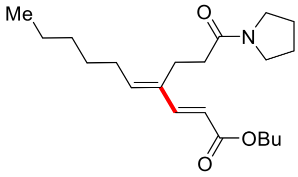


**Butyl (2E, 4E)-4-(3-oxo-3-(pyrrolidin-1-yl) propyl) deca-2, 4-dienoate (7ia)**

Yellow oil, yield = 58%, $^1$H NMR ($CDCl_3$): δ = 7.18 (d, 1H, *J* = 15.5 Hz), 5.86 (t, 1H, *J* = 7.0 Hz), 5.77 (d, 1H, *J* = 16.0 Hz), 4.08 (t, 2H, *J* = 6.5 Hz), 3.40 (t, 2H, *J* = 7.0 Hz), 3.28 (t, 2H, *J* = 6.5 Hz), 2.56 (t, 2H, *J* = 7.5 Hz), 2.25 (t, 2H, *J* = 8.5 Hz), 2.16 (q, 2H, *J* = 7.5 Hz), 1.89-1.84 (m, 2H), 1.81-1.77 (m, 2H), 1.61-1.55 (m, 2H), 1.36-1.31 (m, 4H), 1.23-1.21 (m, 4H), 0.88 (t, 3H, *J* = 7.0 Hz), 0.82 (t, 3H, *J* = 7.0 Hz). $^{13}$C NMR ($CDCl_3$): δ = 169.58, 166.68, 147.25, 142.64, 135.21, 114.23, 63.15, 45.55, 44.71, 32.37, 30.53, 29.76, 27.85, 27.66, 25.08, 23.38, 21.53, 20.79, 18.17, 13.00, 12.74. HRMS (ESI): m/z calculated for $C_{21}H_{35}NO_3$ $[M+H]^+$: 350.2690, found: 350.2696. FTIR (KBr, $cm^{-1}$): 3870.67, 3654.25, 3444.67, 3175.56, 2351.62, 1682.90, 1515.19, 1402.64, 1257.83, 1012.08.

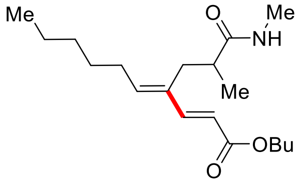


**Butyl (2E, 4E)-4-(2-methyl-3-(methylamino)-3-oxopropyl) deca-2, 4-dienoate (7ja)**

White solid, m.p.: 50.0 - 58.9 °C, yield = 62%, $^1$H NMR ($CDCl_3$): δ = 7.17 (d, 1H, *J* = 16.0 Hz), 5.92 (t, 1H, *J* = 7.5 Hz), 5.70 (d, 1H, *J* = 16.0 Hz), 5.23 (s, 1H), 4.10 (t, 2H, *J* = 7.0 Hz), 2.69 (d, 3H, *J* = 4.5 Hz), 2.61-2.56 (m, 1H), 2.27-2.21 (m, 2H), 2.15-2.10 (m, 2H), 1.62-1.56 (m, 2H), 1.37-1.30 (m, 4H), 1.23-1.21 (m, 4H), 1.08 (d, 3H, *J* = 6.5 Hz), 0.89 (t, 3H, *J* = 7.5 Hz),0.83 (t, 3H, *J* = 7.0 Hz). $^{13}$C NMR ($CDCl_3$): δ = 174.93, 166.62, 147.60, 144.32, 133.48, 114.38, 63.26, 39.12, 30.49, 30.03, 29.78, 27.91, 27.84, 25.28, 21.53, 18.18, 16.64, 12.99, 12.73. HRMS (ESI): m/z calculated for $C_{19}H_{33}NO_3$ $[M+H]^+$: 324.2533, found: 324.2533. FTIR (KBr, $cm^{-1}$): 3708.29, 3664.33, 3417.82, 3145.01, 2337.02, 1738.22, 1470.78, 1397.73, 1372.57, 1360.60.

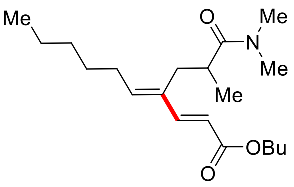


**Butyl (2E, 4E)-4-(3-(dimethylamino)-2-methyl-3-oxopropyl) deca-2, 4-dienoate (7ka)**

Yellow oil, yield = 45%, $^1$H NMR ($CDCl_3$): δ = 7.19 (d, 1H, *J* = 15.5 Hz), 5.91 (t, 1H, *J* = 7.5 Hz), 5.73 (d, 1H, *J* = 16.0 Hz), 4.10 (t, 2H, *J* = 7.0 Hz), 2.88 (s, 3H), 2.85 (s, 3H), 2.58 (q, 1H, *J* = 8.0 Hz), 2.32 (q, 1H, *J* = 7.0 Hz), 2.16 (q, 2H, *J* = 7.0 Hz), 1.62-1.56 (m, 2H), 1.37-1.30 (m, 5H), 1.23-1.18 (m, 4H), 1.04 (d, 3H, *J* = 7.0 Hz), 0.89 (t, 3H, *J* = 7.5 Hz), 0.82 (t, 3H, *J* = 6.5 Hz). $^{13}$C NMR ($CDCl_3$): 174.91, 166.64, 147.99, 144.22, 133.63, 114.22, 63.22, 36.41, 34.73, 32.95, 30.51, 29.85, 29.77, 27.95, 27.85, 21.54, 18.18, 16.74, 13.00, 12.74. HRMS (ESI): m/z calculated for $C_{20}H_{35}NO_3$ $[M+H]^+$: 338.2690, found: 338.2696. FTIR (KBr, $cm^{-1}$): 3850.98, 3731.06, 3444.53, 3175.53, 2357.85, 2338.71, 1732.20, 1557.32, 1470.70, 1402.66.

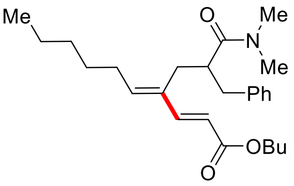


**Butyl (2E, 4E)-4-(2-benzyl-3-(dimethylamino)-3-oxopropyl) deca-2, 4-dienoate (7la)**

Yellow oil, yield = 40%, $^1$H NMR ($CDCl_3$): δ = 7.21-7.08 (m, 6H), 5.88 (t, 1H, *J* = 7.5 Hz), 5.55 (d, 1H, *J* = 16.0 Hz), 4.08 (t, 2H, *J* = 7.0 Hz), 3.01-2.98 (m, 1H), 2.90 (q, 1H, *J* = 9.0 Hz), 2.72 (s, 3H), 2.67-2.63 (m, 2H), 2.48 (s, 3H), 2.34 (q, 1H, *J* = 5.0 Hz), 2.16-2.08 (m, 2H), 1.63-1.56 (m, 2H), 1.37-1.28 (m, 3H), 1.24-1.18 (m, 3H), 0.90 (t, 3H, *J* = 7.5 Hz), 0.82 (t, 3H, *J* = 7.0 Hz). $^{13}$C NMR ($CDCl_3$): 173.60, 166.65, 147.68, 144.33, 138.62, 133.46, 127.98, 127.34, 125.37, 114.28, 63.18, 41.23, 38.57, 36.25, 34.63, 30.50, 29.73, 28.47, 27.85, 21.54, 18.18, 13.02, 12.75. HRMS (ESI): m/z calculated for $C_{26}H_{39}NO_3$ $[M+H]^+$: 414.3003, found: 414.3011. FTIR (KBr, $cm^{-1}$): 3708.55, 3605.93, 3444.59, 3145.02, 1807.97, 1714.32, 1667.40, 1621.44, 1402.61, 1258.45.

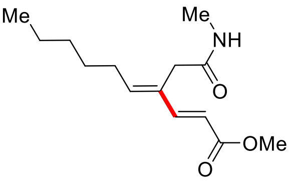


**Methyl (2E, 4E)-4-(2-(methylamino)-2-oxoethyl) deca-2, 4-dienoate (7ma)**

Yellow oil, yield = 51%, $^1$H NMR ($CDCl_3$): δ = 7.28 (d, 1H, *J* = 16.0 Hz), 6.12 (t, 1H, *J* = 7.5 Hz), 5.78 (d, 1H, *J* = 15.5 Hz), 5.49 (s, 1H), 3.68 (s, 3H), 3.17 (s, 2H), 2.70 (d, 3H, *J* = 5.0 Hz), 2.17 (q, 2H, *J* = 7.5 Hz), 1.41-1.35 (m, 2H), 1.24-1.22 (m, 4H), 0.83 (t, 3H, *J* = 7.0 Hz). $^{13}$C NMR ($CDCl_3$): 168.66, 166.56, 146.61, 145.56, 129.98, 115.48, 50.63, 33.71, 30.50, 28.06, 27.51, 25.50, 21.43, 12.95. HRMS (ESI): m/z calculated for $C_{14}H_{23}NO_3$ $[M+H]^+$: 254.1751, found: 254.1746. FTIR (KBr, $cm^{-1}$): 3929.33, 3720.32, 3585.22, 3444.70, 3175.22, 2922.87, 2352.05, 1760.19, 1651.61, 1402.69.

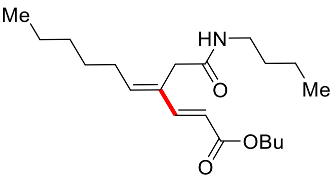


**Butyl (2E, 4E)-4-(2-(butylamino)-2-oxoethyl) deca-2, 4-dienoate (7na)**

Yellow oil, yield = 45%, $^1$H NMR ($CDCl_3$): δ = 7.25 (d, 1H, *J* = 16.0 Hz), 6.12 (t, 1H, *J* = 7.5 Hz), 5.78 (d, 1H, *J* = 16.0 Hz), 52.46 (s, 1H), 4.09 (t, 2H, *J* = 6.5 Hz), 3.17 (s, 2H), 3.15 (q, 2H, *J* = 7.0 Hz), 2.17 (q, 2H, *J* = 7.0 Hz), 1.59-1.54 (m,

2H), 1.35-1.30 (m, 6H), 1.24-1.20 (m, 6H), 0.88 (t, 3H, *J* = 7.5 Hz), 0.84-0.80 (m, 6H). $^{13}$C NMR ($CDCl_3$): 167.91, 166.22, 146.26, 145.17, 130.17, 116.07, 63.35, 38.33, 33.97, 30.52, 29.72, 28.68, 28.06, 27.58, 21.46, 18.97, 18.15, 12.94, 12.69, 12.65. HRMS (ESI): m/z calculated for $C_{20}H_{35}NO_3$ $[M+H]^+$: 338.2690, found: 338.2683. FTIR (KBr, $cm^{-1}$): 3742.04, 3444.56, 3355.10, 3195.06, 2922.38, 1828.05, 1704.18, 1557.15, 1402.99, 1018.94.

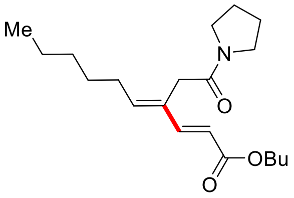


**Butyl (2E, 4E)-4-(2-oxo-2-(pyrrolidin-1-yl) ethyl) deca-2, 4-dienoate (7oa)**

Yellow oil, yield = 43%, $^1$H NMR ($CDCl_3$): δ = 7.29 (d, 1H, *J* = 16.0 Hz), 6.07 (t, 1H, *J* = 7.5 Hz), 5.62 (d, 1H, *J* = 15.5 Hz), 4.06 (t, 2H, *J* = 7.0 Hz), 3.43 (q, 4H, *J* = 7.0 Hz), 3.16 (s, 2H), 2.14-2.09 (m, 2H), 1.94 (t, 2H, *J* = 7.0 Hz), 1.81 (t, 2H, *J* = 7.0 Hz), 1.59-1.53 (m, 2H), 1.38-1.30 (m, 4H), 1.26-1.18 (m, 4H), 0.88 (t, 3H, *J* = 7.5 Hz), 0.82 (t, 3H, *J* = 7.0 Hz). $^{13}$C NMR ($CDCl_3$): 166.74, 166.43, 147.48, 144.20, 129.93, 114.67, 63.11, 45.74, 45.01, 32.02, 30.55, 29.79, 28.12, 27.57, 25.23, 23.33, 21.48, 18.18, 12.98, 12.72. HRMS (ESI): m/z calculated for $C_{20}H_{33}NO_3$ $[M+H]^+$: 336.2533, found: 336.2531. FTIR (KBr, $cm^{-1}$): 3584.98, 3654.36, 2957.58, 1822.97, 1732.16, 1698.72, 1614.83, 1434.74, 1402.70, 443.33.

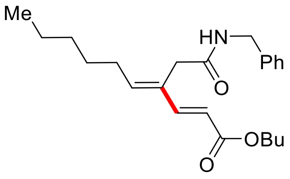


**Butyl (2E, 4E)-4-(2-(benzylamino)-2-oxoethyl)deca-2,4-dienoate (7pa)**

Yellow oil, yield = 51%, $^1$H NMR ($CDCl_3$): δ = 7.25-7.2 (m, 5H), 7.12 (d, 1H, *J* = 7.0 Hz), 6.09 (t, 1H, *J* = 7.5 Hz), 5.81 (d, 1H, *J* = 15.5 Hz), 5.78 (s,1H), 4.34 (d, 2H, *J* = 5.5 Hz), 4.08 (t, 2H, *J* = 6.5 Hz), 3.23 (s, 2H), 2.15 (q, 2H, *J* = 7.0 Hz), 1.60-1.55 (m, 2H), 1.36-1.31 (m, 4H), 1.22-1.17 (m, 4H), 0.89 (t, 3H, *J* = 7.5 Hz), 0.81 (t, 3H, *J* = 6.5 Hz). $^{13}$C NMR ($CDCl_3$): 167.98, 166.16, 146.23, 145.43, 136.92, 129.81, 127.66, 126.51, 126.49, 116.05, 63.36, 42.57, 33.84, 30.49, 29.70, 28.08, 27.51, 21.42, 18.15, 12.94, 12.72. HRMS (ESI): m/z calculated for $C_{23}H_{33}NO_3$ $[M+H]^+$: 372.2533, found: 372.2529. FTIR (KBr, $cm^{-1}$): 3799.54, 3606.33, 3573.73, 3345.83, 3144.66, 1778.96, 1673.72, 1495.22, 1470.85, 1398.08.

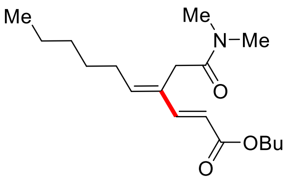


**Butyl (2E, 4E)-4-(2-(dimethylamino)-2-oxoethyl) deca-2, 4-dienoate (7qa)**

Yellow oil, yield = 40%, $^1$H NMR ($CDCl_3$): δ = 7.29 (d, 1H, *J* = 16.0 Hz), 6.07 (t, 1H, *J* = 7.5 Hz), 5.58 (d, 1H, *J* = 16.0 Hz), 4.06 (t, 2H, *J* = 6.5 Hz), 3.21 (s, 2H), 3.02 (s, 3H), 2.89 (s, 3H), 2.11 (q, 2H, *J* = 7.5Hz), 1.59-1.53 (m, 2H), 1.38-1.30 (m, 4H), 1.24-1.21 (m, 4H), 0.87 (t, 3H, *J* = 7.5 Hz), 0.82 (t, 3H, *J* = 7.0 Hz). $^{13}$C NMR ($CDCl_3$): 168.20, 166.40, 147.47, 144.16, 130.16, 114.53, 63.11, 36.34, 34.74, 30.53, 29.76, 28.68, 28.03, 27.54, 21.47, 18.16, 12.99, 12.73. HRMS (ESI): m/z calculated for $C_{18}H_{31}NO_3$ $[M+H]^+$: 310.2413, found: 310.2413. FTIR (KBr, $cm^{-1}$): 3742.88, 3564.59, 3299.49, 3145.53, 2924.32, 1704.30, 1633.87, 1567.88, 1487.19, 1397.98.

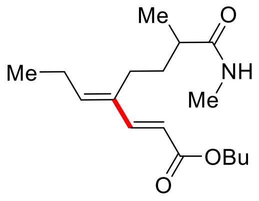


**Butyl (2E, 4E)-7-methyl-8-(methylamino)-8-oxo-4-propylideneoct-2-enoate (7ra)**

Yellow oil, yield = 40%, $^1$H NMR (CDCl$_3$): δ = 7.16 (d, 1H, *J* = 15.5 Hz), 5.80 (t, 1H, *J* = 7.5 Hz), 5.75 (d, 1H, *J* = 15.5 Hz), 4.09 (t, 2H, *J* = 7.0 Hz), 2.96 (s, 3H), 2.90 (s, 3H), 2.64-2.57 (m, 1H), 2.18-2.10 (m, 4H), 1.61-1.56 (m, 2H), 1.37-1.32 (m, 4H), 1.06 (d, 3H, *J* = 6.0 Hz), 0.97 (t, 3H, *J* = 7.5 Hz), 0.89 (t, 3H, *J* = 7.0 Hz). $^{13}$C NMR (CDCl$_3$): 174.78, 166.75, 147.63, 143.36, 135.42, 114.28, 63.12, 36.09, 34.62, 34.40, 31.69, 29.80, 23.25, 20.95, 18.18, 16.48, 12.73. HRMS (ESI): m/z calculated for $C_{18}H_{31}NO_3$ [M+H]$^+$: 310.2377, found: 310.2382. FTIR (KBr, cm$^{-1}$): 3818.98, 3742.21, 3417.80, 3175.44, 2351.88, 1778.86, 1644.87, 1455.33, 1446.12, 1397.93.

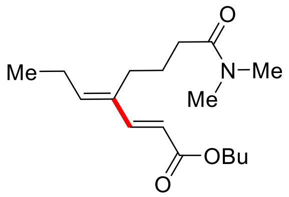


**Butyl (2E, 4E)-8-(dimethylamino)-8-oxo-4-propylideneoct-2-enoate (7sa)**

Yellow oil, yield = 37%, $^1$H NMR (CDCl$_3$): δ = 7.17 (d, 2H, *J* = 16.0 Hz), 5.82 (d, 1H, *J* = 15.0 Hz), 5.81 (t, 1H, *J* = 12.0 Hz), 4.09 (t, 2H, *J* = 12.0 Hz), 2.91 (s, 3H), 2.88 (s, 3H), 2.26-2.22 (m, 4H), 2.18-2.12 (m, 2H), 1.70-1.64 (m, 2H), 1.61-1.55 (m, 2H), 1.38-1.30 (m, 2H), 0.97 (t, 3H, *J* = 7.5 Hz), 0.89 (t, 3H, *J* = 7.5 Hz). $^{13}$C NMR (CDCl$_3$): 171.46, 166.77, 147.64, 143.59, 135.18, 114.38, 63.11, 36.15, 34.37, 31.70, 29.77, 24.90, 22.80, 21.05, 18.17, 12.74, 12.71. HRMS (ESI): m/z calculated for $C_{17}H_{29}NO_3$ [M+H]$^+$: 296.2220, found: 296.2215. FTIR (KBr, cm$^{-1}$): 3835.45, 3564.53, 3520.82, 3444.75, 3144.85, 1823.07, 1770.37, 1434.78, 1422.71, 1402.76.

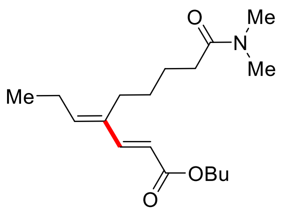


**Butyl (2E, 4E)-9-(dimethylamino)-9-oxo-4-propylidenenon-2-enoate (7ta)**

Yellow oil, yield = 18%, $^1$H NMR (CDCl$_3$): δ = 7.16 (d, 2H, *J* = 16.0 Hz), 5.80 (t, 1H, *J* = 7.5 Hz), 5.72 (d, 1H, *J* = 15.5 Hz), 4.09 (t, 2H, *J* = 6.5 Hz), 2.93 (s, 3H), 2.87 (s, 3H), 2.26-2.10 (m, 6H), 1.65-1.56 (m, 4H), 1.38-1.32 (m, 4H), 0.97 (t, 3H, *J* = 7.5 Hz), 0.89 (t, 3H, *J* = 7.5 Hz). $^{13}$C NMR (CDCl$_3$): 171.81, 166.76, 147.74, 143.14, 135.56, 114.22, 63.12, 36.28, 34.38, 32.20, 29.81, 27.63, 25.47, 24.29, 21.08, 18.19, 12.73, 12.68. HRMS (ESI): m/z calculated for $C_{18}H_{31}NO_3$ [M+H]$^+$: 310.2377, found: 310.2375. FTIR (KBr, cm$^{-1}$): 3748.39, 3654.37, 3472.94, 3144.42, 1714.95, 1694.22, 1557.31, 1495.04, 1434.82, 1398.22.

**Supplementary Table 4.** Competition Experiments Using Alcohols (4, 5 and 6 membered *endo*- or *exo*-palladacycle)

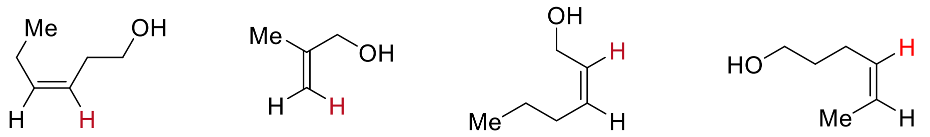

**1a** 5-*exo* **8** 5-*endo* **1v** 4-*exo* **1z'** 6-*exo*

**2a**, Cond. A, 2 h

| alcohol | *vs* | alcohol | by endo-/*exo*-palladacycles: 5-*exo* | 5-*endo* | 4-*exo* | 6-*exo* |
|---|---|---|---|---|---|---|
| **1a** | | **8** | 60% | 18% | - | - |
| **1v** | | **8** | - | 28% | 15% | - |
| **1z'** | | **1v** | - | - | 13% | < 5% |

⇨ 5-*exo* > 5-*endo* > 4-*exo* > 6-*exo*

A 10 mL vial was charged with $Pd(OAc)_2$ (8.9 mg, 10 mol%, 0.04 mmol), Ac-Phe-OH (41.4 mg, 0.20 mmol, 50 mol%), $Ag_2CO_3$ (165.4 mg, 0.6mmol, 1.5 equiv), $Cs_2CO_3$ (39.0 mg, 0.12 mmol, 30 mol%), $CF_3CH_2OH$ (289 μL, 4.0 mmol, 10.0 equiv), 1,4-dioxane (0.6 mL). Then, **2a** (0.8 mmol, 4.0 equiv**)**, two **alcohols** (0.2 mmol each) were added into the solution in sequence. The vial was sealed under Ar and heated to 70 °C with stirring for 2 h. After cooling down, the mixture was concentrated in vacuo and purified by column chromatography, affording the products.

**Supplementary Table 5.** Competition Experiments Using Amides (5, 6, 7 and 8 membered *exo*-palladacycle)

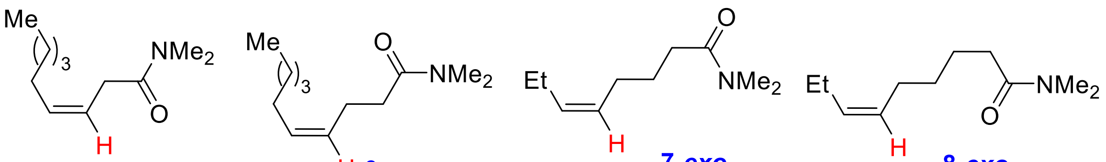

**6q** 5-*exo* **6h** 6-*exo* **6r** 7-*exo* **6t** 8-*exo*

**2a**, Cond. C

| amide | *vs* | amide | alkenylation by *exo*-palladacycles: 5-*exo* | 6-*exo* | 7-*exo* | 8-*exo* |
|---|---|---|---|---|---|---|
| **6q** | | **6r** | 40% | - | 10% | - |
| **6q** | | **6h** | 13% | 34% | - | - |
| **6r** | | **6t** | - | - | 15% | 10% |

⇨ 6-*exo* > 5-*exo* > 7-*exo* > 8-*exo*

An oven-dried screw-cap vial was charged with $Pd(OAc)_2$ (10.0 mol%, 0.02 mmol), Ac-phe-OH (50.0 mol%, 0.1 mmol), $Ag_2CO_3$ (1.5 equiv, 0.3 mmol), LiOH (30 mol%, 0.06 mmol) and MeCN (1.0 mL). Then, two amides (0.2 mmol each), $CF_3CH_2OH$ (5.0 equiv, 1.0 mmol), acrylate **2a** (0.2 mmol) were added into the solution in sequence. The vial was sealed under argon and heated to 70 ℃ with stirring for 2 hours. After cooling down, the mixture was concentrated to give the crude product which was directly applied to a flash column chromatography (EtOAc/Petroleum ether mixtures) for separation.

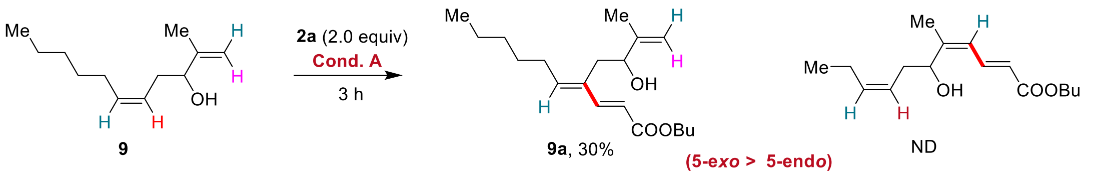


A 10 mL vial was charged with $Pd(OAc)_2$ (8.9 mg, 10 mol%, 0.04 mmol), Ac-Phe-OH (41.4 mg, 0.20 mmol, 50 mol%), $Ag_2CO_3$ (165.4 mg, 0.6mmol, 1.5 equiv), $Cs_2CO_3$ (39.0 mg, 0.12 mmol, 30 mol%), $CF_3CH_2OH$ (289 μL, 4.0 mmol, 10.0 equiv), 1,4-dioxane (0.6 mL). Then, **2a** (0.8 mmol, 4.0 equiv**)**, alcohol **9** (0.2 mmol each) were added into the solution in sequence. The vial was sealed under Ar and heated to 70 °C with stirring for 3 h. After cooling down, the mixture was concentrated in vacuo and purified by column chromatography, affording the products **9a** as a light yellow oil, 19 mg, yield = 30%. $^1$H NMR (500 MHz, $CDCl_3$): δ = 7.29 (td, *J* = 3.5 Hz, *J* = 16.0 Hz, 1H), 6.07 (td, *J* = 3.0 Hz, *J* = 7.0 Hz, 1H), 5.86 (dd, *J* = 4.0 Hz, *J* = 16.0 Hz, 1H), 5.00 (s, 1H), 4.87 (s, 1H), 4.14-4.20 (m, 3H), 2.48-2.58 (m, 2H), 2.22-2.27 (m, 2H), 1.82 (s, 3H), 1.65-1.68 (m, 2H), 1.56-1.59 (m, 2H), 1.41-1.43 (m, 2H), 1.30-1.32 (m, 4H), 0.95 (td, *J* = 4.5 Hz, *J* = 7.5 Hz, 3H), 0.89-0.90 (m, 3H). $^{13}$C NMR (125 MHz, $CDCl_3$): δ = 167.55, 148.67, 146.96, 145.56, 133.49, 115.81, 111.13, 74.25, 64.24, 33.29, 31.57, 30.81, 29.13, 28.87, 22.51, 19.20, 17.93, 13.99, 13.75. HR-MS (ESI): m/z calculatedfor $C_{19}H_{32}O_3$ $[M+H]^+$: 309.2424, found: 309.2426. FTIR (KBr, $cm^{-1}$): 3646.25, 3605.99, 3472.85, 3444.68, 2434.56, 2344.23, 1661.56, 1644.90, 1397.86.

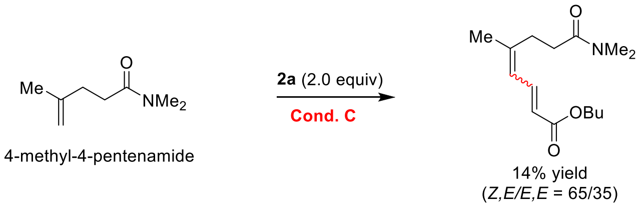


An oven-dried screw-cap vial was charged with $Pd(OAc)_2$ (10.0 mol%, 0.02 mmol), Ac-phe-OH (50.0 mol%, 0.1 mmol), $Ag_2CO_3$ (1.5 equiv, 0.3 mmol), LiOH (30 mol%, 0.06 mmol) and MeCN (1.0 mL). Then, *N*,*N*-dimethyl 4-methyl-4-pentenamide (28.2 mg, 0.2 mmol), $CF_3CH_2OH$ (5.0 equiv, 1.0 mmol), acrylate **2a** (0.2 mmol) were added into the solution in sequence. The vial was sealed under argon and heated to 70 ℃ with stirring for 16 hours. After cooling down, the mixture was concentrated to give the crude product which was directly applied to a flash column chromatography (EtOAc/Petroleum ether mixtures) for separation, affording an isomeric conjugated diene as a light yellow oil (7.5 mg, 14% yield). The configuration of the product was calculated by $^1$H NMR to be *Z,E/E,E* = 65/35 (see Supplementary Figure 212). Minor inseparable impurities were also observed from $^1$H NMR spectra. We attributed the formation of the minor impurities to the isomerization of the olefin moiety.

## Deuterium-Labelled Experiments

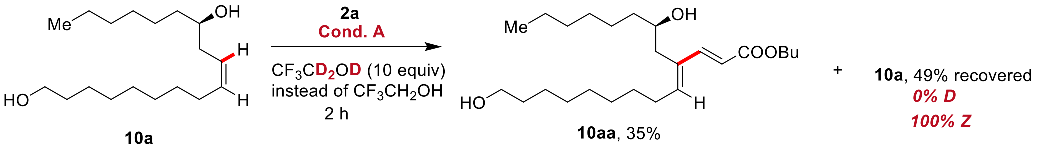


An oven-dried vial was charged with $Pd(OAc)_2$ (4.5 mg, 0.02 mmol, 10 mol%), Ac-Phe-OH (21.0 mg, 0.10 mmol, 50 mol%), $Ag_2CO_3$ (82.7 mg, 0.3 mmol, 1.5 equiv), $Cs_2CO_3$(19.5 mg, 30 mol%, 0.06 mmol), $CF_3CD_2OD$ (144.7 µL, 2.0 mmol, 10.0 equiv), in 1,4-dioxane (0.6 mL). Then, **10a** (57 mg, 0.2 mmol, 1.0 equiv) and acrylate **2a** (0.4 mmol, 2.0 equiv) were added into the solution. The vial was sealed under argon and heated to 70 °C with stirring for 2 hours. After cooling down, the mixture was directly applied to a flash column chromatography (EtOAc/petroleum ether mixtures) on silica gel to provided product **3aa** (28.9 mg, 35%) and unreacted **10a** (27.8 mg, 49%). The D% and configuration of **10aa** and **10a** were estimated by $^1$H NMR.

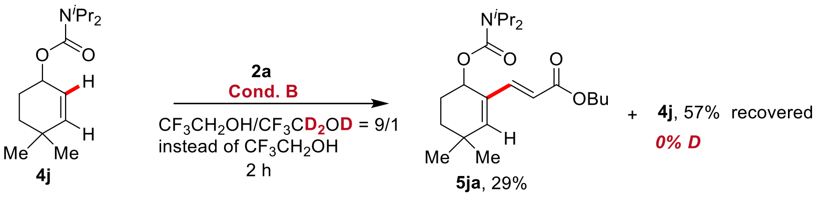


An oven-dried vial was charged with $Pd(OAc)_2$ (4.5 mg, 0.02 mmol, 10.0 mol %), Ac-Gly-OH (4.7 mg, 0.04 mmol, 20.0 mol %), $Ag_2CO_3$ (165.0 mg, 0.6 mmol, 3.0 equiv), $CF_3CD_2OD$ (1.0 mL). Then, carbamate **4j** (45.4 mg, 0.2 mmol) and acrylate **2a** (0.4 mmol, 2.0 equiv) were added into the solution. The vial was sealed under argon and heated to 80 °C with stirring for 2 hours. After cooling down, the mixture was directly applied to a flash column chromatography (EtOAc/petroleum ether mixtures) on silica gel to provide **5ja** (22.0 mg, 29%) and unreacted **4j** (28.8 mg, 57%). The D% of **5ja** and **4j** were estimated by $^1$H NMR.

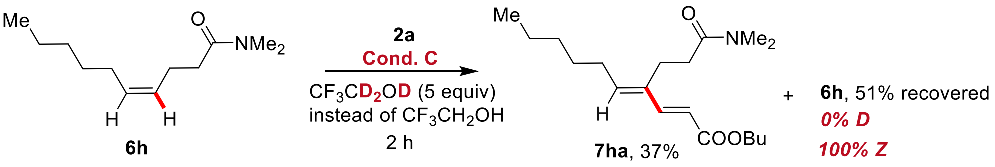


An oven-dried screw-cap vial was charged with $Pd(OAc)_2$ (10.0 mol%, 0.02 mmol), Ac-phe-OH (50.0 mol%, 0.1 mmol), $Ag_2CO_3$ (1.5 eq., 0.3 mmol), LiOH (30 mol%, 0.06 mmol), $CF_3CD_2OD$ (5.0 equiv, 1.0 mmol) and MeCN (1.0 mL). Then, amide **6h** (1.0 equiv, 0.20 mmol) and acrylate **2a** (0.4 mmol, 2.0 equiv) were added into the solution. The vial was sealed under argon and heated to 70 ℃ with stirring for 2 hours. After cooling down, the mixture was concentrated to give the crude product which was directly applied to a flash column chromatography (EtOAc/Petroleum ether mixtures) for separation to provide **7ha** (23.9 mg, 37%) and unreacted **6h** (20.1 mg, 51%). The D% of **7ha** and **6h** were estimated by $^1$H NMR.

## KIE Experiments

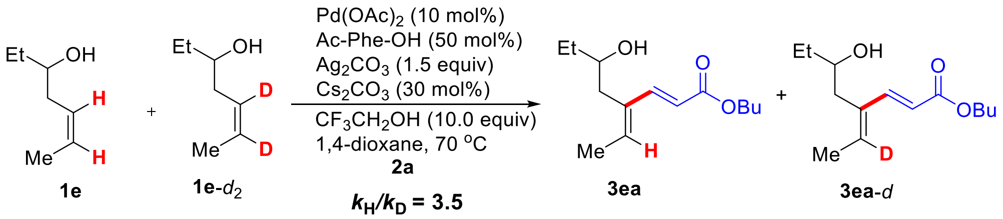


A 10 mL vial was charged with $Pd(OAc)_2$ (8.9 mg, 10 mol%, 0.04 mmol), Ac-Phe-OH (41.4 mg, 0.20 mmol, 50 mol%), $Ag_2CO_3$ (165.4 mg, 0.6mmol, 1.5 equiv), $Cs_2CO_3$ (39.0 mg, 0.12 mmol, 30 mol%), $CF_3CH_2OH$ (289 µL, 4.0 mmol, 10.0 equiv), 1,4-dioxane (0.6 mL). Then, **2a** (102.5 mg, 0.8 mmol, 4.0 equiv**)**, **1e** (22.8 mg, 0.2 mmol, 1.0 equiv) and **1e-*d*₂** (23.2 mg, 0.2 mmol, 1.0 equiv) wereadded into the solution in sequence. The vial was sealed under Ar and heated to 70 °C with stirring for 1 h. After cooling down, the mixture was concentrated in vacuo and purified by column chromatography, affording the product (19.0 mg, 20%) as yellow oil. The ratio of **3ea**/**3ea-*d*** was determined by $^1$H NMR. A kinetic isotope effect value ($k_H/k_D$) of 3.5 was observed.

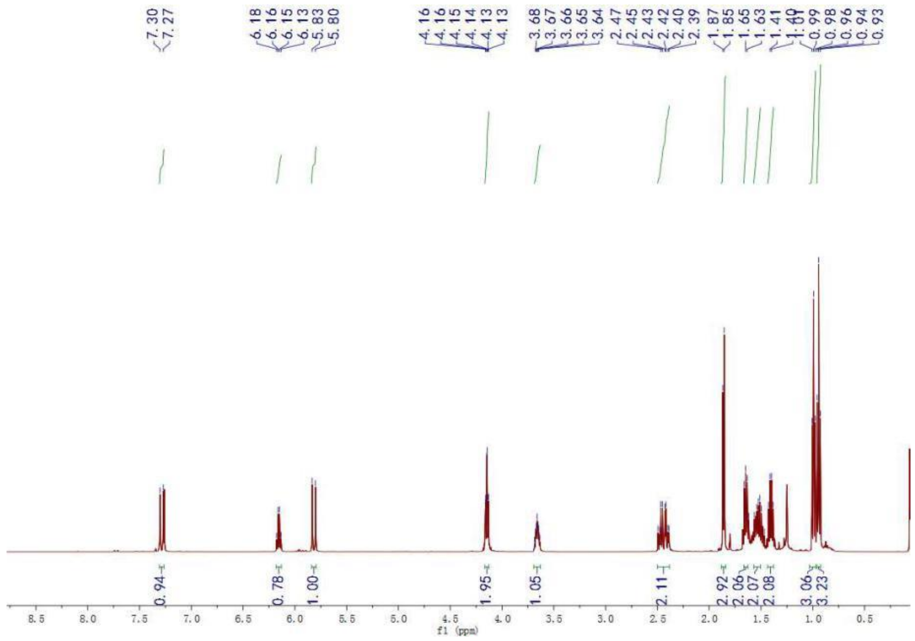

**Supplementary Figure 1.** $^1$H NMR spectrum for KIE experiment of alcohols

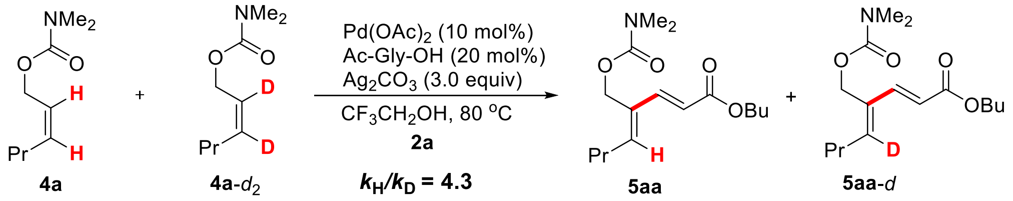


An oven-dried vial was charged with $Pd(OAc)_2$ (4.5 mg, 0.02 mmol, 10.0 mol %), Ac-Gly-OH (4.7 mg, 0.04 mmol, 20.0 mol %), $Ag_2CO_3$ (165 mg, 0.6 mmol, 3.0 eq.),$CF_3CH_2OH$ (1 mL). Then, butyl acrylate **2a** (51.3 mg,0.4 mmol, 2.0 equiv**)**, acrylamide **4a** (34.2 mg, 0.2 mmol, 1.0 equiv) and **4a-*d*₂** (34.2 mg, 0.2 mmol, 1.0 equiv) wereadded into the solution in sequence. The vial was sealed under Ar and heated to 80 ℃ with stirring for 7.0 hours. After cooling down, the mixture was concentrated in vacuo and purified by column chromatography affording the product (15 mg, 8%). The ratio of **5aa**/**5aa-*d*** was determined by $^1$H NMR (500 MHz, $CDCl_3$). A kinetic isotope effect value ($k_H/k_D$) of 4.3 was observed.

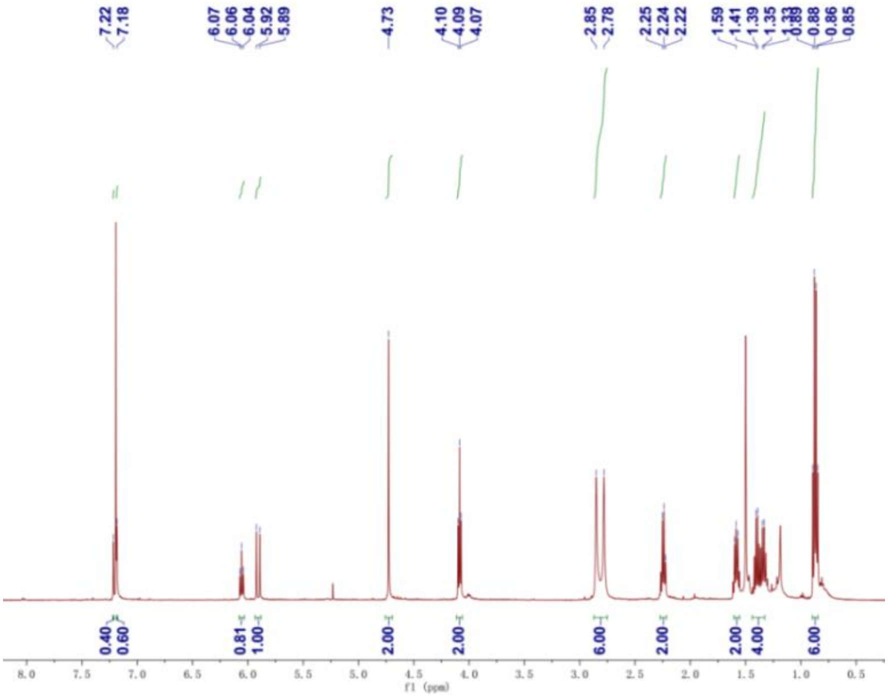


**Supplementary Figure 2.** $^1$H NMR spectrum for KIE experiment of carbamates

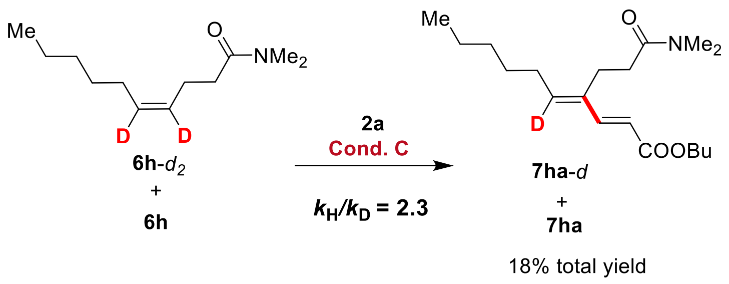


An oven-dried screw-cap vial was charged with $Pd(OAc)_2$ (10.0 mol%, 0.02 mmol), Ac-phe-OH (50.0 mol%, 0.1 mmol), $Ag_2CO_3$ (1.5 eq, 0.3 mmol), LiOH (30 mol%, 0.06 mmol) and MeCN (1.0 mL). Then, amides **6h** (1.0 eq, 0.2 mmol) and **6h-d$_2$** (1.0 eq, 0.2 mmol) and $CF_3CD_2OD$ (5.0 eq, 1.0 mmol) were added into the solution in sequence. The vial was sealed under argon and heated to 70 ℃ with stirring for 2 hour. After cooling down, the mixture was concentrated to give the crude product which was directly applied to a flash column chromatography (EtOAc/Petroleum ether mixtures) to provide **7ha** and **7ha-*d*** (12.0 mg, 18% total yield). The KIE was estimated by $^1$HNMR to be 2.3.

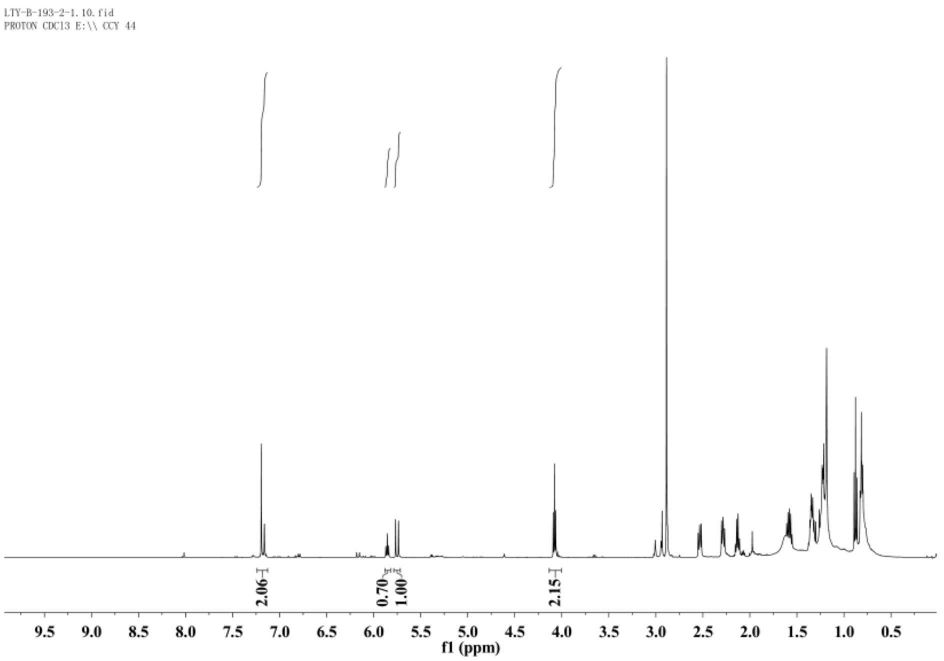


**Supplementary Figure 3.** $^1$H NMR spectrum for KIE experiment of amides

## Proposed Mechanism

The possible catalytic cycle is proposed to proceed as follows (Supplementary Figure 4). Functional-group-directed insertion of Pd(II) into the geminal C($sp^2$)-H bond results in cyclic [Pd(II)-alkene] species **I**. Then **I** coordinates with alkene **2** and undergoes 1,2-migratory insertion, followed by *β*-hydride elimination to generate the alkenylation products. Pd(0) is then reoxidized by Ag(I) and re-enters the next catalytic cycle.

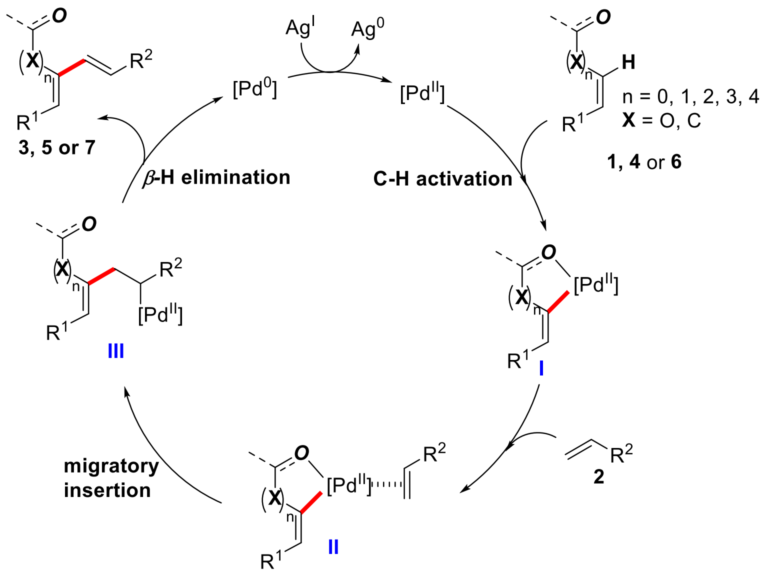


**Supplementary Figure 4.** Possible mechanism (*representative*)

## Synthetic Applications

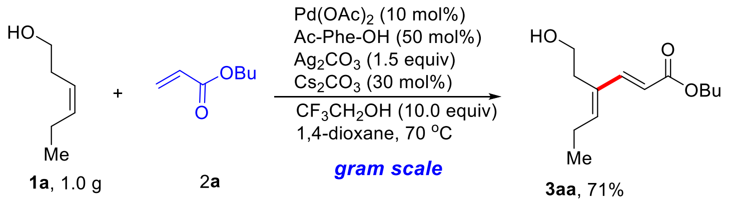


An oven-dried vial was charged with $Pd(OAc)_2$ (224.0 mg, 0.001 mol, 10 mol%), Ac-Phe-OH (1.03 g, 0.005 mol, 50 mol%), $Ag_2CO_3$ (4.14 g, 0.015 mol, 1.5 equiv), $Cs_2CO_3$ (0.98 g, 0.003 mol, 30 mol%), $CF_3CH_2OH$ (7.24mL, 0.1 mol, 10.0 equiv), 1,4-dioxane (18 mL). Then, **2a** (2.58 g, 0.02 mol, 2.0 equiv) and **1a** (1.00 g, 0.01 mol, 1.0 equiv) were added into the solution in sequence. The vial was sealed under argon and heated to 70 °C with stirring for 19 hours. After cooling down, the mixture was concentrated to give the crude product which was directly applied to a flash column chromatography for purification (EtOAc/Petroleum ether mixtures). The desired product **3aa** was obtained as a yellow oil (1.61 g, 71% yield).

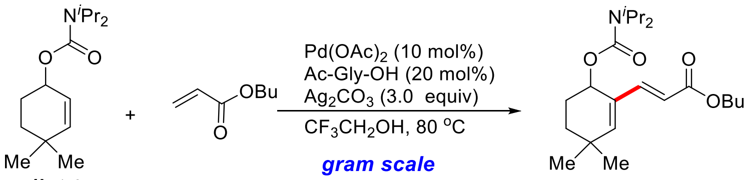


**4j**, 1.0 g    **2a**    **5ja**, 92%

An oven-dried vial was charged with $Pd(OAc)_2$ (106.4 mg, 0.47 mol, 10 mol%), Ac-Gly-OH (111.0 mg, 0.94 mol, 20 mol%), $Ag_2CO_3$ (3.9 g, 14.1 mol, 3.0 equiv), $CF_3CH_2OH$ (20 mL). Then, **2a** (1.22 g, 9.5 mmol, 2.0 equiv) and **4i** (1.20 g, 4.7mmol, 1.0 equiv) were added into the solution in sequence. The vial was sealed under argon and heated to 80 °C with stirring for 18 hours. After cooling down, the mixture was concentrated to give the crude product which was directly applied to a flash column chromatography for purification (EtOAc/petroleum ether mixtures). The desired product **5ja** was obtained as a yellow oil (1.59 g, 92% yield).

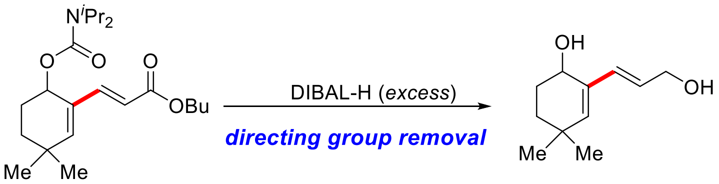


**5ja**    **11**, 73%

To a solution of (E)-3-(6-((diisopropylcarbamoyl)oxy)-3,3-dimethylcyclohex-1-en-1-yl) acrylate (**5ja**) (50.0 mg, 0.132 mmol, 1.0 equiv) inTHF (1 mL), diisobutylaluminium hydride (DIBAL-H, 1.5 M in toluene) (0.88 mL, 1.32 mmol, 10.0 equiv) was added dropwise. Thereaction mixture was stirred overnight at room temperature. After slow addition of $Na_2SO_4{\bullet}10H_2O$, the mixture was filtrated, evaporated in vacuo and purified by silica gel column chromatography, giving the alcohol **11** as a white solid (18.4 mg, 73% yield). Melting point: 90 °C. $^1$H NMR (500 MHz, $CDCl_3$): δ = 6.14 (d, $J$ = 16.0 Hz, 1H), 6.01 (dt, $J$ = 6.0 Hz, $J$ = 16.0 Hz, 1H), 5.56 (s, 1H), 4.39 (t, $J$ = 3.5 Hz, 1H), 4.20 (d, $J$ = 6.0 Hz, 2H), 1.81-1.84 (m, 2H), 1.63-1.69 (m, 1H), 1.39-1.43 (m, 1H), 1.05 (s, 3H), 0.96 (s, 3H). $^{13}$C NMR (125 MHz, $CDCl_3$):δ = 141.61, 133.01, 131.61, 125.73, 62.83, 62.41, 31.60, 30.44, 29.37, 27.41, 26.14. HR-MS (ESI): m/z calculated for $C_{11}H_{18}O_2[M+H]^+$: 183.1380, found: 183.1385. FTIR (KBr, $cm^{-1}$): 3851.14, 3626.15, 2955.86, 2921.81, 2850.53, 1384.14, 1258.87, 1082.10, 1023.69.

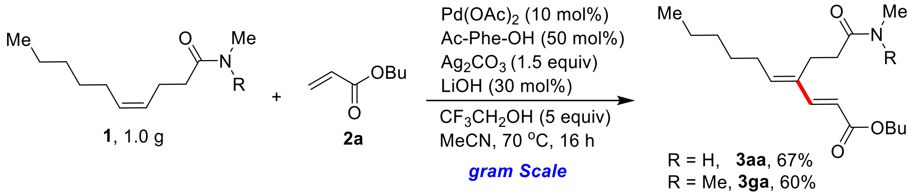


An oven-dried screw-cap vial was charged with $Pd(OAc)_2$ (10.0 mol%, 0.49 mmol), Ac-phe-OH (50.0 mol%, 2.5 mmol), $Ag_2CO_3$ (1.5 eq, 7.4 mmol), LiOH (30 mol%, 1.5 mmol) and MeCN (25.0 mL). Then, **1** (1.0 eq, 4.9 mmol), alkene **2a** (2.0 eq, 9.8 mmol), $CH_3CH_2OH$ (5.0 eq, 24.5 mmol) were added into the solution in sequence. The vial was sealed under argon and heated to 70 ℃ with stirring for 16 hours. After cooling down, the mixture was concentrated to give the crude product which was directly applied to a flash column chromatography (EtOAc/Petroleum ether mixtures) for separation.

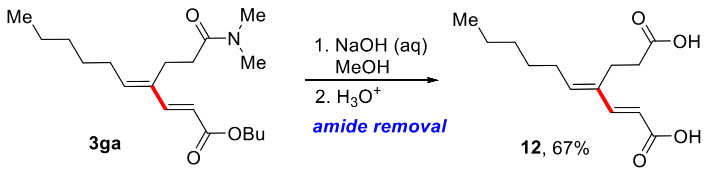


Ester **3ga** (65 mg, 0.20 mmol, 1.0 equiv) and NaOH (40 mg, 1.0 mmol, 5.0 equiv) in a mixture of methanol and water (1:1, 1.0 mL) refluxing for 10 hours at 100 ℃. Afterwards the reaction was acidified by HCl (aq., 2 M) to pH = 1 and extracted with ethyl acetate. The combined organic layers were dried over anhydrous $Na_2SO_4$, the volatiles were removed under reduced pressure, the crude product which was directly applied to a flash column chromatography (EtOAc/Petroleum ether mixtures) for separation to provide (2*E*, 4*E*)-4-hexylidenehept-2-enedioic acid (**12**) as yellow oil, 32 mg, yield = 67%. $^1$H NMR ($CDCl_3$): δ = 7.27 (d, 1H, *J* = 15.5 Hz), 5.96 (t, 1H, *J* = 7.5 Hz), 5.75 (d, 1H, *J* = 16.0 Hz), 2.57 (t, 2H, *J* = 7.5 Hz), 2.38 (t, 2H, *J* = 8.5 Hz), 2.18 (q, 2H, *J* = 7.5 Hz), 1.38-1.34 (m, 2H), 1.26-1.21 (m, 4H), 0.83 (t, 3H, *J* = 6.5 Hz). $^{13}$C NMR ($CDCl_3$): 178.12, 171.95, 149.40, 144.71, 134.02, 113.40, 31.93, 30.52, 27.82, 27.71, 21.49, 20.60, 12.97. HRMS (ESI): m/z calculated for $C_{13}H_{20}O_4$ $[M+H]^+$: 241.1444, found: 241.1449. FTIR (KBr, $cm^{-1}$): 3867.88, 3444.69, 3332.31, 3175.71, 1755.27, 1682.44, 1505.07, 1422.85, 1402.93, 984.77.

## C-H Modifications Including Natural Products and Drug Derivatives

**(3S,8S,9S,10R,13R,14S,17R)-17-((2R,5R)-5-ethyl-6-methylheptan-2-yl)-10,13-dimethyl-2,3,4, 7,8,9, 10,11,12,13,14,15,16,17-tetradecahydro-1H-cyclopenta[a]phenanthren-3-yl (2E,4E)-4- (2-hydroxy ethyl) hepta-2,4-dienoate(3ap)**. Light yellow oil, yield = 45%. Reaction time: 36 h. $^1$H NMR (500 MHz, $CDCl_3$): δ = 7.26 (d, *J* = 15.5 Hz, 1H), 6.02 (t, *J* = 7.5 Hz, 1H), 5.84 (d, *J* = 15.5 Hz, 1H), 5.38 (d, *J* = 4.5 Hz, 1H), 4.67-4.72 (m, 1H), 3.67 (t, *J* = 7.0 Hz, 2H), 2.57 (t, *J* = 7.0 Hz, 2H), 2.36-2.37 (m, 2H), 2.23-2.29 (m, 2H), 1.96-2.03 (m, 3H), 1.86-1.91 (m, 3H), 1.47-1.61 (m, 8H), 1.26-1.30 (m, 2H), 1.16-1.19 (m, 4H), 1.03-1.06 (m, 6H), 0.91-0.93 (m, 4H), 0.77-0.86 (m, 14H), 0.70 (d, *J* = 3.0 Hz, 1H), 0.68 (s, 3H). $^{13}$C NMR (125 MHz, $CDCl_3$): δ = 165.81, 147.24, 145.20, 138.71, 131.60, 121.63, 115.12, 72.84, 60.19, 55.68, 55.02, 49.03, 44.82, 41.30, 38.72, 38.62, 37.63, 36.01, 35.61, 32.93, 30.90, 30.86, 28.95, 28.14, 27.24, 26.87, 25.06, 23.28, 22.05, 21.24, 20.02, 18.81, 18.32, 18.02, 17.77, 17.24, 12.71, 10.85. HR-MS (ESI): m/z calculated for $C_{38}H_{62}O_3$ $[M+H]^+$: 567.4772, found: 567.4766. FTIR (KBr, $cm^{-1}$): 3646.04, 3585.01, 3416.53, 3132.57, 1651.51, 1492.95, 1462.10, 1400.17.

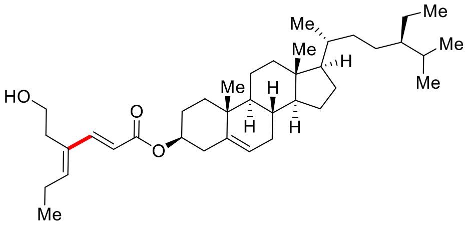


**(2E,4E)-4-(2-hydroxyethyl)-N-(((1R,4aS,10aR)-7-isopropyl-1,4a-dimethyl-1,2,3,4,4a,9,10,10a-octa hydrophenanthren-1-yl)methyl)hepta-2,4-dienamide (3aq)**. Yellow solid, yield = 62%. Melting point: 82 °C. Reaction time: 24 h. $^1$H NMR (500 MHz, $CDCl_3$): δ = 7.18 (d, *J* = 7.0 Hz, 1H), 7.16 (s, 1H), 6.99 (dd, *J* = 2.0 Hz, *J* = 8.5 Hz, 1H), 6.89 (d, *J* = 1.0 Hz, 1H), 5.93 (t, *J* = 7.5 Hz, 1H), 5.81 (d, *J* = 15.5 Hz, 1H), 5.69 (t, *J* = 6.5 Hz,

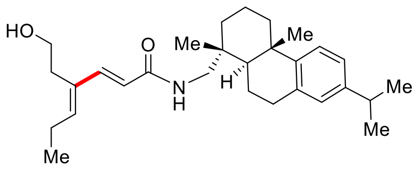

1H), 3.62 (t, $J$ = 7.5 Hz, 2H), 3.30 (dd, $J$ = 6.5 Hz, $J$ = 13.5 Hz, 1H), 3.19 (dd, $J$ = 7.0 Hz, $J$ = 14.5 Hz, 1H), 2.79-2.94 (m, 3H), 2.52 (t, $J$ = 7.0 Hz, 2H), 2.28 (d, $J$ = 12.5 Hz, 1H), 2.18-2.22 (m, 2H), 1.63-1.78 (m, 4H), 1.42-1.46 (m, 2H), 1.29-1.35 (m, 2H), 1.22 (s, 3H), 1.21 (s, 6H), 1.01 (t, $J$ = 7.5 Hz, 3H), 0.95 (s, 3H). $^{13}$C NMR (125 MHz, $CDCl_3$): δ = 165.67, 146.16, 144.61, 143.82, 143.62, 133.82, 131.34, 125.91, 123.14, 122.83, 117.19, 60.27, 48.83, 44.16, 37.28, 36.60, 36.44, 35.13, 32.40, 29.16, 24.28, 22.96, 21.11, 17.94, 17.81, 17.60, 12.78. HR-MS (ESI): m/z calculated for $C_{29}H_{43}NO_2$ $[M+H]^+$: 438.3367, found: 438.3359. FTIR (KBr, cm$^{-1}$): 3626.78, 3585.28, 3564.71, 1651.64, 1633.92, 1470.74, 1463.01, 1400.05.

**(E)-3,7-dimethylocta-2,6-dien-1-yl (2E,4E)-4- (2-hydroxyethyl) hepta-2,4-dienoate (3ar)**. Yellow oil, yield = 54%. Reaction time: 36 h. $^1$H NMR (500 MHz, $CDCl_3$): δ = 7.37-7.29 (d, $J$ = 13.0 Hz, 1H), 6.01 (t, $J$ = 7.5 Hz, 1H), 5.87 (d, $J$ = 15.9 Hz, 1H), 5.38 (t, $J$ = 6.6 Hz, 1H), 5.09 (t, $J$ = 6.7 Hz, 1H), 4.67 (d, $J$ = 7.1 Hz, 2H), 3.66 (t, $J$ = 7.0 Hz, 2H), 2.56 (t, $J$ = 7.0 Hz, 2H), 2.23-2.29 (m, 2H), 2.09-2.13 (m, 2H), 2.04-2.07 (m, 2H), 1.72 (s, 3H), 1.68 (s, 3H), 1,60 (s, 3H), 1.04 (t, $J$ = 7.5 Hz, 3H). $^{13}$C NMR (125 MHz, $CDCl_3$): δ = 166.47, 147.50, 145.34, 141.24, 131.62, 130.81, 122.76, 117.37, 114.64, 60.26, 60.15, 38.54, 28.95, 25.29, 24.67, 21.25, 16.68, 15.49, 12.69. HR-MS (ESI): m/z calculated for $C_{19}H_{30}O_3$ $[M+H]^+$: 307.2268, found: 307.2271. FTIR (KBr, cm$^{-1}$): 3626.76, 3585.23, 3133.10, 1682.42, 1633.80, 1505.12, 1462.92, 1400.22.

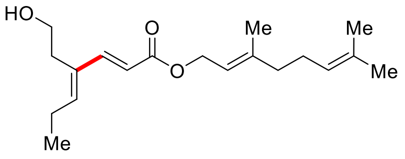


**(3S,8S,9S,10R,13R,14S,17R)-17-((2R,5R)-5-ethyl-6-methylheptan-2-yl)-10,13-dimethyl-2,3,4,7,8, 9,10,11,12,13,14,15,16,17-tetradecahydro-1H-cyclopenta[a]phenanthren-3-yl (E)-3-(6-((diiso propylcarbamoyl)oxy)-3,3-dimethylcyclohex-1-en-1-yl) acrylate (5jp)**. Yellow oil, yield = 74%. Reaction time: 24 h. $^1$H NMR (500 MHz, $CDCl_3$): δ = 7.17 (d, $J$ = 16.0 Hz, 1H), 6.04 (s, 1H), 5.85 (s, 1H), 5.49 (t, $J$ = 3.0 Hz, 1H), 5.37 (s, 1H), 4.63-4.69 (m, 1H), 4.16 (brs, 1H), 3.60 (brs, 1H), 2.30-2.42 (m, 2H), 1.81-2.02 (m, 8H), 1.43-1.69 (m, 11H), 1.26-1.33 (m, 5H), 1.10-1.20 (m, 13H), 1.10 (s, 6H), 1.02-1.03 (m, 9H), 0.92 (t, $J$ = 5.5 Hz, 3H), 0.77-0.86 (m, 9H), 0.68-0.70 (m, 3H). $^{13}$C NMR (125 MHz, $CDCl_3$): δ = 165.80, 154.27, 150.24, 144.20, 130.45, 121.52, 121.48, 116.65, 72.59, 64.15, 55.70, 55.04, 49.04, 44.84, 41.31, 38.73, 37.17, 36.00, 35.60, 35.59, 35.15, 32.94, 32.06, 30.91, 30.87, 28.11, 28.15, 27.24, 26.82, 25.80, 25.08, 24.90, 23.28, 22.06, 20.07, 20.02, 19.20, 18.80, 18.30, 18.03, 17.77, 17.69, 17.25, 10.97, 10.85. HR-MS (ESI): m/z calculated for $C_{47}H_{77}NO_4$ $[M+H]^+$: 720.5925, found: 720.5913. FTIR (KBr, cm$^{-1}$): 3626.74, 3592.68, 3132.81, 2488.73, 1694.10, 1682.49, 1462.99, 1400.15.

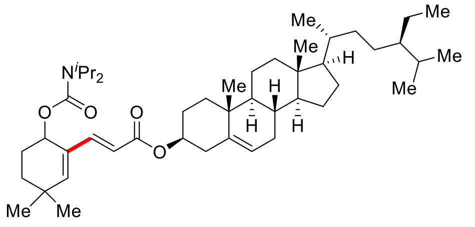


**Butyl (2E,4E)-13-hydroxy-4-(2-hydroxy octyl) trideca-2,4-dienoate (10aa)**. White solid, yield = 82%. Melting point: 44 °C. Reaction time: 16 h. $^1$H NMR (400 MHz, $CDCl_3$): δ = 7.28 (d, $J$ = 16.4 Hz, 1H), 6.05 (t, $J$ = 7.6 Hz, 1H), 5.82 (d, $J$ = 16.0 Hz, 1H), 4.15 (t, $J$ = 6.8 Hz, 2H), 3.69-3.74 (m, 1H), 3.64 (t, $J$ = 6.8 Hz, 2H), 2.36-2.48 (m, 2H), 2.24 (q, $J$ = 7.2 Hz, 2H), 1.63-1.69 (m, 2H), 1.56-1.58 (m, 2H), 1.48-1.50 (m, 4H), 1.40 (q, $J$ = 7.2 Hz, 4H), 1.31-1.33 (m, 14H), 0.95 (t, $J$ = 7.6 Hz, 3H), 0.88 (t, $J$ = 5.2 Hz, 3H). $^{13}$C NMR (125 MHz, $CDCl_3$): δ = 166.54, 147.74, 144.31, 132.70, 114.88, 69.50, 63.22, 62.00, 36.33, 33.91, 31.73, 30.81, 29.78, 28.38, 28.32, 28.27, 28.17, 28.15, 24.80, 24.67, 21.60, 18.18, 13.07, 12.73. HR-MS (ESI): m/z calculated for

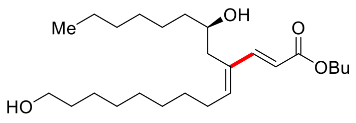

$C_{25}H_{46}O_4$ $[M+Na]^+$: 411.3469, found: 411.3468. FTIR (KBr, $cm^{-1}$): 3564.43, 3416.76, 2970.62, 2925.50, 1633.49, 1621.48, 1462,81, 1393.77, 1048.78.

**1-Butyl-13-methyl-4-(*R*)-2-hydroxyoctyl)-(2E,4E)-2,4-tridecadiene-1,13-dioate (10ba)**. White solid,

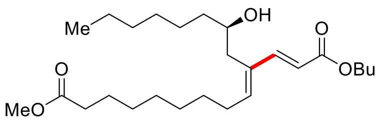


yield = 67%. Melting point: 47 °C. Reaction time: 16 h. $^1H$ NMR (500 MHz, $CDCl_3$): δ = 7.20 (d, *J* = 16.0 Hz, 1H), 5.96 (t, *J* = 7.5 Hz, 1H), 5.75 (d, *J* = 16.0 Hz, 1H), 4.07 (td, *J* = 1.0 Hz, *J* = 6.5 Hz, 2H), 3.62-3.64 (m, 1H), 3.59 (s, 3H), 2.30-2.40 (m, 2H), 2.23 (t, *J* = 6.5 Hz, 2H), 2.17 (q, *J* = 7.5 Hz, 2H), 1.53-1.59 (m, 4H), 1.32-1.36 (m, 4H), 1.18-1.24 (m, 16H), 0.87 (t, *J* = 7.5 Hz, 3H), 0.82 (t, *J* = 6.5 Hz, 3H). $^{13}$CNMR (125 MHz, $CDCl_3$): δ = 173.27, 166.57, 147.80, 144.15, 132.83, 114.86, 69.48, 63.21, 50.45, 36.33, 33.95, 33.03, 30.82, 29.78, 28.33, 28.15, 28.10, 29.07, 28.00, 24.82, 23.86, 21.60, 18.18, 13.07, 12.73. HR-MS (ESI): m/z calculated for $C_{26}H_{46}O_5\{[M-H_2O]+H\}^+$: 421.3318, found: 421.3326. FTIR (KBr, $cm^{-1}$): 3583.23, 3456.28, 1842.15, 1773.28, 1428.16, 1422,86, 1360.65, 1337.42.

**10-(E)-2-(Diethylphosphinyl) ethenyl methyl ricinoleate (10bl)**. Yellow oil, yield = 61%. Reaction

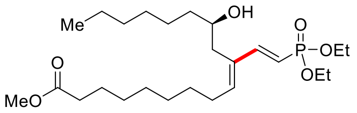


time: 16 h. $^1H$ NMR (500 MHz, $CDCl_3$): δ = 7.07 (dd, *J* = 17.5 Hz, *J* = 22.5 Hz, 1H), 5.99 (t, *J* = 7.5 Hz, 1H), 5.60 (t, *J* = 18.0 Hz , 1H), 4.05-4.09 (m, 4H), 3.66-3.68 (m, 4H), 2.41 (dq, *J* = 8.5 Hz, *J* = 14.0 Hz, 2H), 2.29 (t, *J* = 7.5 Hz, 2H), 2.22 (q, *J* = 7.5 Hz, 2H), 1.61 (t, *J* = 7.0 Hz, 3H), 1.25-1.34 (m, 23H), 0.88 (t, *J* = 7.0 Hz, 3H). $^{13}C$ NMR (125 MHz, $CDCl_3$): δ = 173.27, 151.77 (d, $J_{C-P}$ = 6.3 Hz), 143.63, 133.07 (d, $J_{C-P}$ = 22.5 Hz), 110.04 (d, $J_{C-P}$ = 190.0 Hz), 69.52, 60.68, 60.64, 50.46, 36.36, 33.61, 33.03, 30.79, 28.30, 28.13, 28.05, 28.00, 24.81, 23.86, 21.59, 15.41, 15.36, 13.05. HR-MS (ESI): m/z calculated for $C_{25}H_{47}O_6P[M+H]^+$: 475.3183, found: 475.3203. FTIR (KBr, $cm^{-1}$): 3564.02, 3383.12, 1738.33, 1645.99, 1625.66, 1534.89, 1621.55, 1416.88, 1242.32, 1027.57, 963.56.

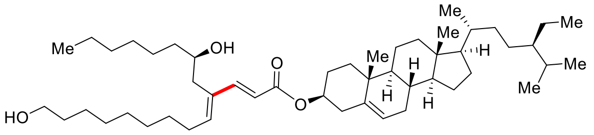


**(3S,8S,9S,10R,13R,14S,17R)-17-((2R,5R)-5-ethyl-6-methylheptan-2-yl)-10,13-dimethyl-2,3,4,7,8,9,10,11,12,13,14,15,16,17-tetradecahydro-1H-cyclopenta[a]phenanthren-3-yl (2E,4E)-13-hydroxy -4-((R)-2-hydroxyoctyl)trideca-2,4-dienoate (10ap)**. Yellow oil, yield = 59%. Reaction time: 24 h. $^1H$ NMR (500 MHz, $CDCl_3$): δ = 7.27 (d, *J* = 15.5 Hz, 1H), 6.04 (t, *J* = 7.5 Hz, 1H), 5.80 (d, *J* = 16.0 Hz, 1H), 5.38 (d, *J* = 4.5 Hz, 1H), 4.06-4.72 (m, 1H), 3.70-3.73 (m, 1H), 3.63 (t, *J* = 6.5 Hz, 2H), 2.36-2.47 (m, 4H), 2.24 (q, *J* = 7.5 Hz, 2H), 1.91-2.03 (m, 2H), 1.84-1.89 (m, 3H), 1.42-1.66 (m, 18H), 1.26-1.31 (m, 18H), 1.15-1.21 (m, 3H), 1.01-1.03 (m, 6H), 0.77-0.93 (m, 17H), 0.68-0.70 (m, 3H). $^{13}C$ NMR (125 MHz, $CDCl_3$): δ = 165.87, 147.66, 144.23, 138.71, 132.74, 121.62, 115.25, 72.87, 69.49, 61.95, 55.68, 55.02, 50.22, 49.03, 44.82, 41.30, 38.72, 37.22, 36.31, 36.02, 35.60, 35.14, 33.94, 31.72, 30.86, 30.82, 28.38, 28.34, 28.28, 28.26, 28.16, 27.24, 26.87, 25.06, 24.81, 24.67, 23.28, 22.05, 21.61, 20.02, 19.20, 18.32, 18.03, 17.77, 17.25, 14.36, 13.09, 11.25, 10.97, 10.85. HR-MS (ESI): m/z calculated for $C_{50}H_{86}O_4$ $[M+H]^+$: 751.6599, found: 751.6589. FTIR (KBr, $cm^{-1}$): 3444.39, 3417.55, 2455,66, 1866.20, 1630.41, 1524.97, 1336.90.

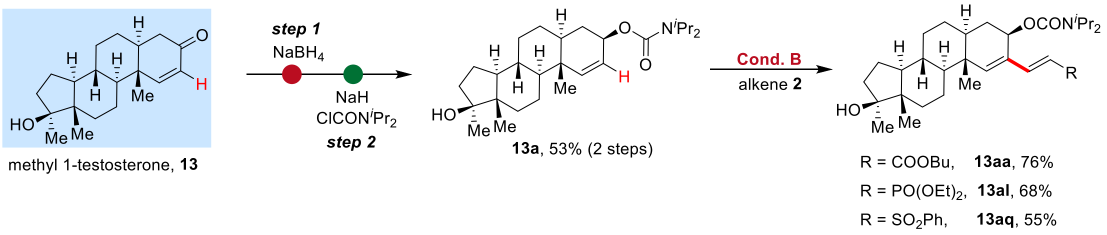


**Step 1**: A magnetically stirred solution of the relevant enone **13** (1.81 g, 6.0 mmol, 1 equiv) and $CeCl_3·7H_2O$ (2.22 g, 6.0 mmol, 1 equiv) in MeOH (30 mL) was cooled to 0°C and then treated with sodium borohydride (0.26 g, 7.0 mmol) in portions (CAUTION: EVOLUTION OF HYDROGEN GAS). The resulting mixture was stirred at 0 °C for 1 h before being quenched with water (10 mL) and then concentrated under reduced pressure. The residue so-formed was extracted with ethyl acetate (3 × 20 mL), and the combined organic phases were dried ($Na_2SO_4$), filtered, and concentrated under reduced pressure to give the crude allylic alcohol, which was applied to a flash column chromatography for purification (EtOAc/petroleum ether mixtures). (3β,5α,17β)-17-Methyl-androst-1-ene-3,17-diol was obtained as a white solid, 1.55 g, yield = 70%. $^1$H NMR (500 MHz, $CDCl_3$): δ = 5.85 (dd, *J* = 1.5 Hz, *J* = 10.0 Hz, 1H), 5.42 (dt, *J* = 1.5 Hz , *J* = 10.0 Hz , 1H), 4.23 (dd, *J* = 6.5 Hz, *J* = 9.0 Hz, 1H), 1.61-1.75 (m, 6H), 1.39-1.47 (m, 4H), 1.27-1.33 (m, 4H), 1.18-1.20 (m, 4H), 1.14 (s, 3H), 0.86 (s, 3H), 0.79 (s, 3H).

**Step 2**: A solution of the alcohol (152.2 mg, 0.5 mmol, 1.0 equiv) in dry THF (2.0 mL) was added dropwise to a suspension of sodium hydride (60% in mineral oil) (40.0 mg, 1.0 mmol, 2.0 equiv) in THF (0.5 mL) at 0 ℃. After stirring at room temperature for 1 h, *N*,*N*-diisopropylcarbamoyl chloride (165.0 mg, 1.0 mmol, 2.0 equiv) was added as a solid over a period of 15 min. The mixture was refluxed for 3 h and cooled down to room temperature before it was poured into a 2N HCl solution (2.0 mL). After separation of the organic phase, the aqueous layer was extracted three times with ethyl acetate (2 mL) and the combined organic phases were washed with a sat. aq. $NaHCO_3$ solution (2.0 mL). After drying over $Na_2SO_4$ and evaporation of the solvent under vacuum, the crude product was purified by column chromatography. Carbamate **(13a)** was obtained as a white solid, 162.0 mg, yield = 75%. $^1$H NMR (500 MHz, $CDCl_3$): δ = 5.98 (d, *J* = 10.5 Hz, 1H), 5.50 (d, *J* = 10.0 Hz, 1H), 5.29-5.33 (m, 1H), 4.13 (brs, 1H), 3.70 (brs, 1H), 1.61-1.87 (m, 12H), 1.35-1.50 (m, 3H), 1.31 (d, *J* = 6.5 Hz, 3H), 1.24 (d, *J* = 7.5 Hz, 6H), 1.20 (d, *J* = 8.0 Hz, 9H). $^{13}$C NMR (125 MHz, $CDCl_3$): δ = 155.66, 138.73, 125.71, 81.65, 71.28, 51.25, 50.73, 47.47, 46.69, 45.68, 43.44, 38.96, 38.05, 36.45, 32.00, 31.62, 31.53, 27.96, 25.85, 23.19, 21.35, 20.87, 20.25, 15.48, 14.13.

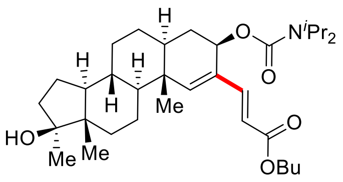


**Butyl (E)-3-((5S,8R,9S,10S,13S,14S,17S)-3-((diisopropyl carbamoyl) oxy)-17-hydroxy-10,13,17-trimethyl -4,5,6,7,8,9,10, 11,12,13,14,15,16,17-tetradecahydro-3H-cyclopenta[a] phenanthren-2-yl) acrylate (13aa)**. Yellow solid, yield = 76%. Melting point: 40 °C. Reaction time: 16 h. $^1$H NMR (500 MHz, $CDCl_3$): δ = 7.16 (d, *J* = 16.5 Hz, 1H), 6.51 (s, 1H), 5.76 (d, *J* = 16.0 Hz, 1H), 5.61 (t, *J* = 8.0 Hz, 1H), 4.06-4.17 (m, 3H), 3.68 (brs, 1H), 2.11-2.16 (m, 1H), 1.78-1.93 (m, 2H), 1.68-1.74 (m, 2H), 1.54-1.64 (m, 5H), 1.35-1.41 (m, 4H), 1.12-1.26 (m, 23H), 1.01 (s, 3H), 0.92 (t, *J* = 7.0 Hz, 3H), 0.88 (s, 3H). $^{13}$C NMR (125 MHz, $CDCl_3$): δ = 166.49, 154.18, 148.37, 144.25, 132.02, 116.74, 80.54, 67.89, 62.97, 49.77, 49.70, 45.47, 44.65, 44.21, 41.73, 37.90, 37.69, 35.27, 31.61, 30.58, 30.28, 29.73, 26.44, 24.86, 22.17, 20.67, 19.75, 19.53, 18.11, 13.43, 13.15, 12.67. HR-MS (ESI): m/z calculated for $C_{34}H_{55}NO_5$

[M+H]$^+$: 558.4153, found: 558.4150. FTIR (KBr, cm$^{-1}$): 2955.82, 2923.55, 2853.05, 1694.23, 1682.45, 1463.03, 1383.91, 1082.25.

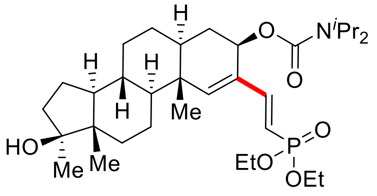


**(5S,8R,9S,10S,13S,14S,17S)-2-((E)-2-(diethoxyphosphoryl)vinyl)-17-hydroxy-10,13,17-trimethyl-4,5,6,7,8,9,10,11,12,13,14,15,16,17-tetradecahydro-3H-cyclopenta[a]phenanthren-3-yl diisopropyl carbamate (13al)**. Yellow oil, yield = 68%. Reaction time: 16 h. $^1$H NMR (500 MHz, CDCl$_3$): δ = 6.98 (dd, $J$ = 17.5 Hz, $J$ = 23.5 Hz, 1H), 6.50 (s, 1H), 5.65 (t, $J$ = 8.5 Hz, 1H), 5.56 (t, $J$ = 18.0 Hz, 1H), 4.14 (brs, 1H), 4.00-4.05 (m, 4H), 3.63 (brs, 1H), 2.11-2.16 (m, 1H), 1.97 (s, 1H), 1.78-1.84 (m, 2H), 1.65-1.73 (m, 2H), 1.54-1.65 (m, 4H), 1.38-1.46 (m, 4H), 1.26-1.31 (m, 9H), 1.14-1.21 (m, 16H), 1.00 (s, 3H), 0.88 (s, 3H). $^{13}$C NMR (125 MHz, CDCl$_3$): δ = 154.28, 148.68 (d, $J_{C-P}$ = 6.5 Hz), 148.37, 132.26 (d, $J_{C-P}$ = 23.3 Hz), 111.82 (d, $J_{C-P}$ = 188.9 Hz), 80.48, 67.40, 60.54, 60.49, 60.36, 60.31, 49.74, 49.72, 45.63, 44.66, 44.02, 41.77, 37.87, 37.56, 35.26, 31.57, 30.58, 30.28, 26.44, 24.88, 22.18, 20.85, 20.68, 19.72, 15.36, 13.31, 13.32, 13.31, 13.16. HR-MS (ESI): m/z calculated for $C_{33}H_{56}NO_6P$ [M+H]$^+$: 594.3918, found: 594.3926. FTIR (KBr, cm$^{-1}$): 2955.19, 2921.93, 2852.25, 1682.36, 1651.55, 1383.97, 1027.41.

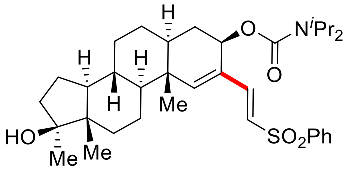


**(5S,8R,9S,10S,13S,14S,17S)-17-hydroxy-10,13,17-trimethyl-2-((E)-2-(phenylsulfonyl)vinyl)-4,5,6,7,8,9,10,11,12,13,14,15,16,17-tetradecahydro-3H-cyclopenta[a]phenanthren-3-yl diisopropyl carbamate (13aq)**. Light yellow oil, yield = 55%. Reaction time: 16 h. $^1$H NMR (500 MHz, CDCl$_3$): δ = 7.84-7.86 (m, 2H), 7.57 (m, 1H), 7.49 (t, $J$ = 8.0 Hz, 2H), 7.17 (d, $J$ = 15.5 Hz, 1H), 6.63 (s, 1H), 6.22 (d, $J$ = 15.5 Hz, 1H), 5.56 (t, $J$ = 8.5 Hz, 1H), 4.05 (brs, 1H), 3.49 (brs, 1H), 2.04-2.11 (m, 1H), 1.79-1.84 (m, 2H), 1.68-1.74 (m, 2H), 1.55-1.63 (m, 3H), 1.37-1.49 (m, 5H), 1.28-1.33 (m, 2H), 1.21 (s, 3H), 1.03-1.12 (m, 9H), 1.01 (s, 3H), 0.86-0.90 (m, 9H). $^{13}$C NMR (125 MHz, CDCl$_3$): δ = 153.97, 151.53, 141.87, 140.10, 132.01, 130.70, 128.15, 126.60, 125.48, 80.50, 67.27, 49.67, 49.56, 45.67, 44.64, 44.01, 41.61, 37.99, 37.87, 35.22, 31.35, 30.54, 30.19, 26.35, 24.88, 22.16, 19.73, 19.54, 19.38, 13.28, 13.16. HR-MS (ESI): m/z calculated for $C_{35}H_{51}NO_5S$ [M+H]$^+$: 598.3561, found: 598.3565. FTIR (KBr, cm$^{-1}$): 2956.07, 2922.27, 2852.61, 1673.98, 1300.74, 1191.30, 1147.04, 1083.32, 1026.12, 848.38.

## Reaction of Inseparable *Z/E* Isomers

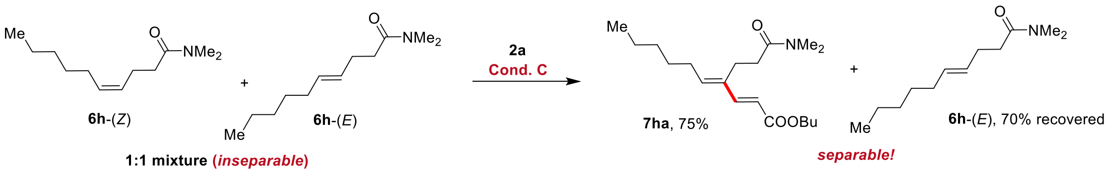


An oven-dried screw-cap vial was charged with Pd(OAc)$_2$ (10.0 mol%, 0.02 mmol), Ac-phe-OH (50.0 mol%, 0.1 mmol), $Ag_2CO_3$ (1.5 equiv, 0.3 mmol), LiOH (30 mol%, 0.06 mmol) and MeCN (1.0 mL). Then, an inseparable *Z/E* mixture of amide **6a** (0.4 mmol, *Z/E* ratio = 1:1) were added into the solution in sequence. The vial was sealed under argon and heated to 70 ℃ with stirring for 16 hour. After cooling down, the mixture was concentrated to give the crude product which was directly applied to a flash column chromatography (EtOAc/Petroleum ether mixtures) for separation, providing **7ha** (48.5 mg, 75% recovered) and pure *E*-amide (27.6 mg, 66% recovered).

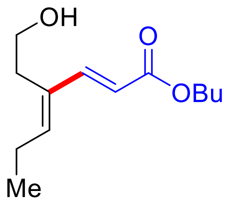


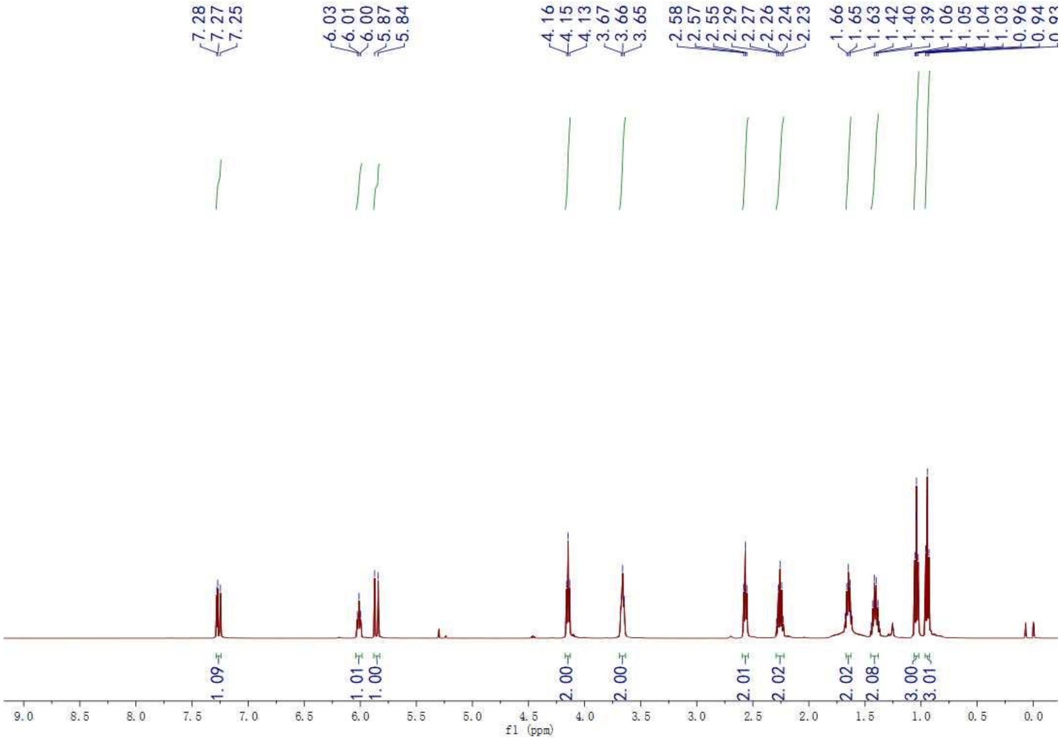


**Supplementary Figure 5.** $^{1}$H NMR spectrum for **3aa** in $CDCl_3$

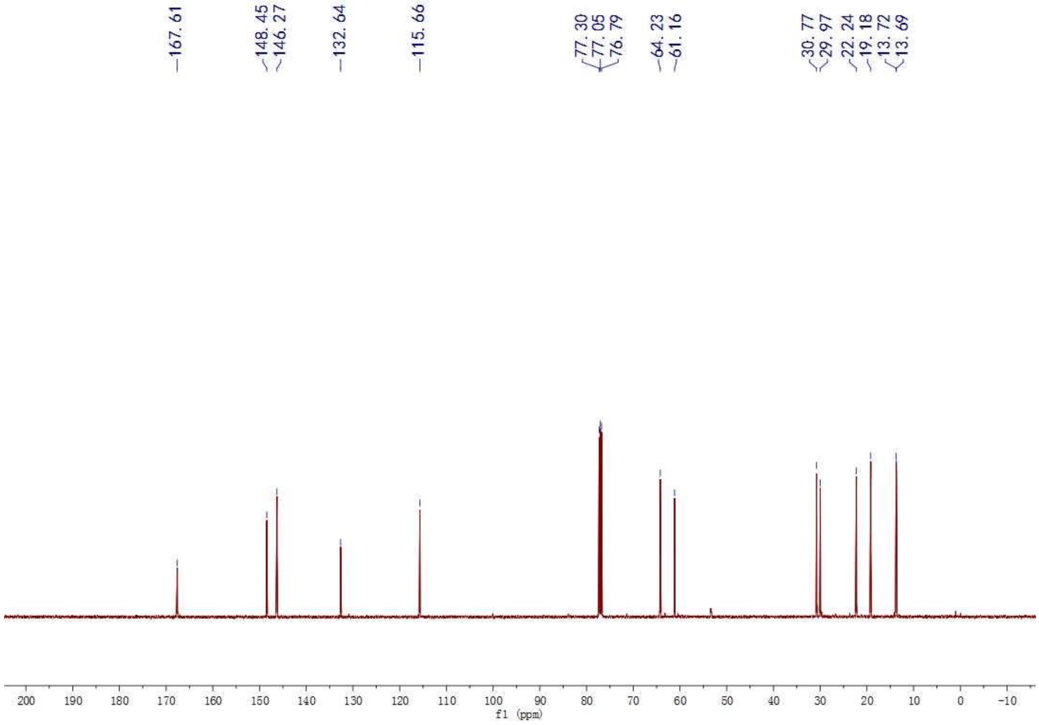


**Supplementary Figure 6.** $^{13}$C NMR spectrum for **3aa** in $CDCl_3$

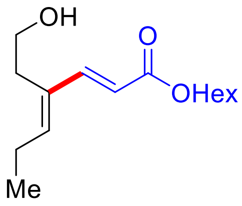


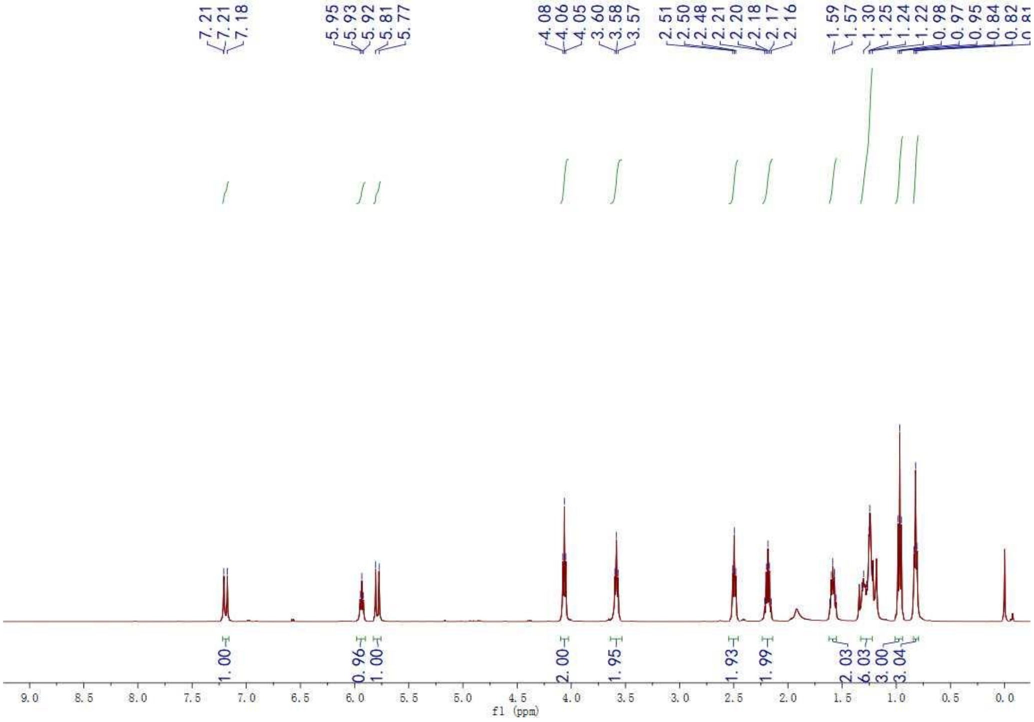


**Supplementary Figure 7.** $^{1}$H NMR spectrum for **3ab** in $CDCl_3$

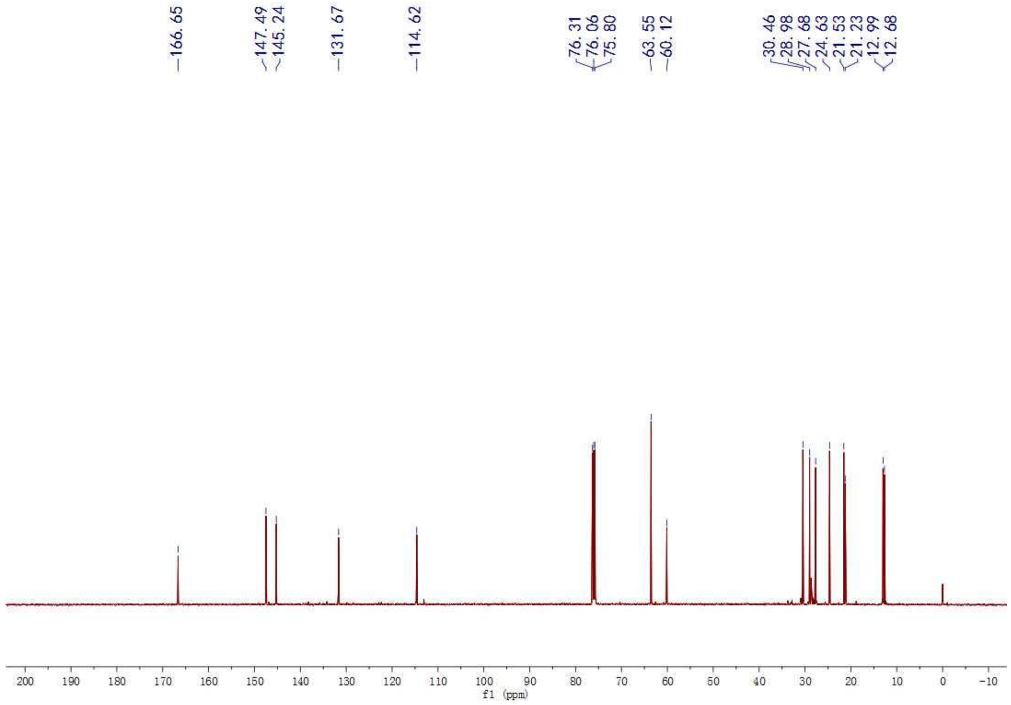


**Supplementary Figure 8.** $^{13}$C NMR spectrum for **3ab** in $CDCl_3$

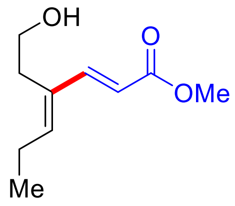


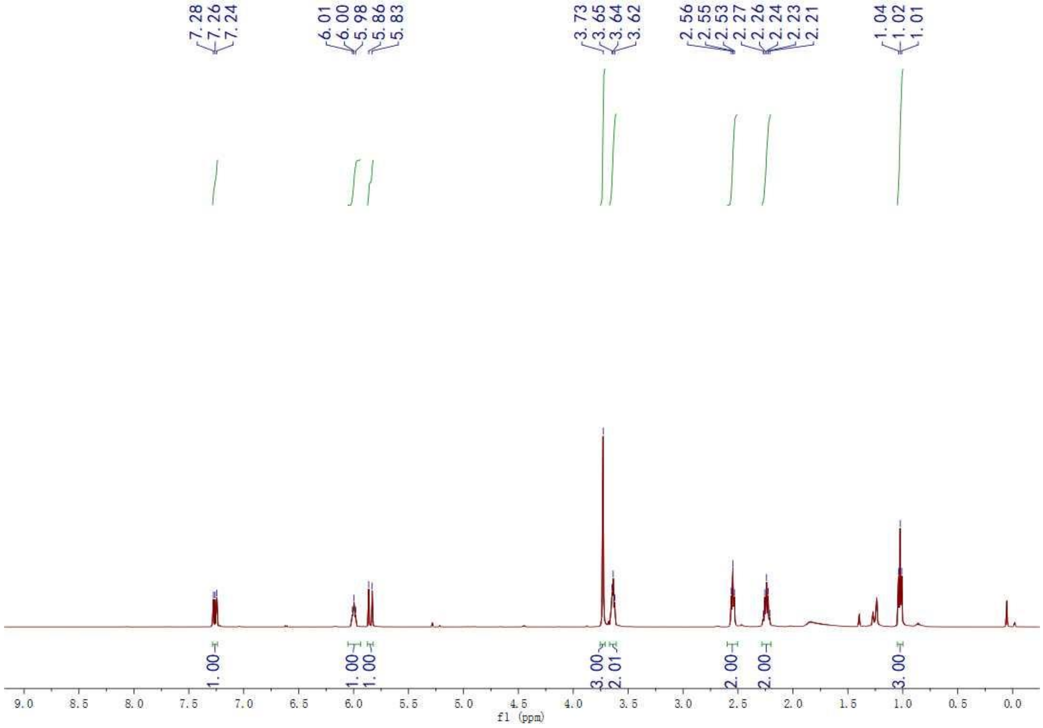


**Supplementary Figure 9.** $^{1}$H NMR spectrum for **3ac** in $CDCl_3$

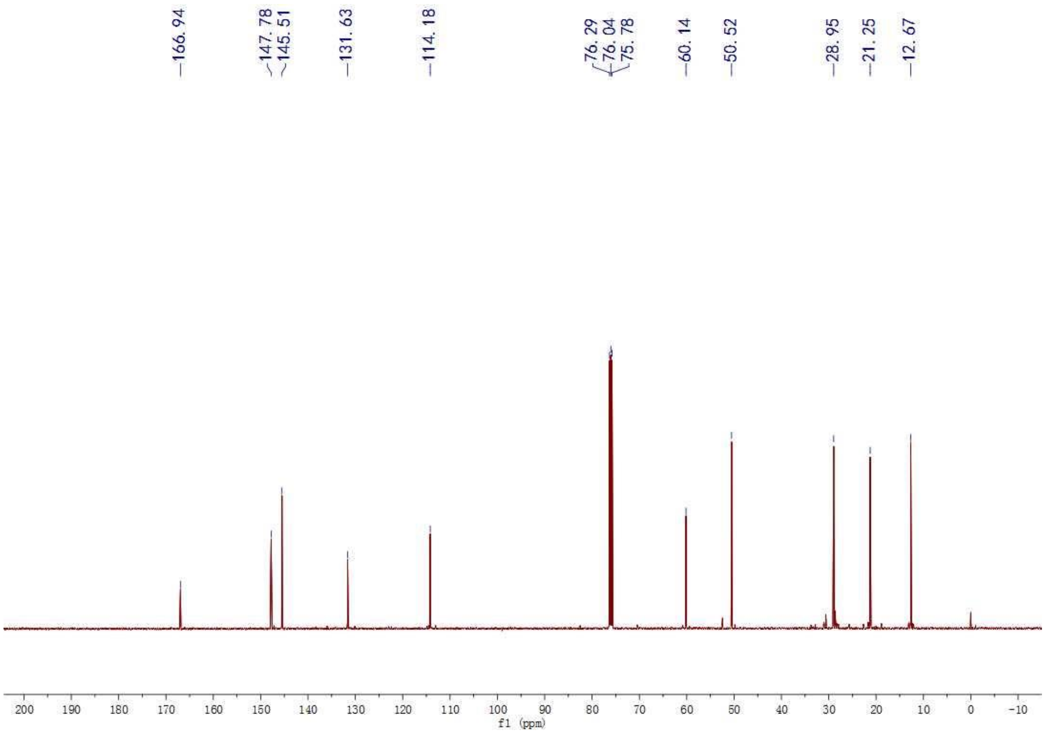


**Supplementary Figure 10.** $^{13}$C NMR spectrum for **3ac** in $CDCl_3$

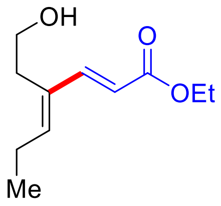


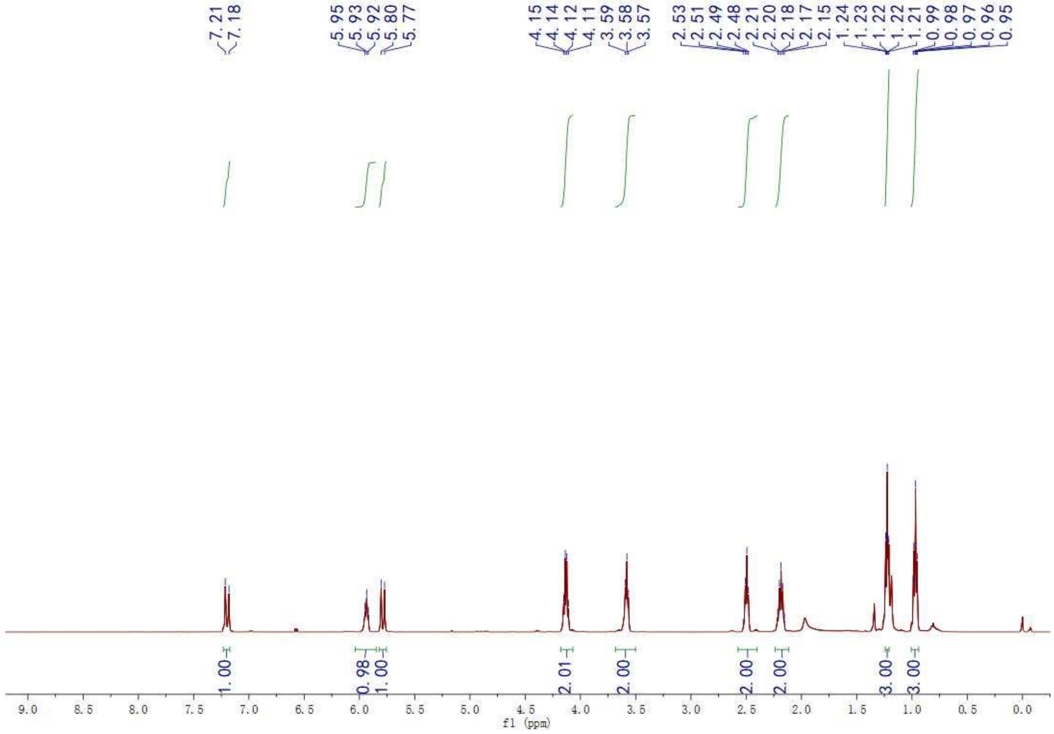

**Supplementary Figure 11.** $^{1}H$ NMR spectrum for **3ad** in $CDCl_3$

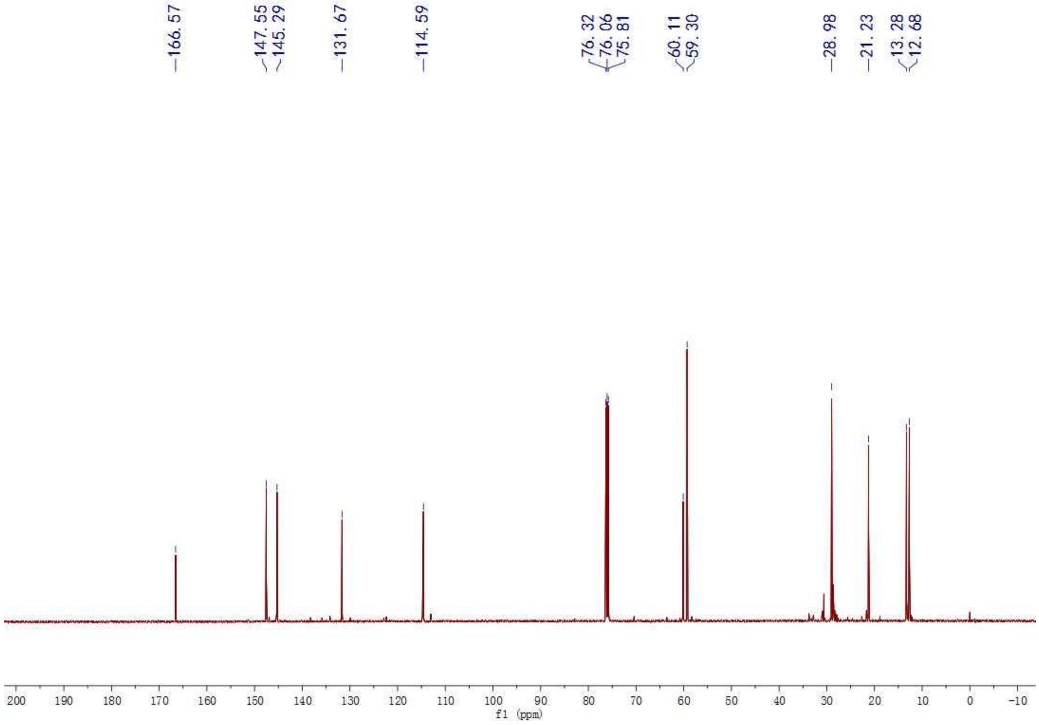

**Supplementary Figure 12.** $^{13}C$ NMR spectrum for **3ad** in $CDCl_3$

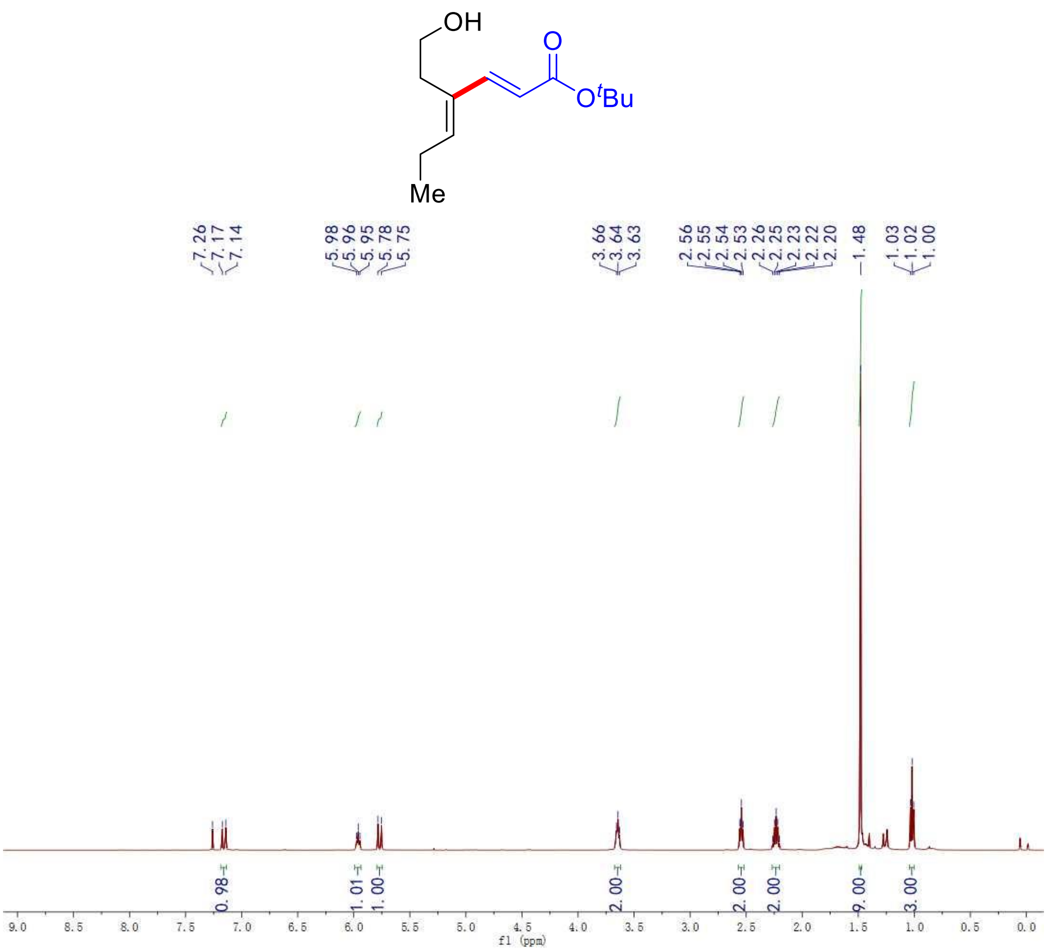


**Supplementary Figure 13.** $^{1}$H NMR spectrum for **3ae** in $CDCl_3$

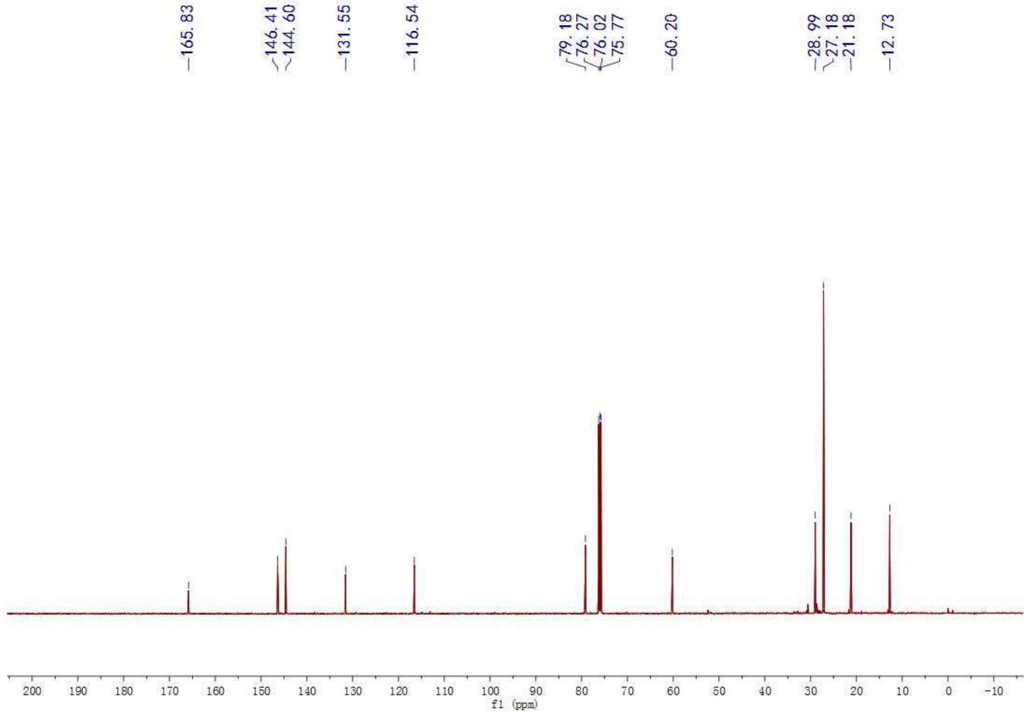


**Supplementary Figure 14.** $^{13}$C NMR spectrum for **3ae** in $CDCl_3$

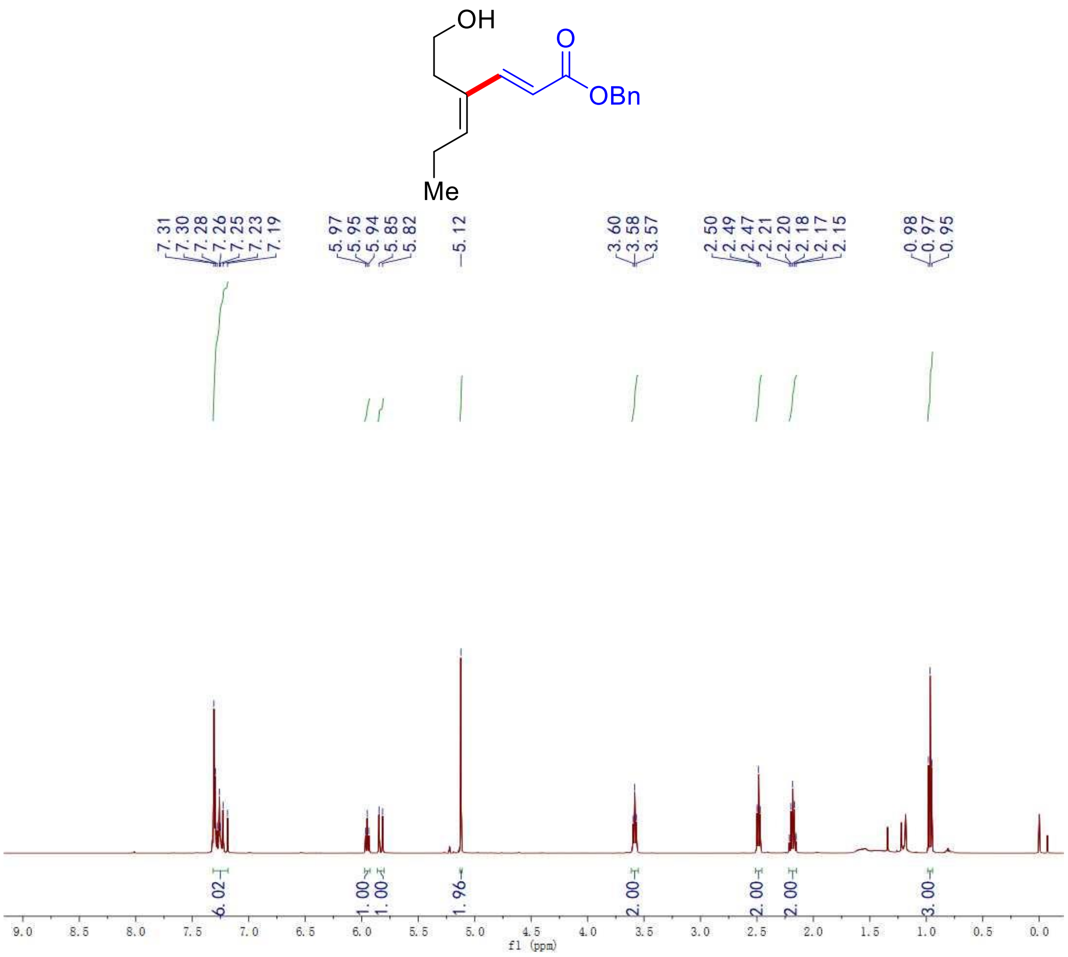


**Supplementary Figure 15.** $^{1}$H NMR spectrum for **3af** in $CDCl_3$

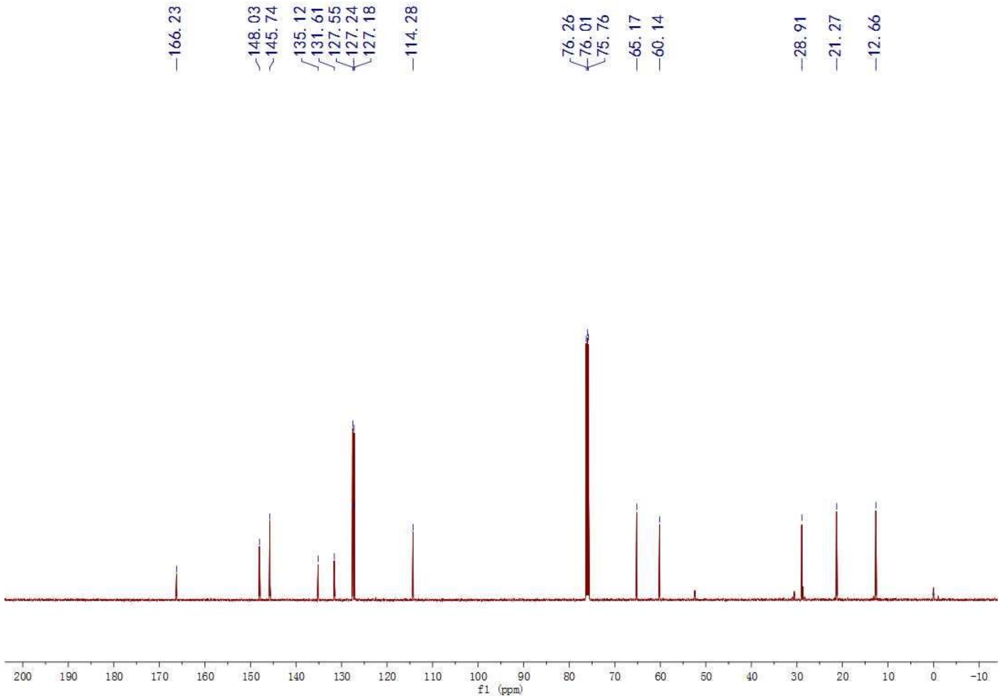


**Supplementary Figure 16.** $^{13}$C NMR spectrum for **3af** in $CDCl_3$

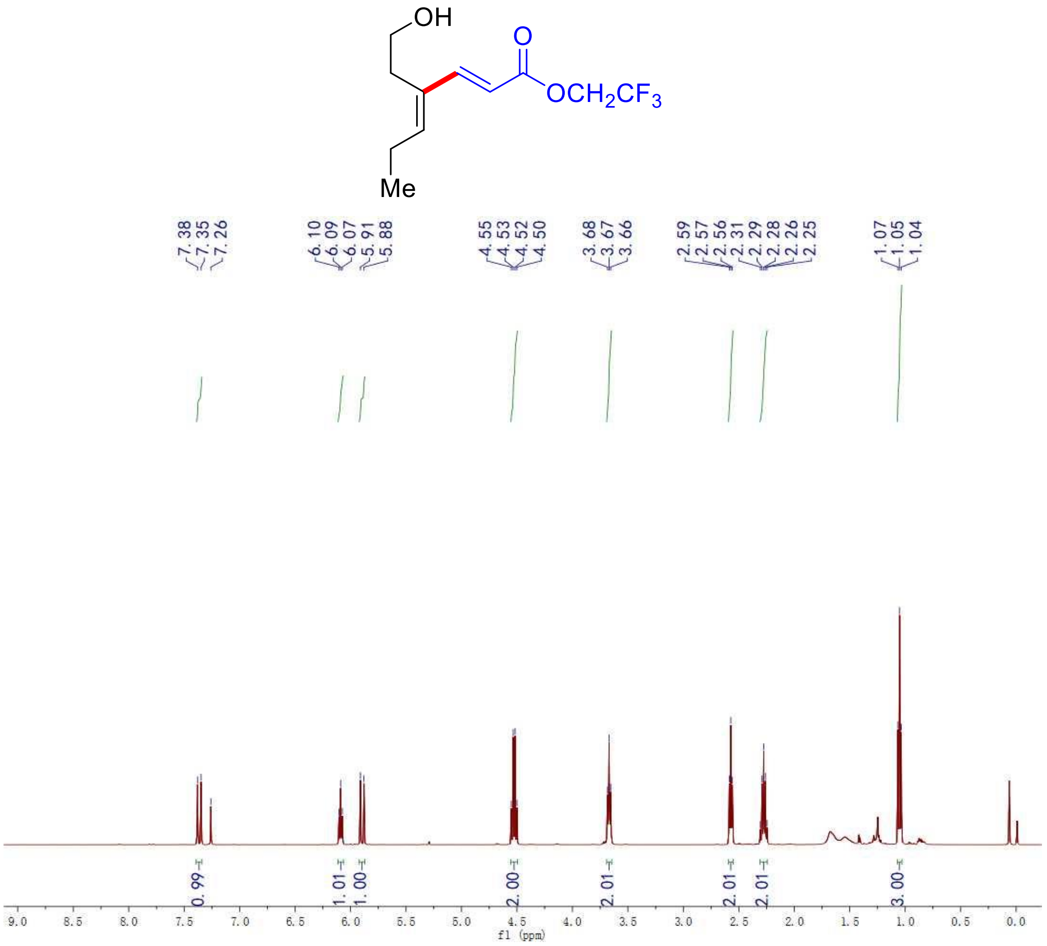


**Supplementary Figure 17.** $^{1}$H NMR spectrum for **3ag** in $CDCl_3$

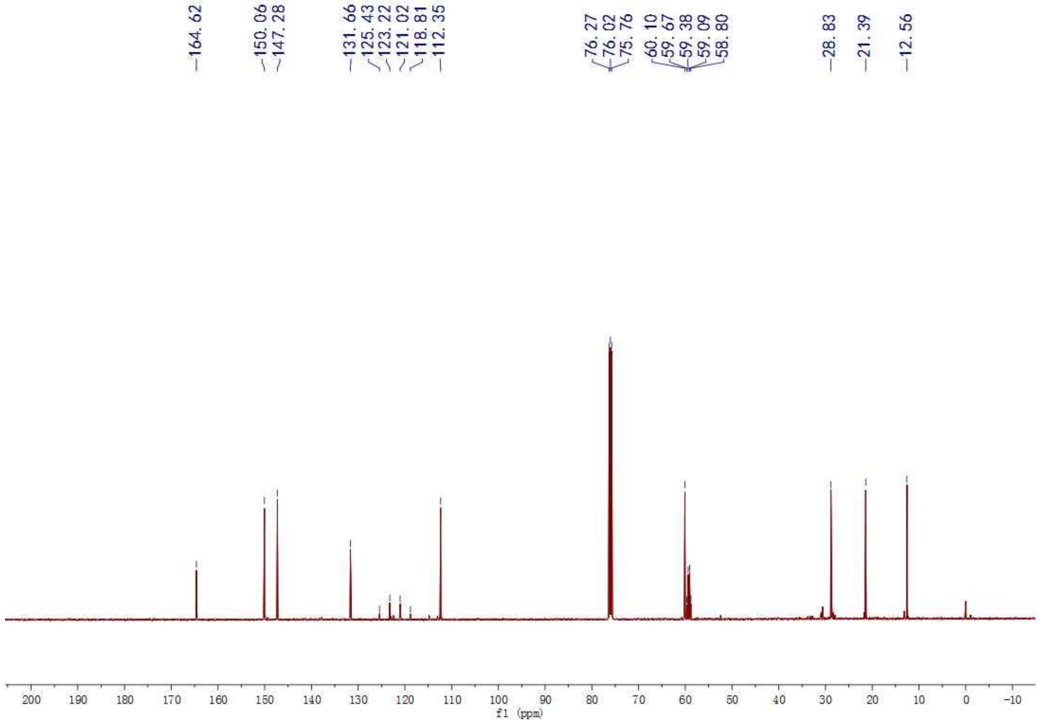


**Supplementary Figure 18.** $^{13}$C NMR spectrum for **3ag** in $CDCl_3$

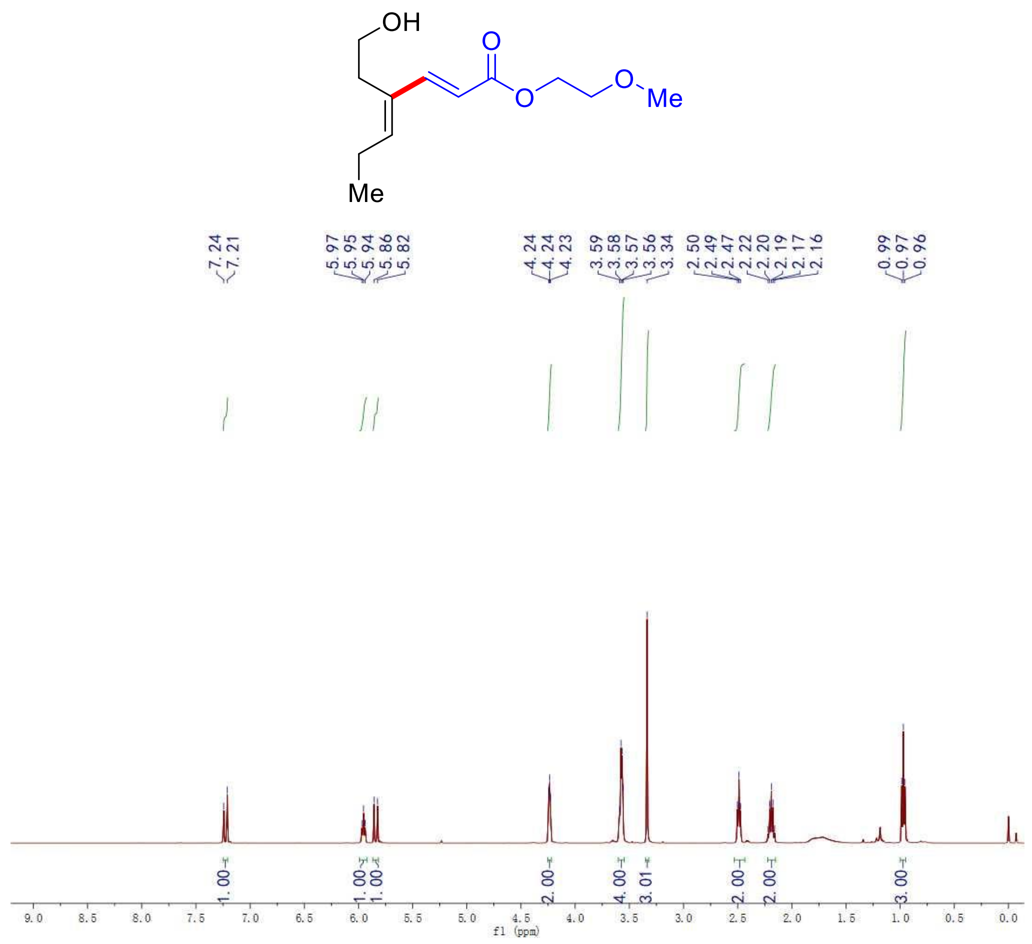


**Supplementary Figure 19.** $^{1}$H NMR spectrum for **3ah** in $CDCl_3$

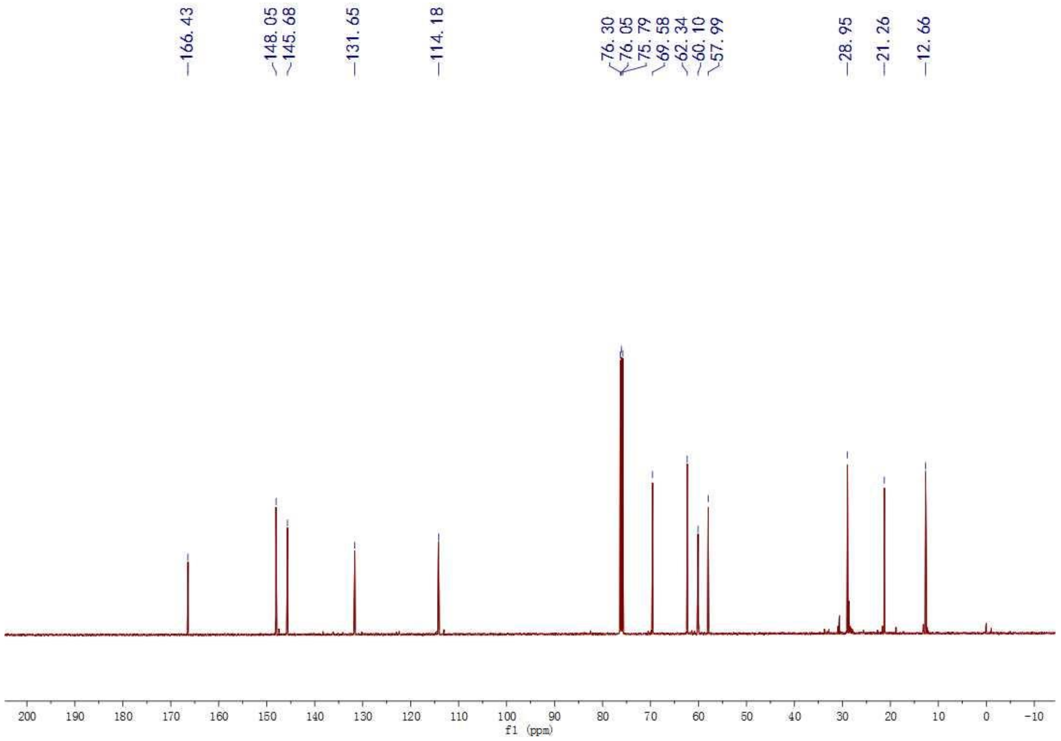


**Supplementary Figure 20.** $^{13}$C NMR spectrum for **3ah** in $CDCl_3$

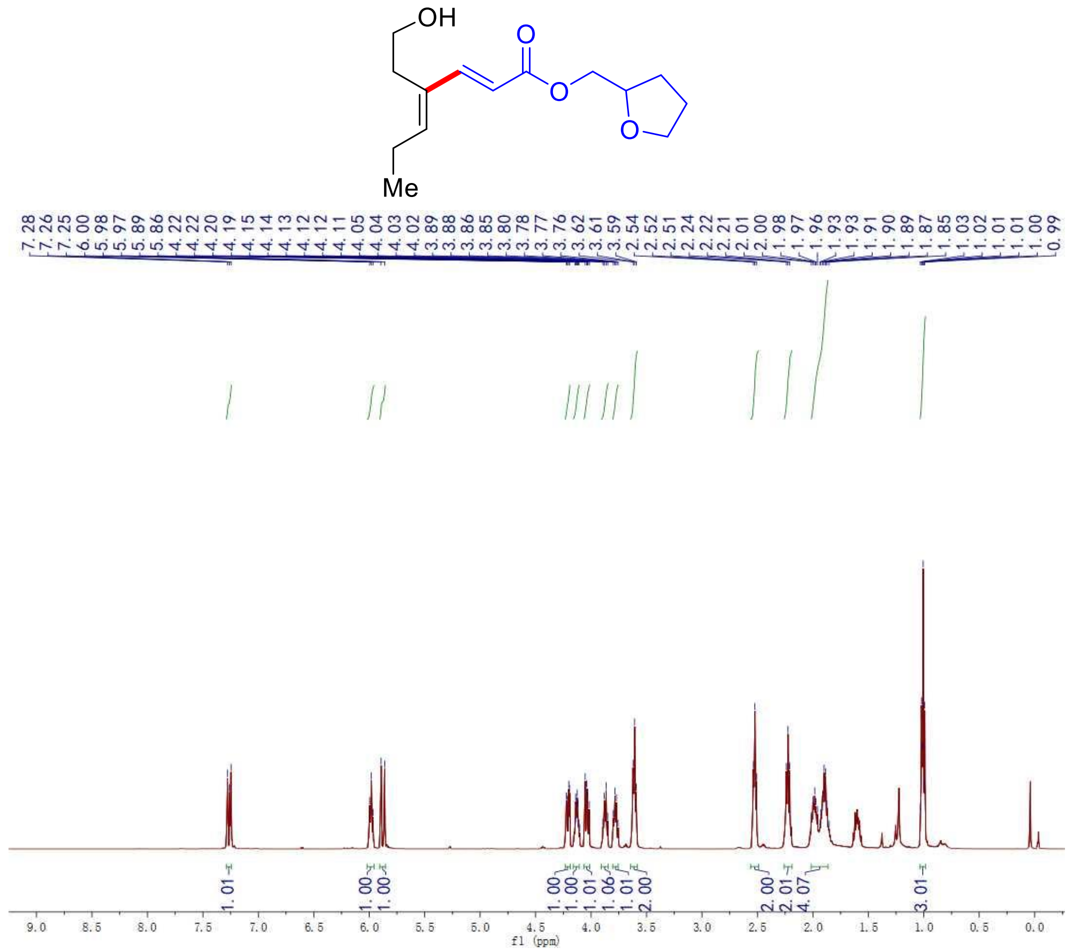


**Supplementary Figure 21.** $^{1}$H NMR spectrum for **3ai** in $CDCl_3$

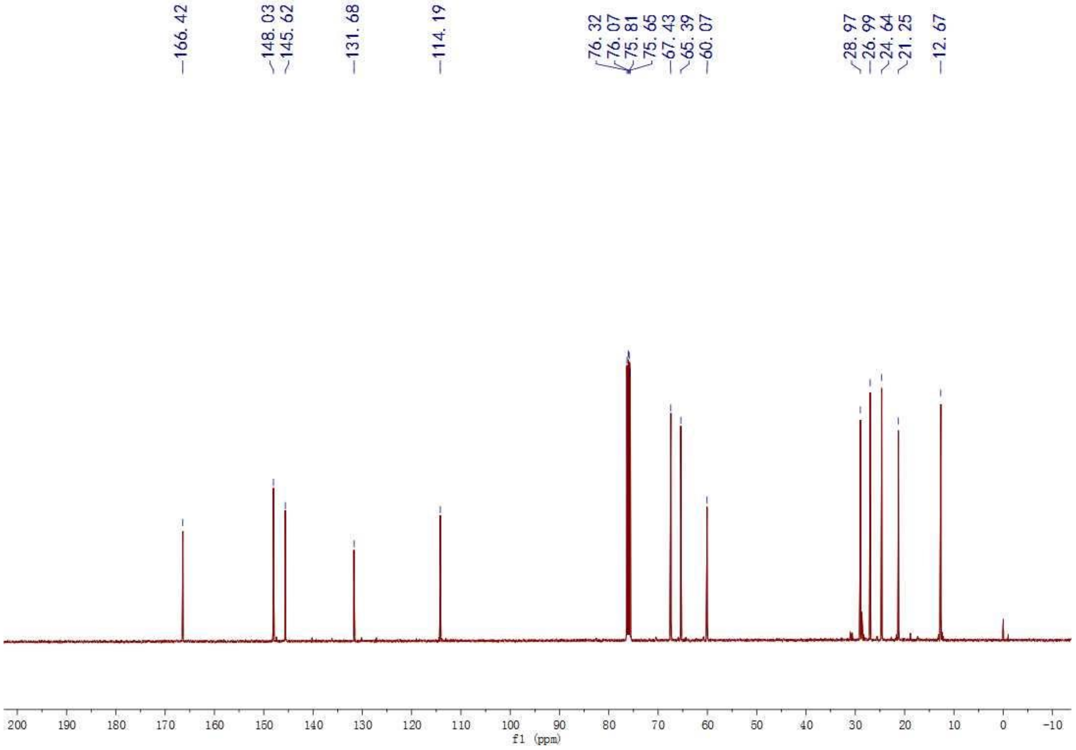


**Supplementary Figure 22.** $^{13}$C NMR spectrum for **3ai** in $CDCl_3$

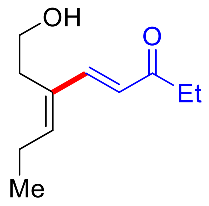


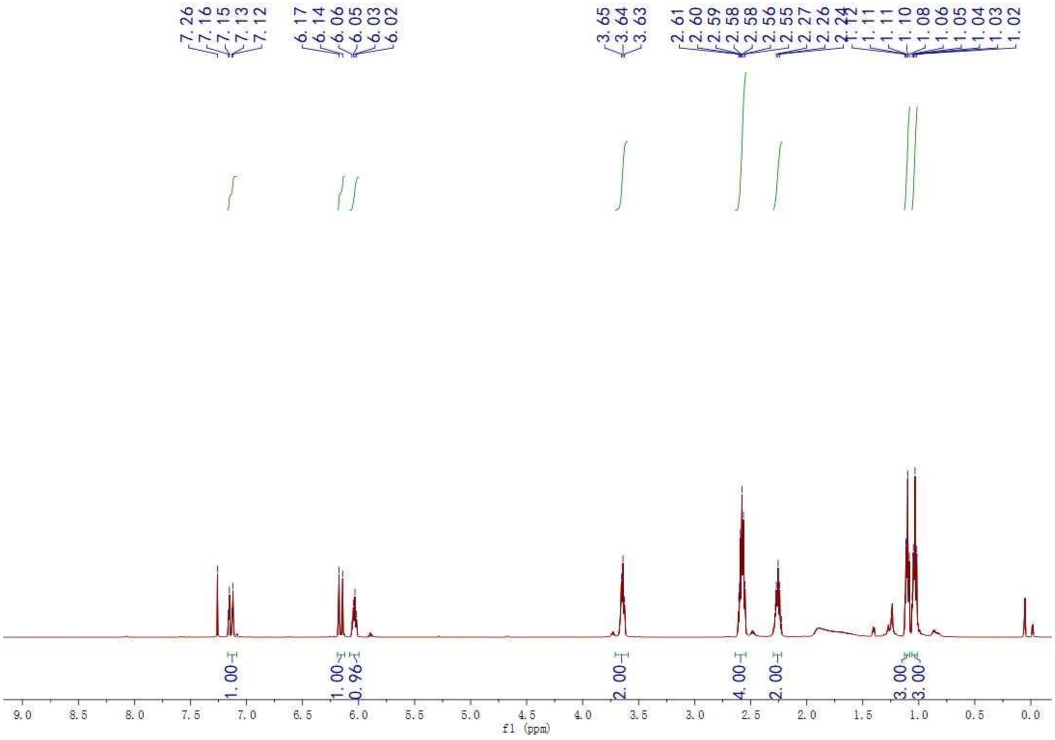


**Supplementary Figure 23.** $^{1}$H NMR spectrum for **3aj** in $CDCl_3$

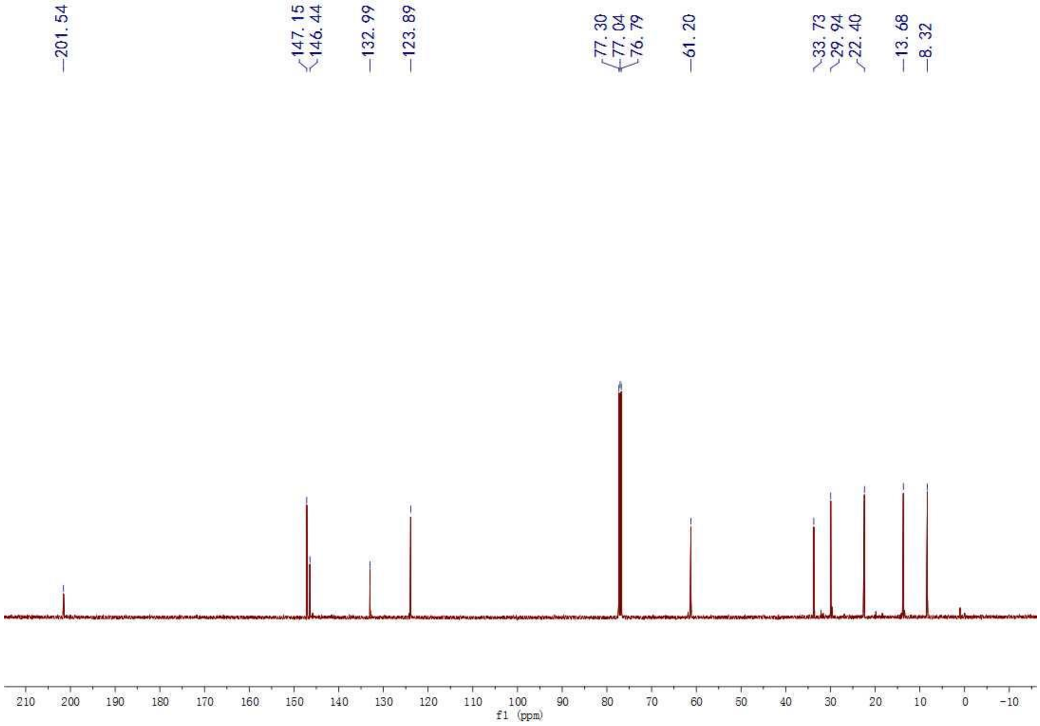


**Supplementary Figure 24.** $^{13}$C NMR spectrum for **3aj** in $CDCl_3$

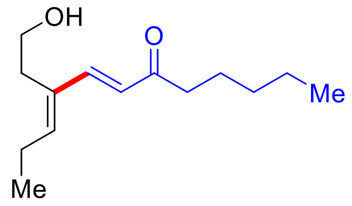


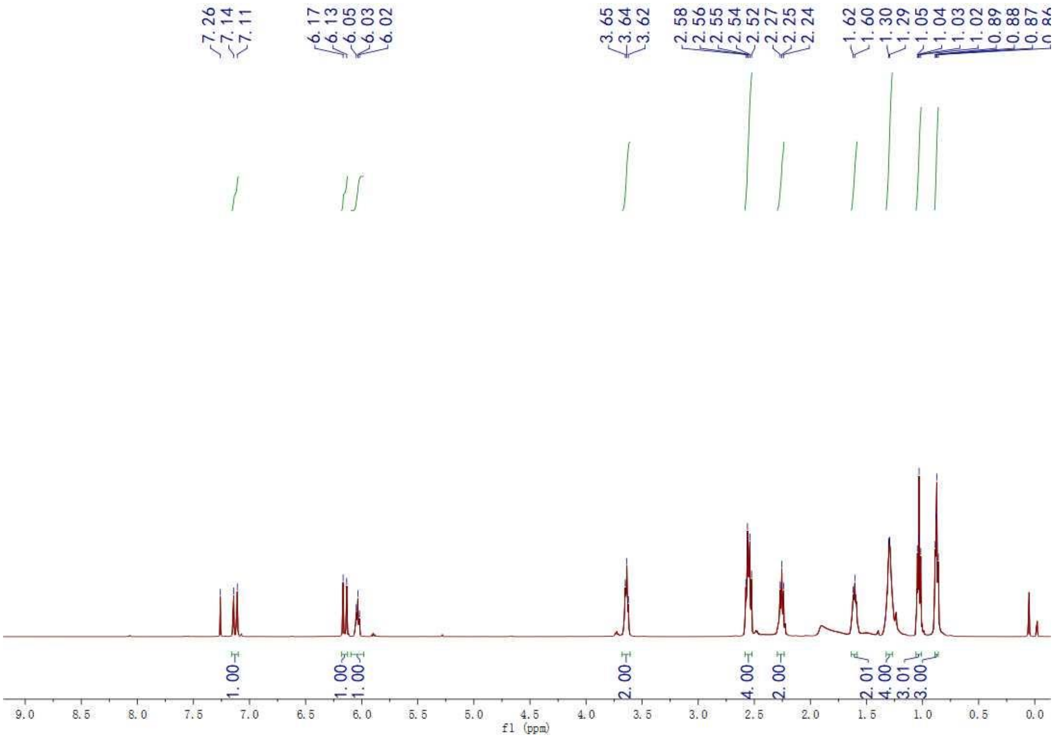


**Supplementary Figure 25.** $^{1}$H NMR spectrum for **3ak** in $CDCl_3$

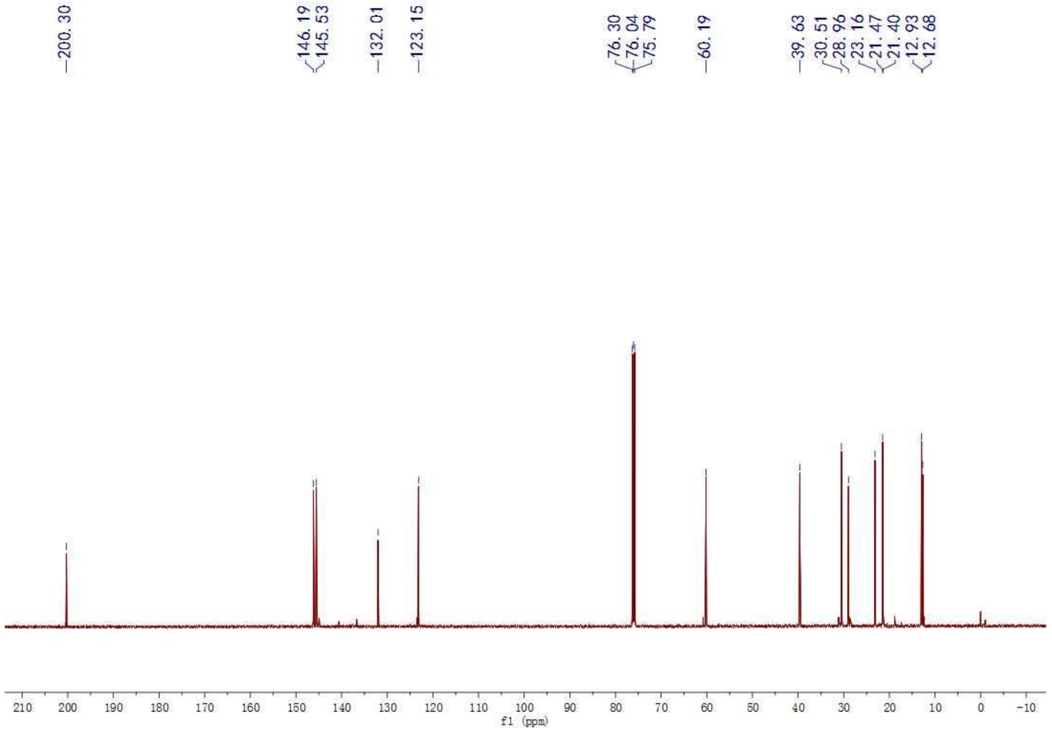


**Supplementary Figure 26.** $^{13}$C NMR spectrum for **3ak** in $CDCl_3$

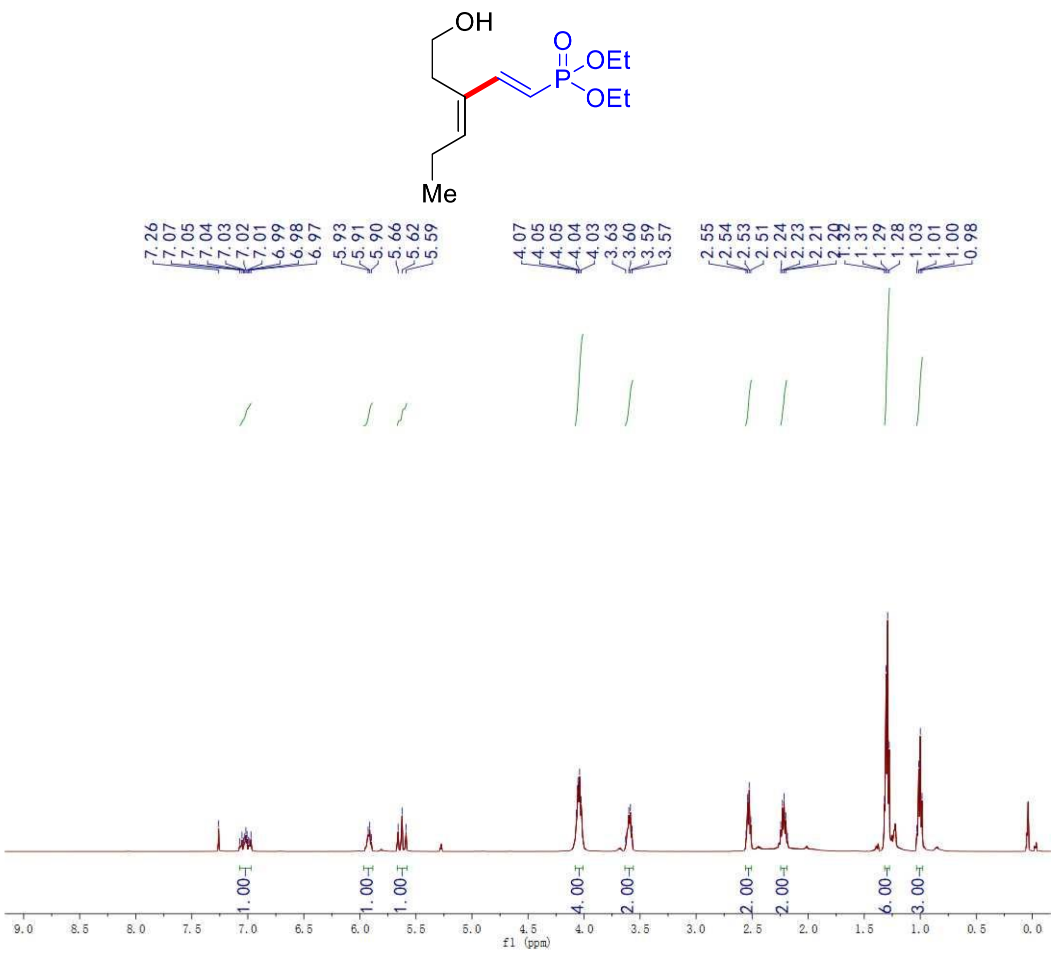


**Supplementary Figure 27.** $^{1}$H NMR spectrum for **3al** in $CDCl_3$

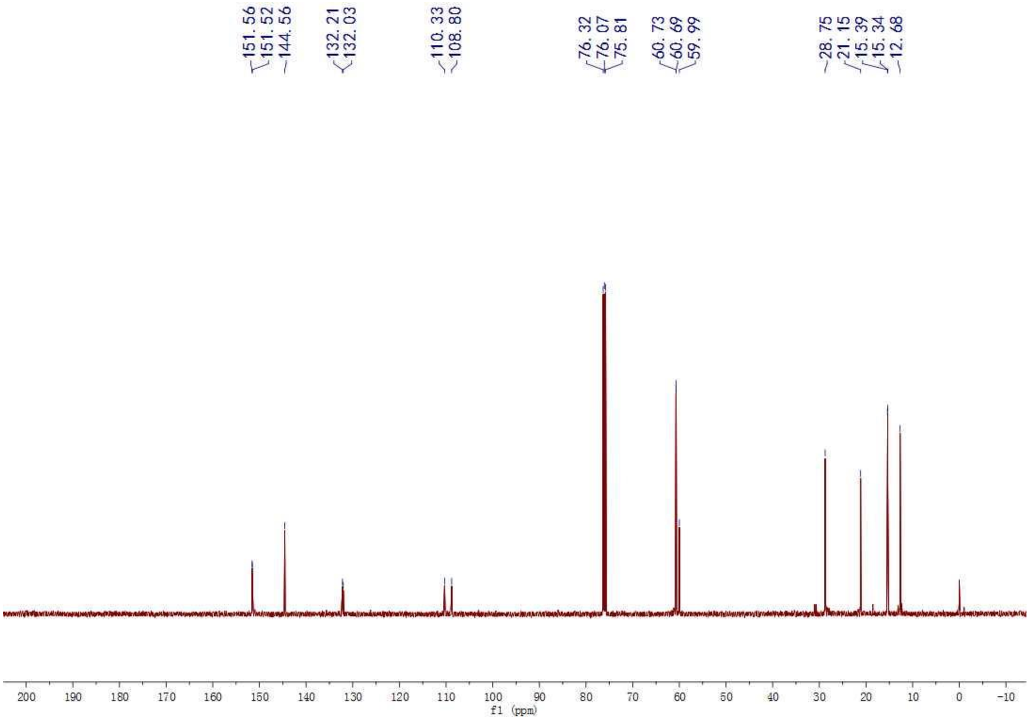


**Supplementary Figure 28.** $^{13}$C NMR spectrum for **3al** in $CDCl_3$

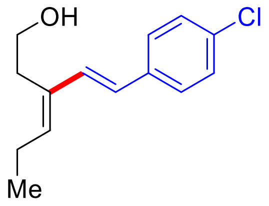


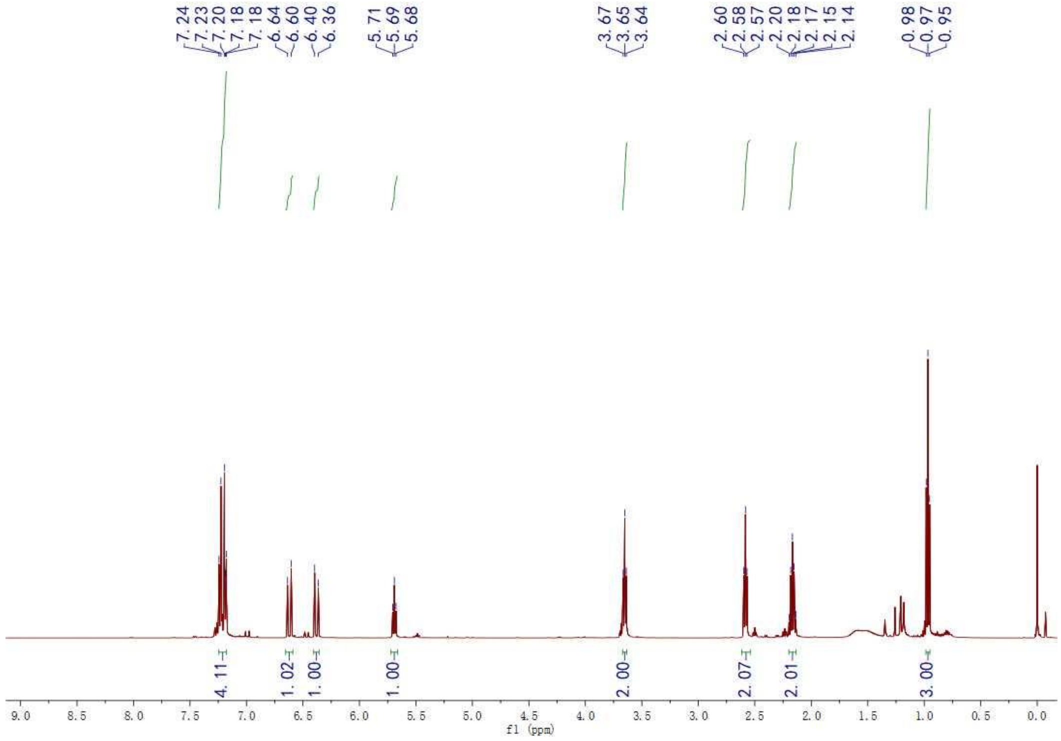


**Supplementary Figure 29.** $^{1}$H NMR spectrum for **3am** in $CDCl_3$

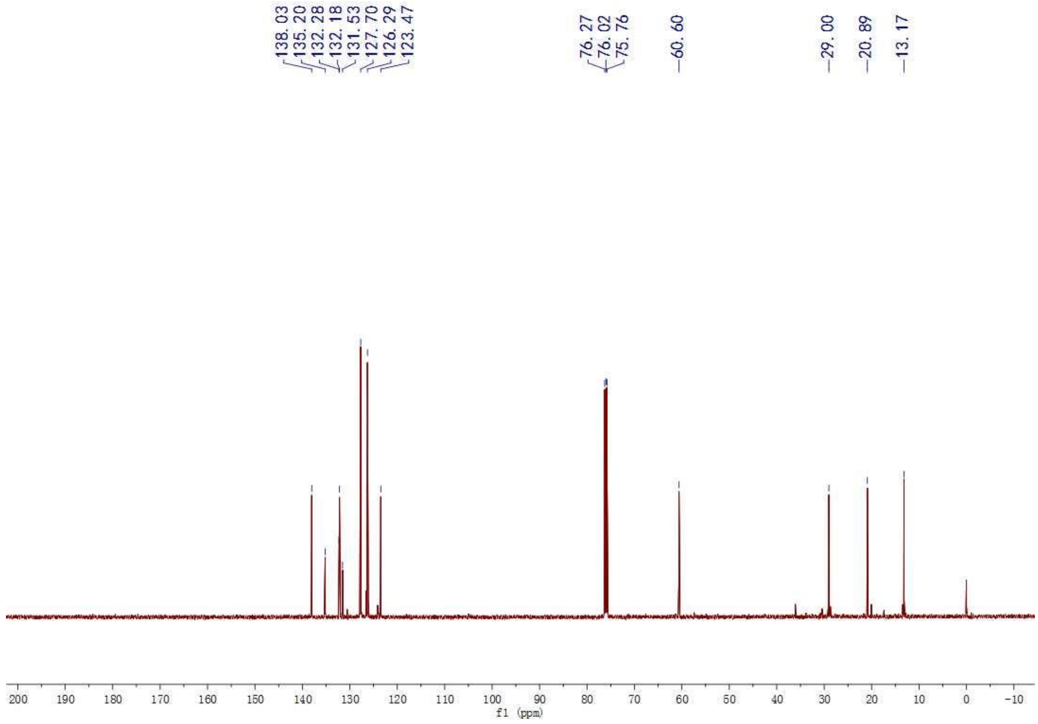


**Supplementary Figure 30.** $^{13}$C NMR spectrum for **3am** in $CDCl_3$

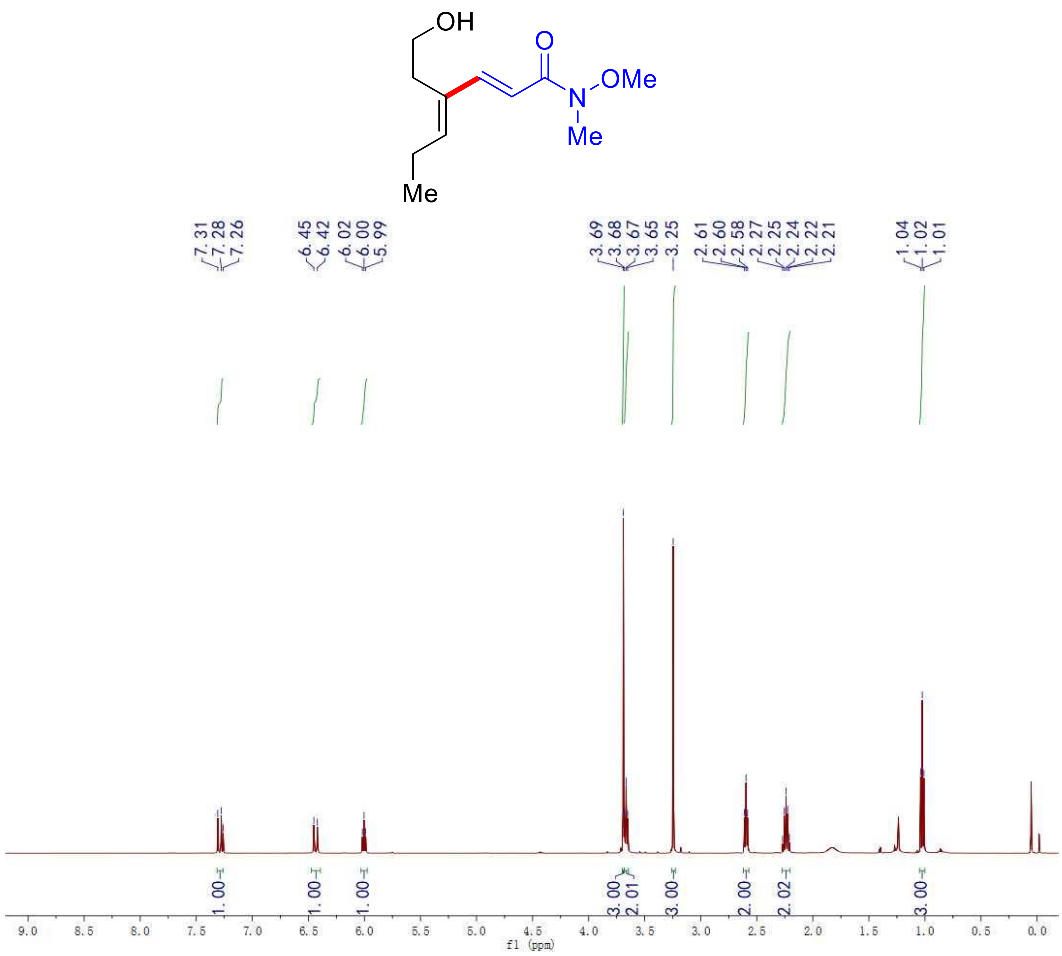


**Supplementary Figure 31.** $^{1}$H NMR spectrum for **3an** in $CDCl_3$

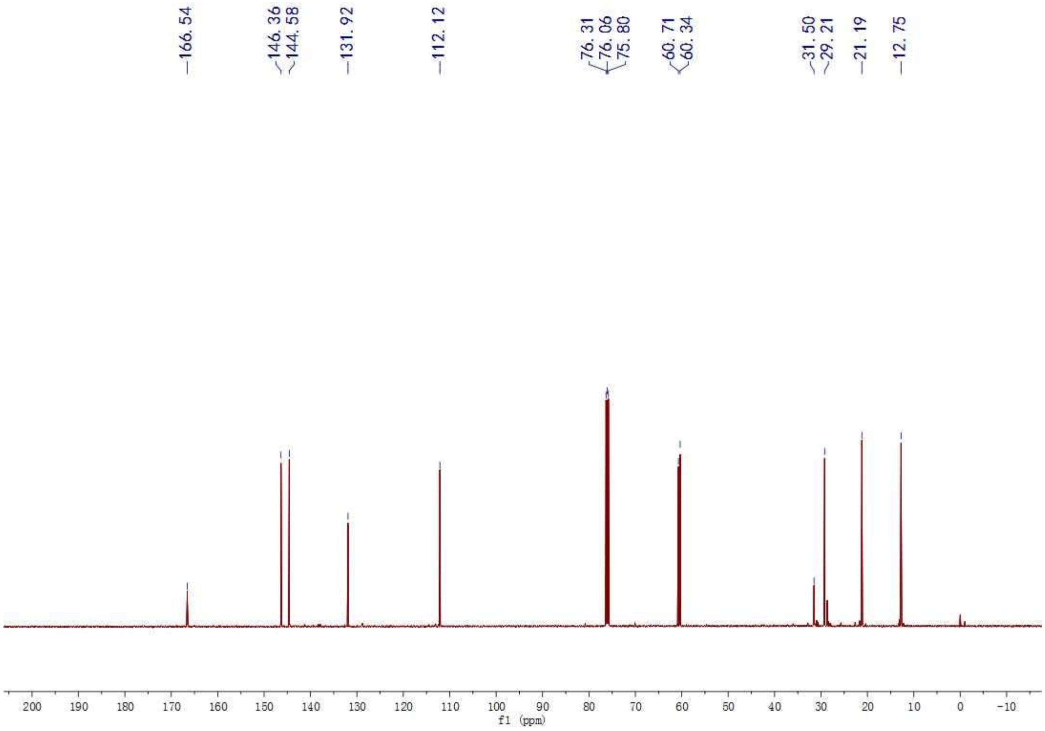


**Supplementary Figure 32.** $^{13}$C NMR spectrum for **3an** in $CDCl_3$

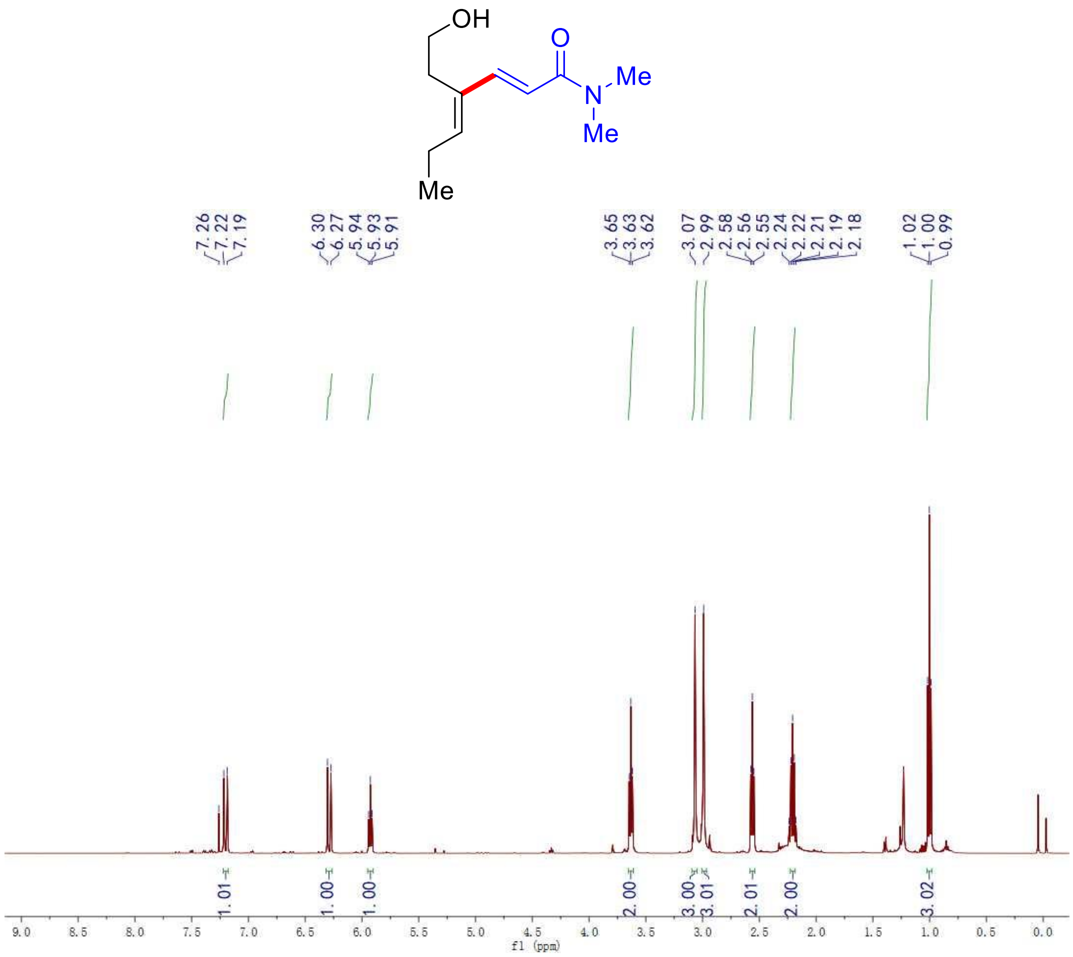


**Supplementary Figure 33.** $^{1}$H NMR spectrum for **3ao** in $CDCl_3$

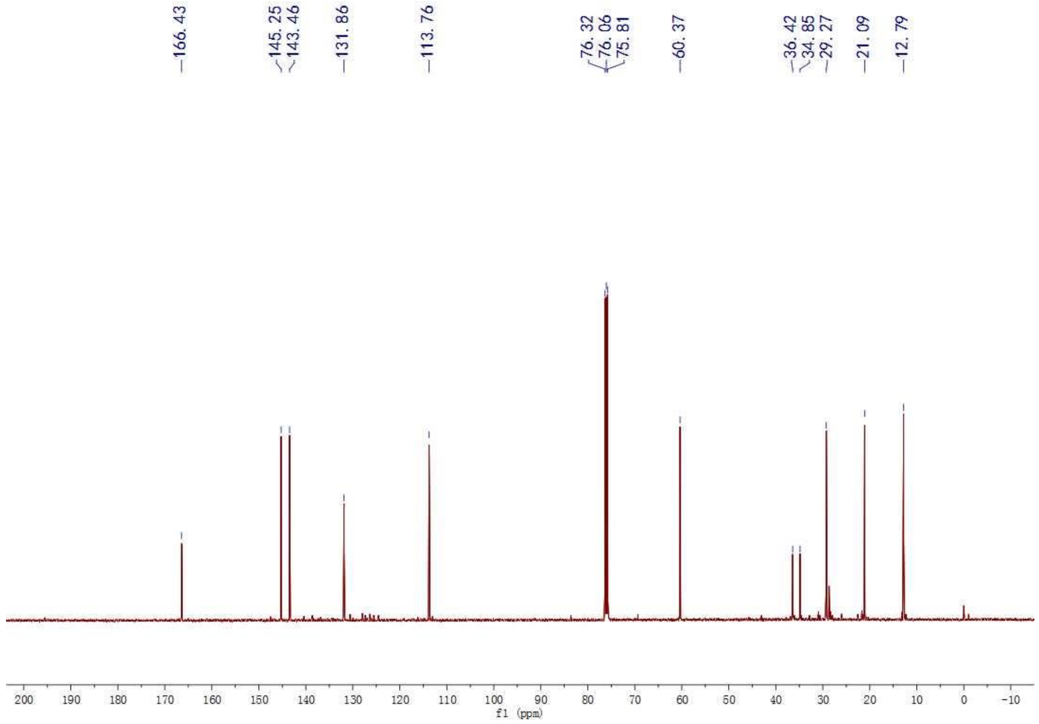


**Supplementary Figure 34.** $^{13}$C NMR spectrum for **3ao** in $CDCl_3$

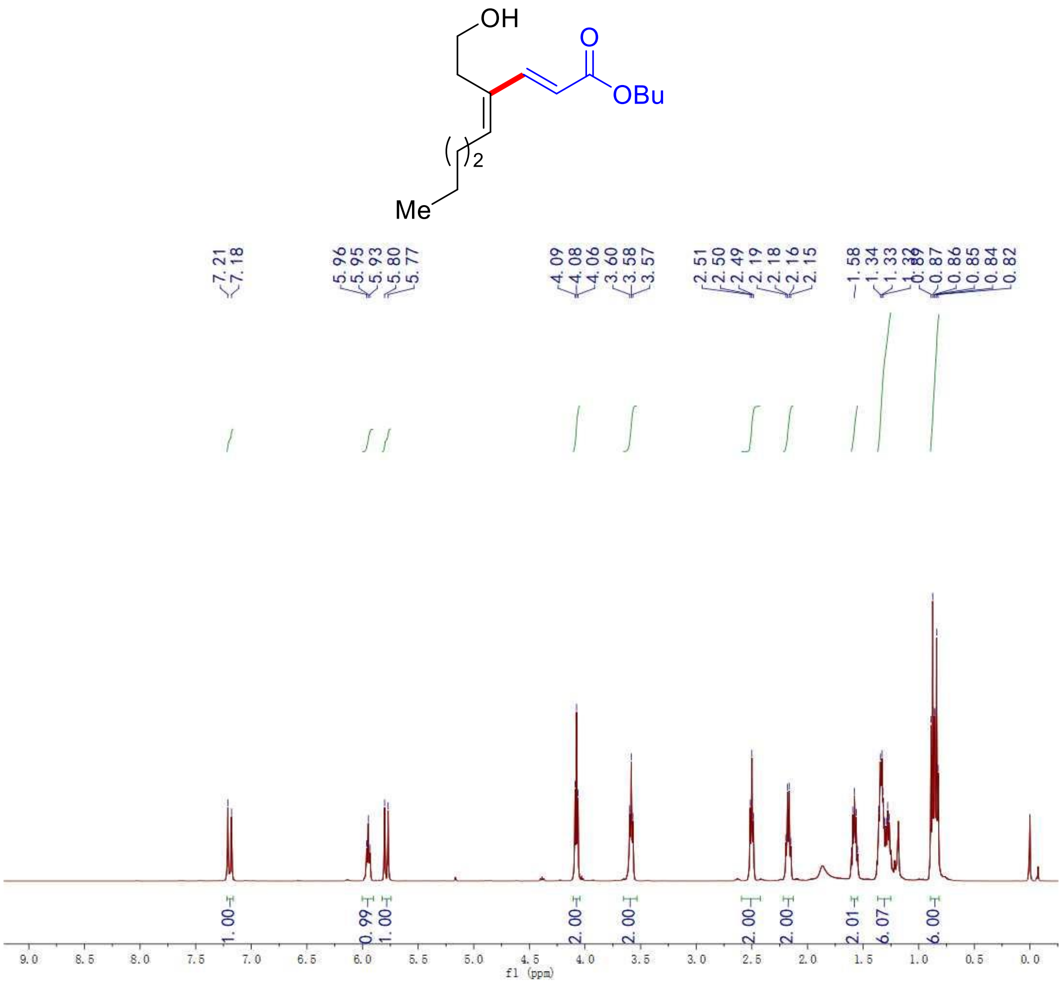

**Supplementary Figure 35.** $^{1}H$ NMR spectrum for **3ba** in $CDCl_3$

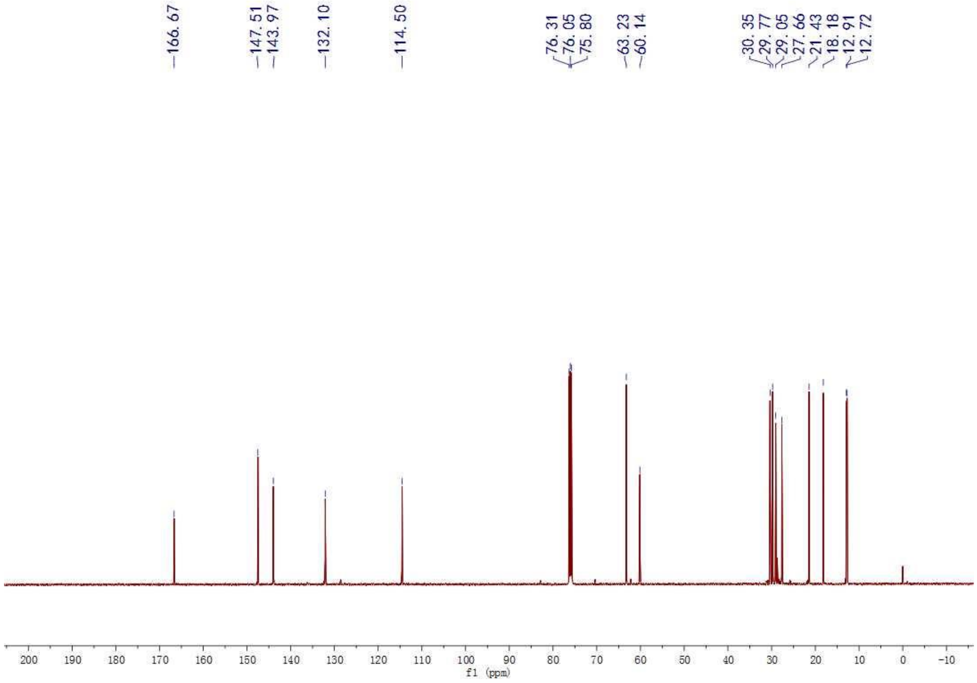

**Supplementary Figure 36.** $^{13}C$ NMR spectrum for **3ba** in $CDCl_3$

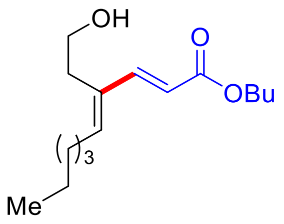


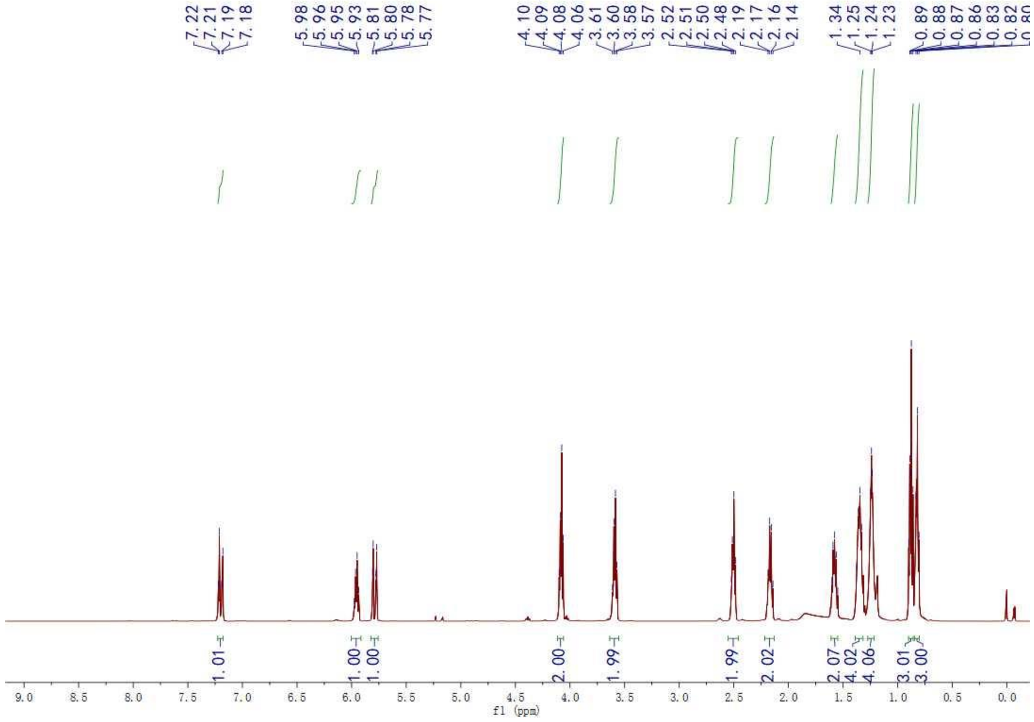


**Supplementary Figure 37.** $^{1}H$ NMR spectrum for **3ca** in $CDCl_3$

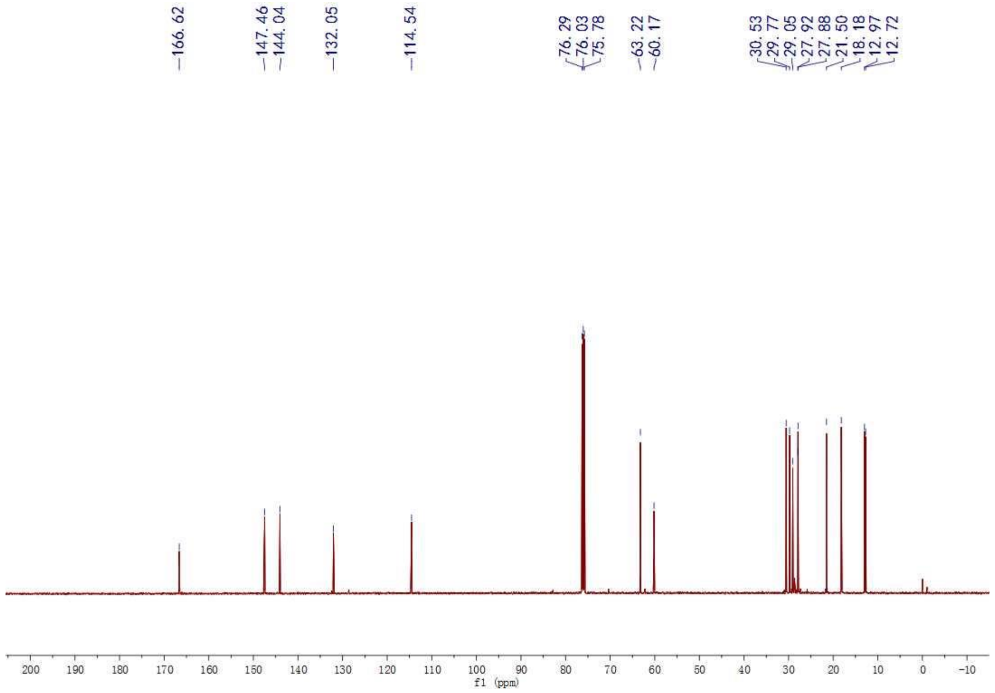


**Supplementary Figure 38.** $^{13}C$ NMR spectrum for **3ca** in $CDCl_3$

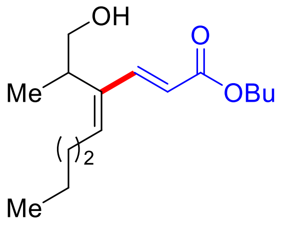


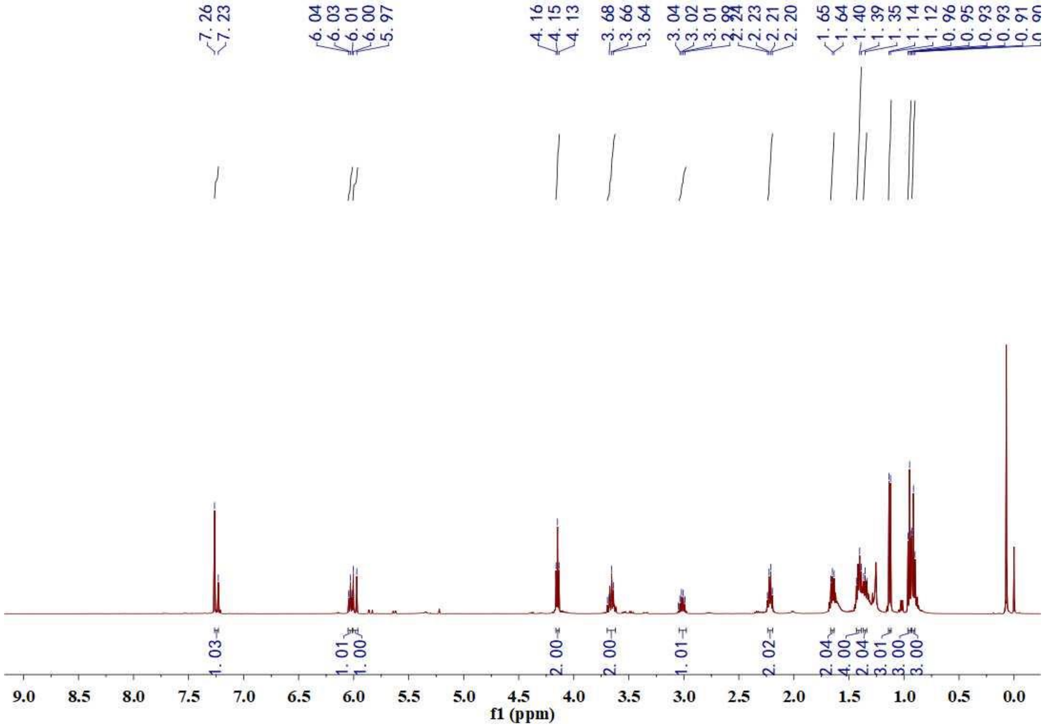


**Supplementary Figure 39.** $^{1}$H NMR spectrum for **3da** in $CDCl_3$

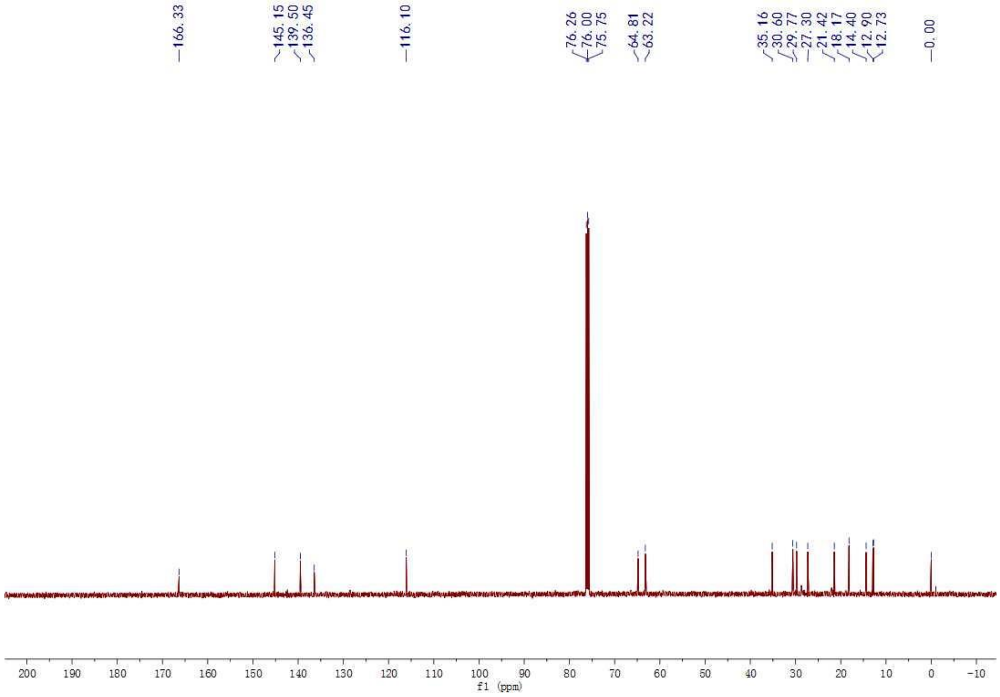


**Supplementary Figure 40.** $^{13}$C NMR spectrum for **3da** in $CDCl_3$

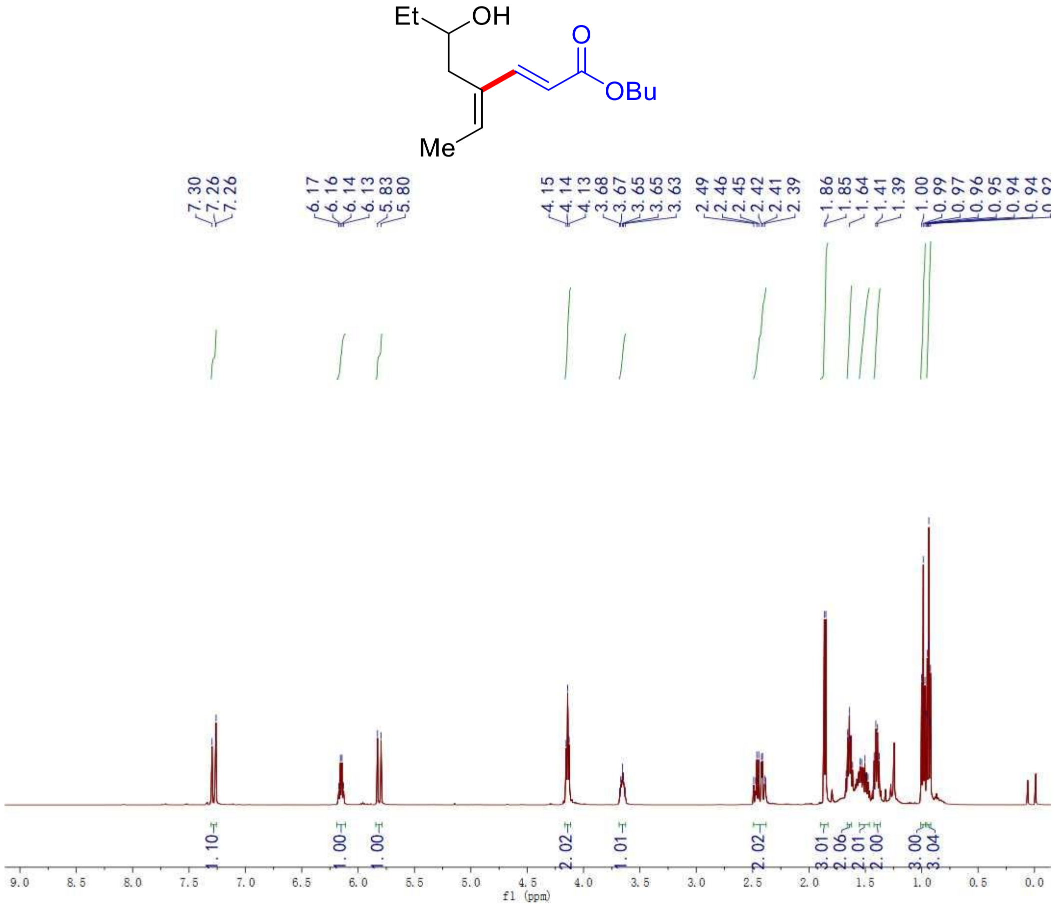


**Supplementary Figure 41.** $^{1}$H NMR spectrum for **3ea** in $CDCl_3$

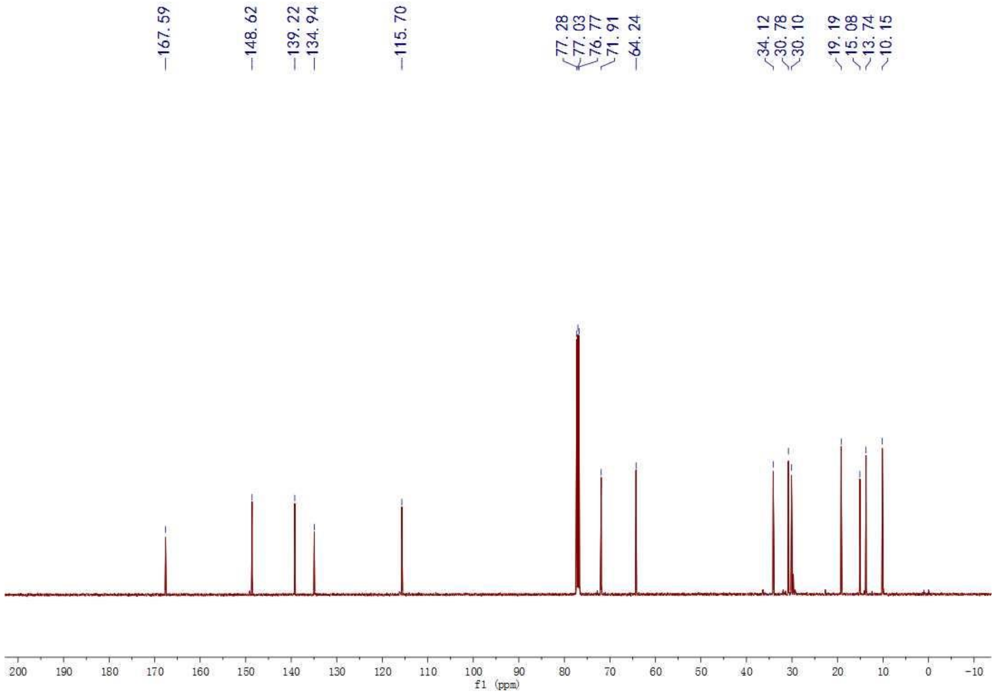


**Supplementary Figure 42.** $^{13}$C NMR spectrum for **3ea** in $CDCl_3$

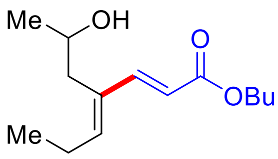


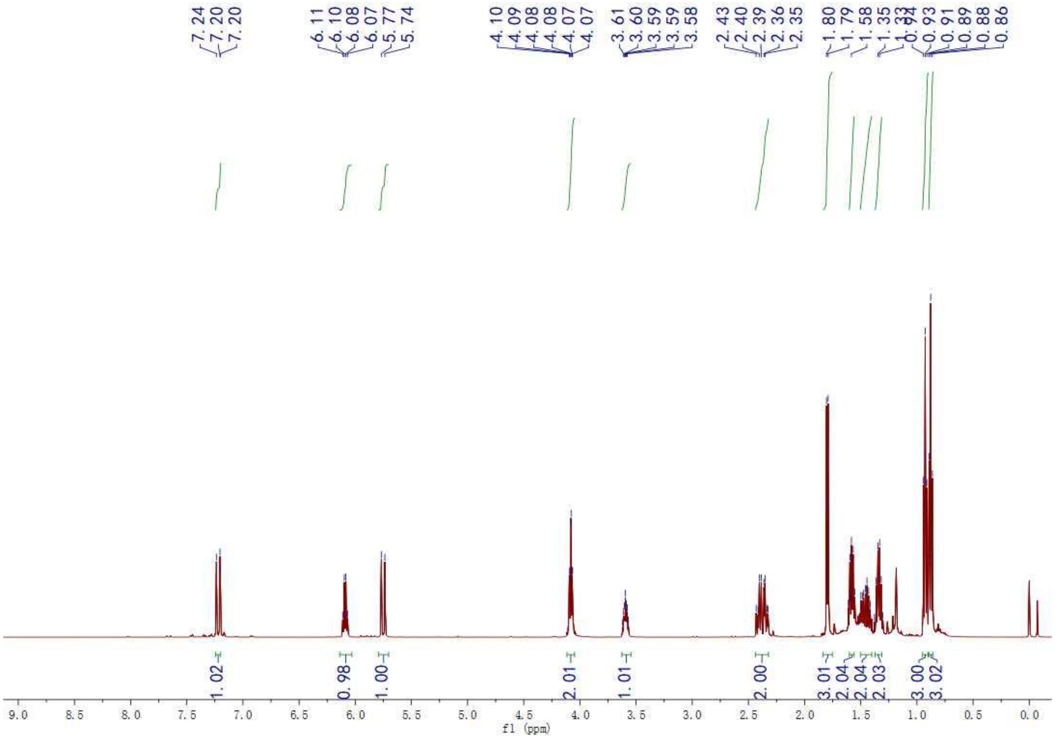


**Supplementary Figure 43.** $^{1}$H NMR spectrum for **3fa** in $CDCl_3$

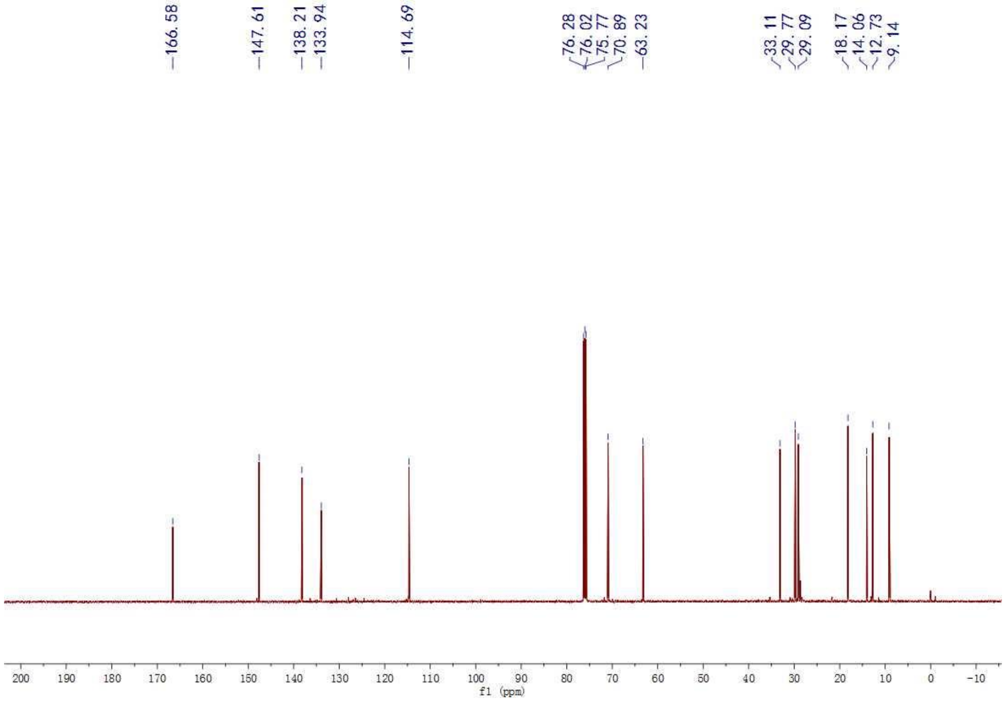


**Supplementary Figure 44.** $^{13}$C NMR spectrum for **3fa** in $CDCl_3$

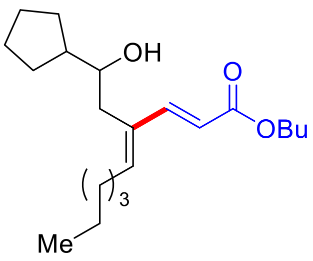


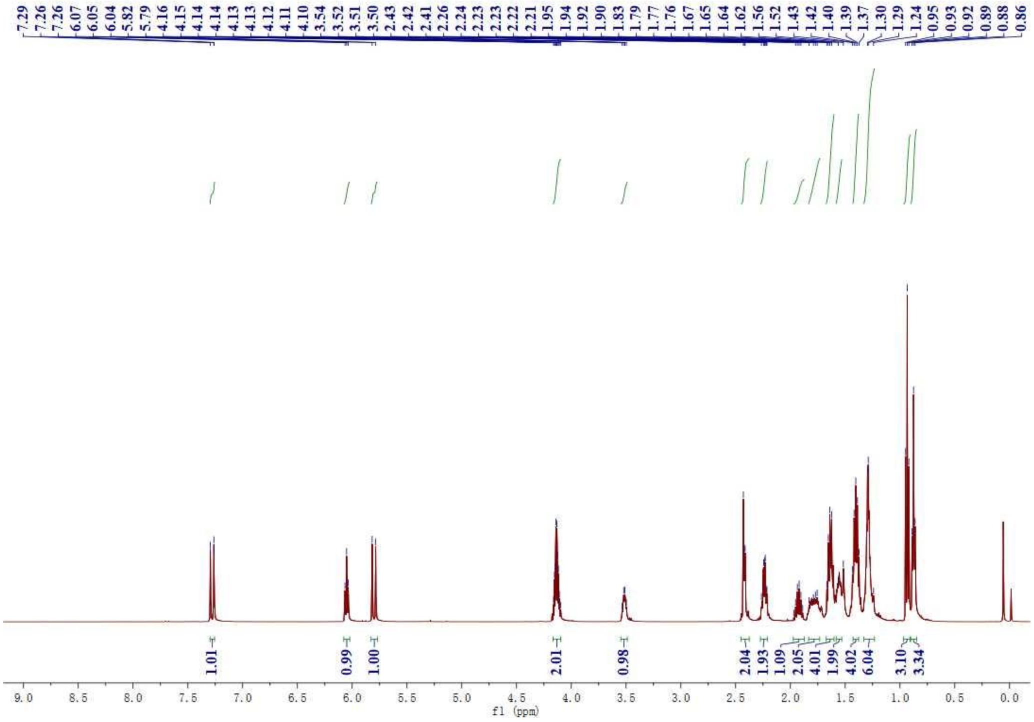


**Supplementary Figure 45.** $^{1}$H NMR spectrum for **3ga** in $CDCl_3$

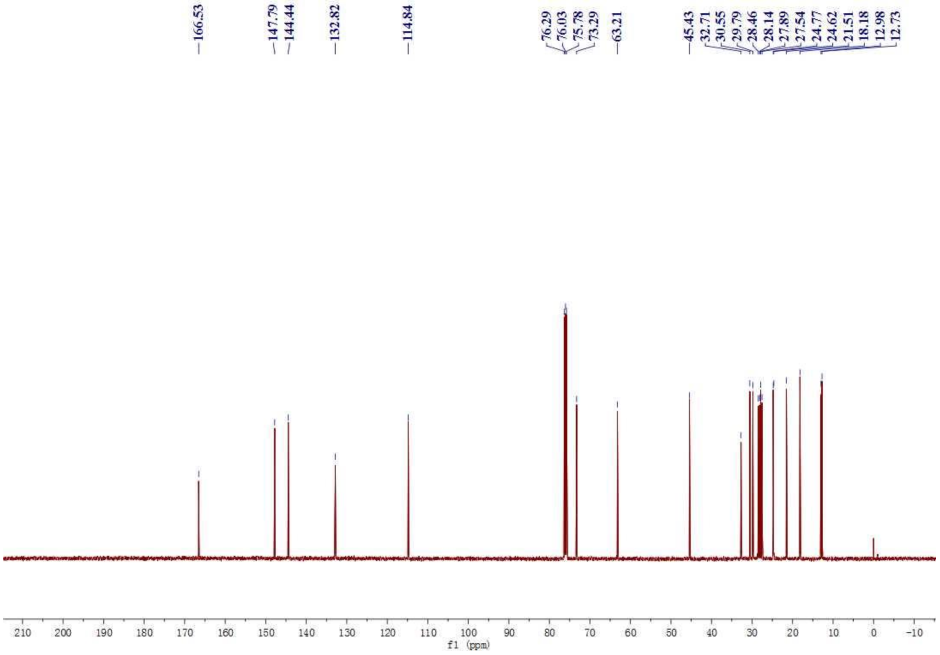


**Supplementary Figure 46.** $^{13}$C NMR spectrum for **3ga** in $CDCl_3$

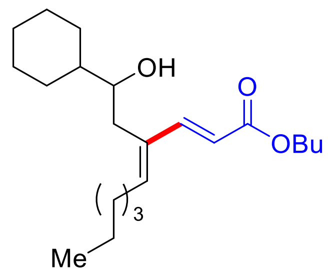


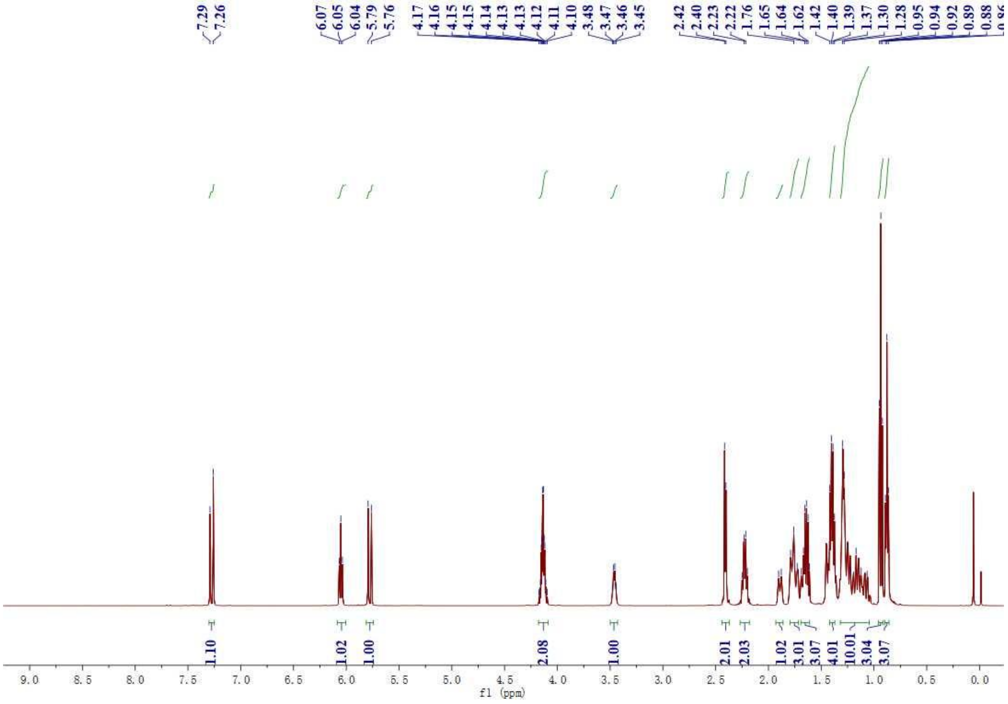


**Supplementary Figure 47.** $^{1}$H NMR spectrum for **3ha** in $CDCl_3$

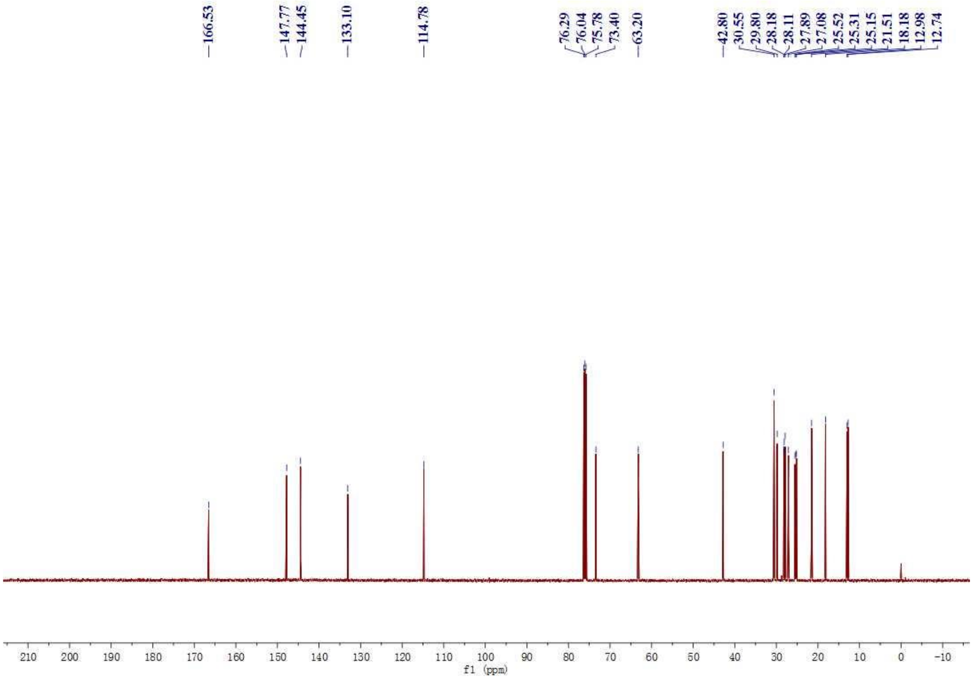


**Supplementary Figure 48.** $^{13}$C NMR spectrum for **3ha** in $CDCl_3$

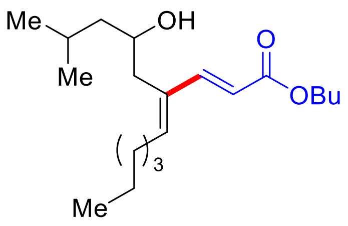


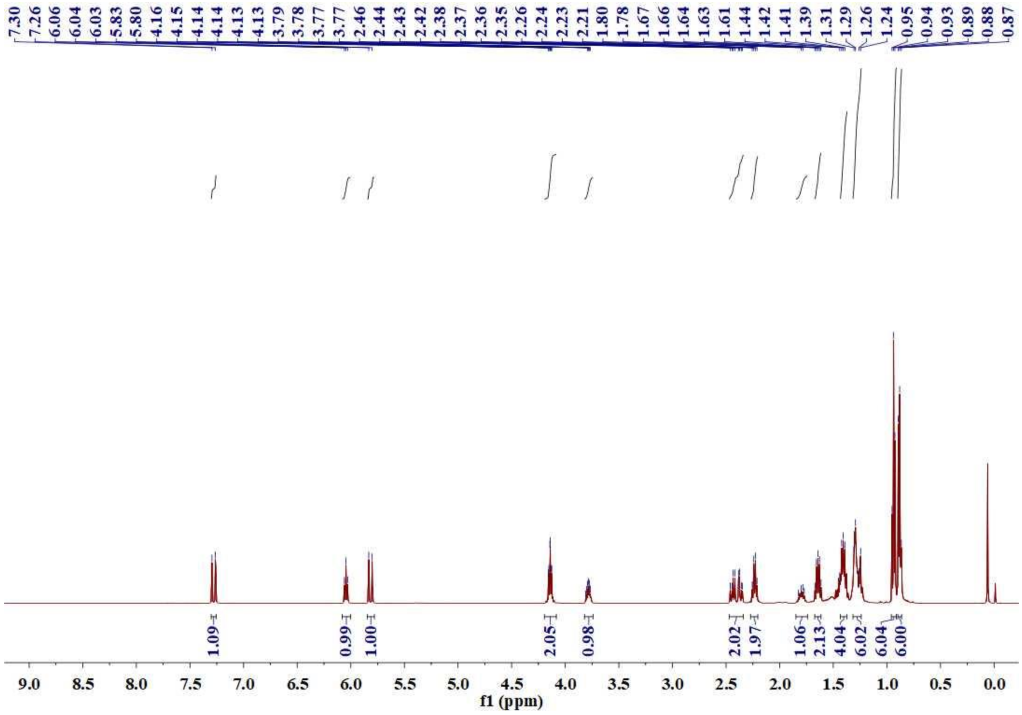


**Supplementary Figure 49.** $^{1}H$ NMR spectrum for **3ia** in $CDCl_3$

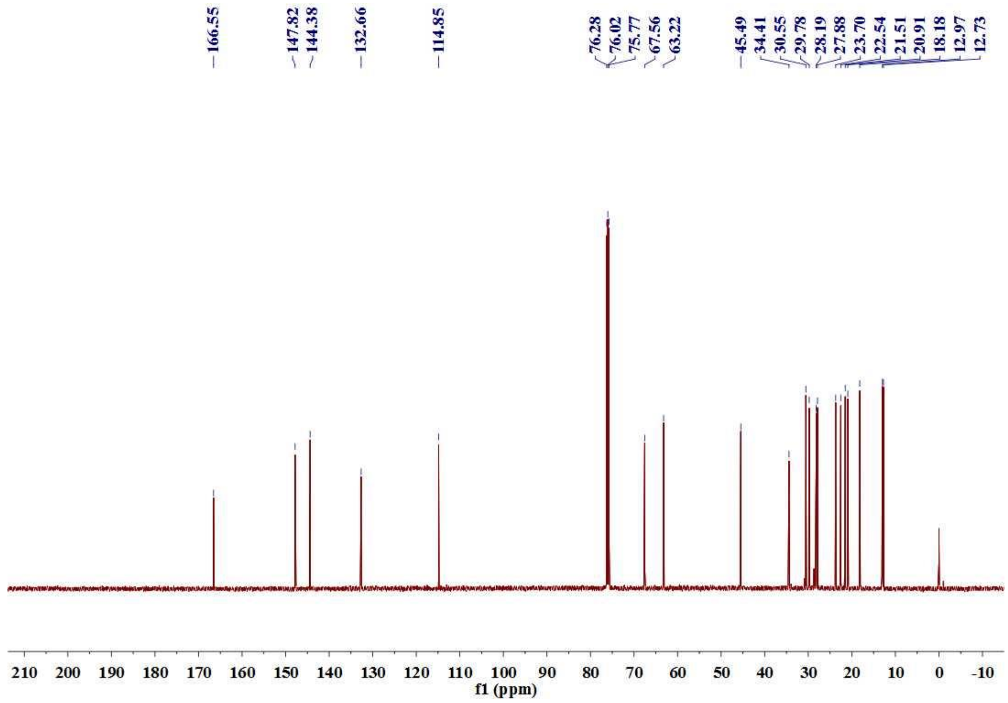


**Supplementary Figure 50.** $^{13}C$ NMR spectrum for **3ia** in $CDCl_3$

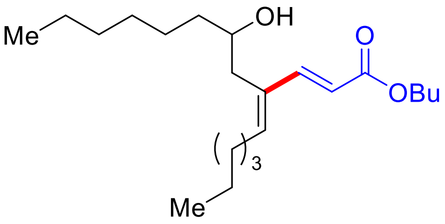


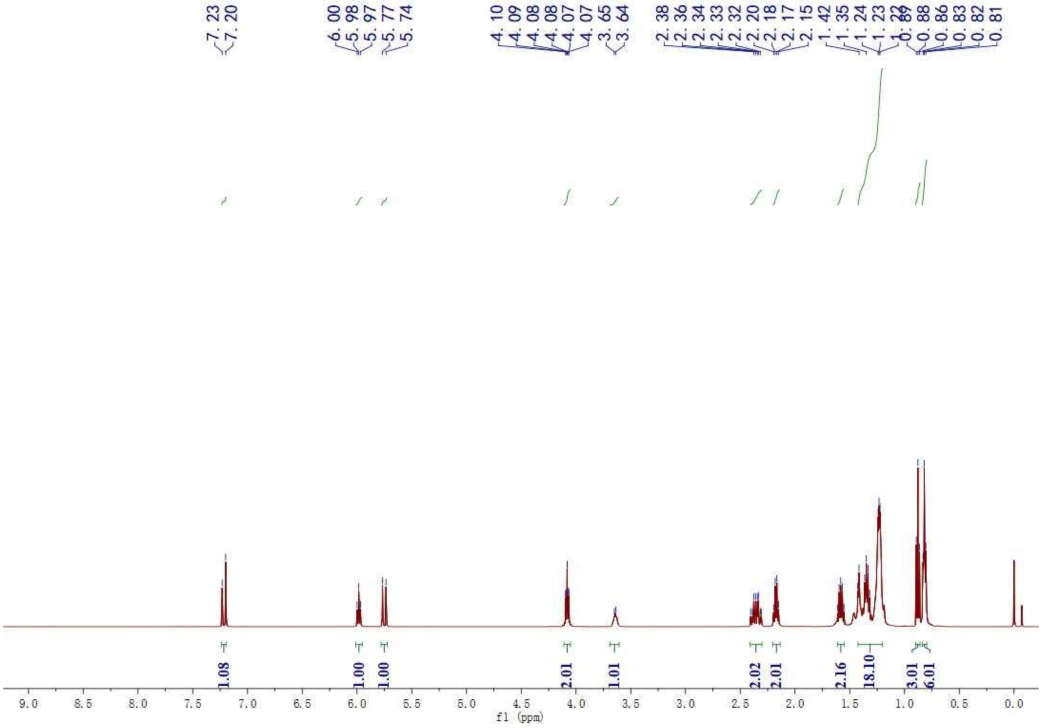


**Supplementary Figure 51.** $^{1}$H NMR spectrum for **3ja** in $CDCl_3$

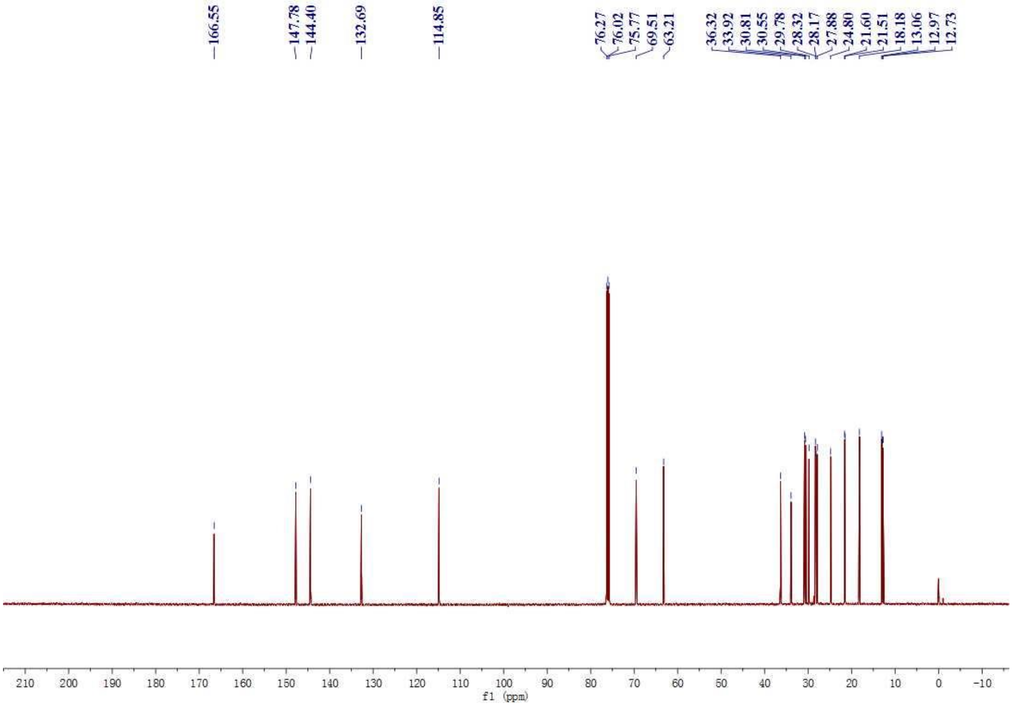


**Supplementary Figure 52.** $^{13}$C NMR spectrum for **3ja** in $CDCl_3$

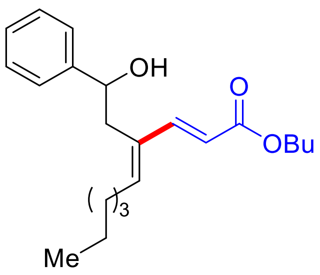


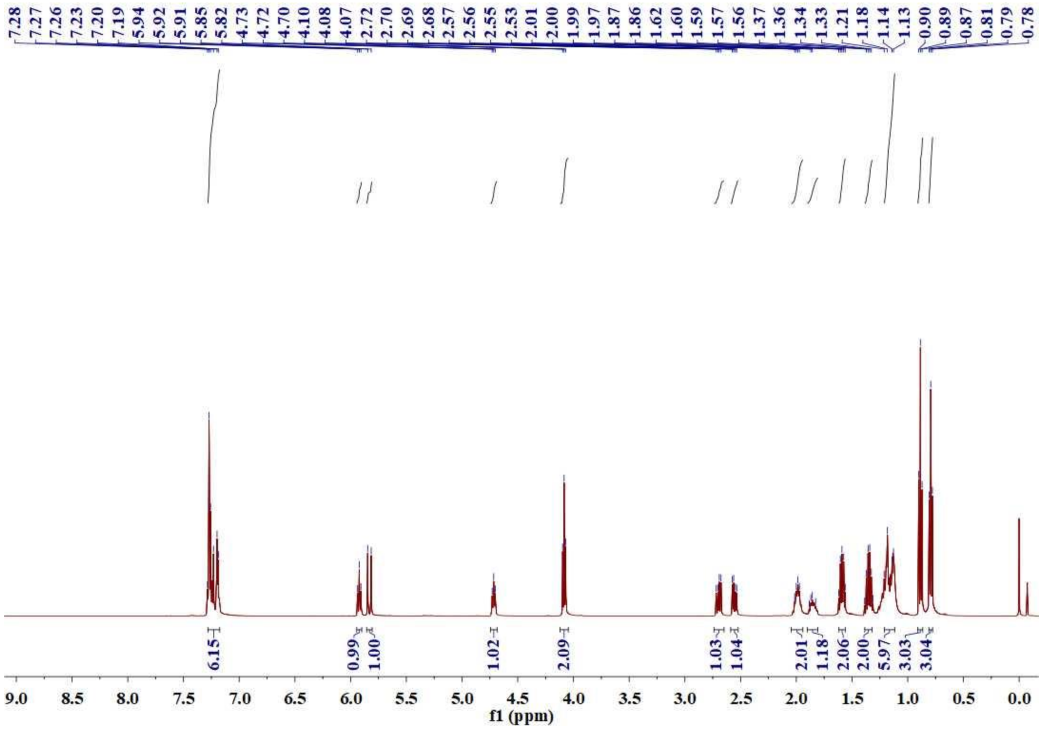


**Supplementary Figure 53.** $^{1}H$ NMR spectrum for **3ka** in $CDCl_3$

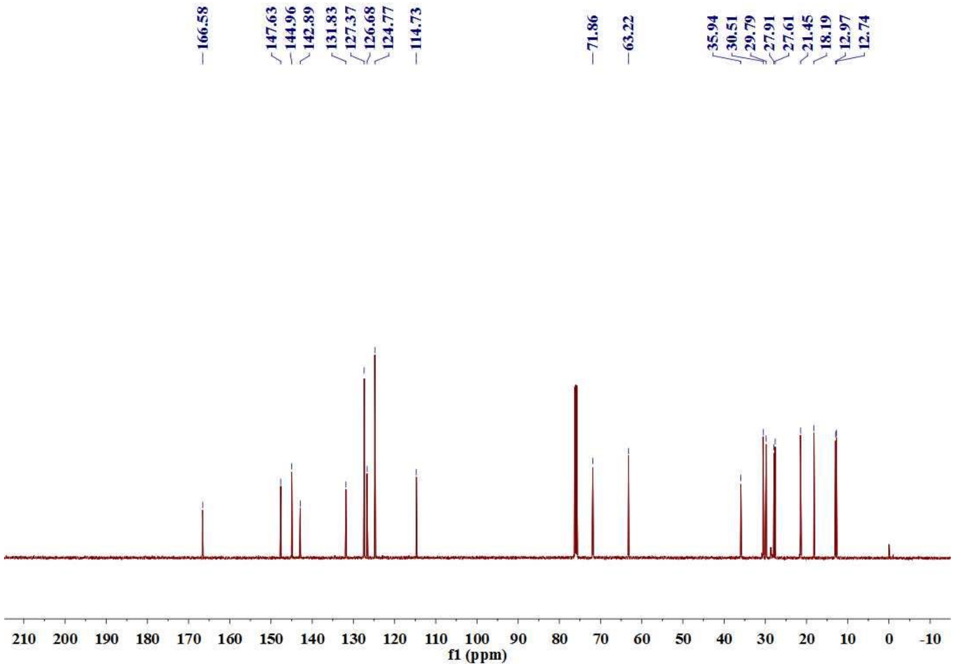


**Supplementary Figure 54.** $^{13}C$ NMR spectrum for **3ka** in $CDCl_3$

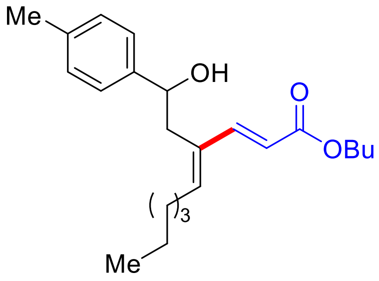


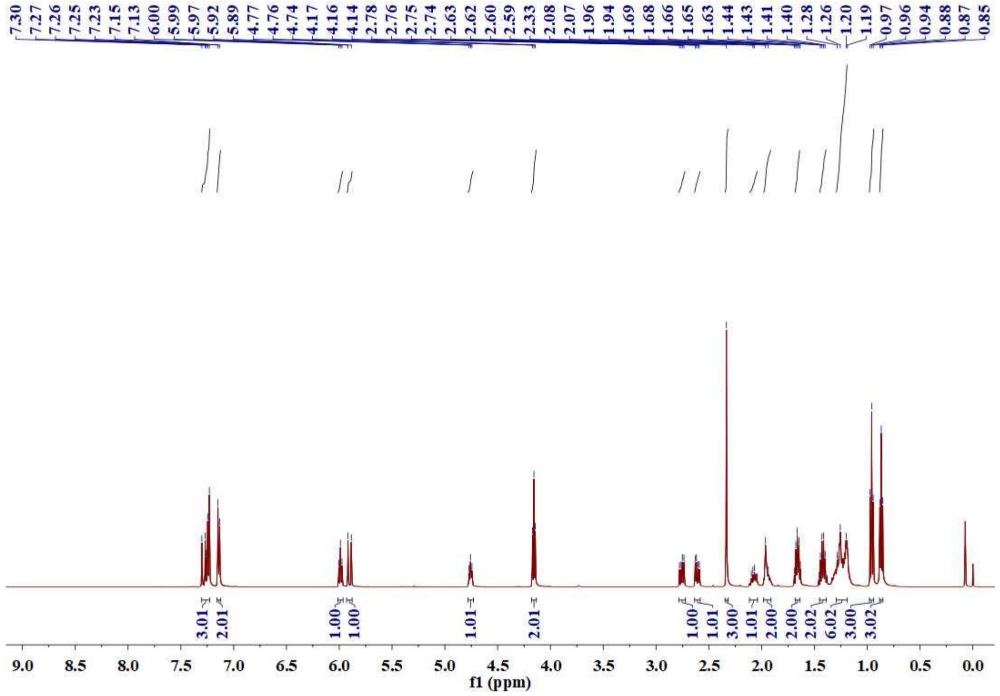


**Supplementary Figure 55.** $^{1}$H NMR spectrum for **3la** in $CDCl_3$

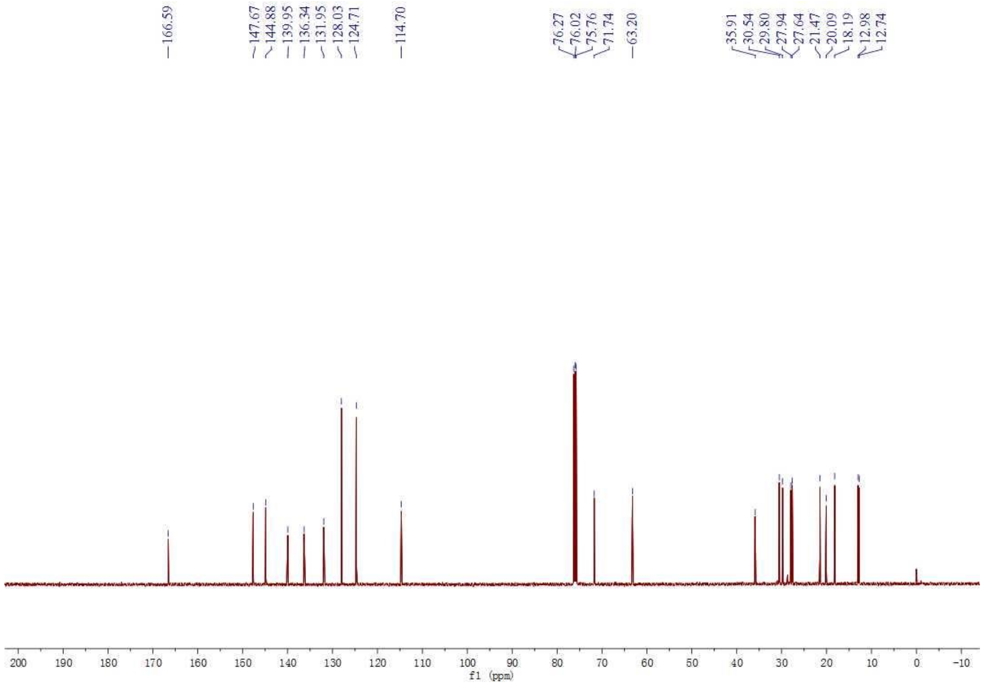


**Supplementary Figure 56.** $^{13}$C NMR spectrum for **3la** in $CDCl_3$

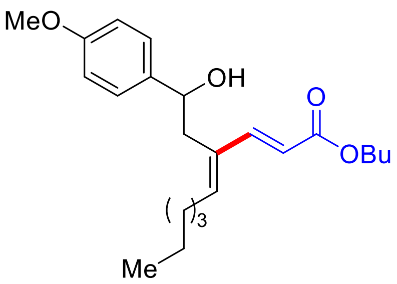


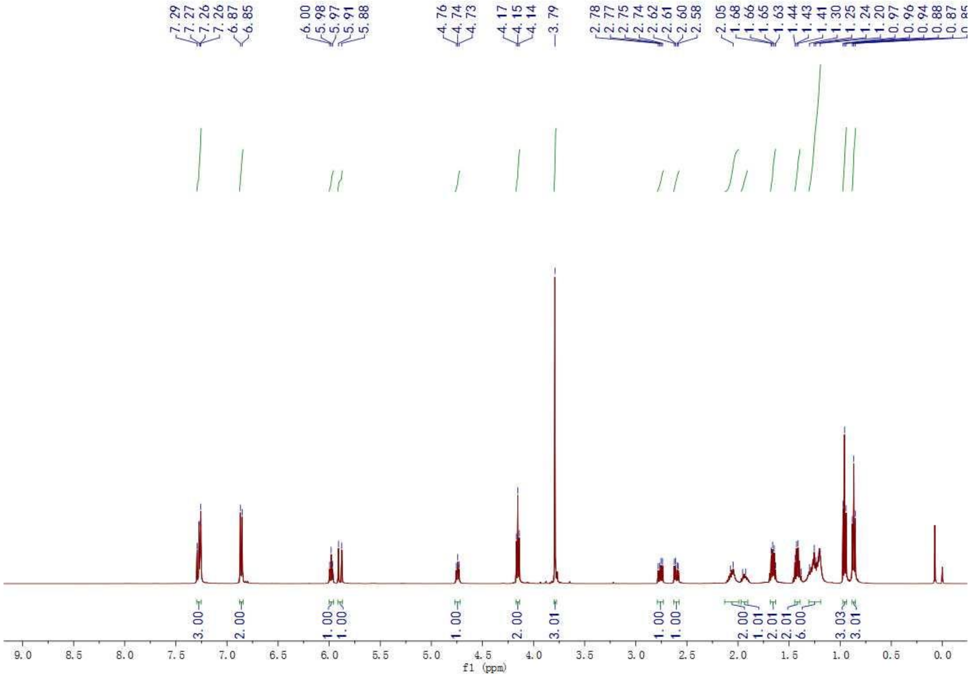


Supplementary Figure 57. $^{1}$H NMR spectrum for **3ma** in $CDCl_3$

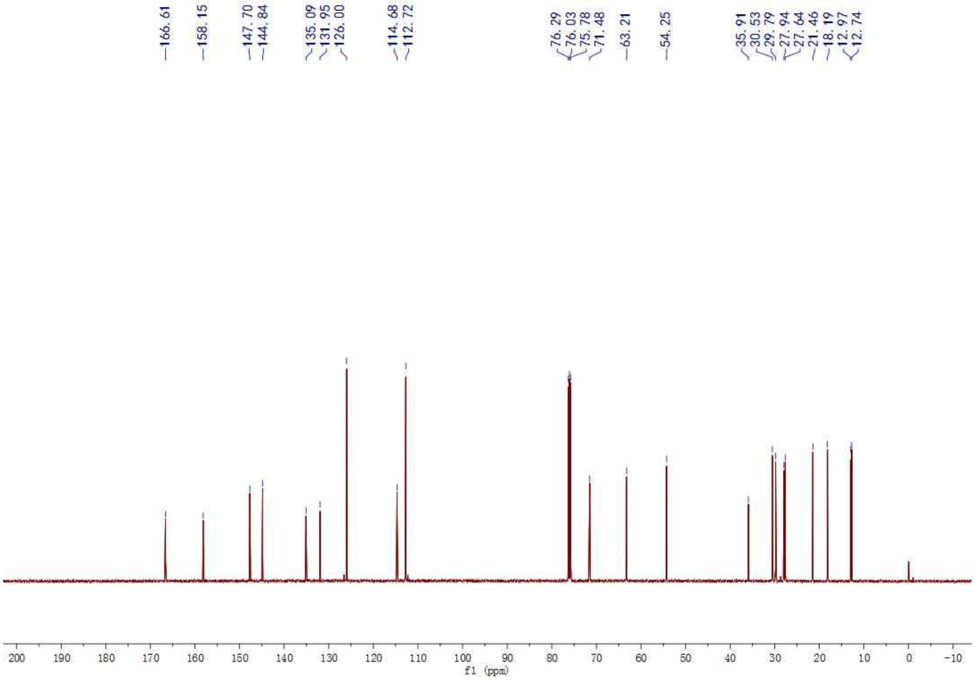


**Supplementary Figure 58.** $^{13}$C NMR spectrum for **3ma** in $CDCl_3$

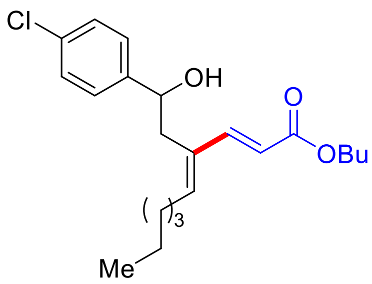


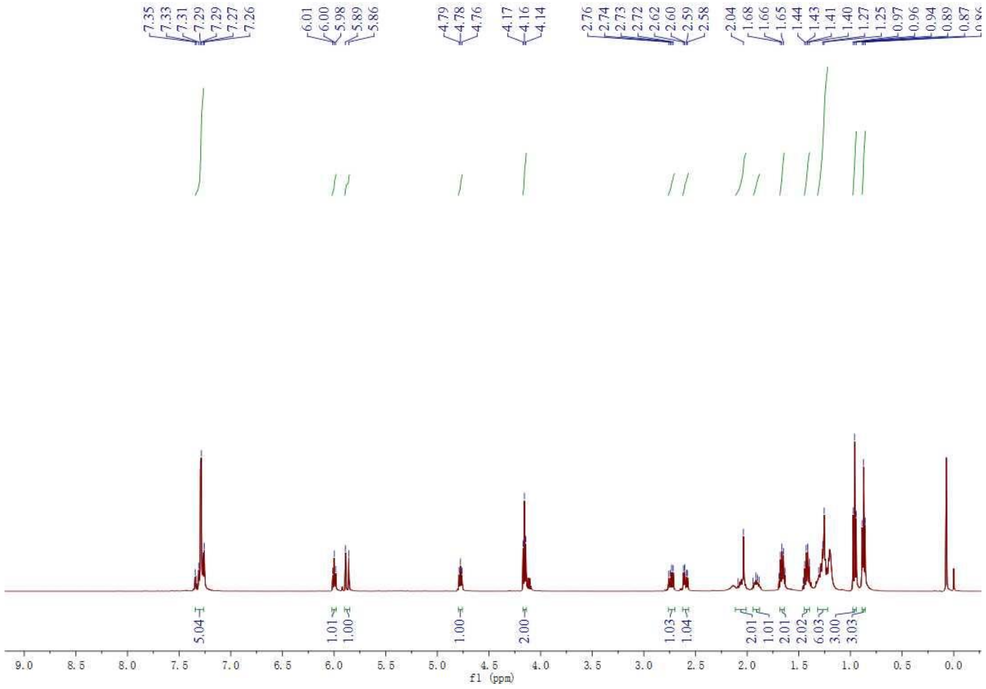

**Supplementary Figure 59.** $^{1}$H NMR spectrum for **3na** in $CDCl_3$

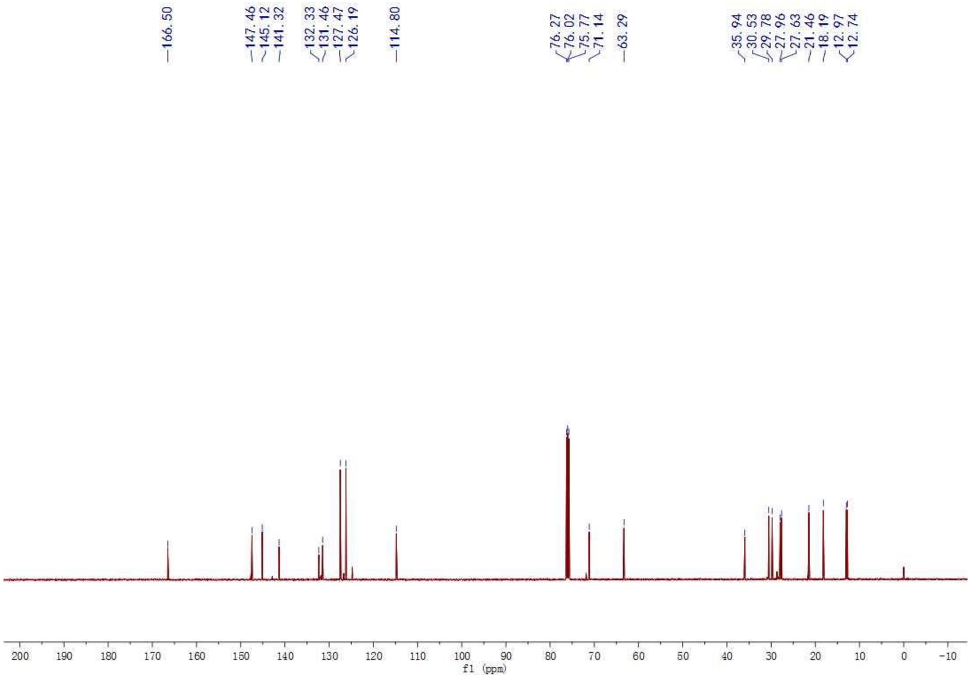

**Supplementary Figure 60.** $^{13}$C NMR spectrum for **3na** in $CDCl_3$

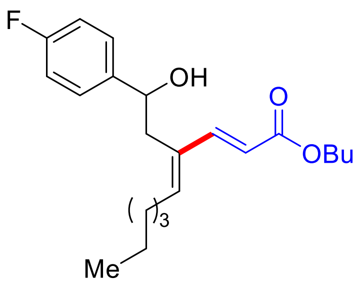


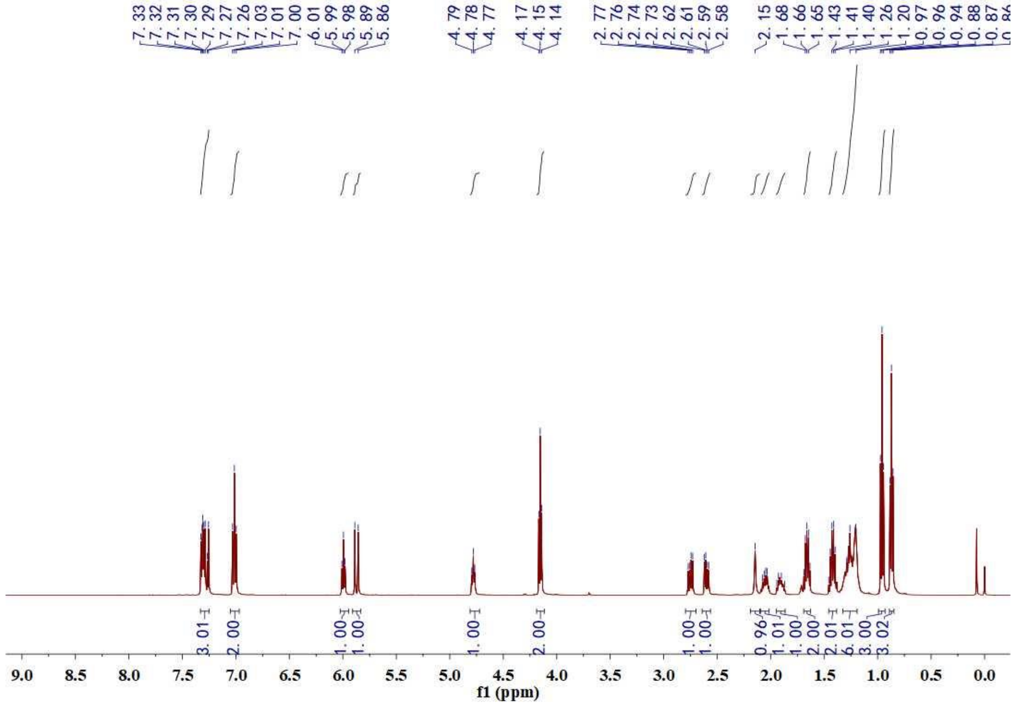


**Supplementary Figure 61.** $^{1}$H NMR spectrum for **3oa** in $CDCl_3$

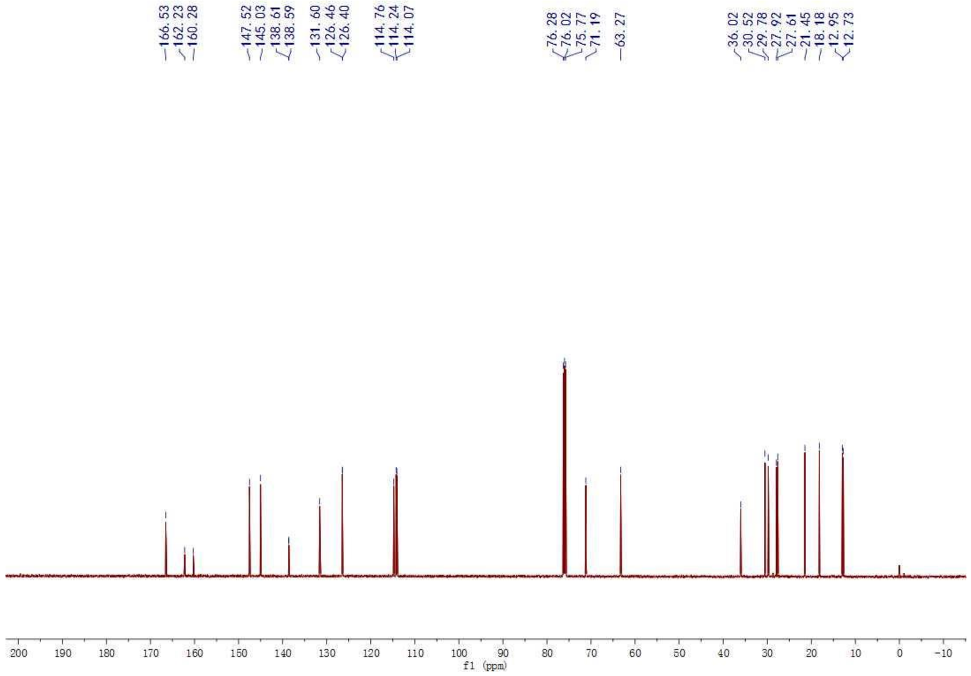


**Supplementary Figure 62.** $^{13}$C NMR spectrum for **3oa** in $CDCl_3$

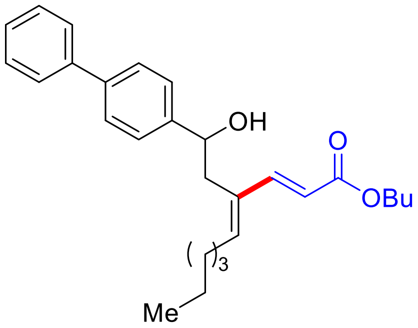


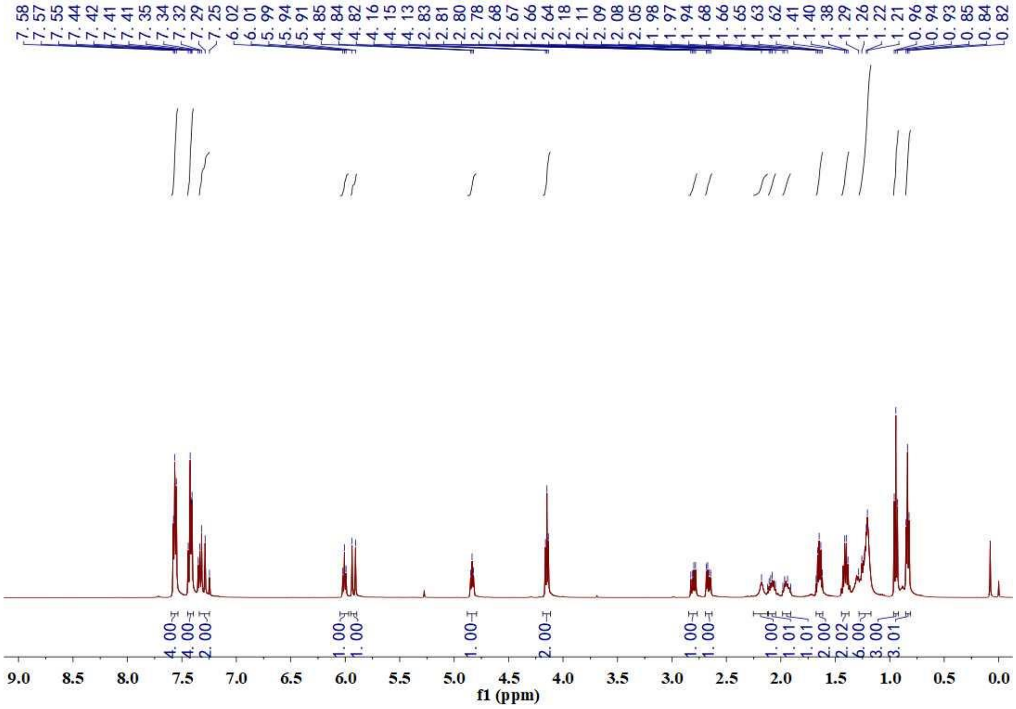

**Supplementary Figure 63.** $^{1}H$ NMR spectrum for **3pa** in $CDCl_3$

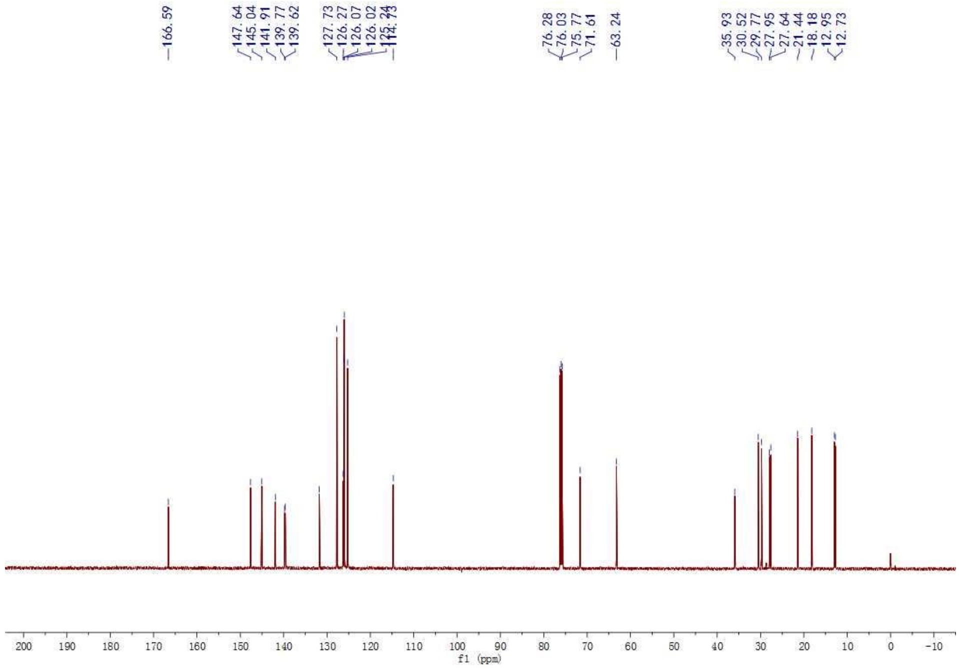

**Supplementary Figure 64.** $^{13}C$ NMR spectrum for **3pa** in $CDCl_3$

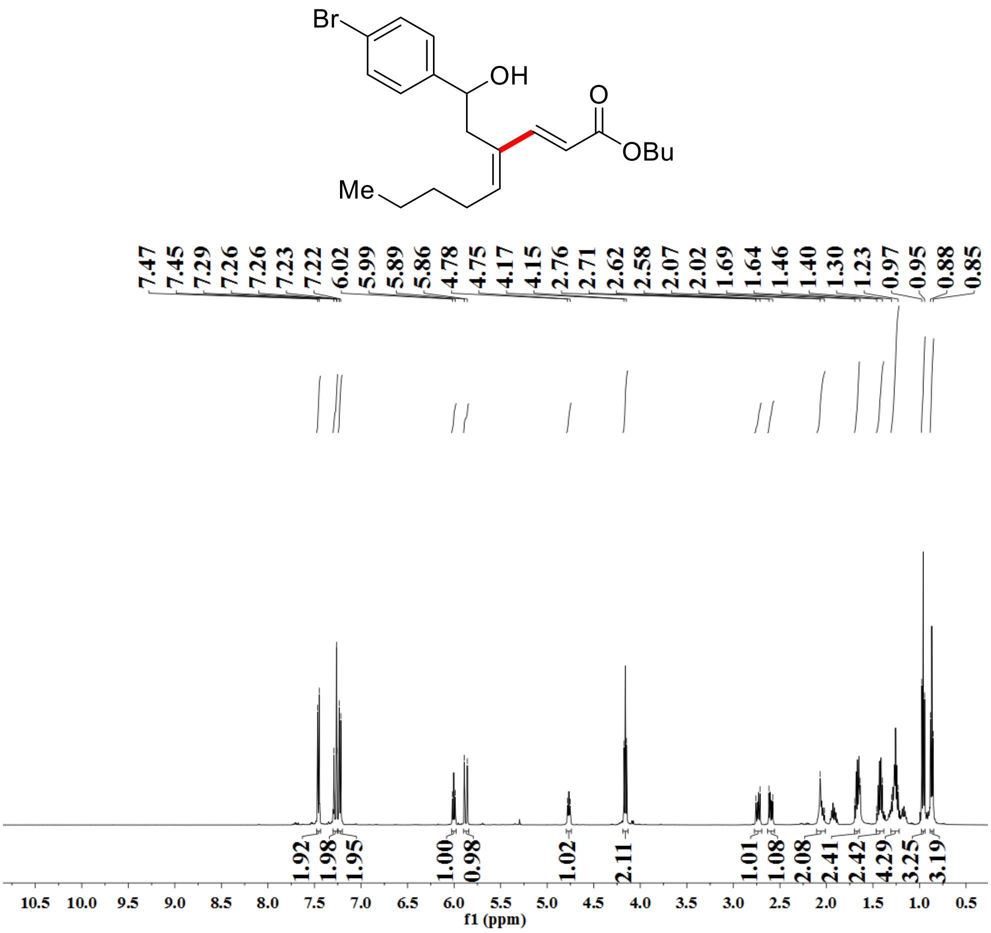


**Supplementary Figure 65.** $^{1}$H NMR spectrum for **3qa** in $CDCl_3$

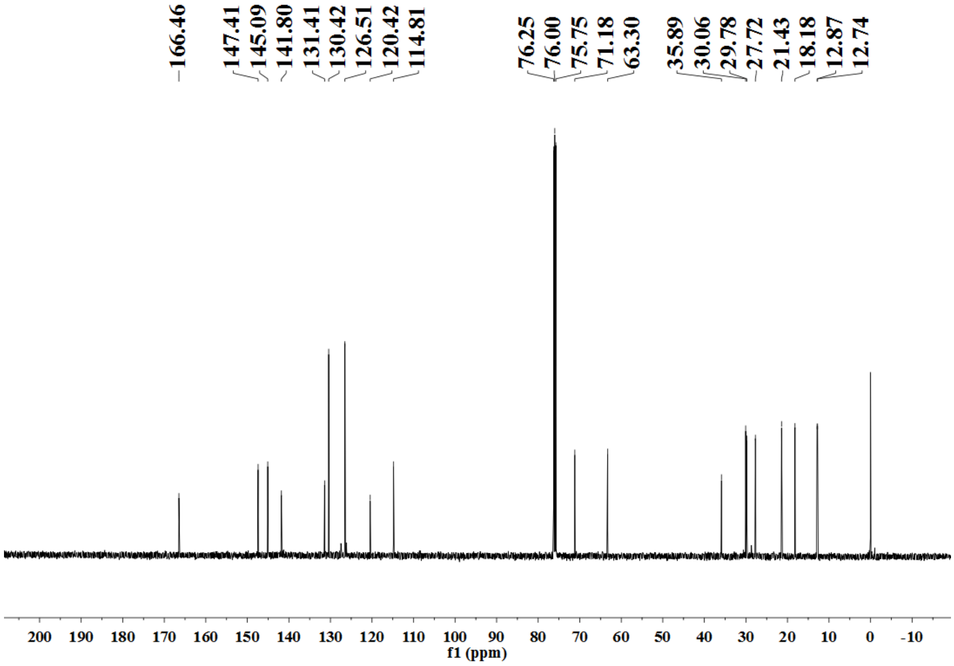


**Supplementary Figure 66.** $^{13}$C NMR spectrum for **3qa** in $CDCl_3$

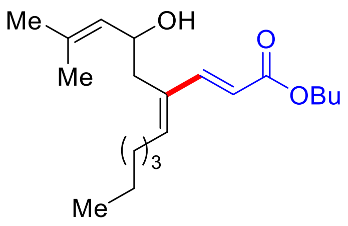


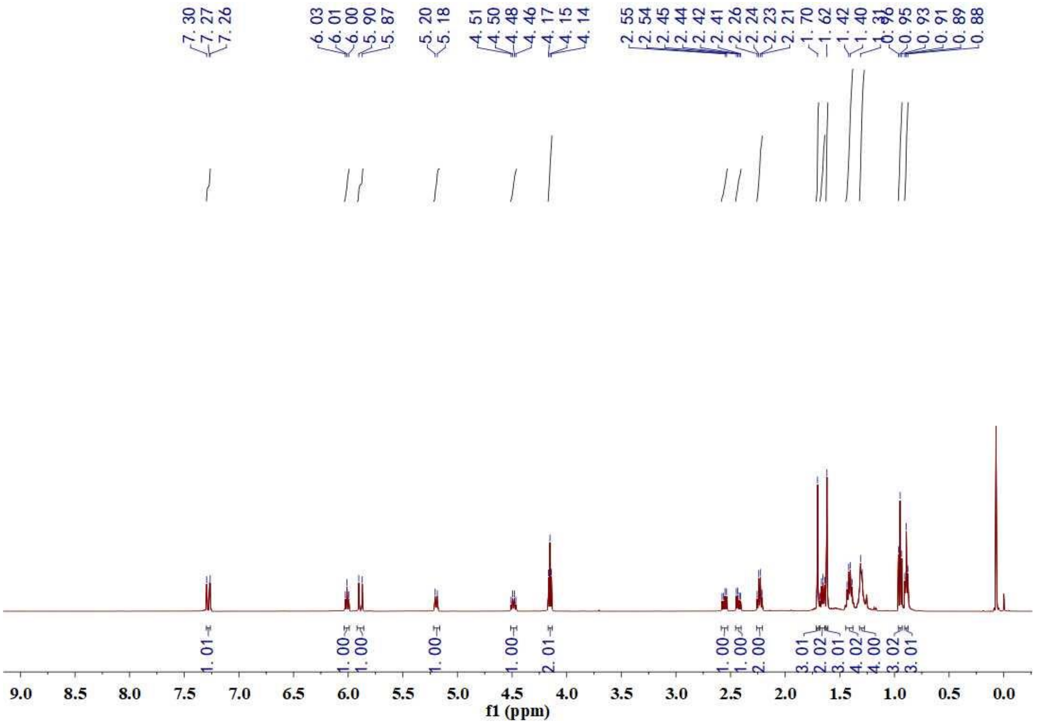


**Supplementary Figure 67.** $^{1}$H NMR spectrum for **3ra** in $CDCl_3$

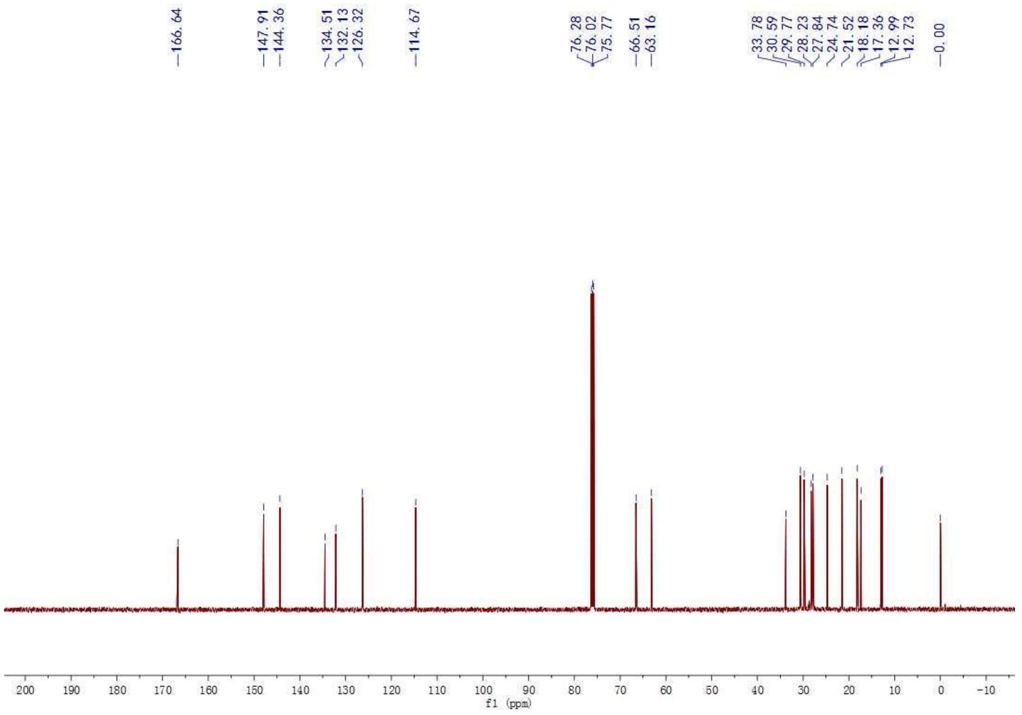


**Supplementary Figure 68.** $^{13}$C NMR spectrum for **3ra** in $CDCl_3$

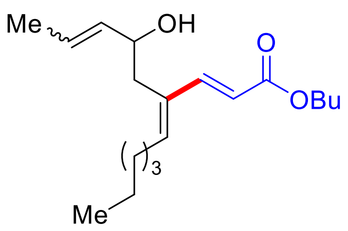


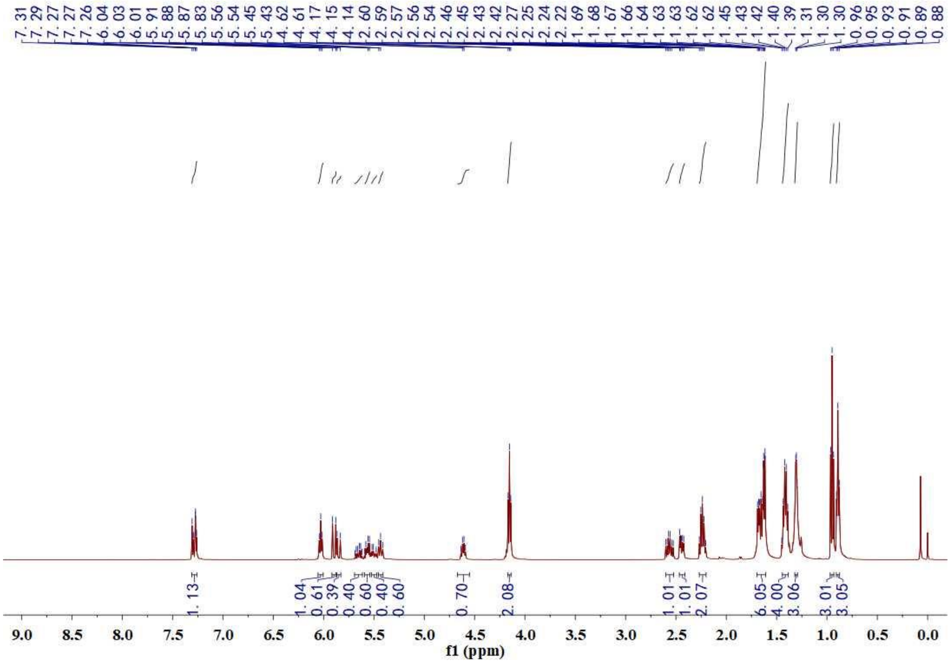

**Supplementary Figure 69.** $^{1}H$ NMR spectrum for **3sa** in $CDCl_3$

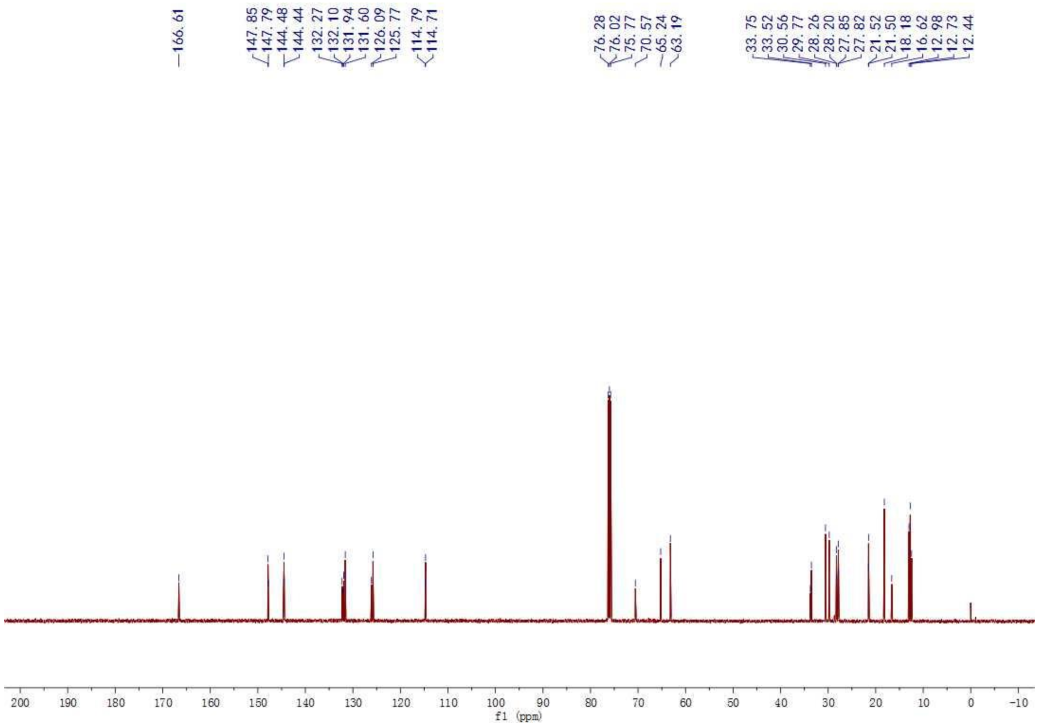

**Supplementary Figure 70.** $^{13}C$ NMR spectrum for **3sa** in $CDCl_3$

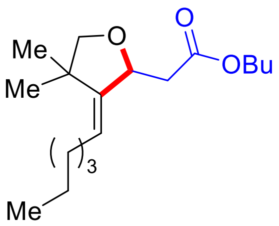


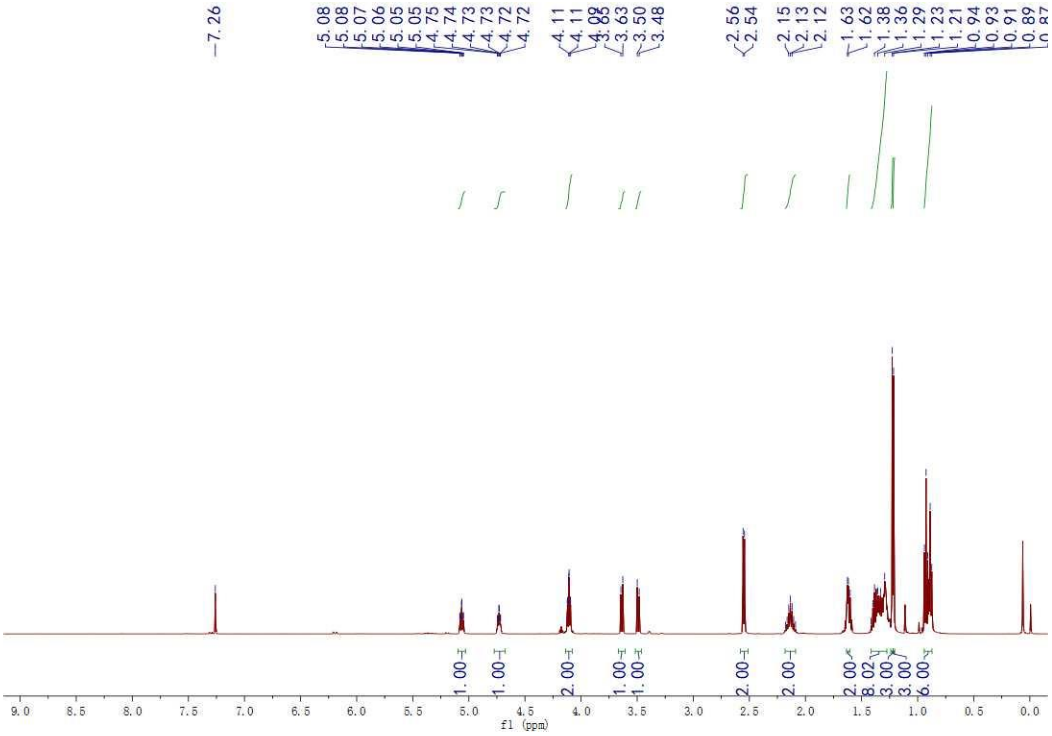

**Supplementary Figure 71.** $^{1}$H NMR spectrum for **3A** in $CDCl_3$

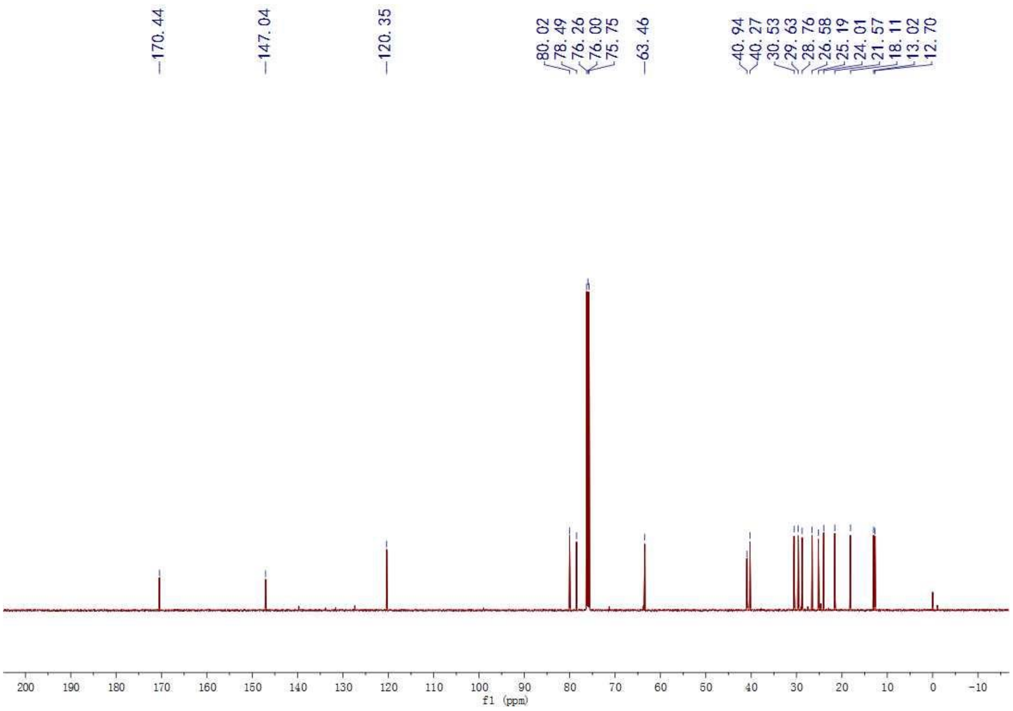

**Supplementary Figure 72.** $^{13}$C NMR spectrum for **3A** in $CDCl_3$

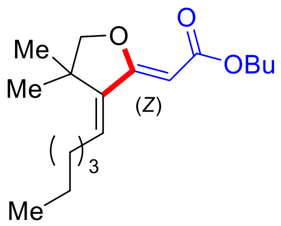


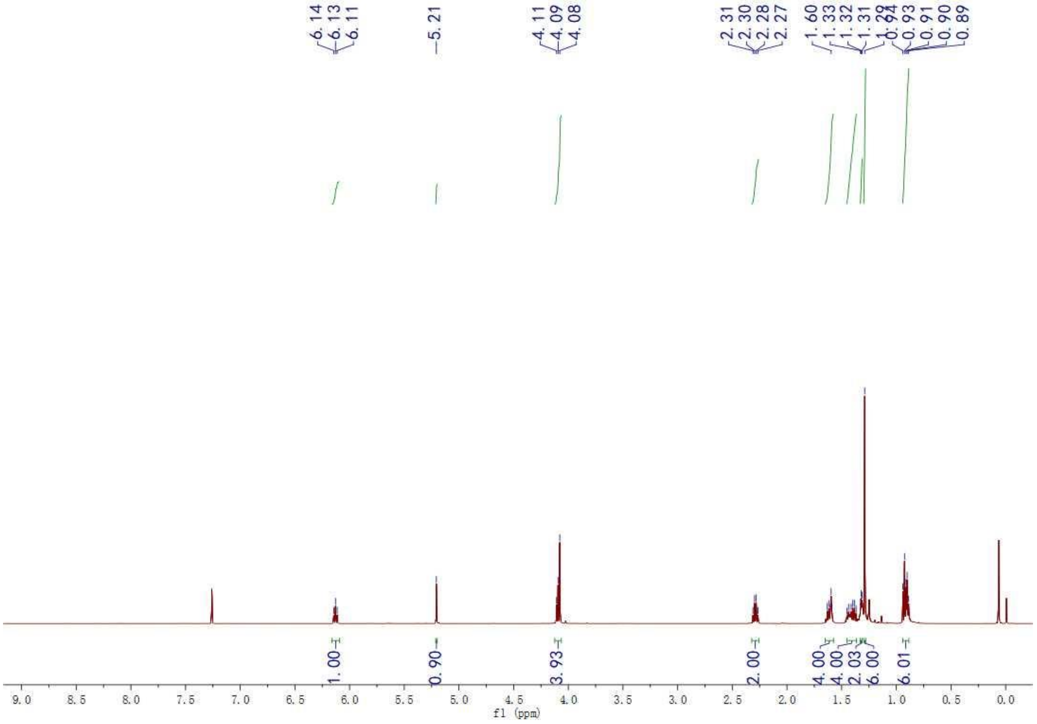


**Supplementary Figure 73.** $^{1}$H NMR spectrum for **3A′** in $CDCl_3$

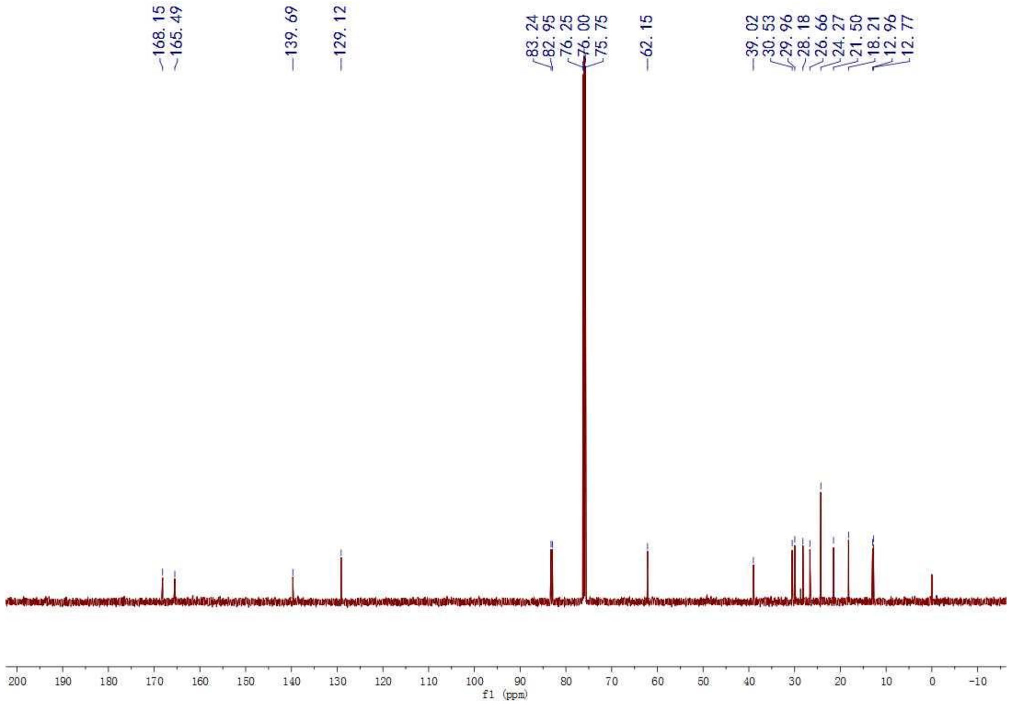


**Supplementary Figure 74.** $^{13}$C NMR spectrum for **3A′** in $CDCl_3$

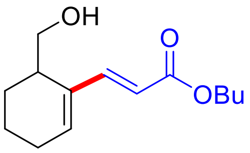


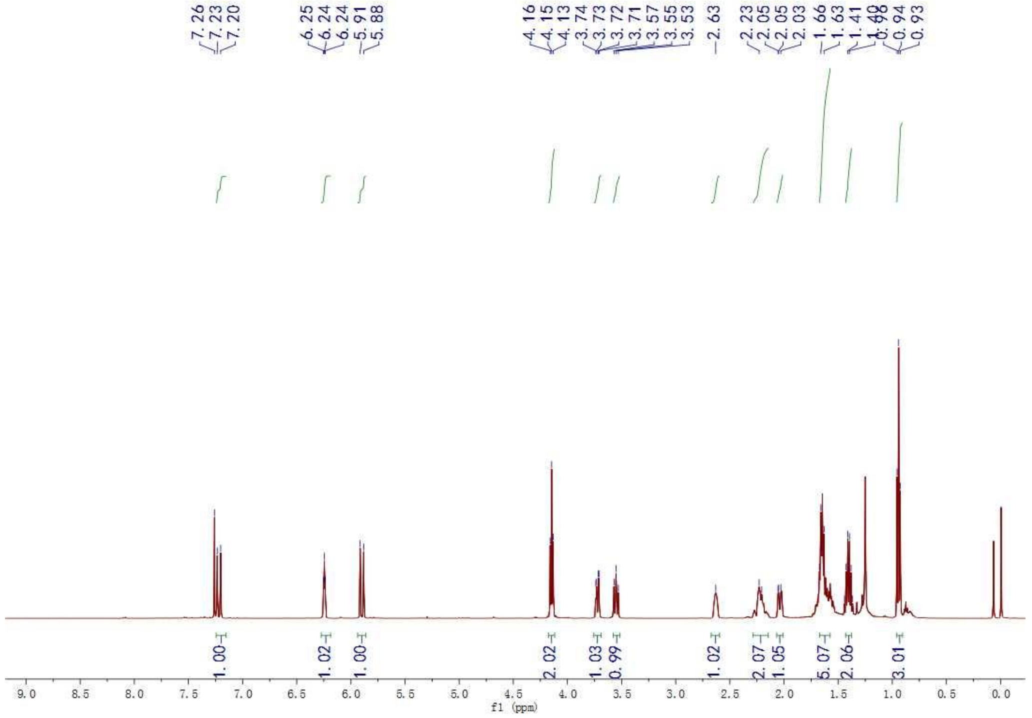


**Supplementary Figure 75.** $^{1}$H NMR spectrum for **3ta** in $CDCl_3$

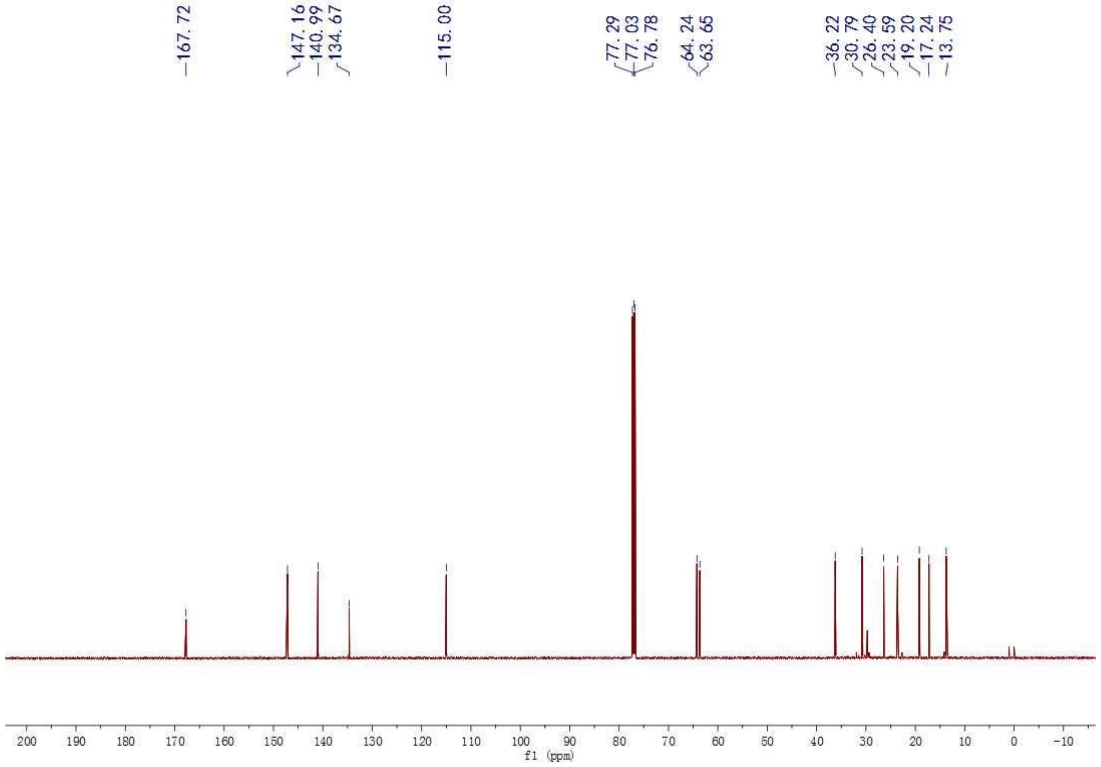


**Supplementary Figure 76.** $^{13}$C NMR spectrum for **3ta** in $CDCl_3$

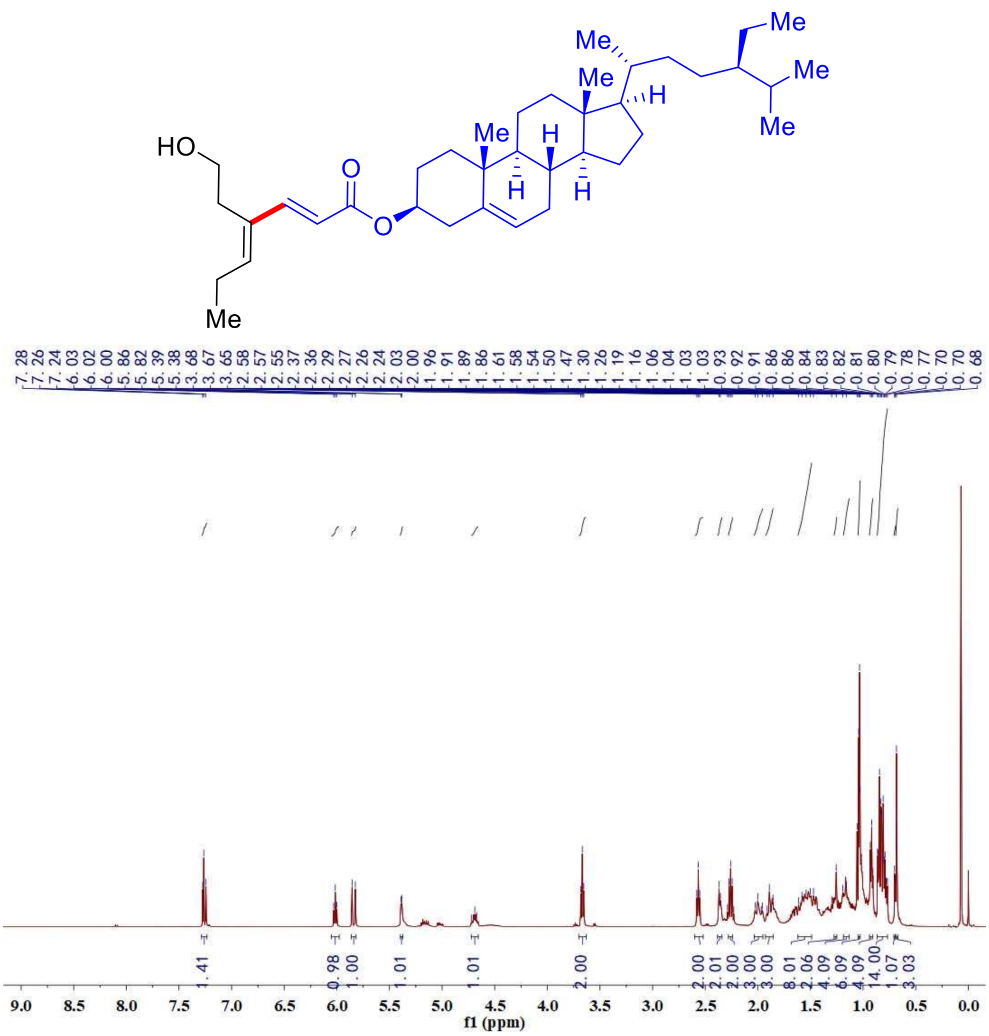


**Supplementary Figure 77.** $^{1}$H NMR spectrum for **3ap** in $CDCl_3$

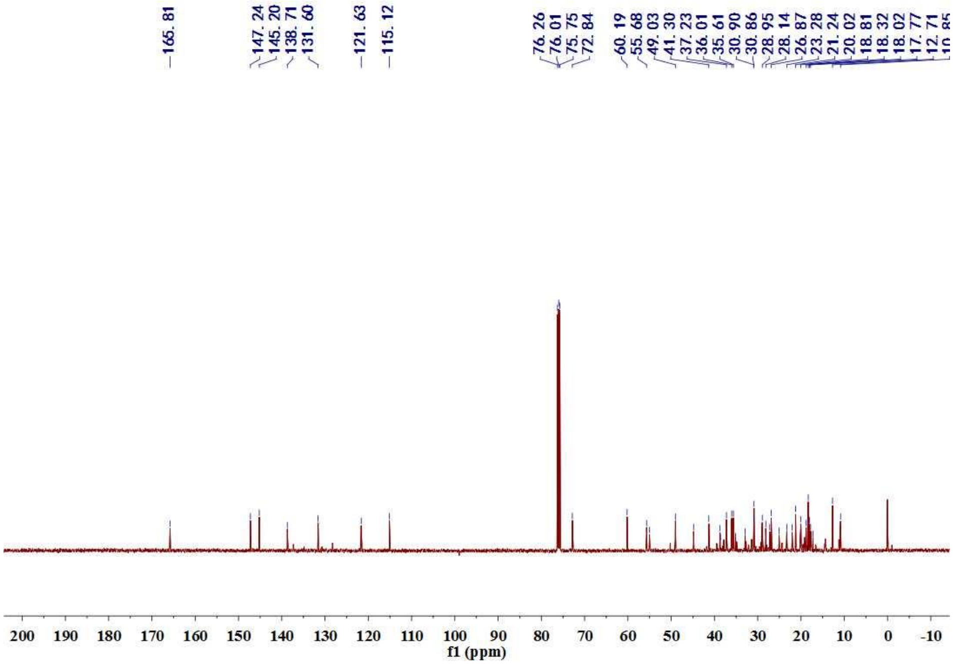


**Supplementary Figure 78.** $^{13}$C NMR spectrum for **3ap** in $CDCl_3$

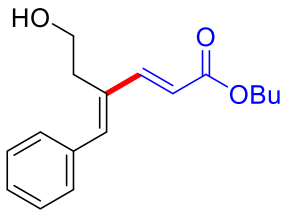


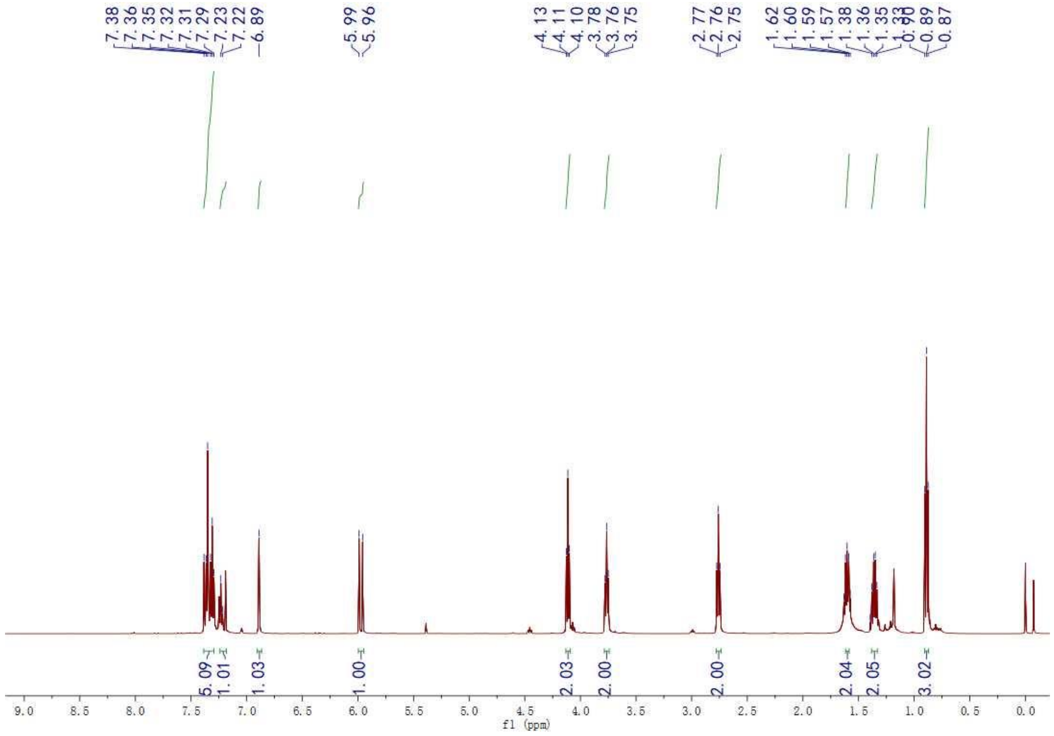


**Supplementary Figure 79.** $^{1}$H NMR spectrum for **3ua** in $CDCl_3$

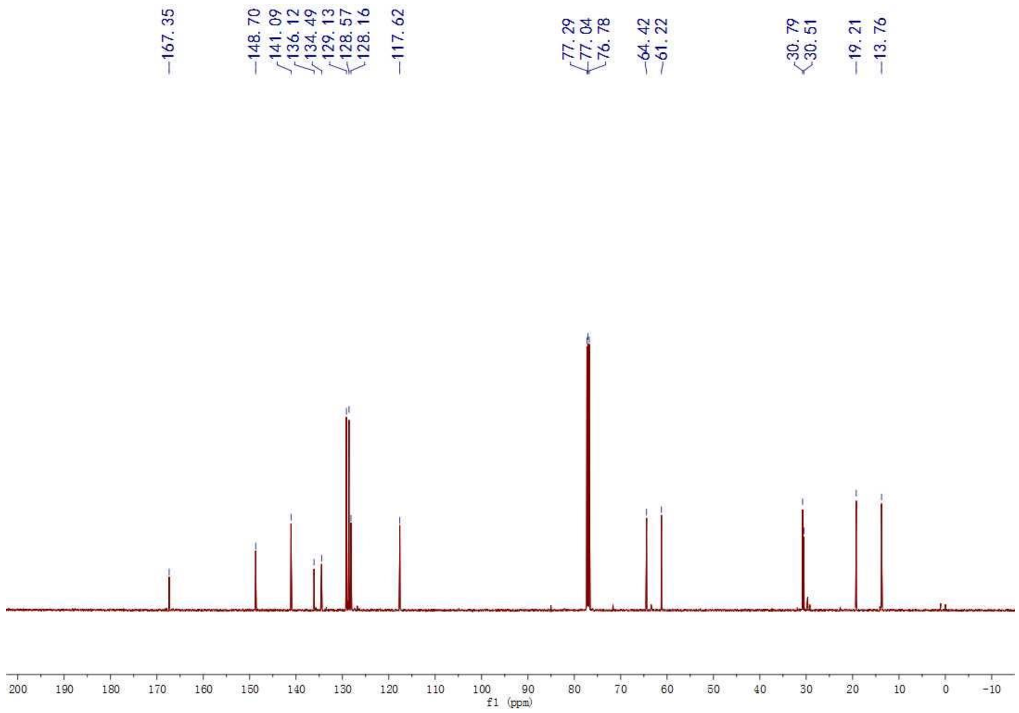


**Supplementary Figure 80.** $^{13}$C NMR spectrum for **3ua** in $CDCl_3$

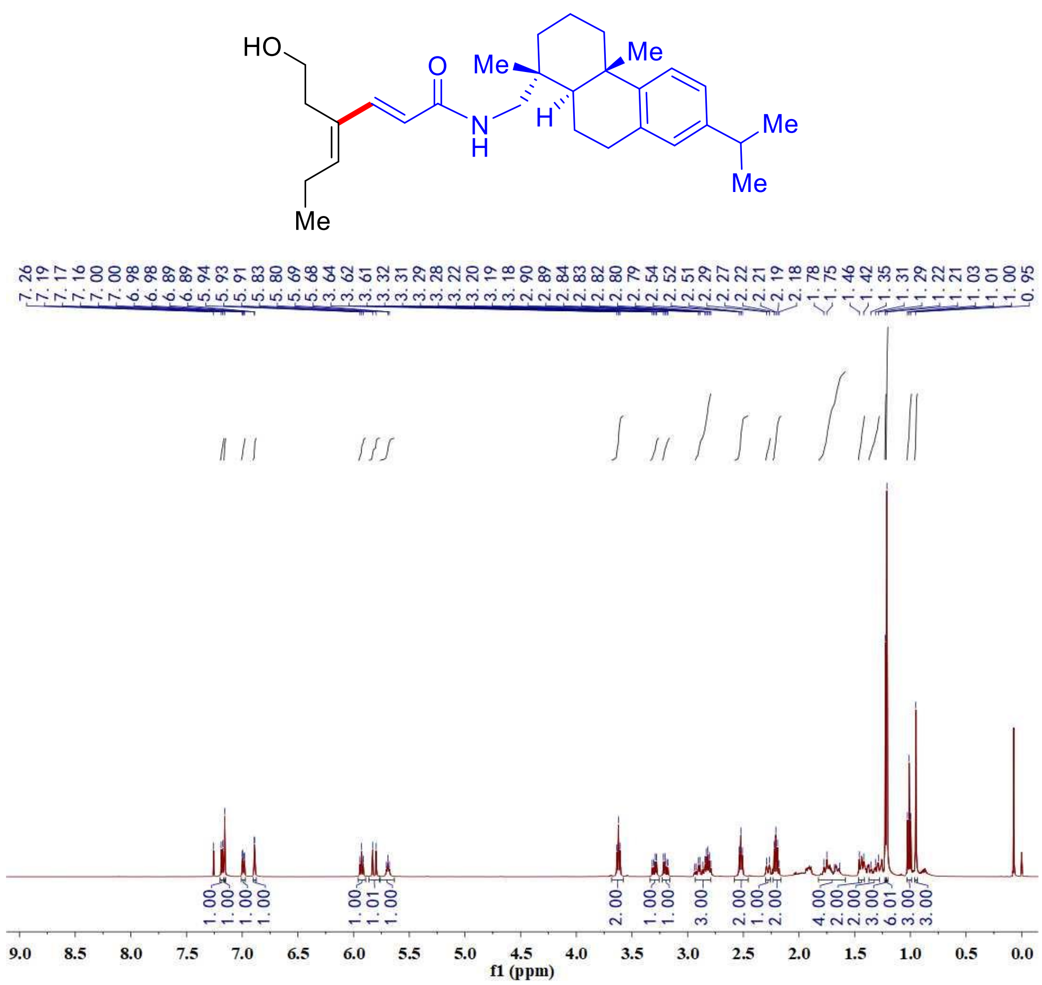


**Supplementary Figure 81.** $^{1}$H NMR spectrum for **3aq** in $CDCl_3$

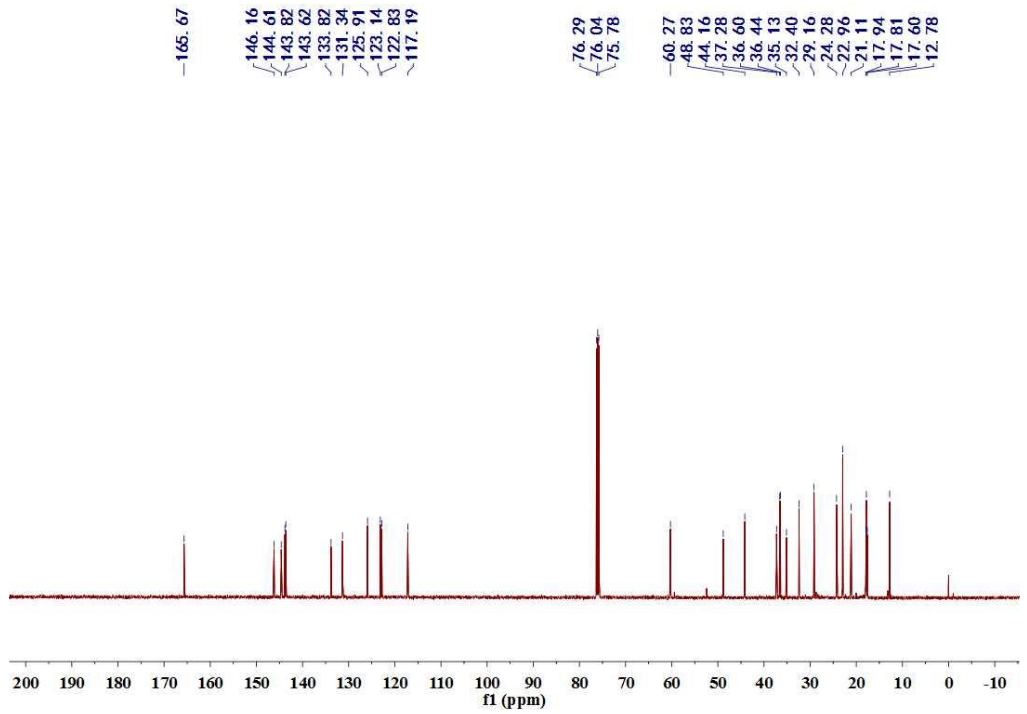


**Supplementary Figure 82.** $^{13}$C NMR spectrum for **3aq** in $CDCl_3$

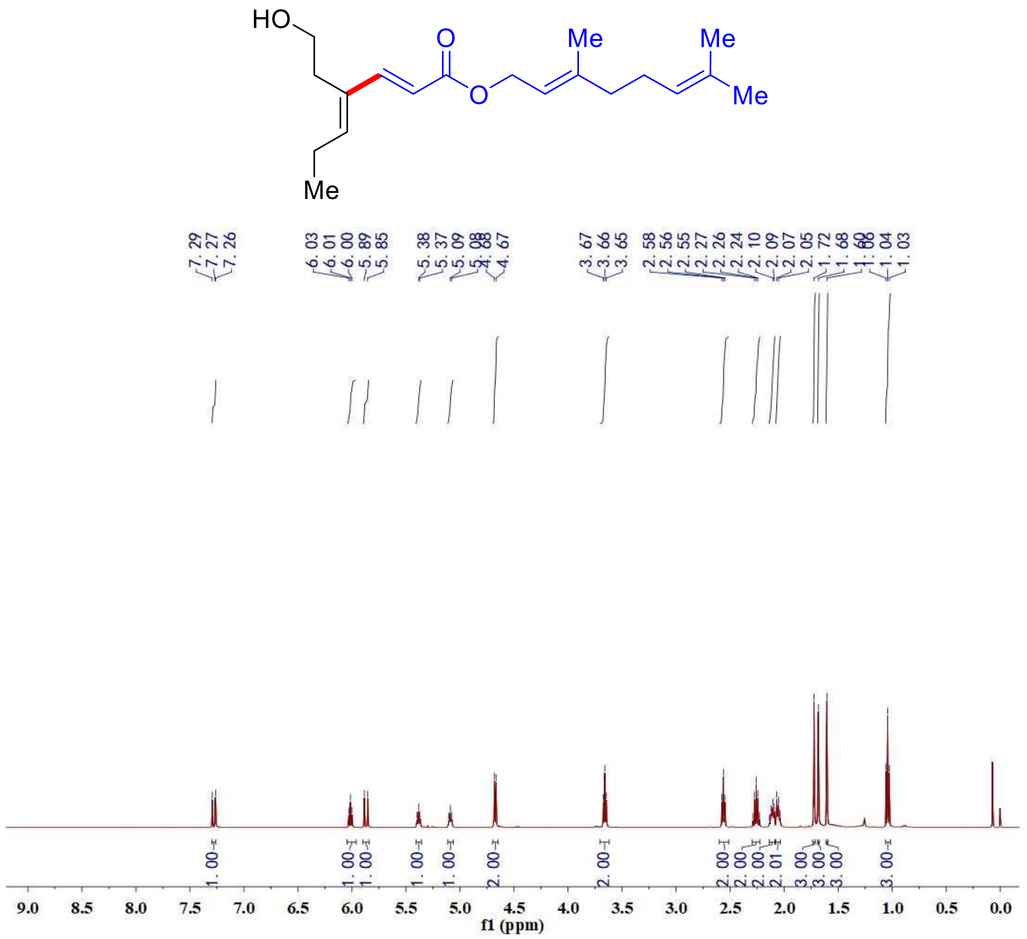


**Supplementary Figure 83.** $^{1}$H NMR spectrum for **3ar** in $CDCl_3$

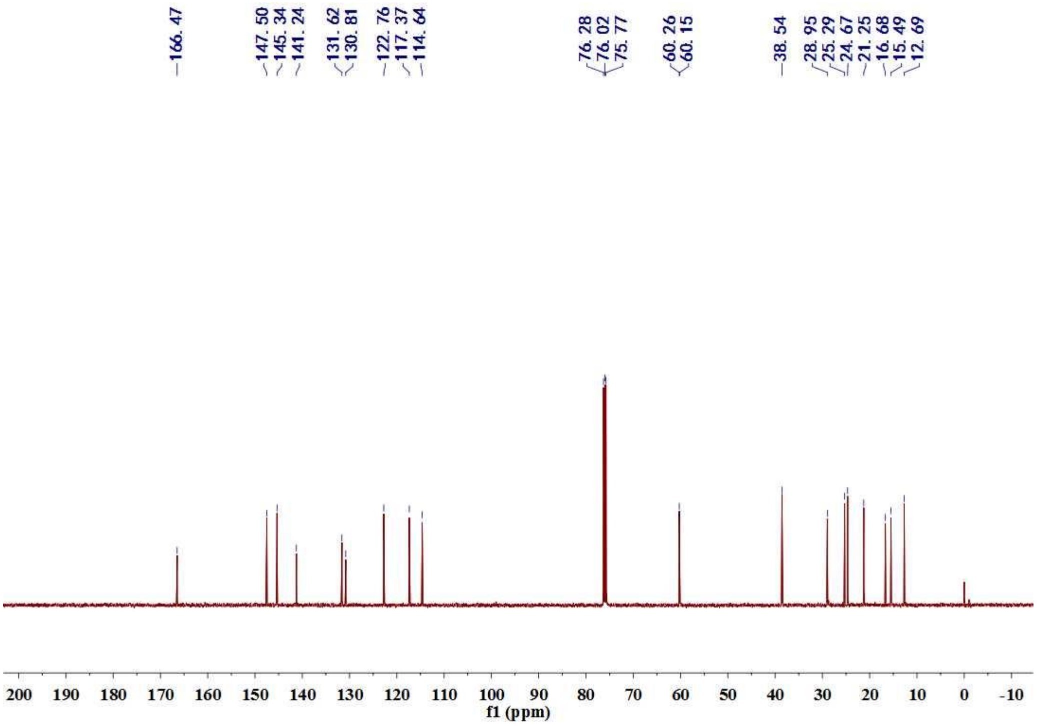


**Supplementary Figure 84.** $^{13}$C NMR spectrum for **3ar** in $CDCl_3$

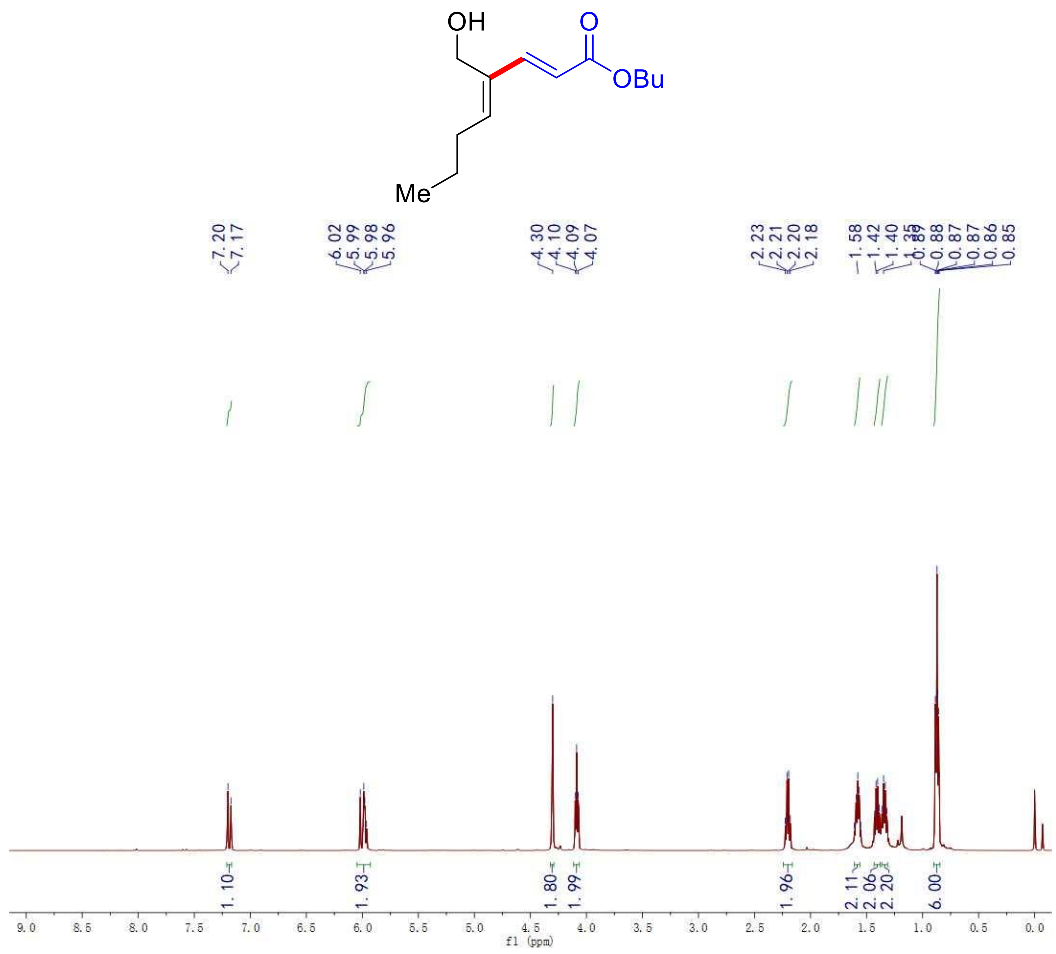


**Supplementary Figure 85.** $^{1}$H NMR spectrum for **3va** in $CDCl_3$

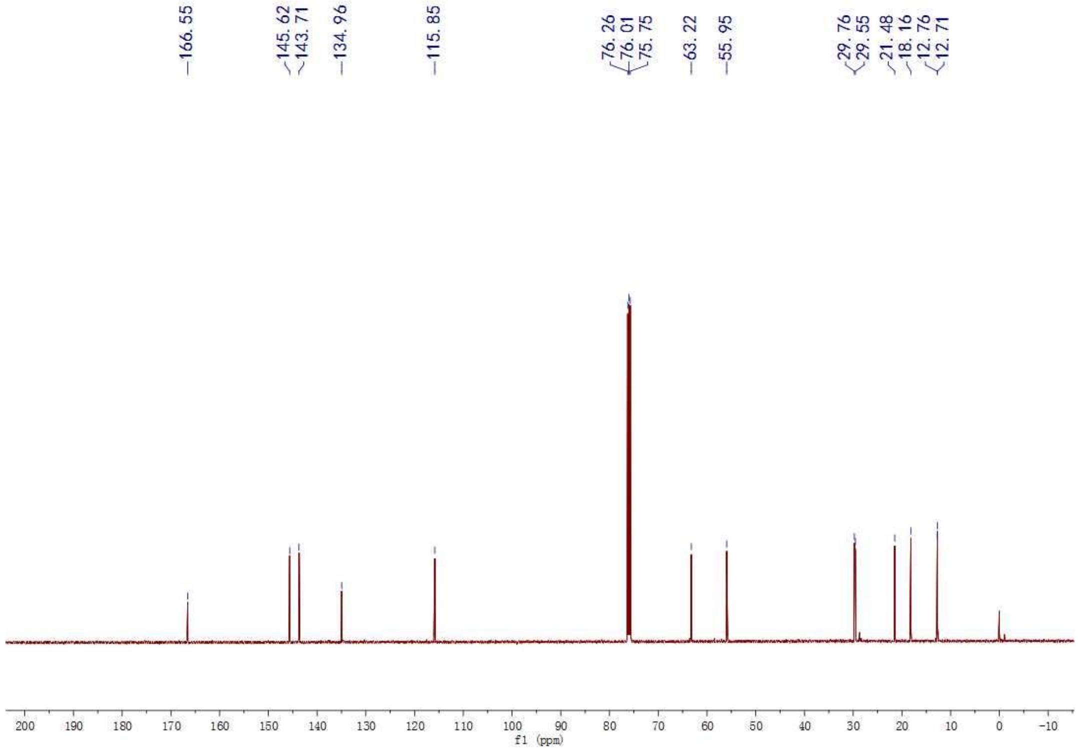


**Supplementary Figure 86.** $^{13}$C NMR spectrum for **3va** in $CDCl_3$

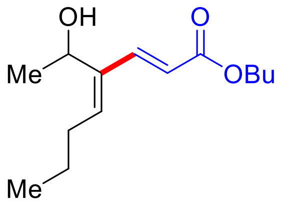


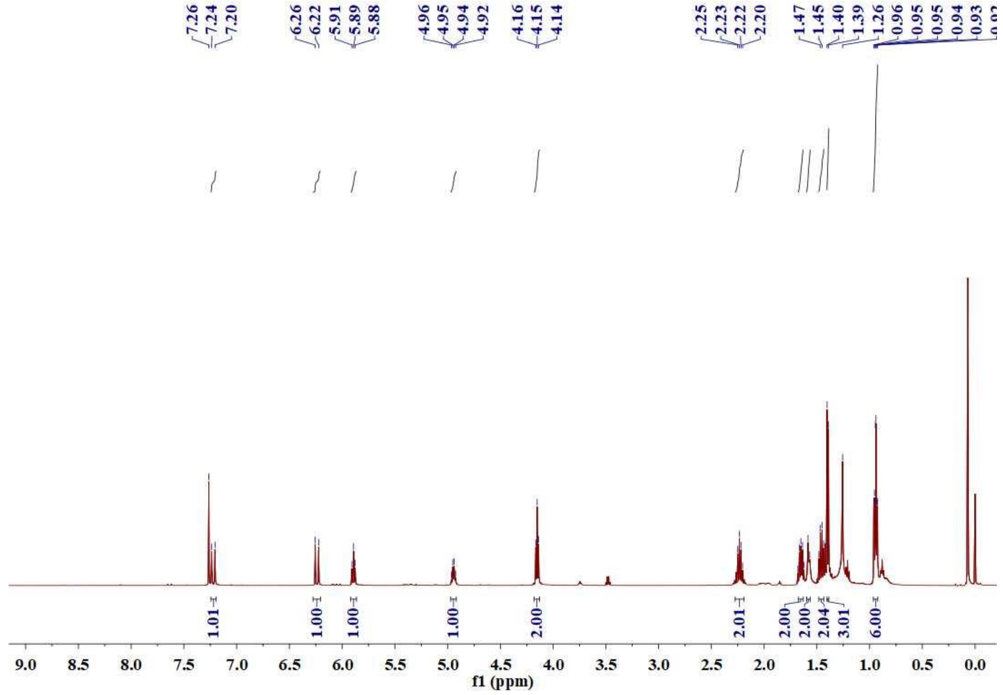


**Supplementary Figure 87.** $^{1}$H NMR spectrum for **3wa** in $CDCl_3$

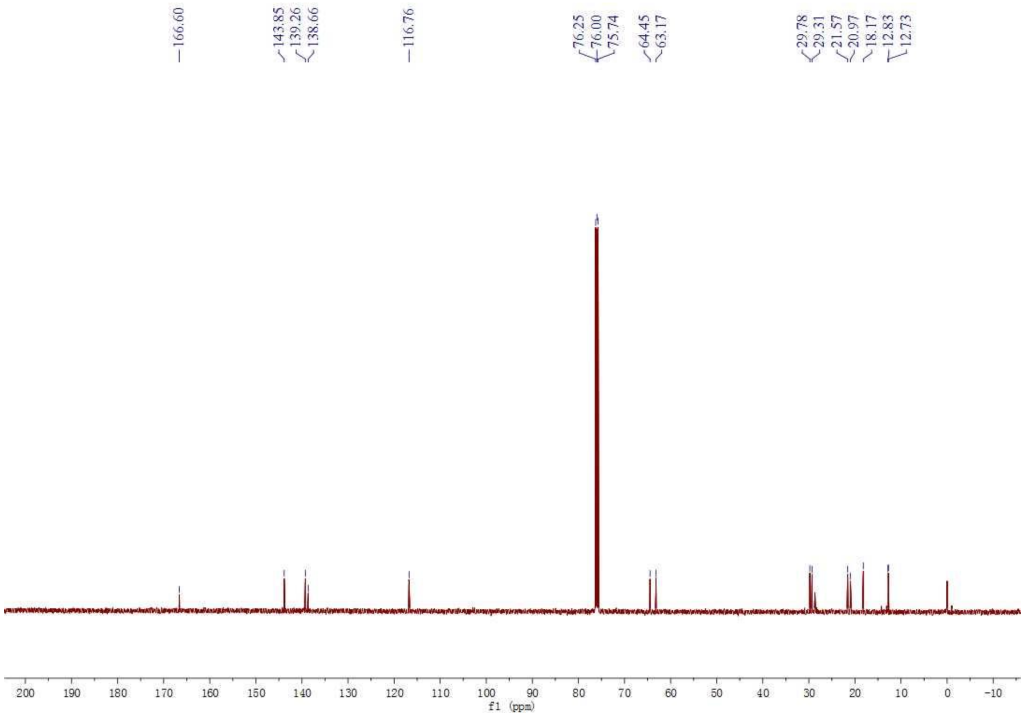


**Supplementary Figure 88.** $^{13}$C NMR spectrum for **3wa** in $CDCl_3$

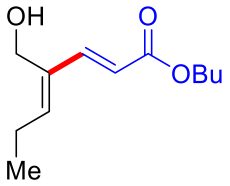


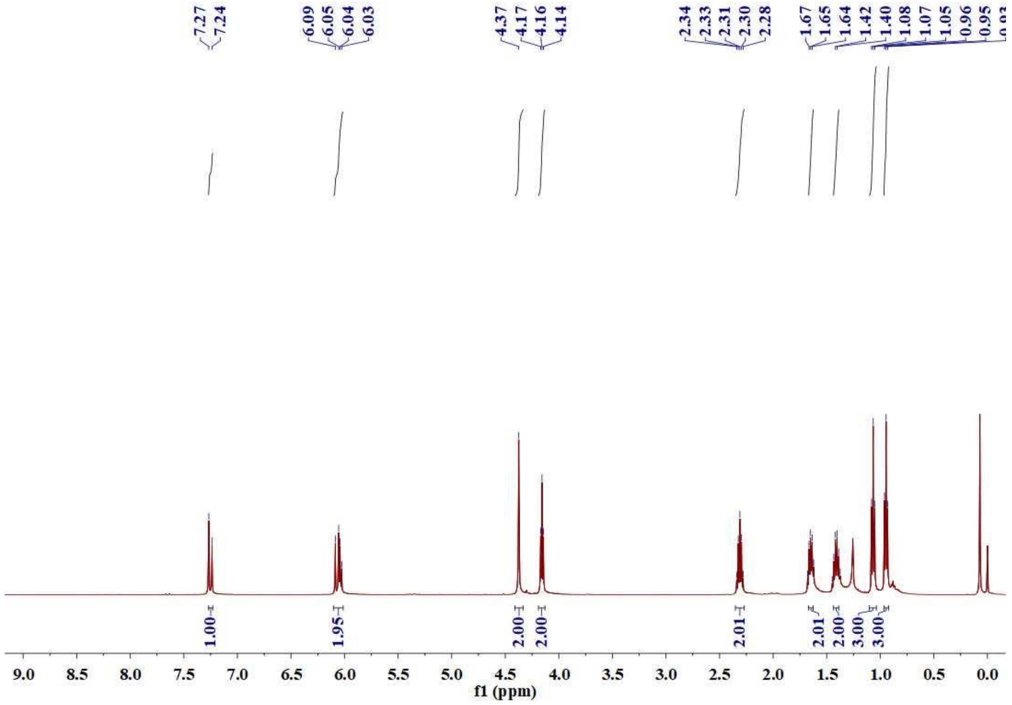


**Supplementary Figure 89.** $^{1}$H NMR spectrum for **3xa** in $CDCl_3$

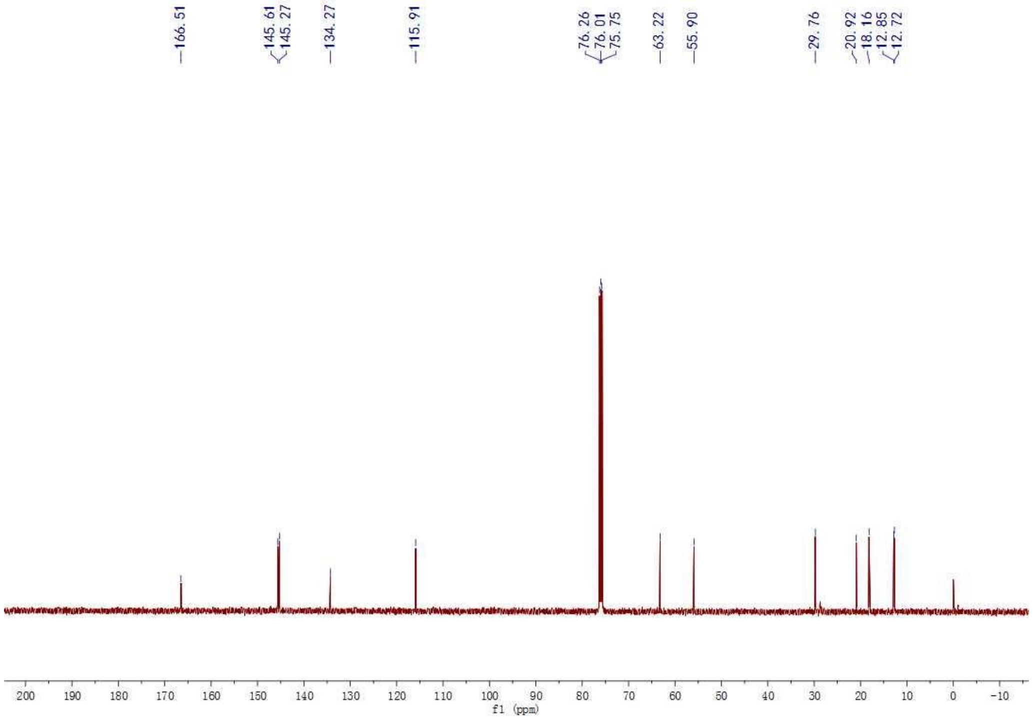


**Supplementary Figure 90.** $^{13}$C NMR spectrum for **3xa** in $CDCl_3$

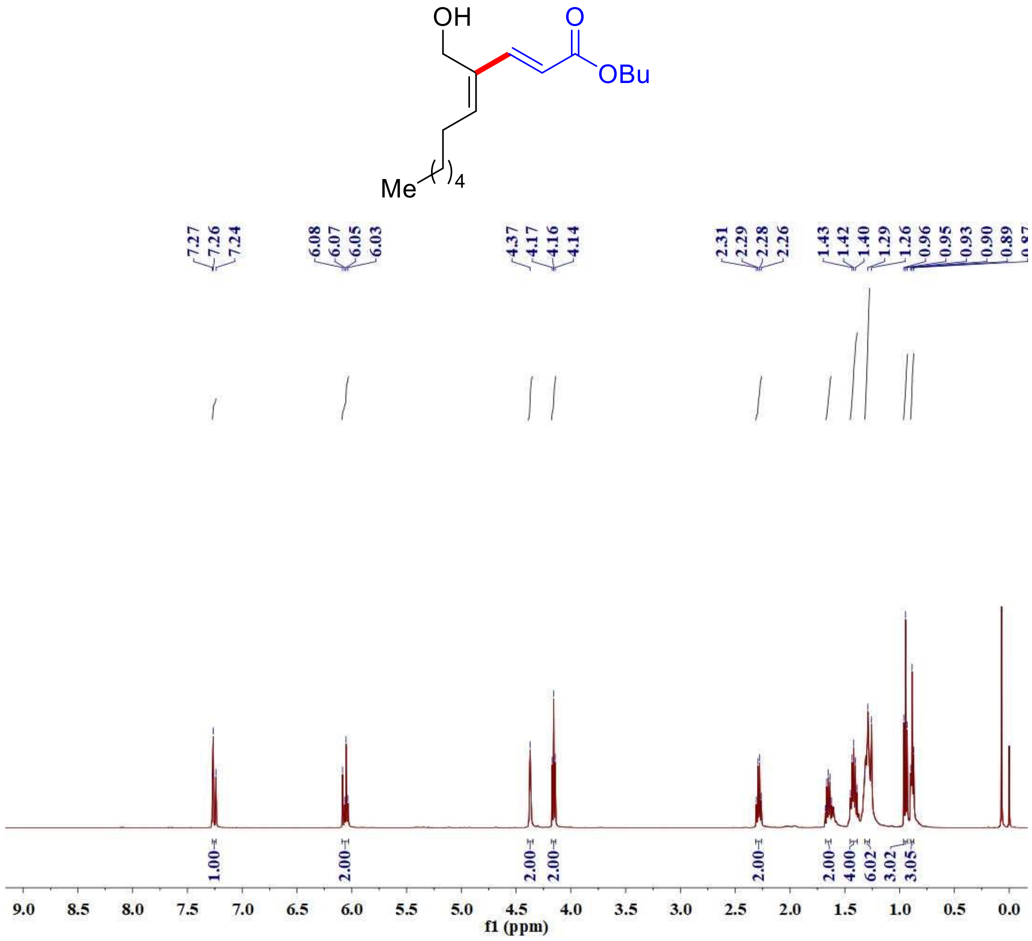


**Supplementary Figure 91.** $^{1}$H NMR spectrum for **3ya** in $CDCl_3$

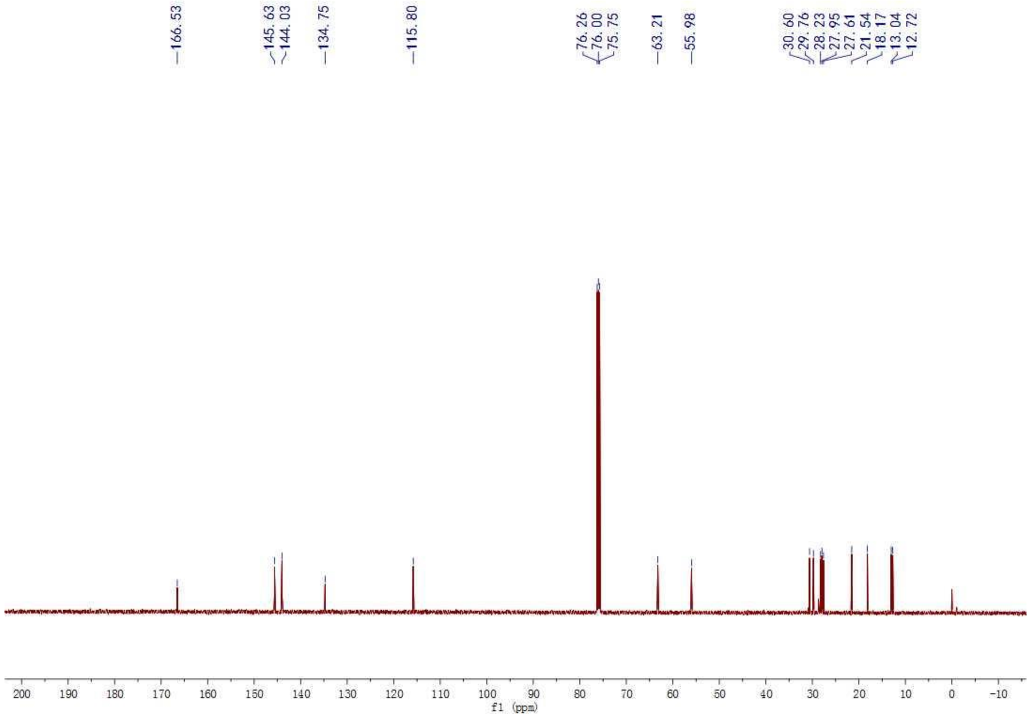


**Supplementary Figure 92.** $^{13}$C NMR spectrum for **3ya** in $CDCl_3$

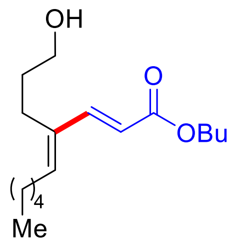


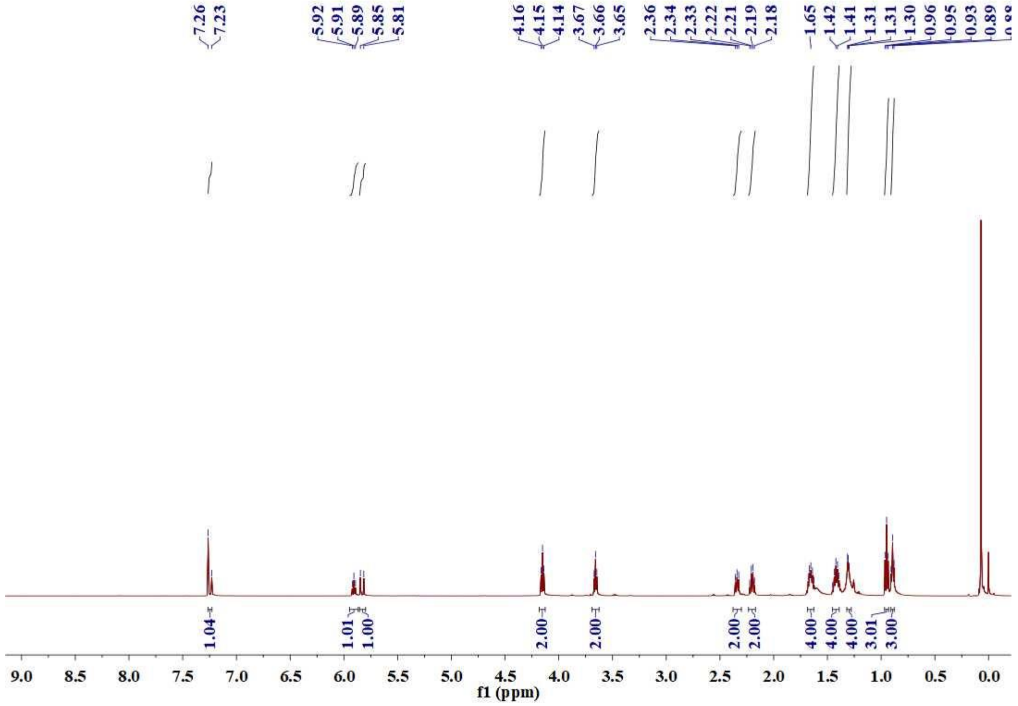


**Supplementary Figure 93.** $^1$H NMR spectrum for **3za** in $CDCl_3$

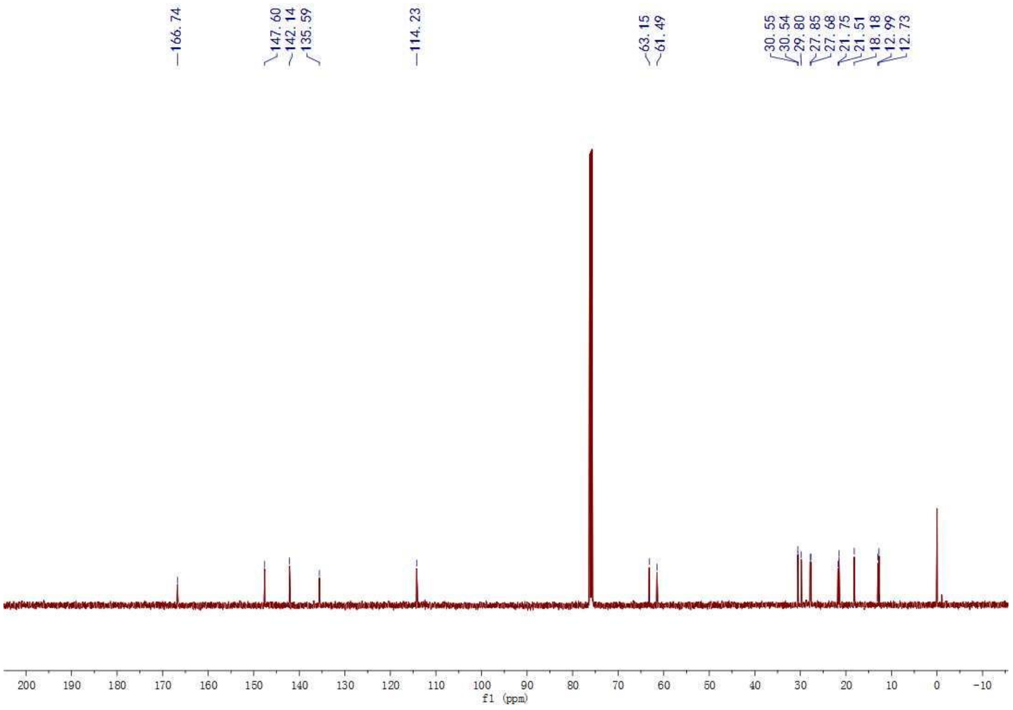


**Supplementary Figure 94.** $^{13}$C NMR spectrum for **3za** in $CDCl_3$

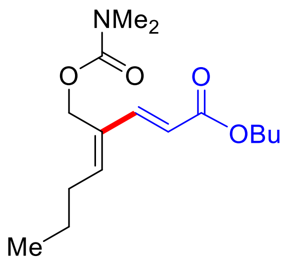


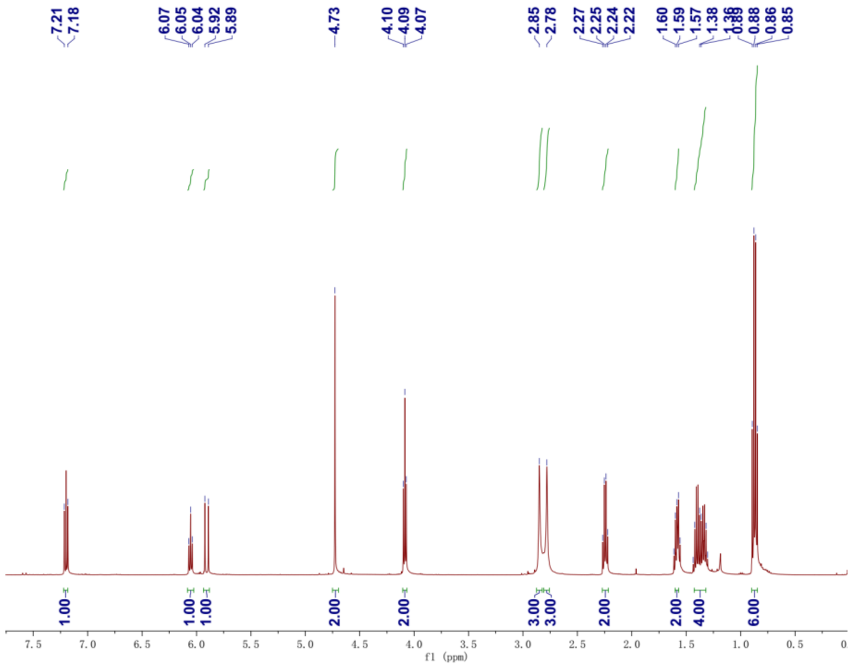


**Supplementary Figure 95.** $^{1}$H NMR spectrum for **5aa** in $CDCl_3$

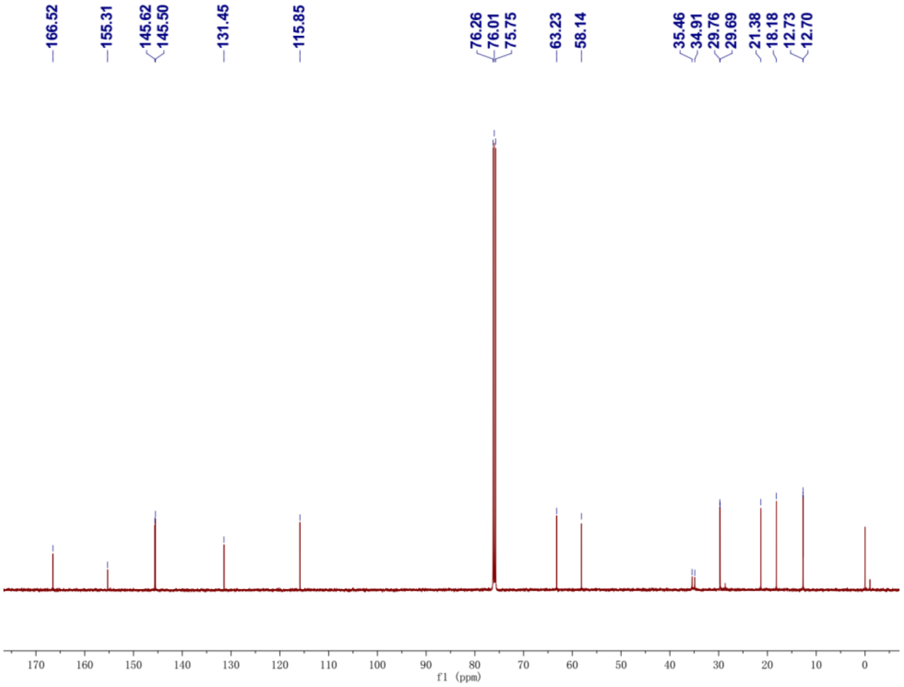


**Supplementary Figure 96.** $^{13}$C NMR spectrum for **5aa** in $CDCl_3$

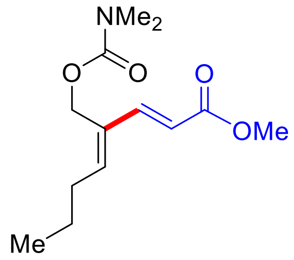


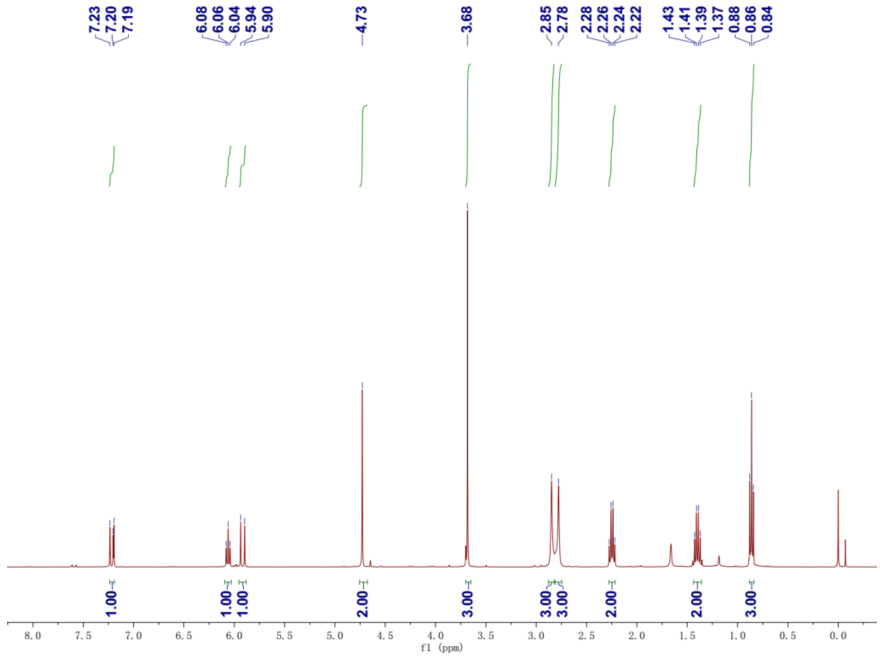

**Supplementary Figure 97.** $^{1}$H NMR spectrum for **5ab** in $CDCl_3$

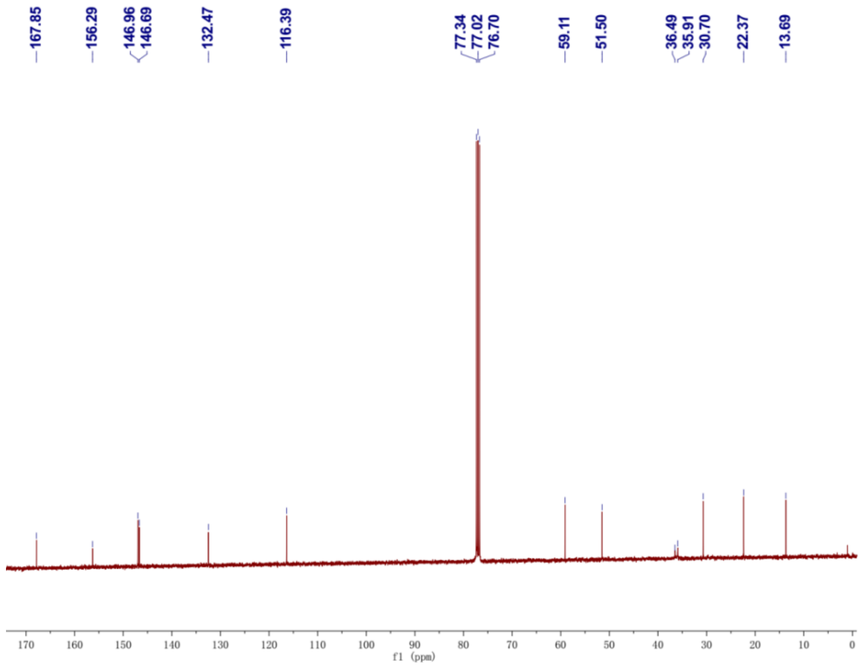

**Supplementary Figure 98.** $^{13}$C NMR spectrum for **5ab** in $CDCl_3$

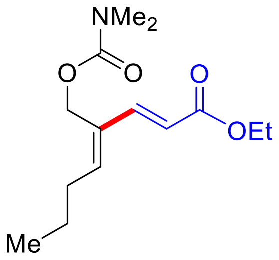


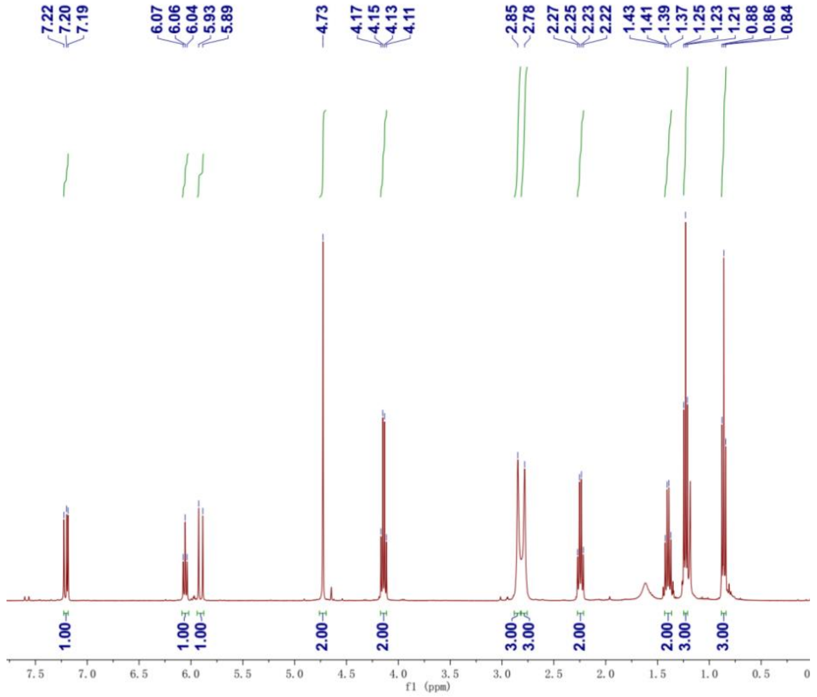


**Supplementary Figure 99.** $^{1}$H NMR spectrum **5ac** in $CDCl_3$

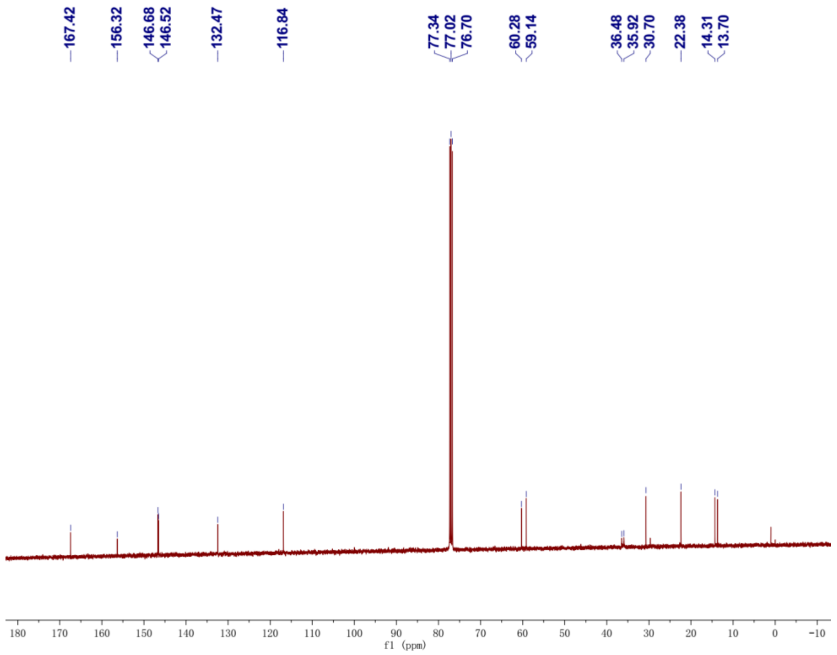


**Supplementary Figure 100.** $^{13}$C NMR spectrum for **5ac** in $CDCl_3$

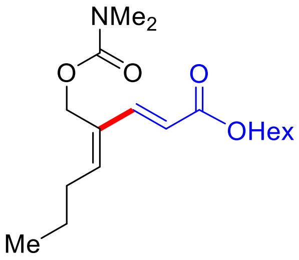


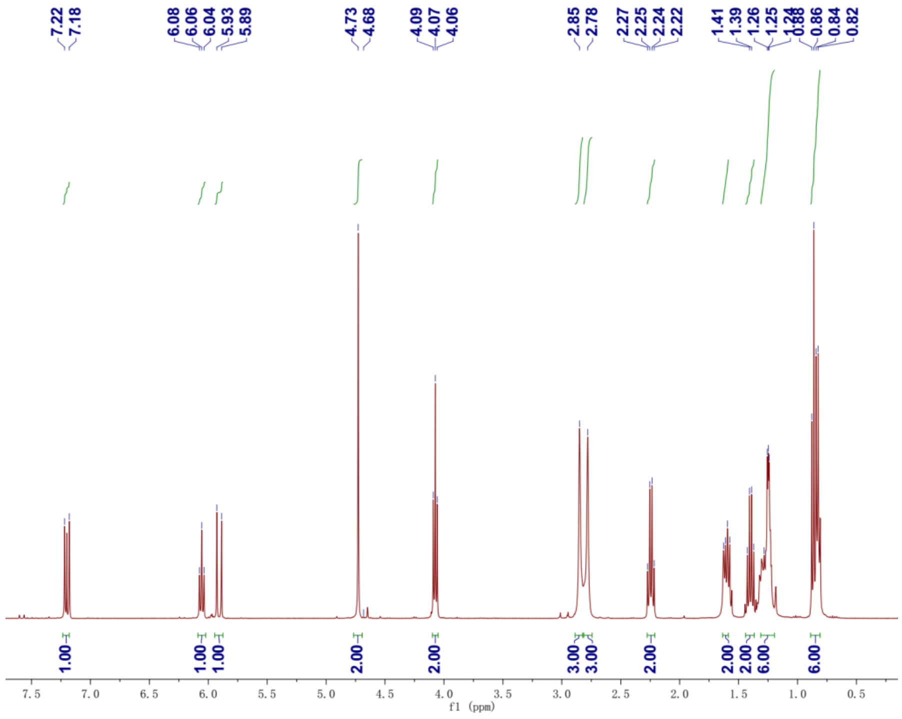

**Supplementary Figure 101.** $^{1}H$ NMR spectrum for **5ad** in $CDCl_3$

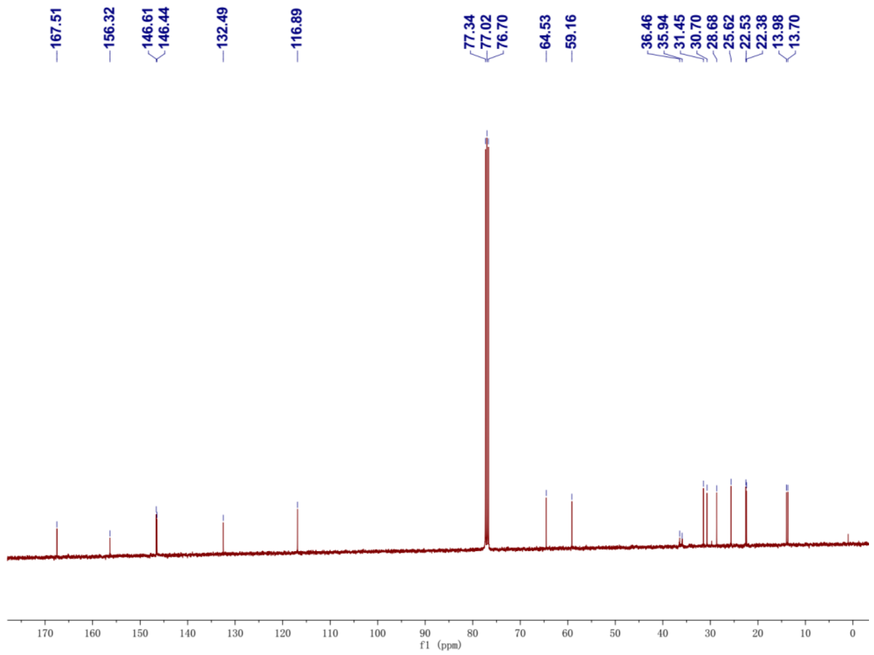

**Supplementary Figure 102.** $^{13}C$ NMR spectrum for **5ad** in $CDCl_3$

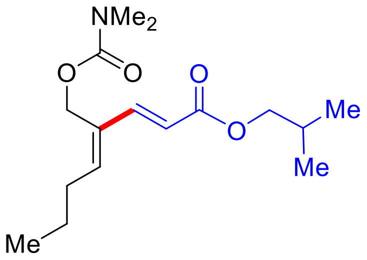


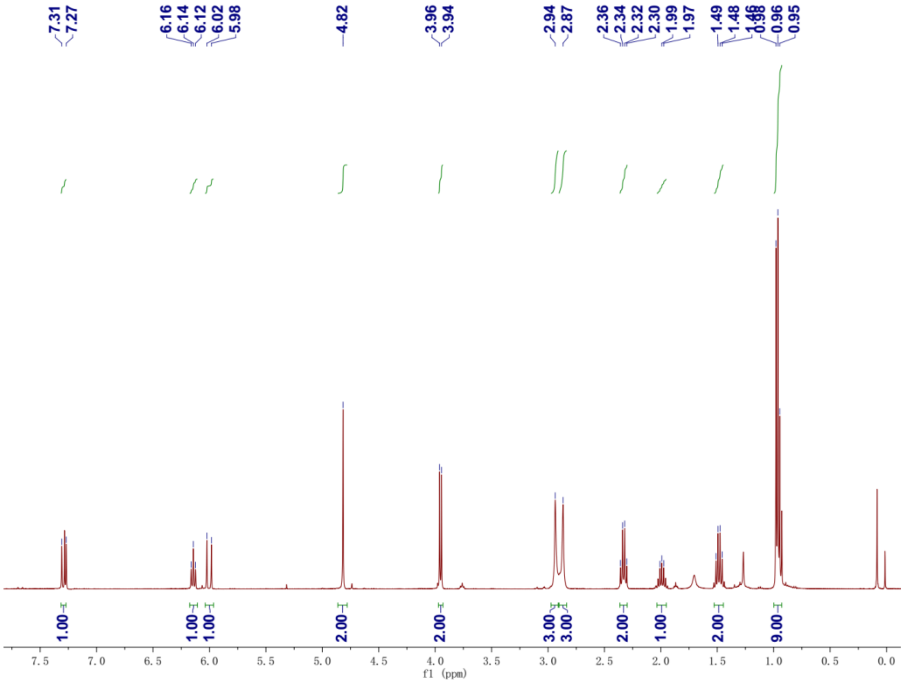


**Supplementary Figure 103.** $^{1}H$ NMR spectrum for **5ae** in $CDCl_3$

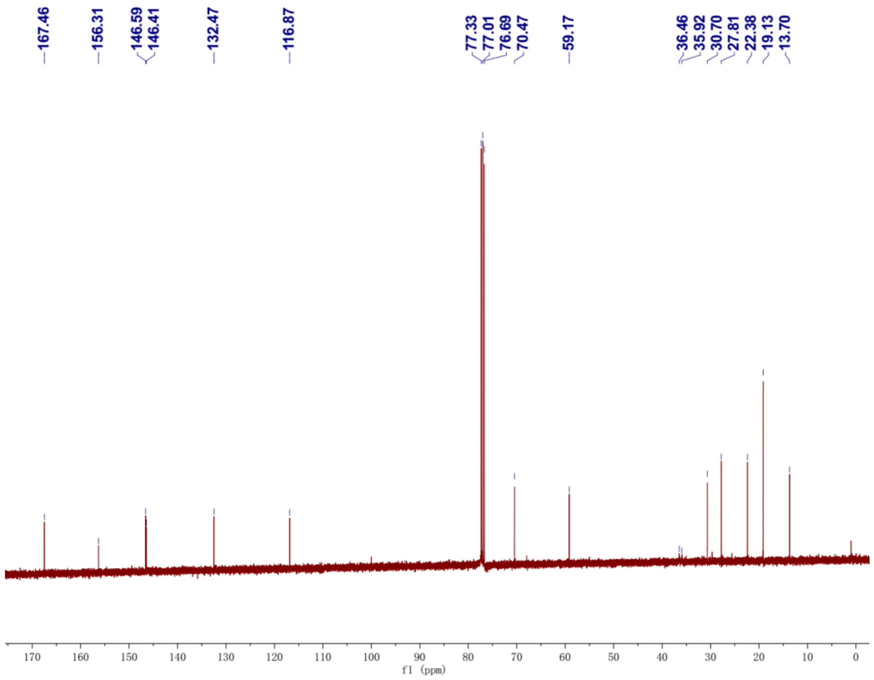


**Supplementary Figure 104.** $^{13}C$ NMR spectrum for **5ae** in $CDCl_3$

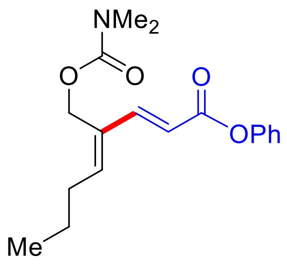


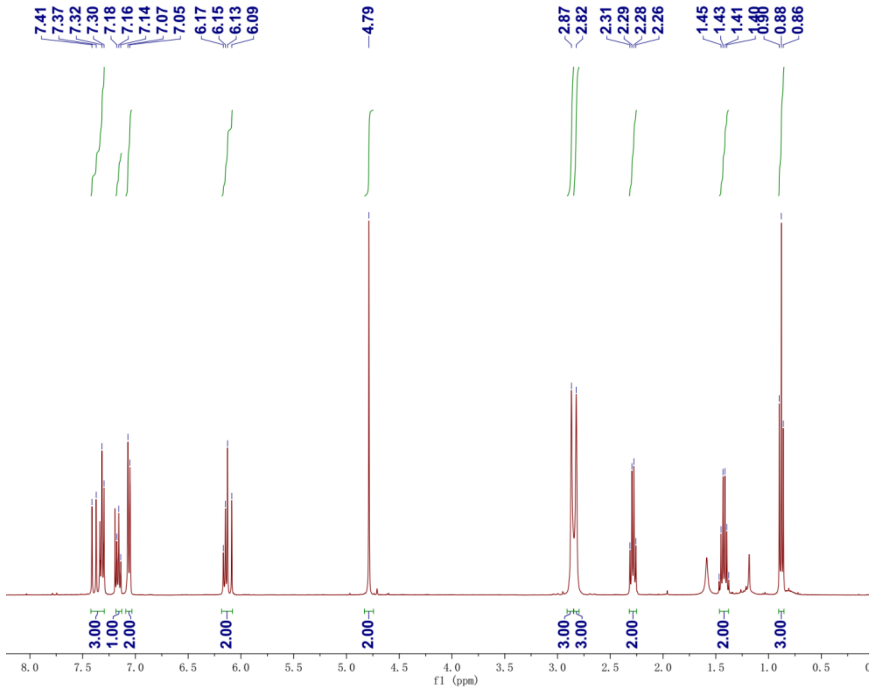


**Supplementary Figure 105.** $^{1}$H NMR spectrum for **5af** in $CDCl_3$

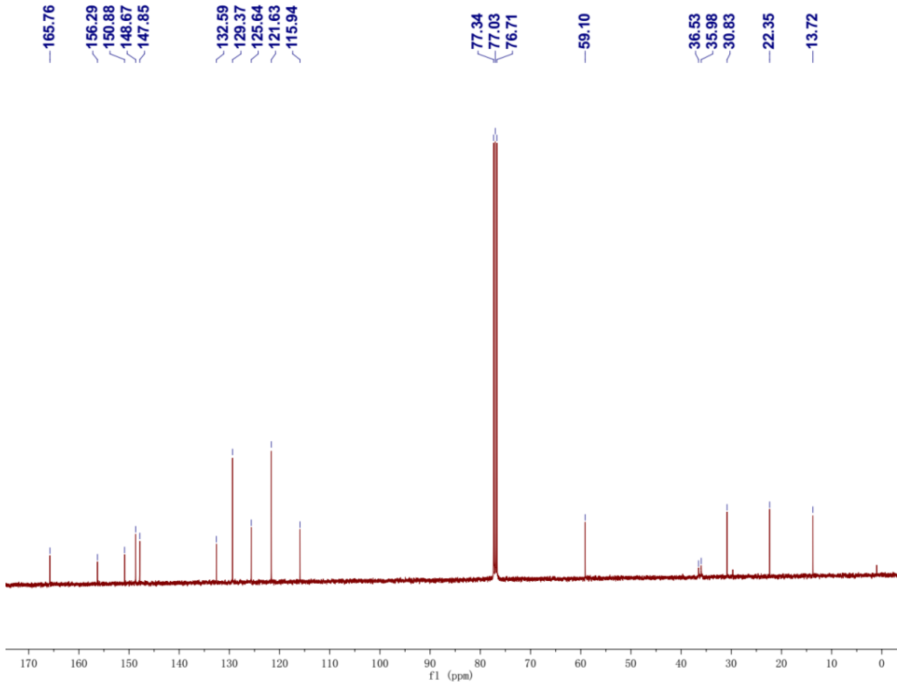


**Supplementary Figure 106.** $^{13}$C NMR spectrum for **5af** in $CDCl_3$

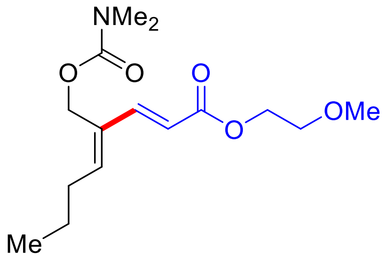


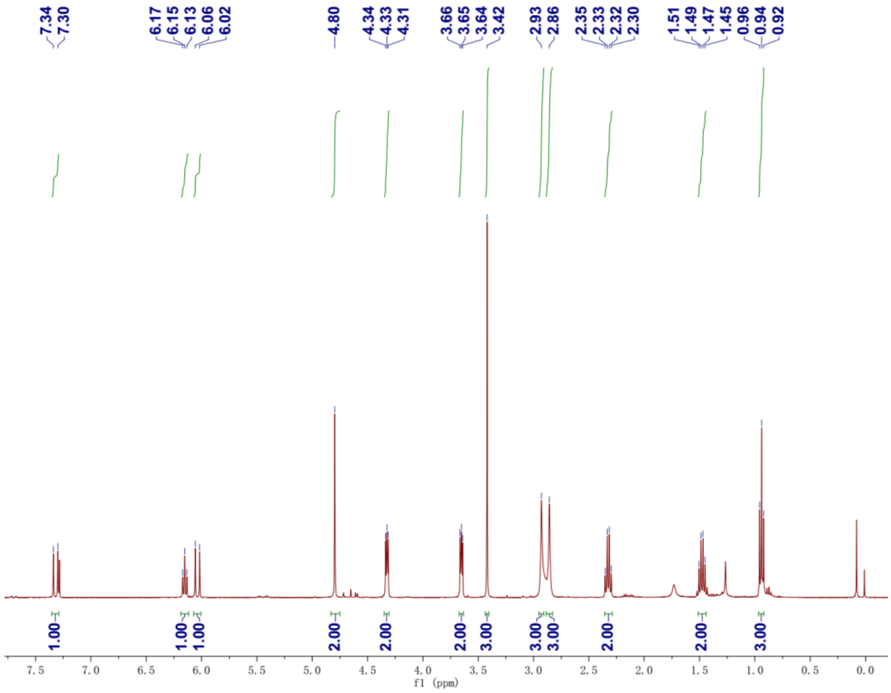


**Supplementary Figure 107.** $^{1}$H NMR spectrum for **5ag** in $CDCl_3$

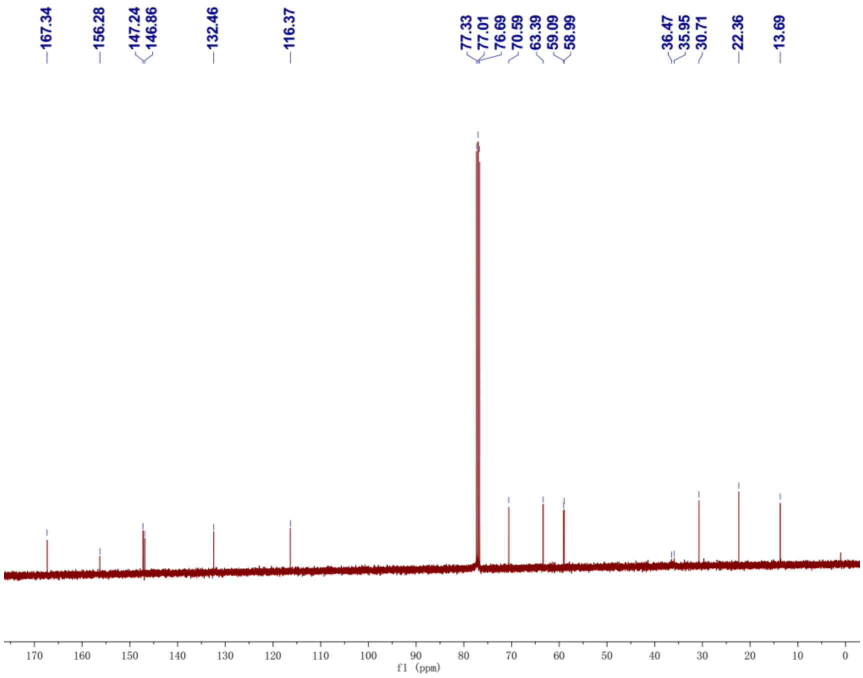


**Supplementary Figure 108.** $^{13}$C NMR spectrum for **5ag** in $CDCl_3$

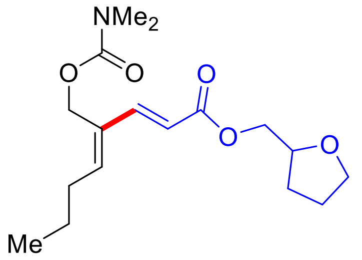


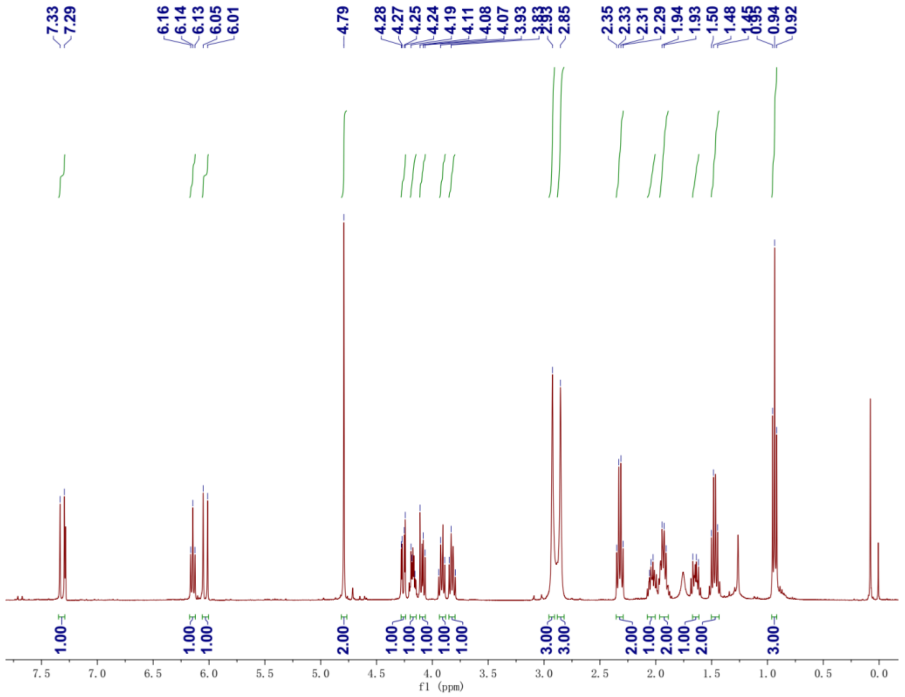


**Supplementary Figure 109.** $^{1}$H NMR spectrum for **5ah** in $CDCl_3$

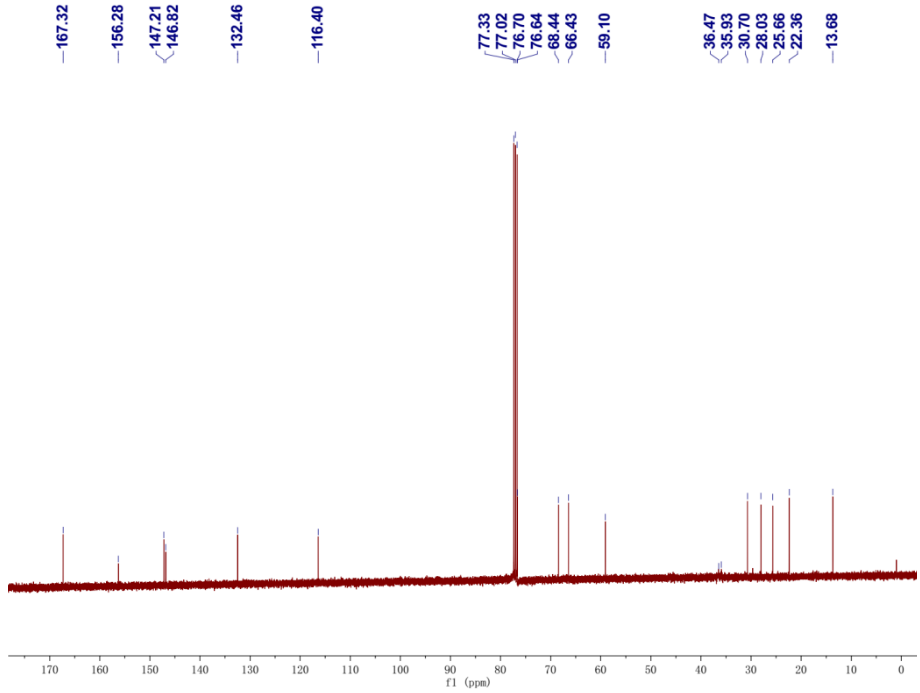


**Supplementary Figure 110.** $^{13}$C NMR spectrum for **5ah** in $CDCl_3$

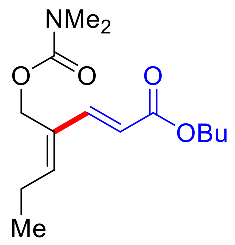


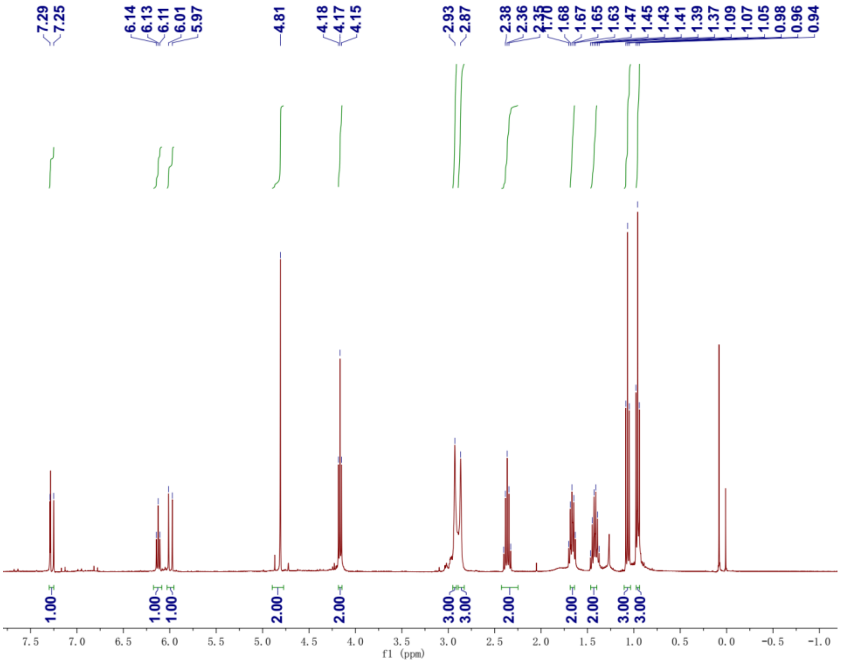


**Supplementary Figure 111.** $^{1}H$ NMR spectrum for **5ba** in $CDCl_3$

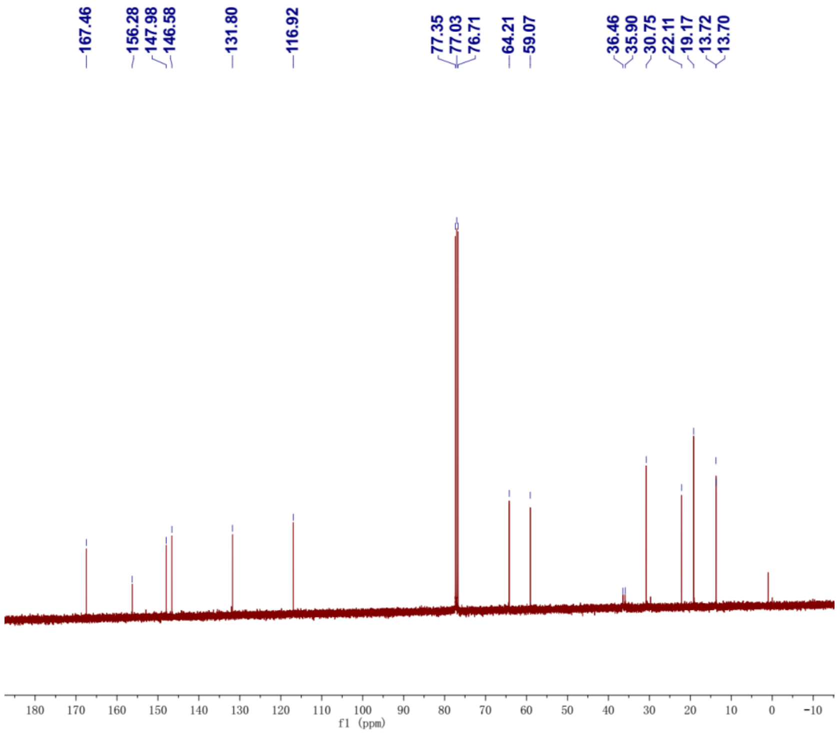


**Supplementary Figure 112.** $^{13}C$ NMR spectrum for **5ba** in $CDCl_3$

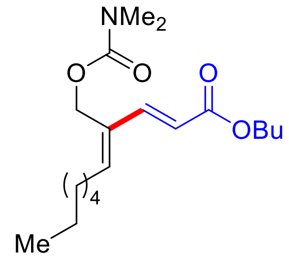


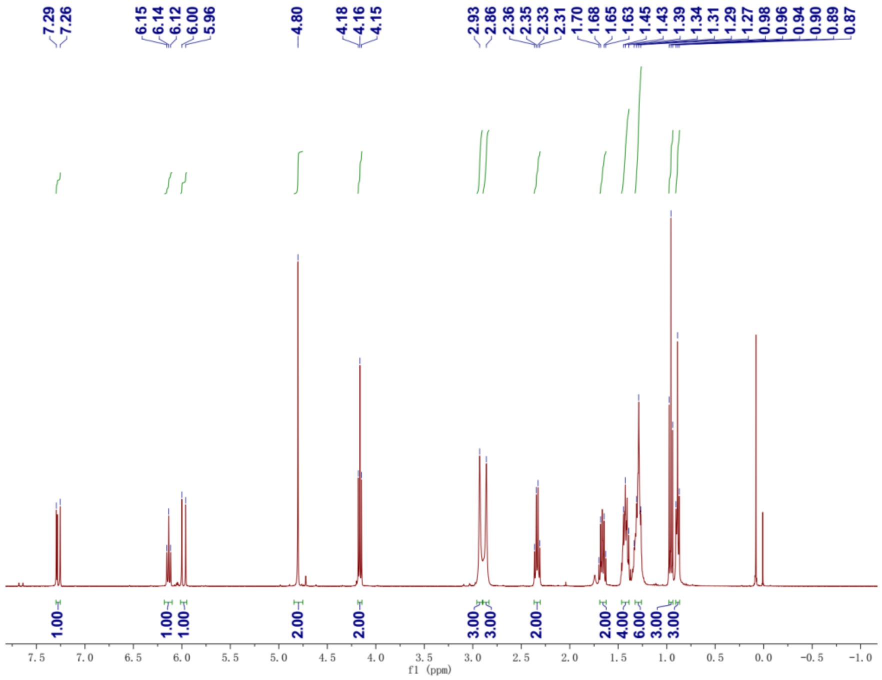


**Supplementary Figure 113.** $^{1}$H NMR spectrum for **5ca** in $CDCl_3$

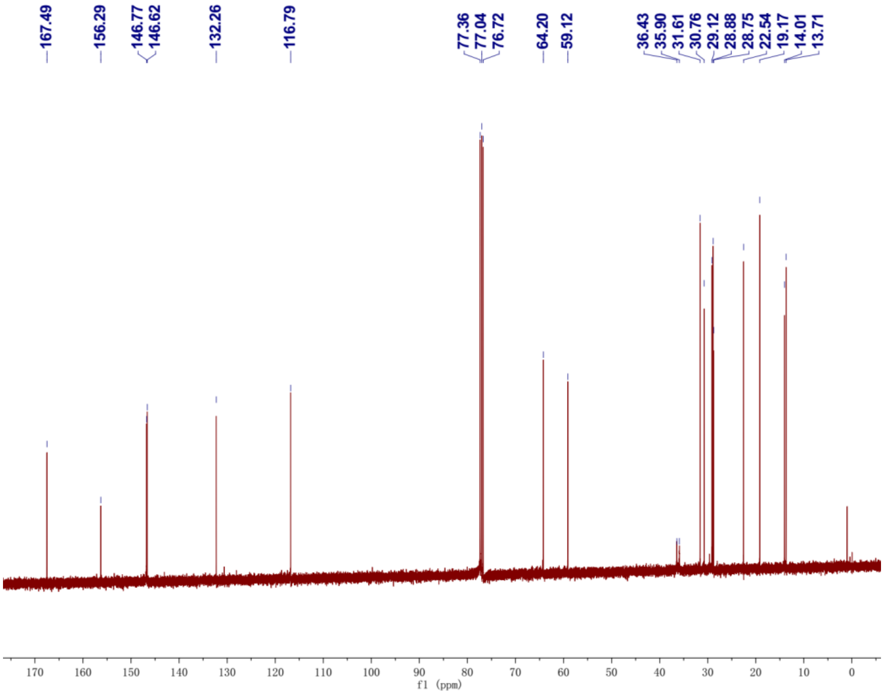


**Supplementary Figure 114.** $^{13}$C NMR spectrum for **5ca** in $CDCl_3$

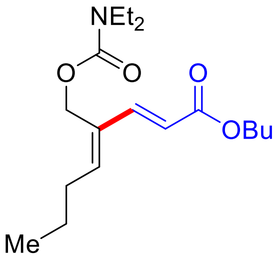


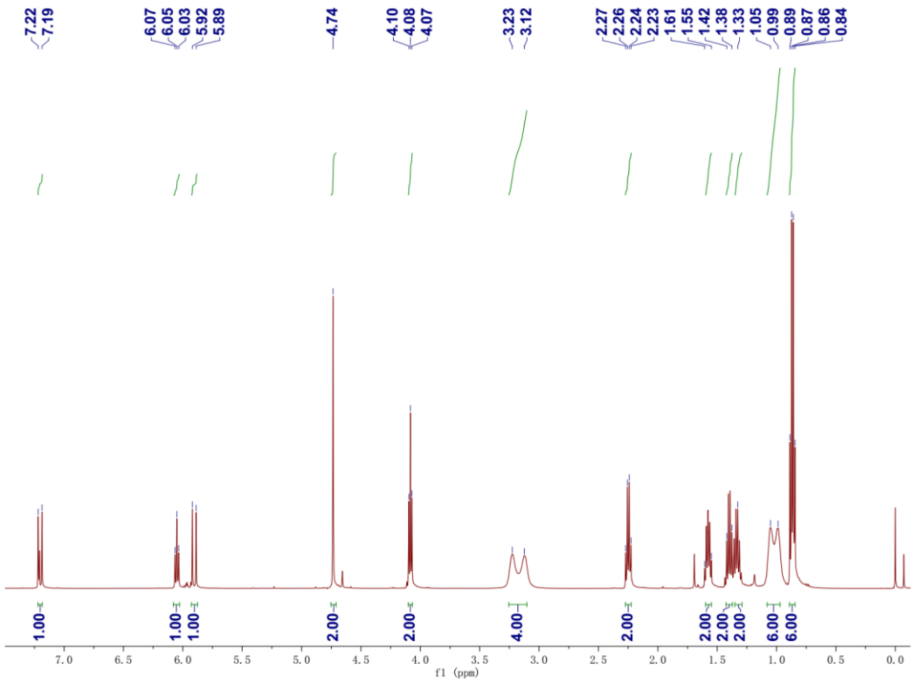

**Supplementary Figure 115.** $^{1}$H NMR spectrum for **5da** in $CDCl_3$

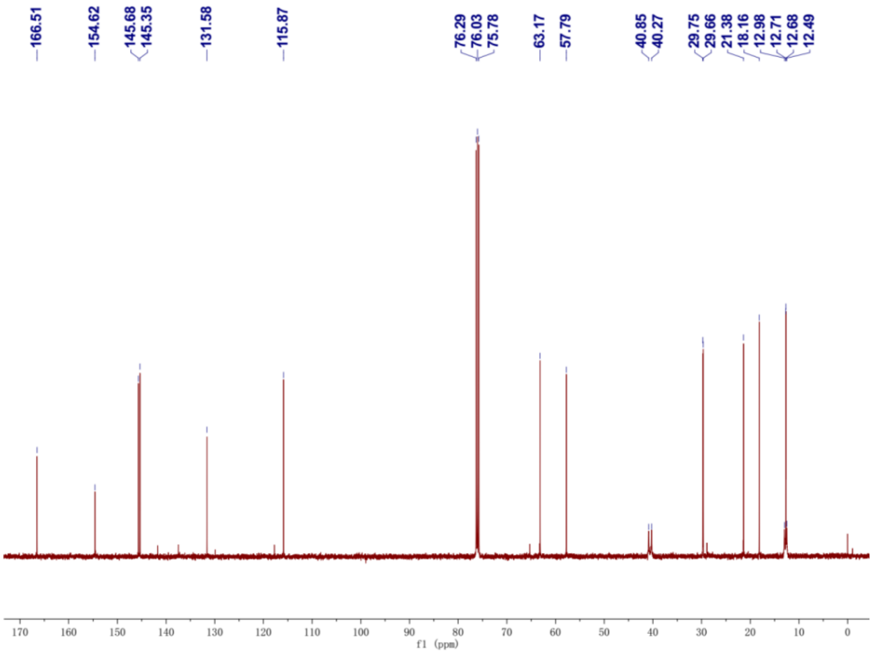

**Supplementary Figure 116.** $^{13}$C NMR spectrum for **5da** in $CDCl_3$

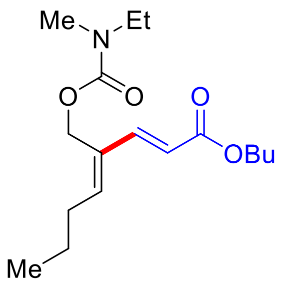


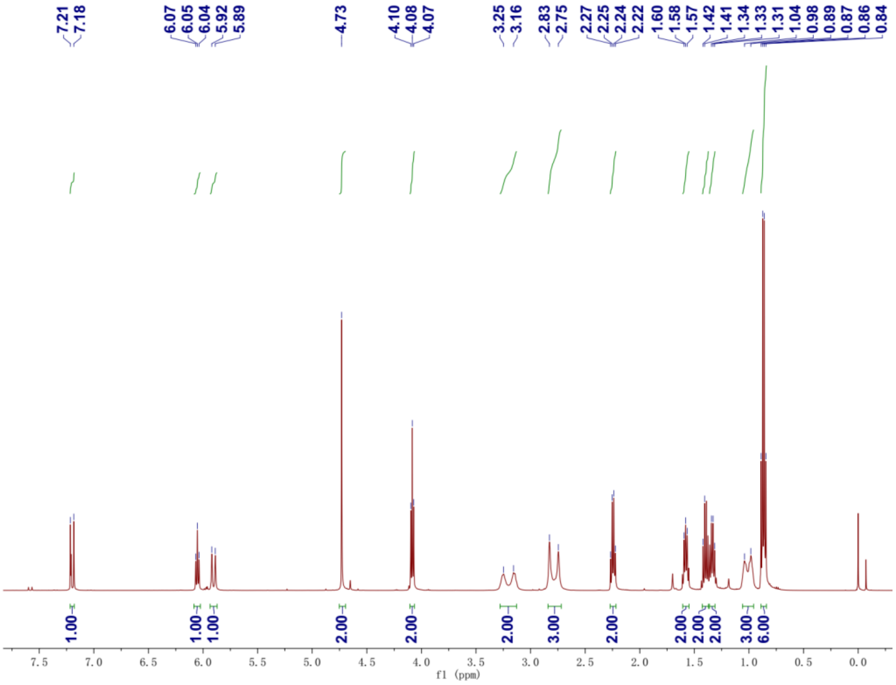


**Supplementary Figure 117.** $^{1}$H NMR spectrum for **5ea** in $CDCl_3$

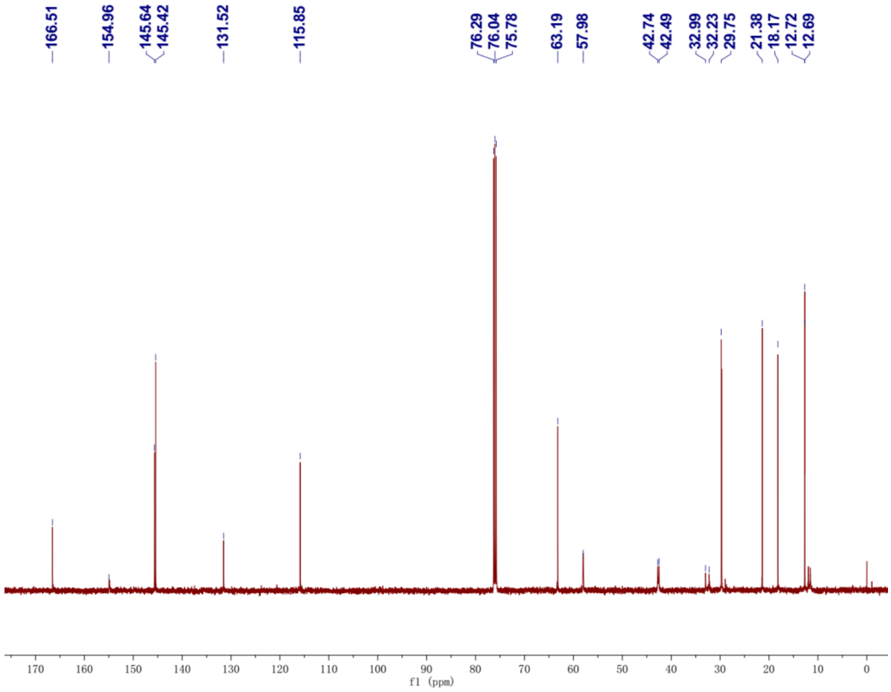


**Supplementary Figure 118.** $^{13}$C NMR spectrum for **5ea** in $CDCl_3$

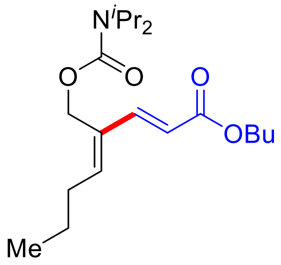


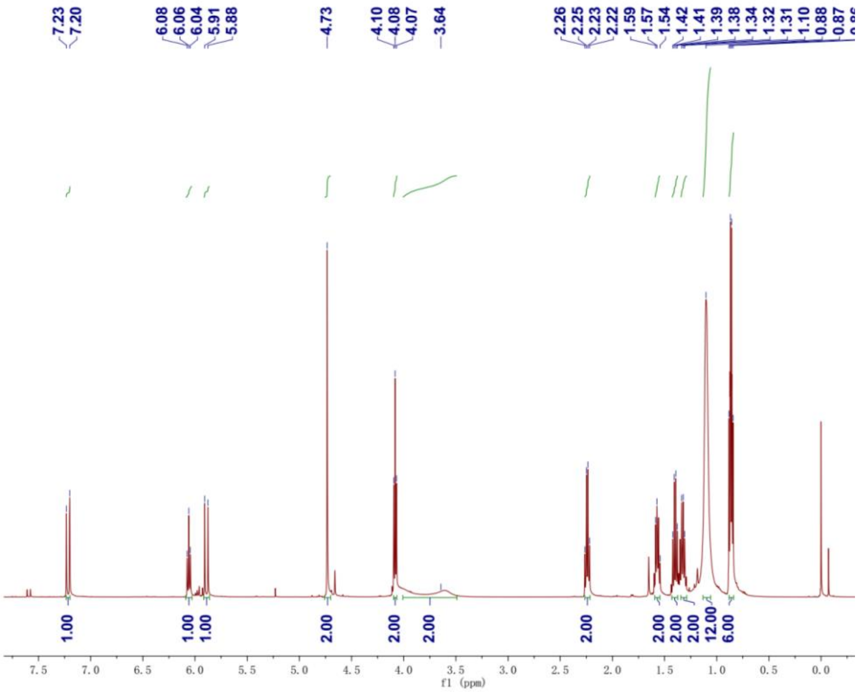


**Supplementary Figure 119.** $^{1}$H NMR spectrum for **5fa** in $CDCl_3$

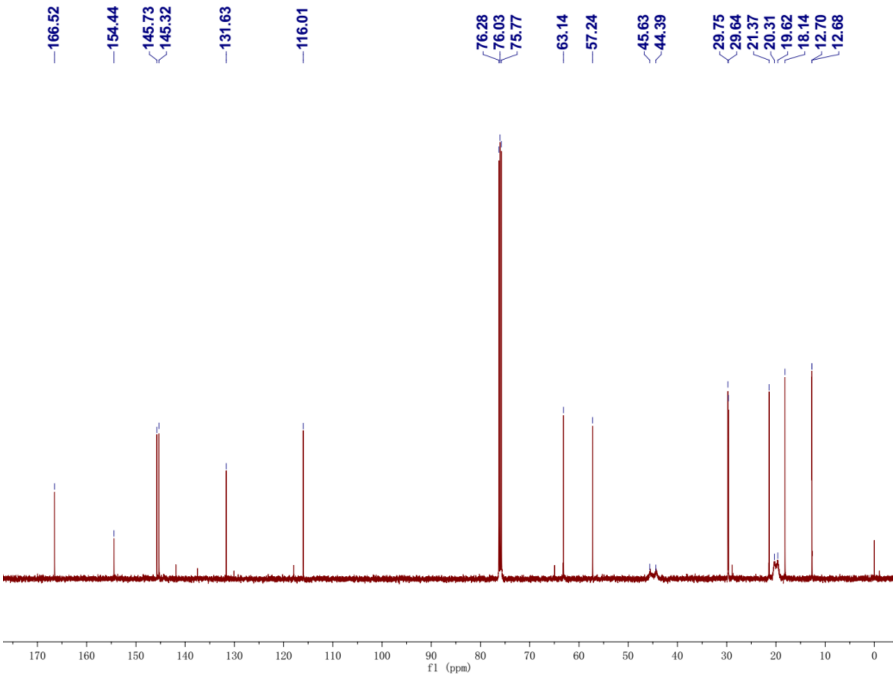


**Supplementary Figure 120.** $^{13}$C NMR spectrum for **5fa** in $CDCl_3$

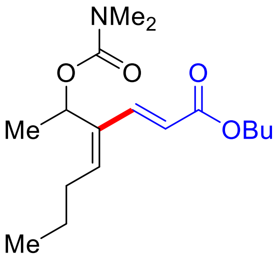


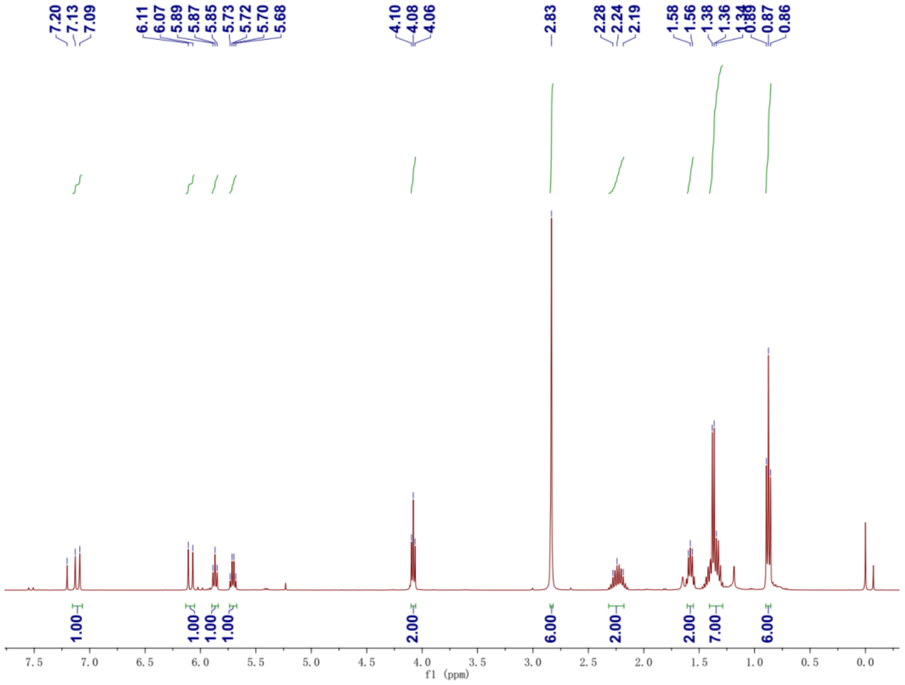


**Supplementary Figure 121.** $^{1}H$ NMR spectrum for **5ga** in $CDCl_3$

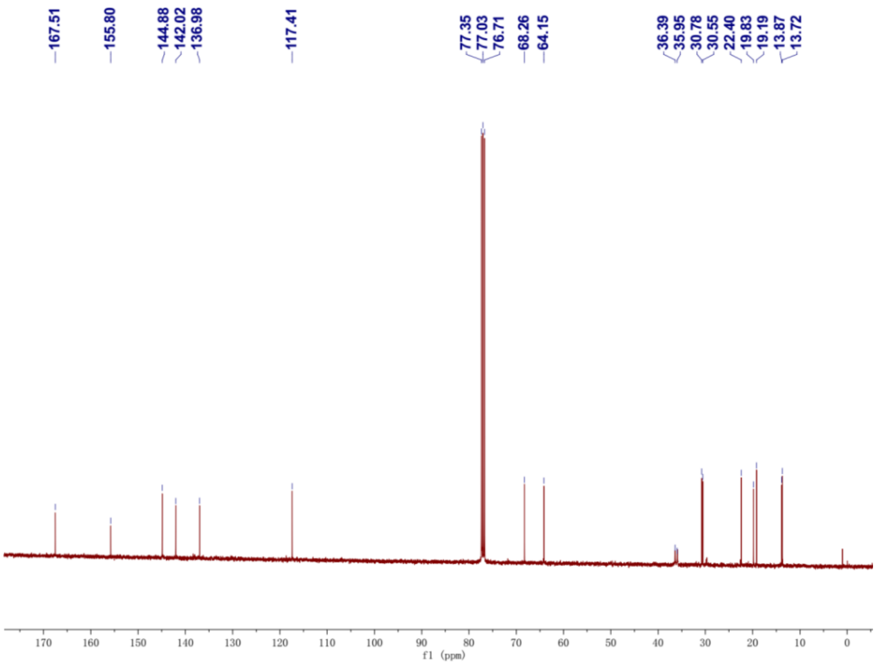


**Supplementary Figure 122.** $^{13}C$ NMR spectrum for **5ga** in $CDCl_3$

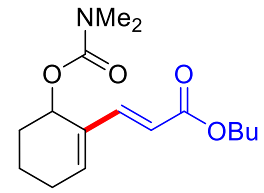


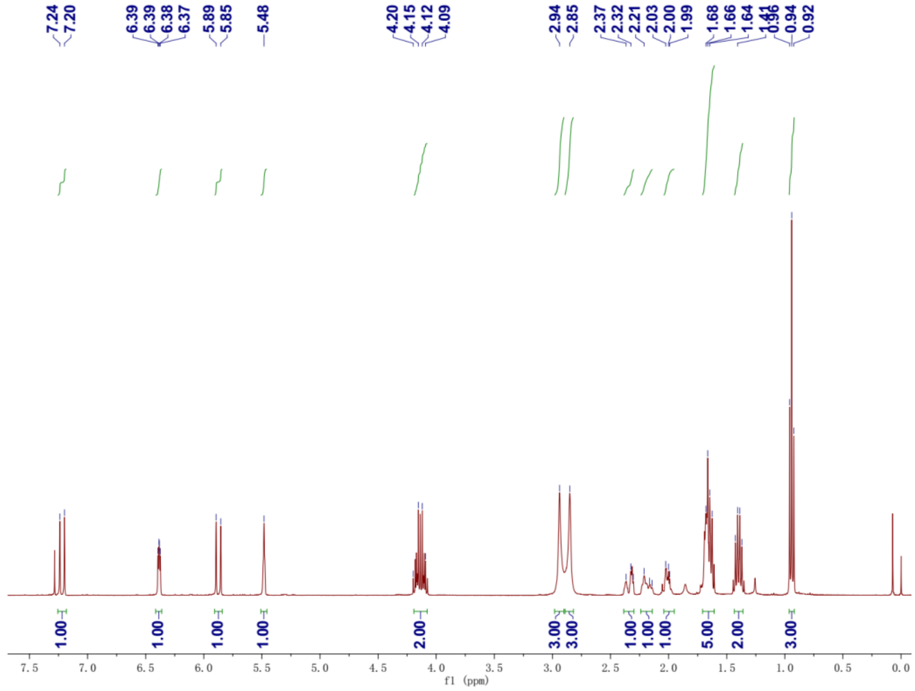

**Supplementary Figure 123.** $^{1}$H NMR spectrum for **5ha** in $CDCl_3$

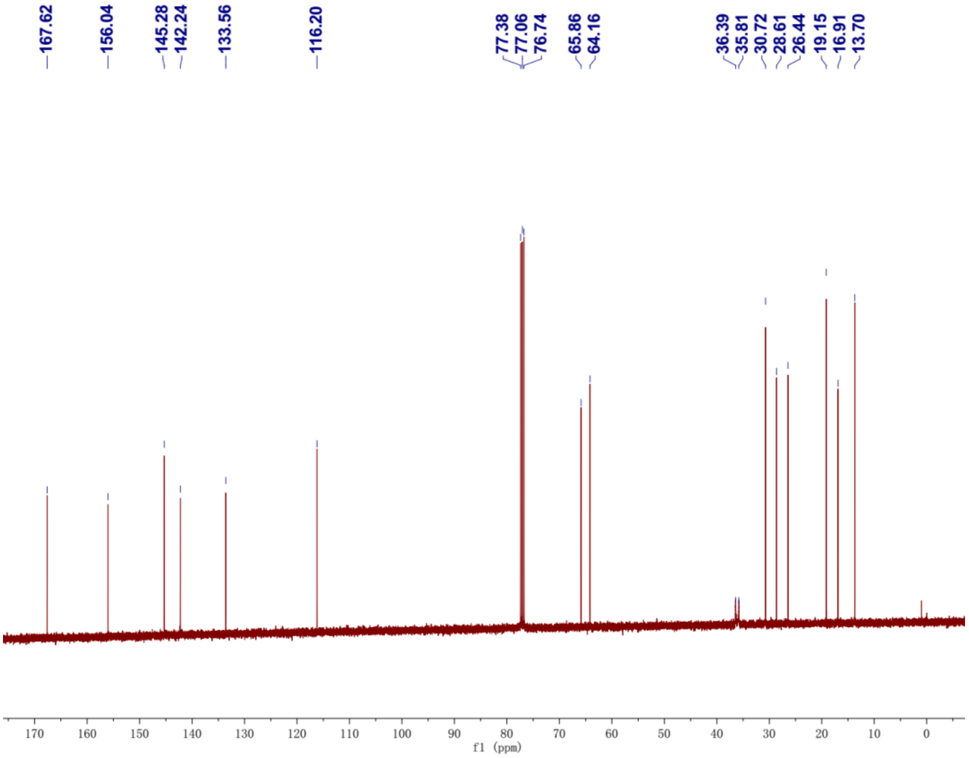

**Supplementary Figure 124.** $^{13}$C NMR spectrum for **5ha** in $CDCl_3$

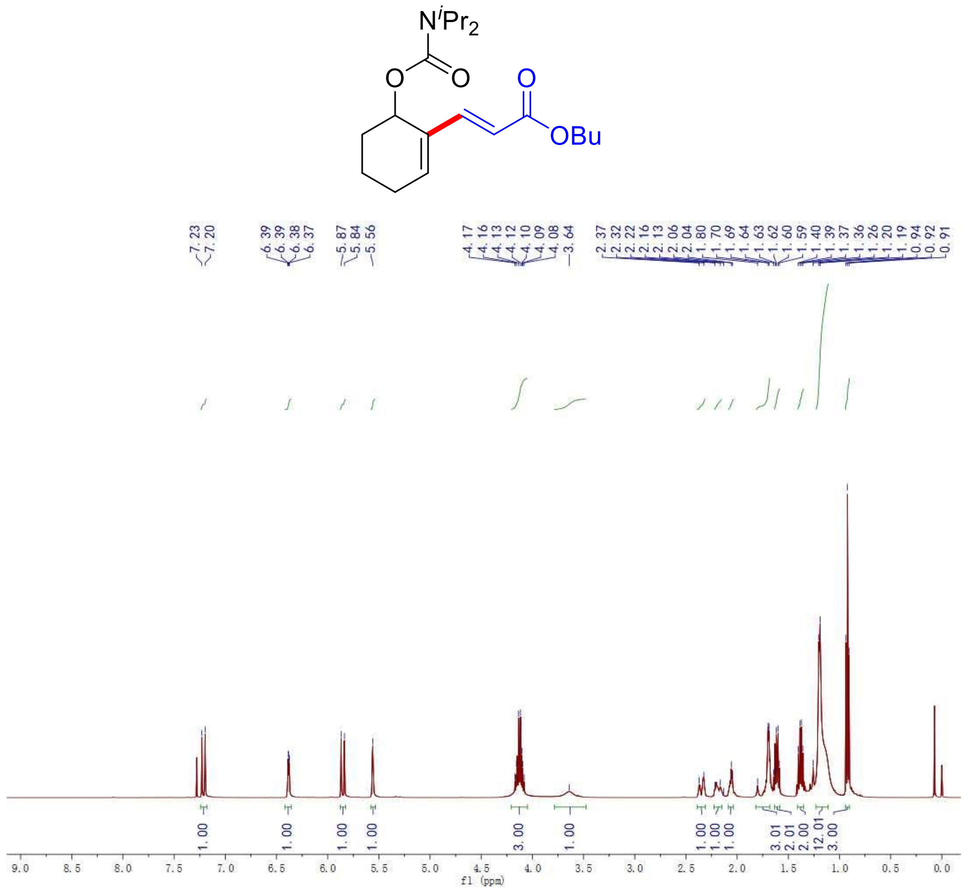


**Supplementary Figure 125.** $^1$H NMR spectrum for **5ia** in $CDCl_3$

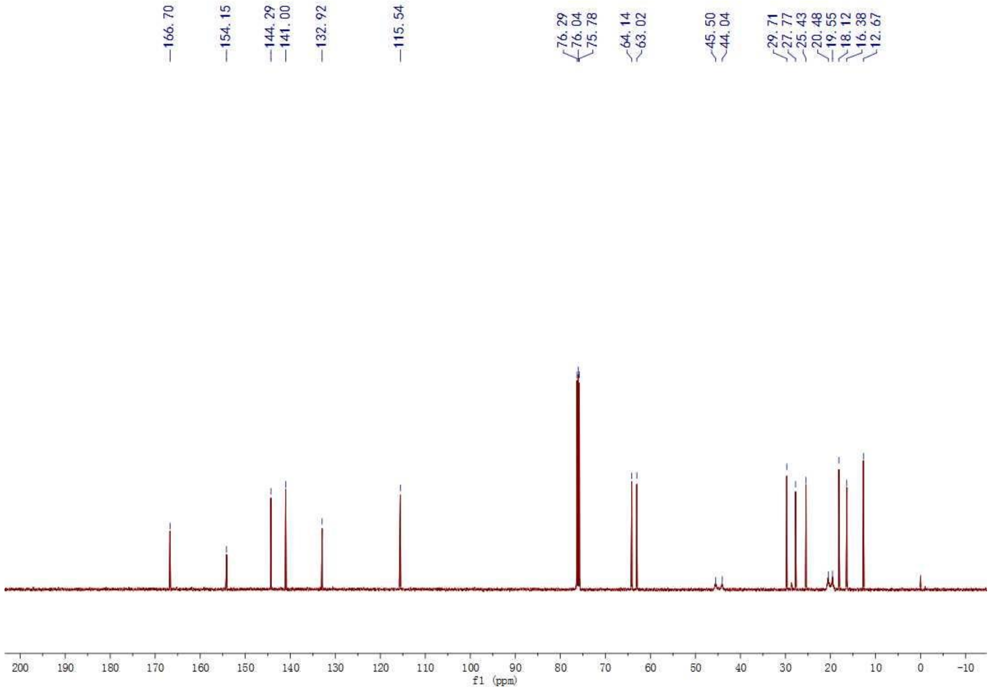


**Supplementary Figure 126.** $^{13}$C NMR spectrum for **5ia** in $CDCl_3$

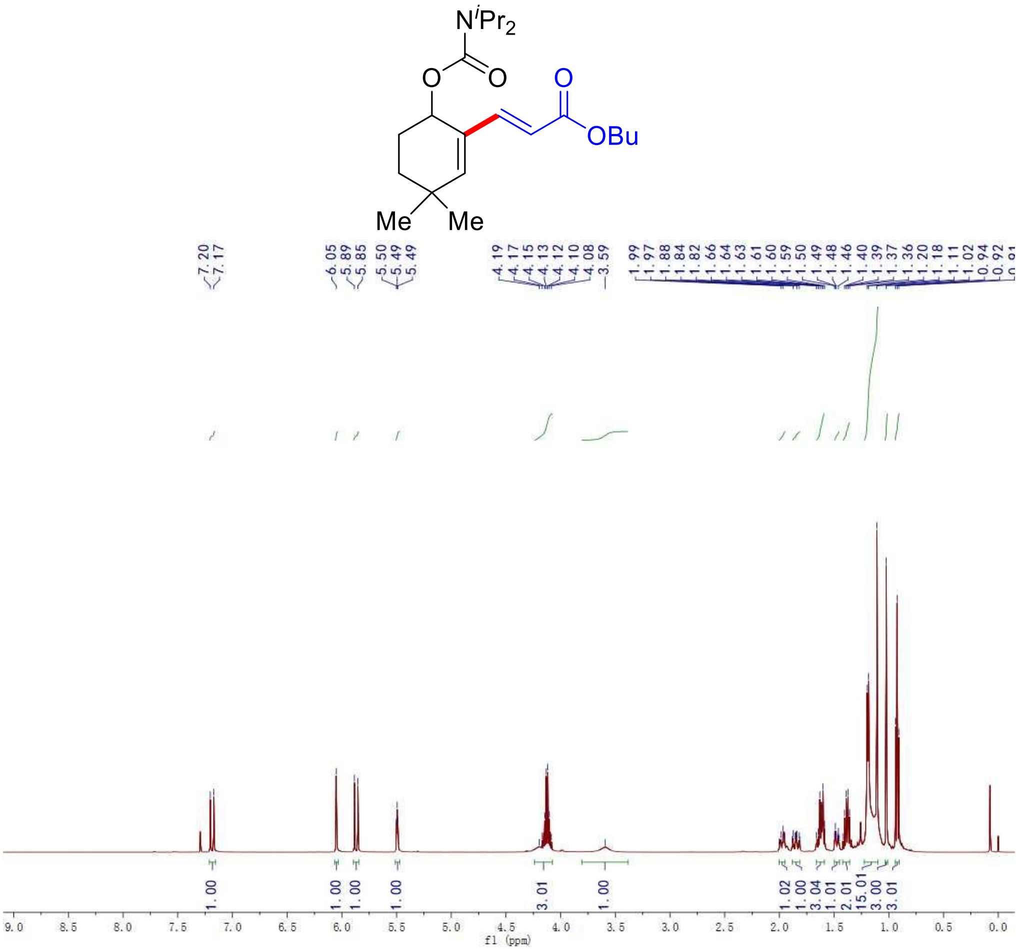


**Supplementary Figure 127.** $^{1}$H NMR spectrum for **5ja** in $CDCl_3$

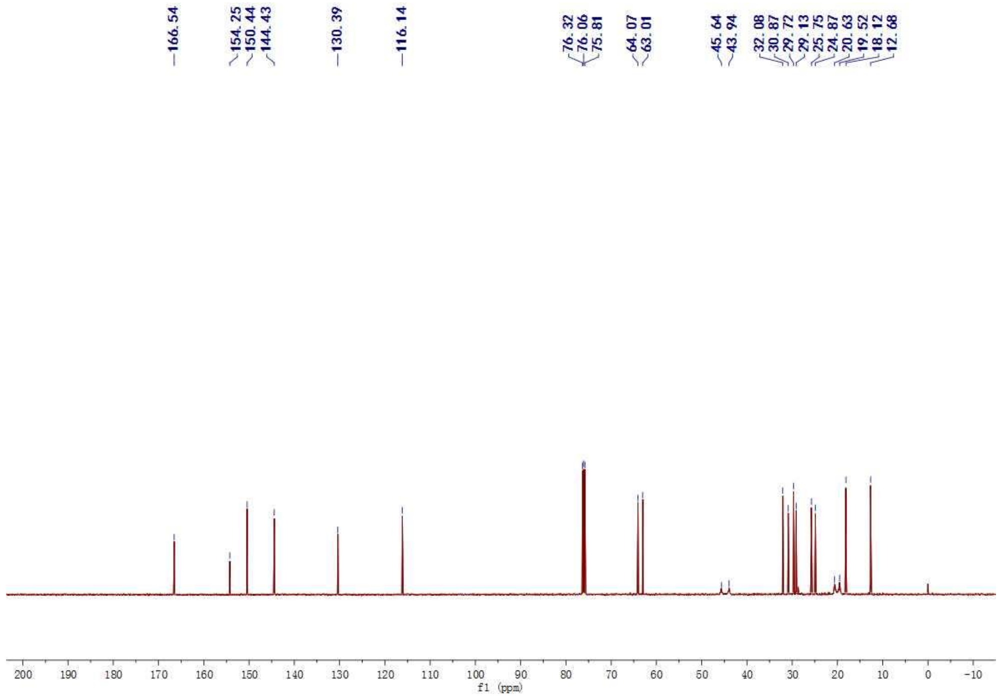


**Supplementary Figure 128.** $^{13}$C NMR spectrum for **5ja** in $CDCl_3$

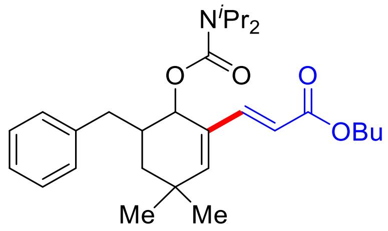


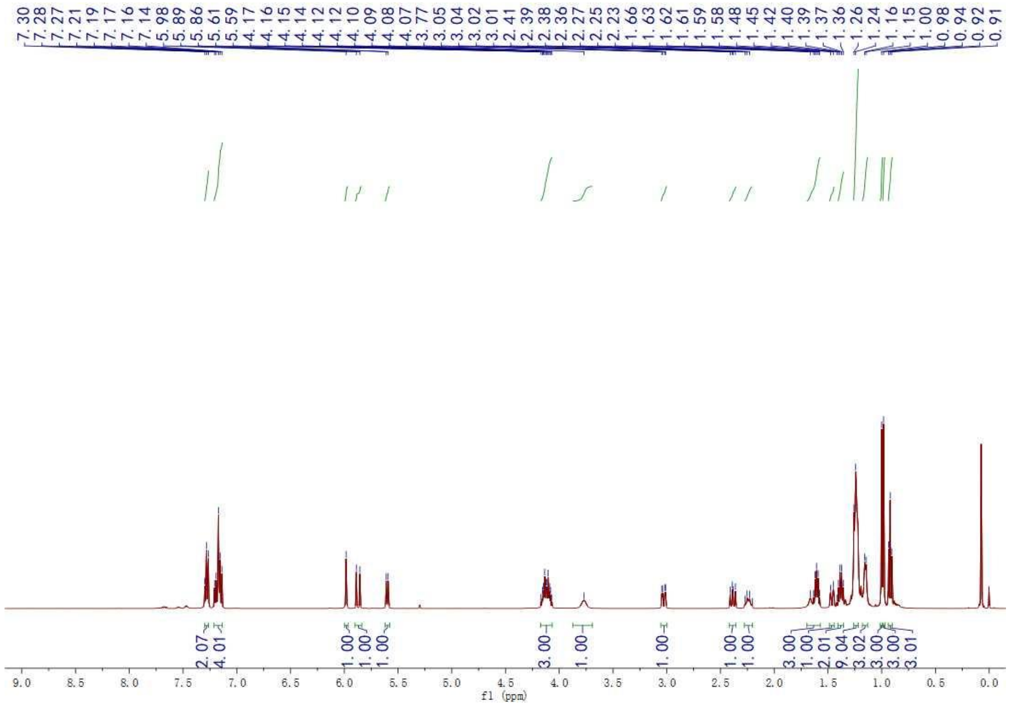


**Supplementary Figure 129.** $^{1}$H NMR spectrum for **5ka** in $CDCl_3$

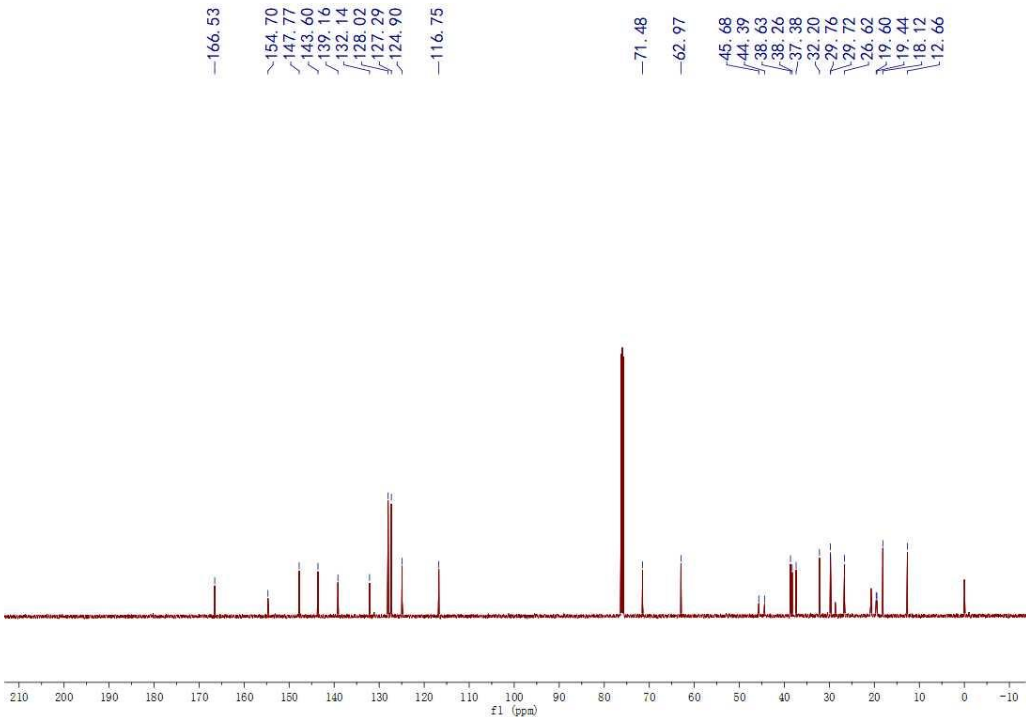


**Supplementary Figure 130.** $^{13}$C NMR spectrum for **5ka** in $CDCl_3$

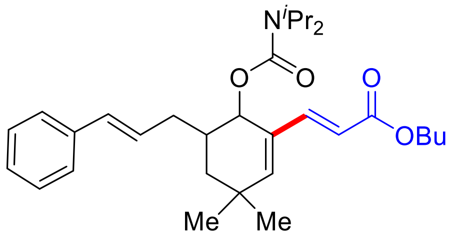

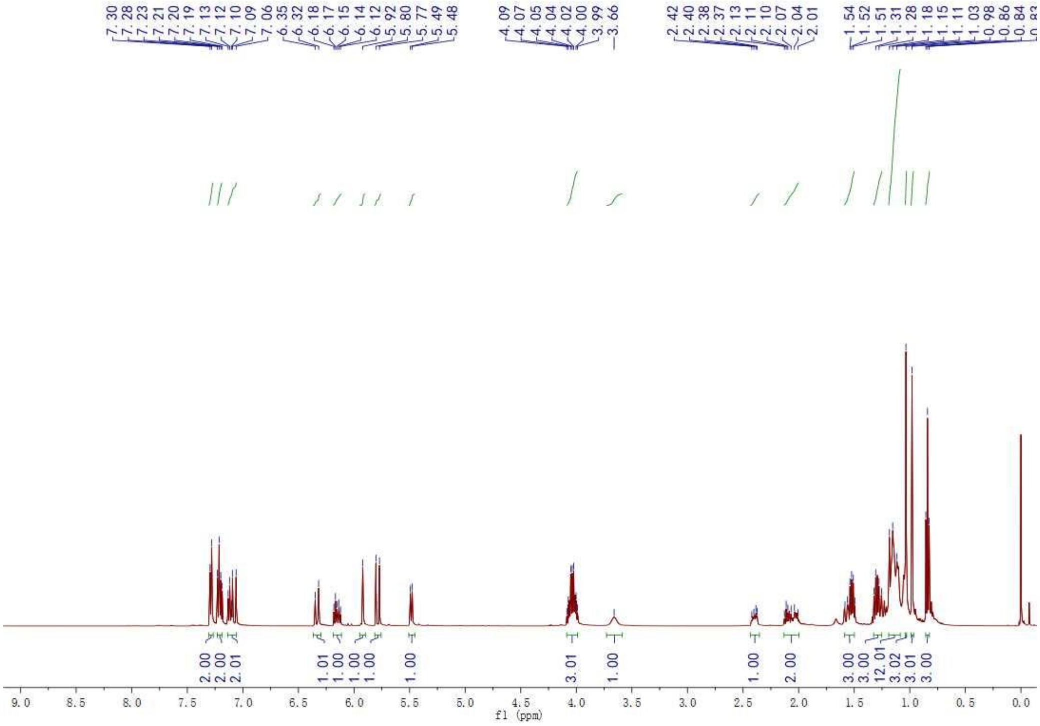

**Supplementary Figure 131.** $^{1}H$ NMR spectrum for **5la** in $CDCl_3$

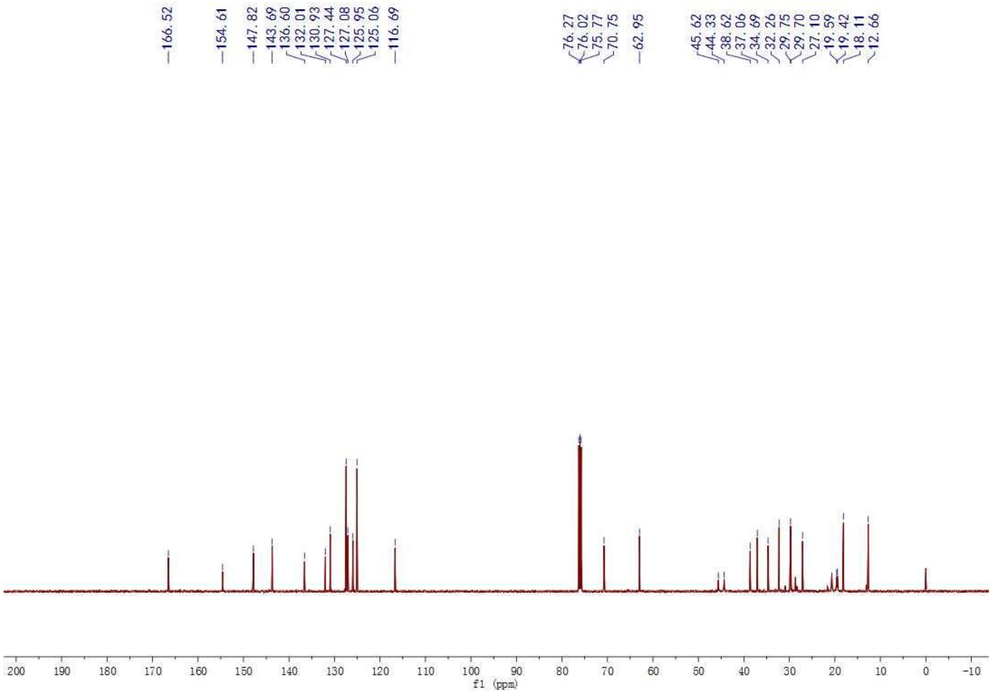

**Supplementary Figure 132.** $^{13}C$ NMR spectrum for **5la** in $CDCl_3$

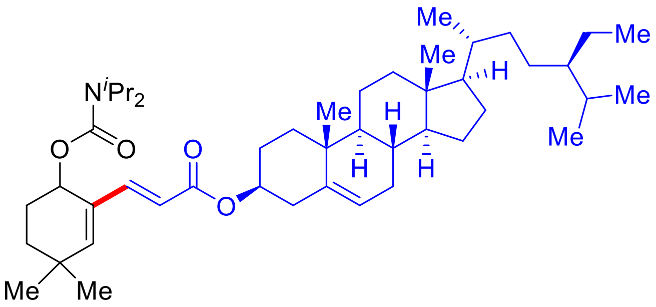


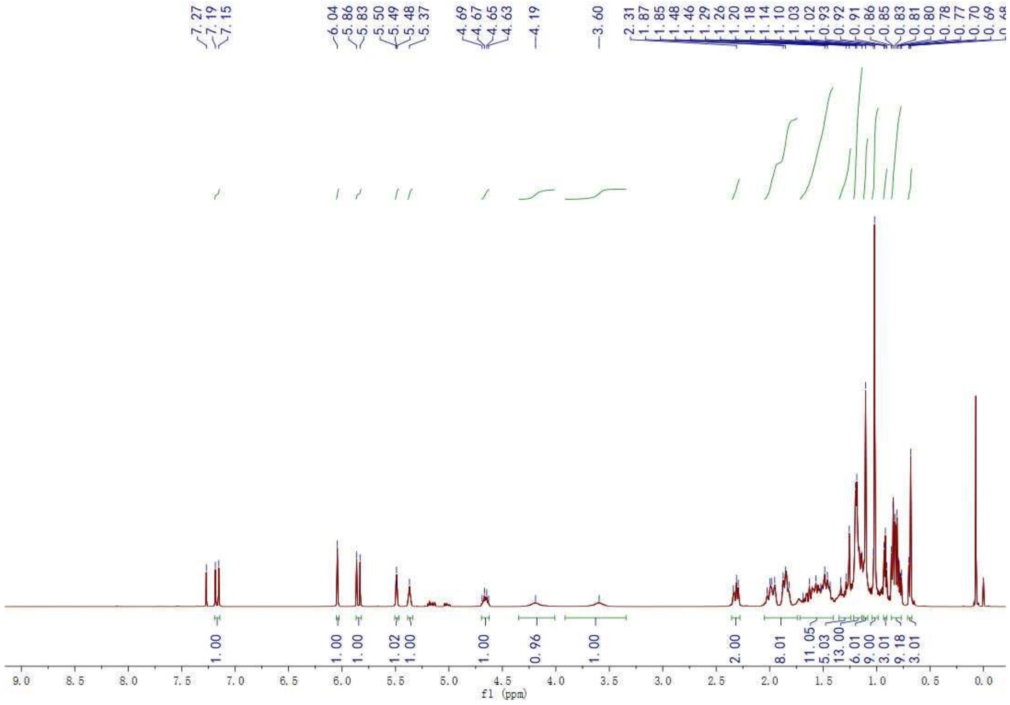

**Supplementary Figure 133.** $^{1}$H NMR spectrum for **5jp** in $CDCl_3$

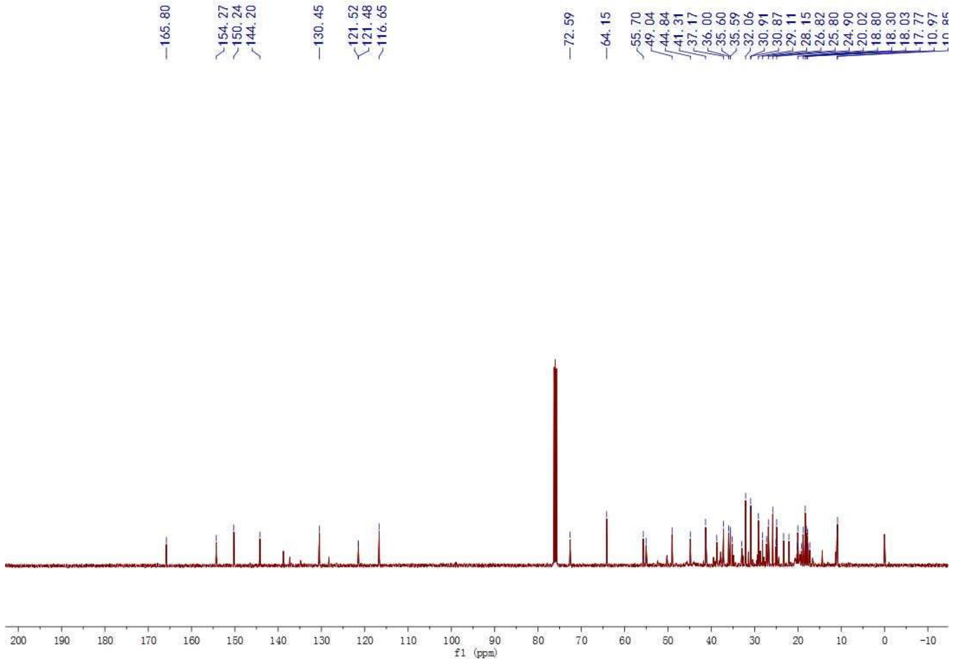

**Supplementary Figure 134.** $^{13}$C NMR spectrum for **5jp** in $CDCl_3$

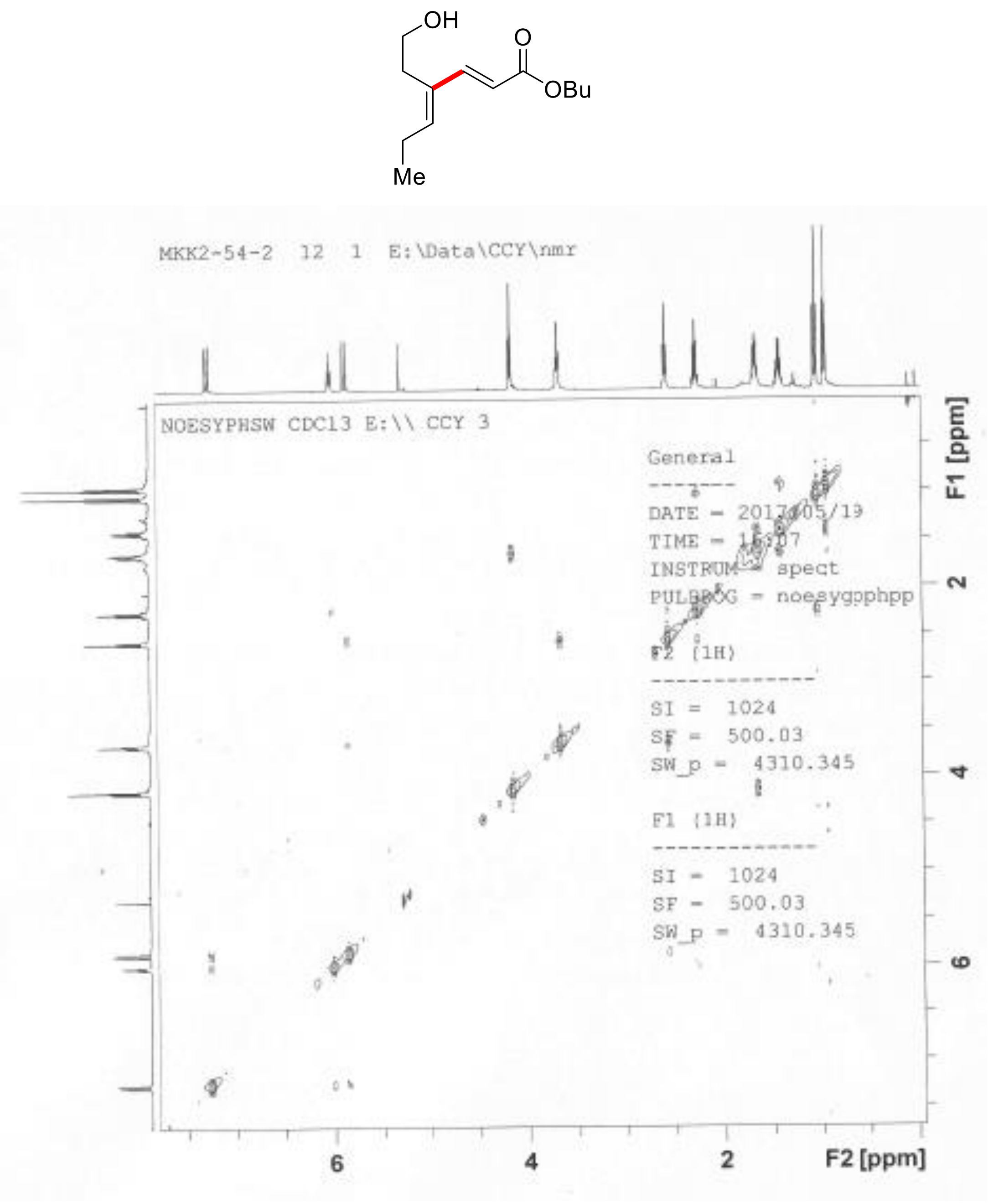


**Supplementary Figure 135.** NOE spectrum for **3aa** in $CDCl_3$

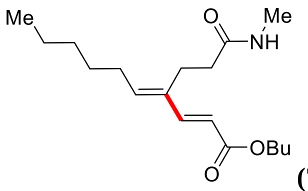


**(7aa)**

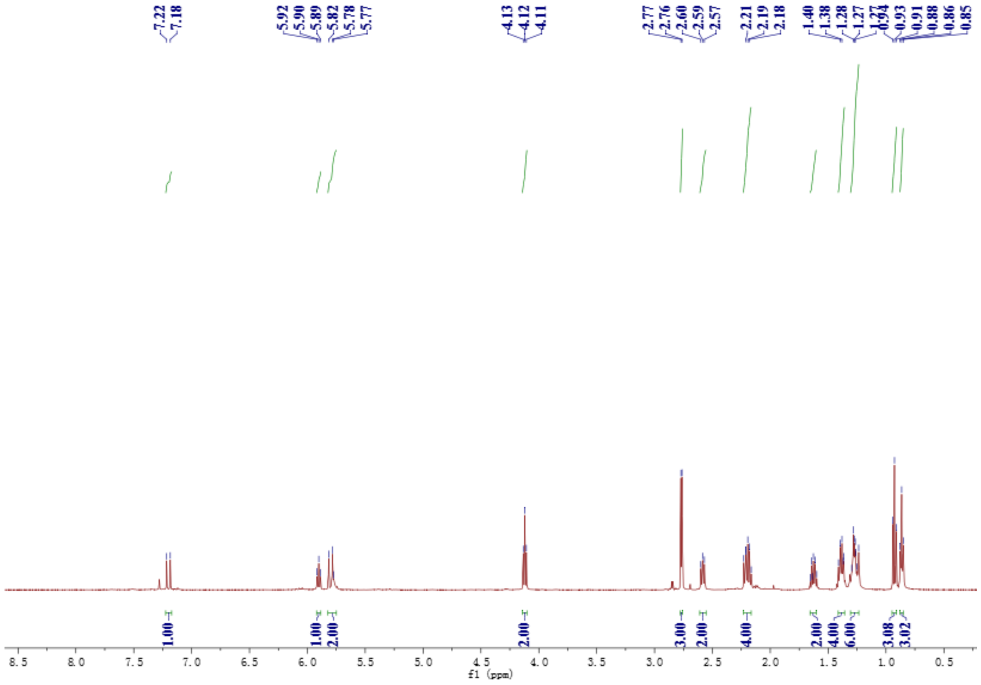

**Supplementary Figure 136.** $^1$H NMR spectrum for **7aa** in $CDCl_3$

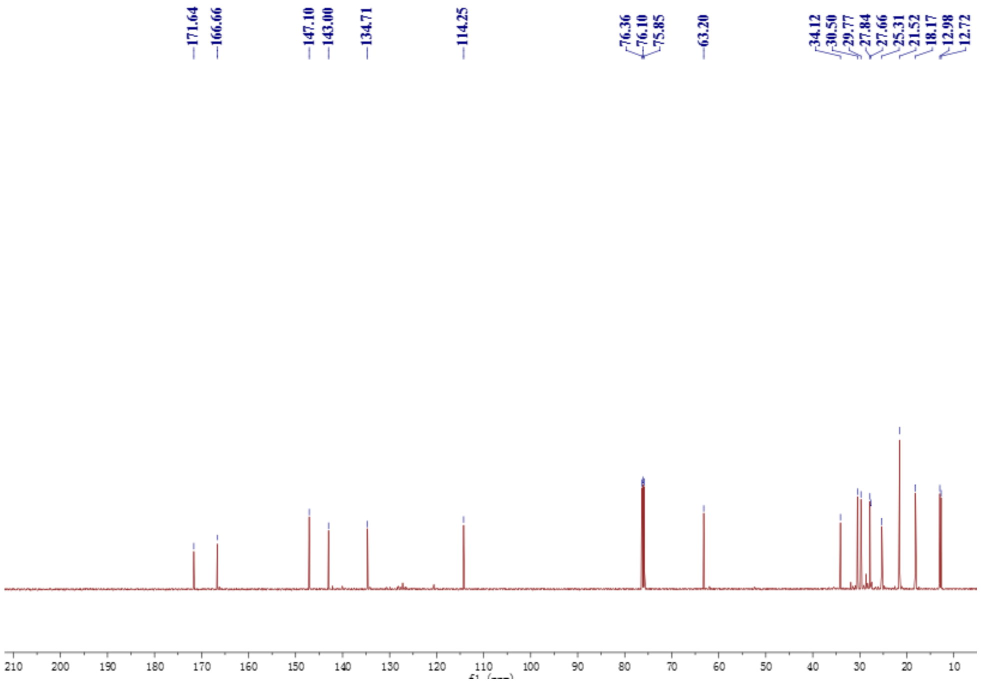

**Supplementary Figure 137.** $^{13}$C NMR spectrum for **7aa** in $CDCl_3$

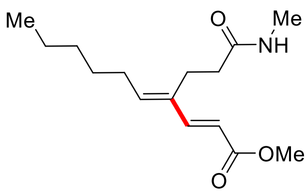


(7ab)

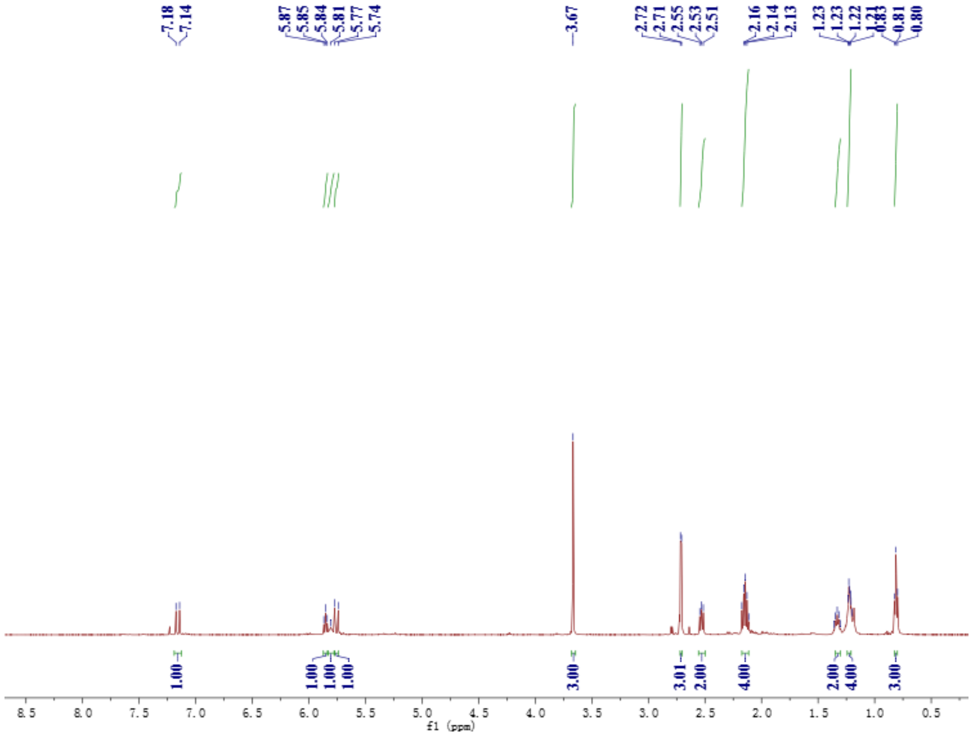

Supplementary Figure 138. $^{1}H$ NMR spectrum for **7ab** in $CDCl_3$

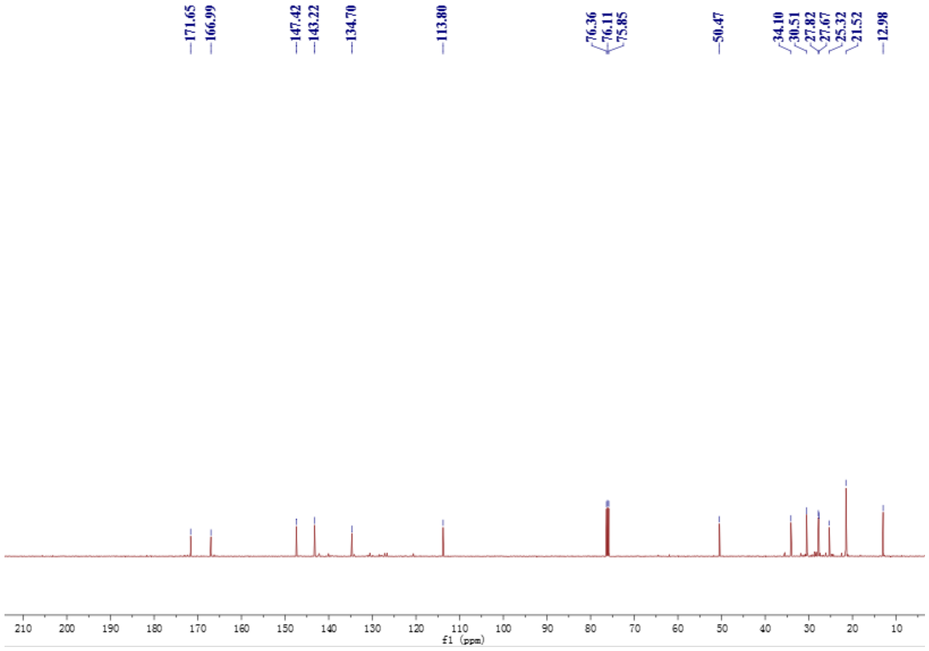

**Supplementary Figure 139.** $^{13}C$ NMR spectrum for **7ab** in $CDCl_3$

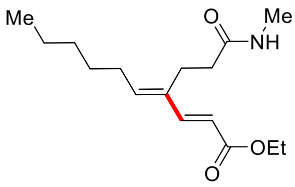


**(7ac)**

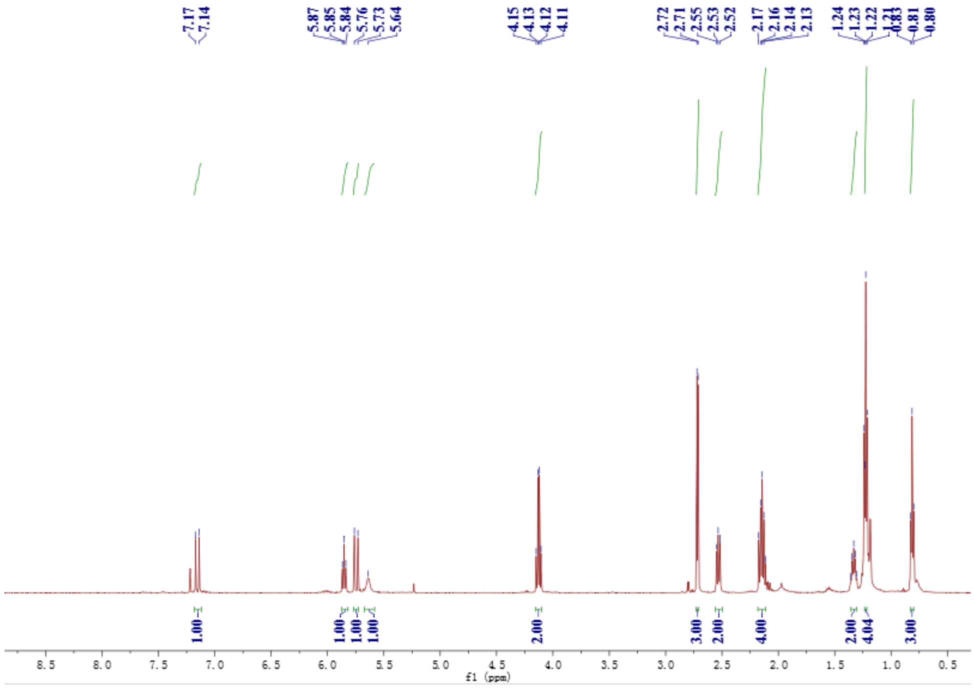


**Supplementary Figure 140.** $^{1}$H NMR spectrum for **7ac** in $CDCl_3$

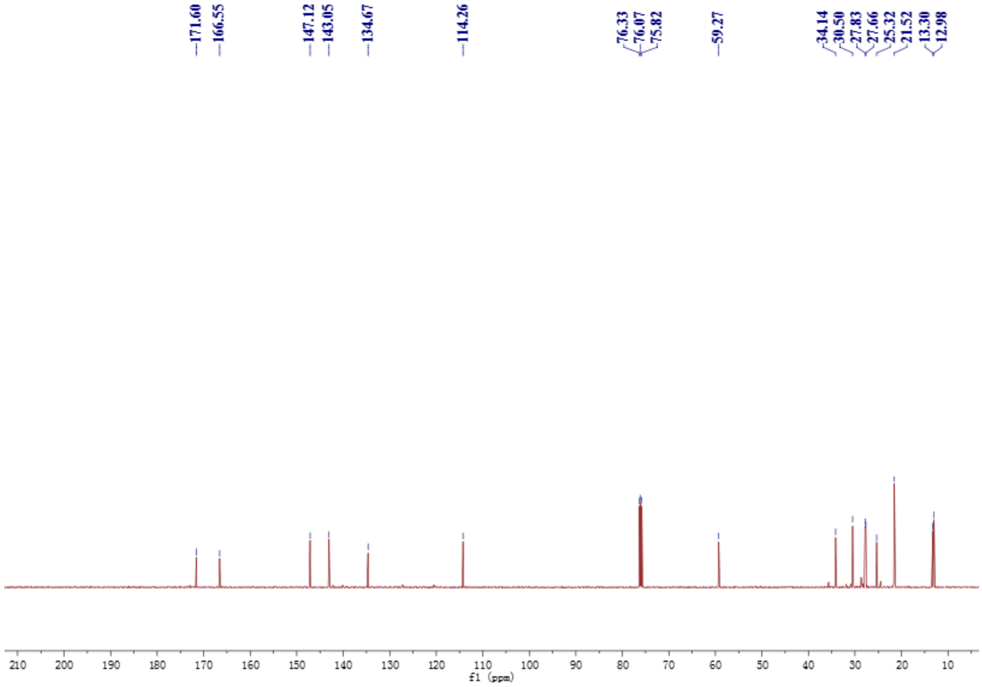


**Supplementary Figure 141.** $^{13}$C NMR spectrum for **7ac** in $CDCl_3$

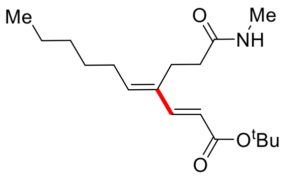


**(7ad)**

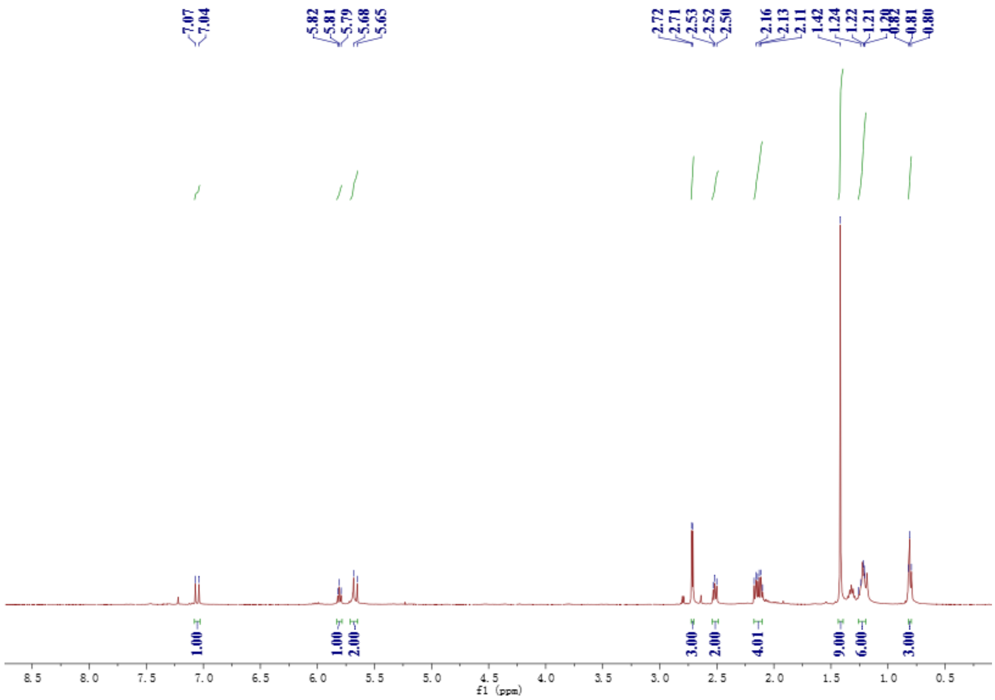

**Supplementary Figure 142.** $^{1}H$ NMR spectrum for **7ad** in $CDCl_3$

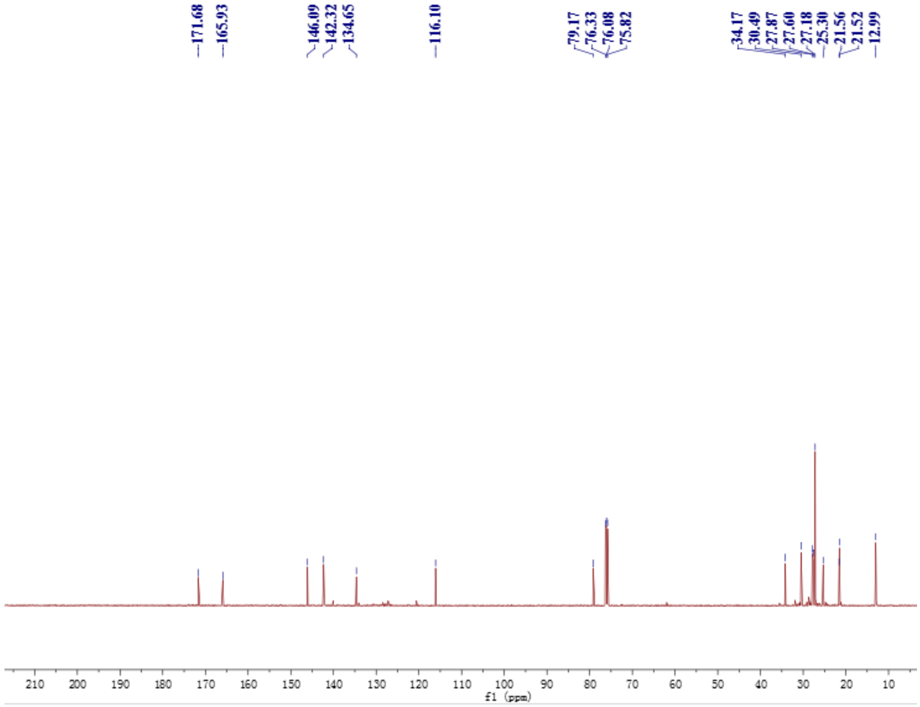

**Supplementary Figure 143.** $^{13}C$ NMR spectrum for **7ad** in $CDCl_3$

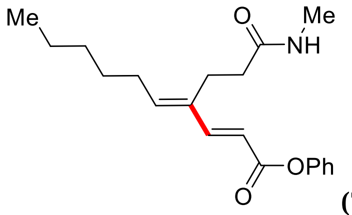


**(7ae)**

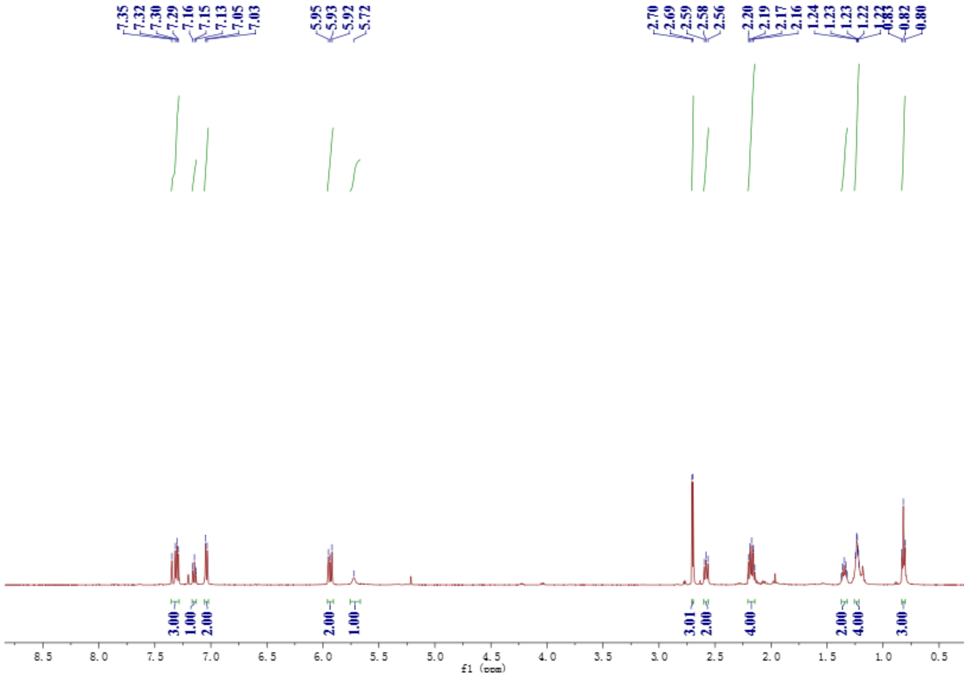


**Supplementary Figure 144.** $^{1}$H NMR spectrum for **7ae** in $CDCl_3$

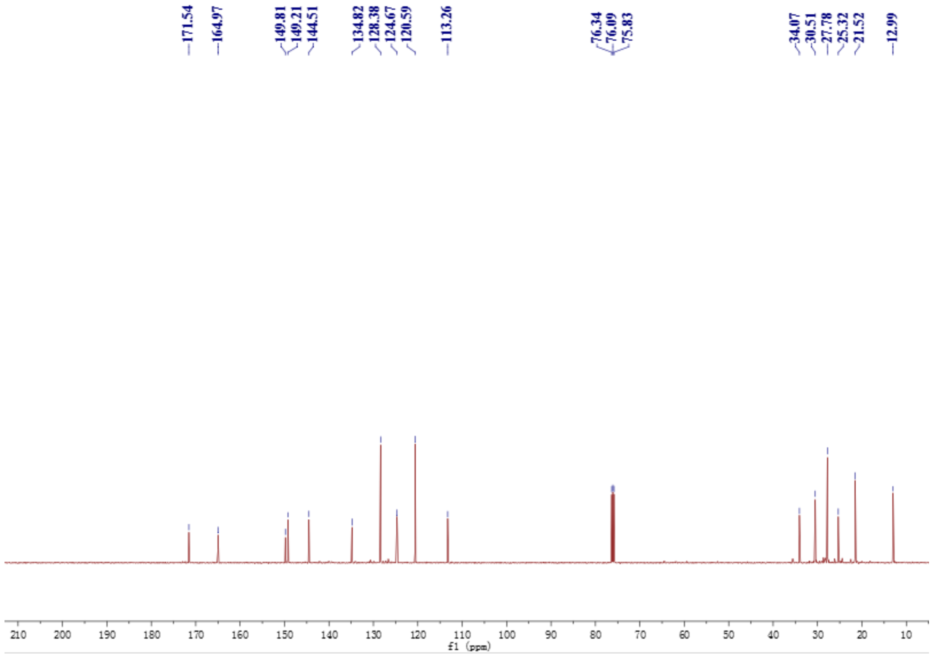


**Supplementary Figure 145.** $^{13}$C NMR spectrum for **7ae** in $CDCl_3$

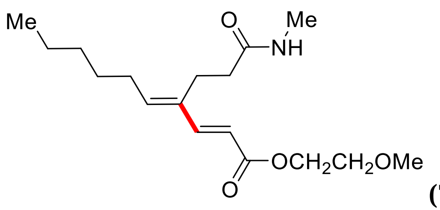


**(7af)**

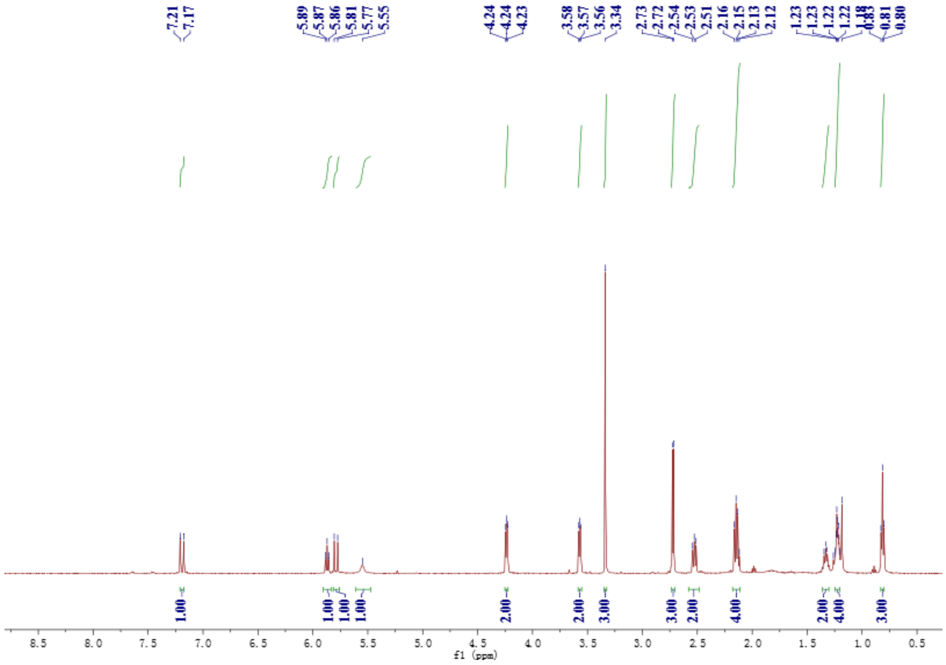

**Supplementary Figure 146.** $^1$H NMR spectrum for **7af** in $CDCl_3$

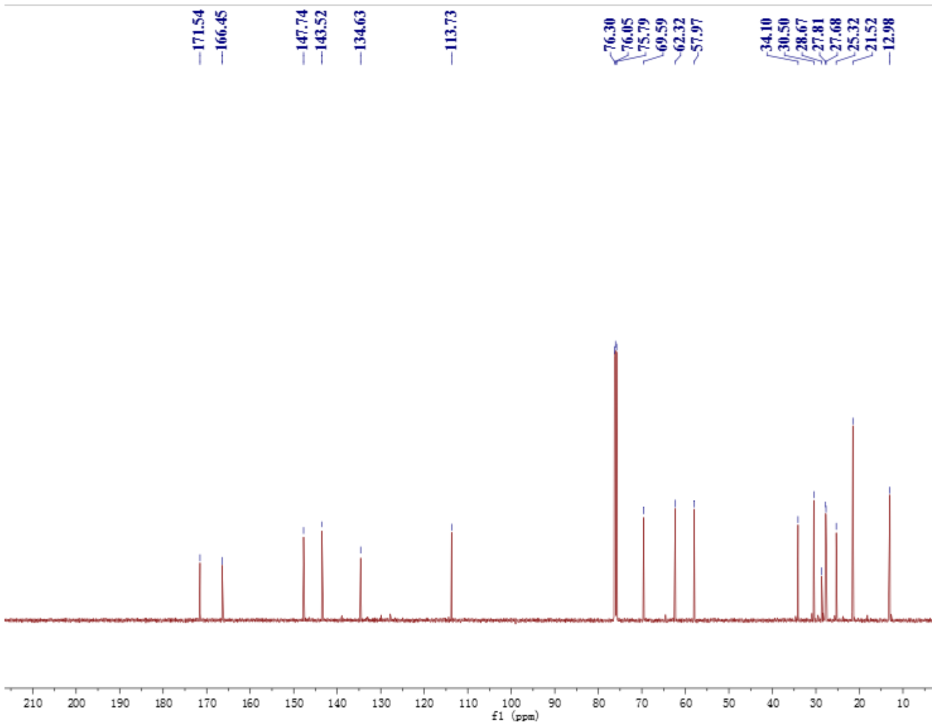

**Supplementary Figure 147.** $^{13}$C NMR spectrum for **7af** in $CDCl_3$

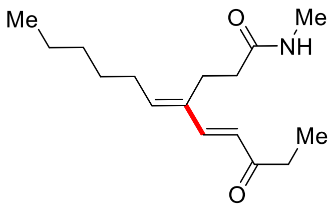


**(7ag)**

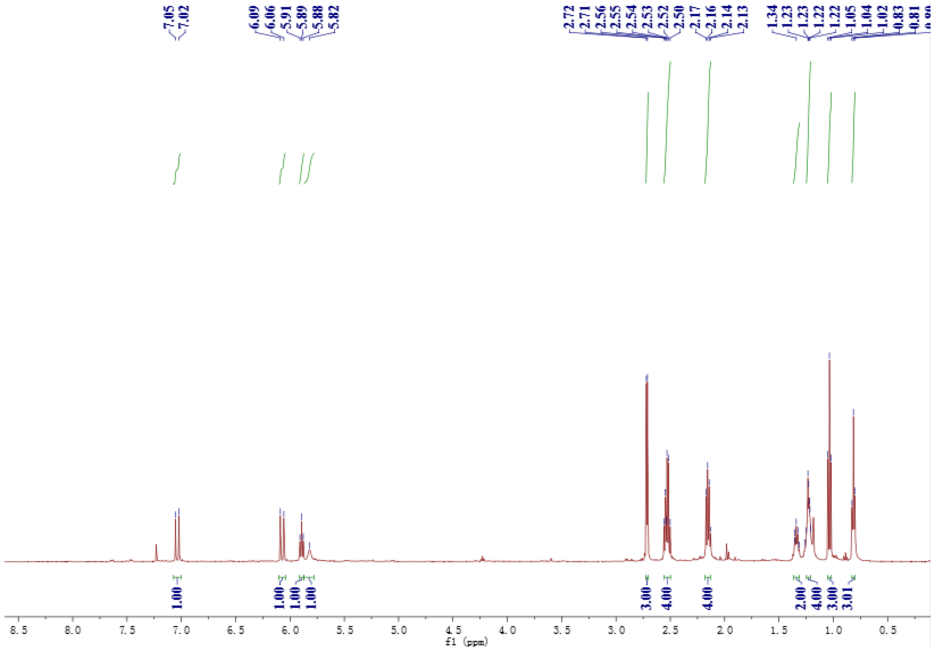


**Supplementary Figure 148.** $^{1}$H NMR spectrum for **7ag** in $CDCl_3$

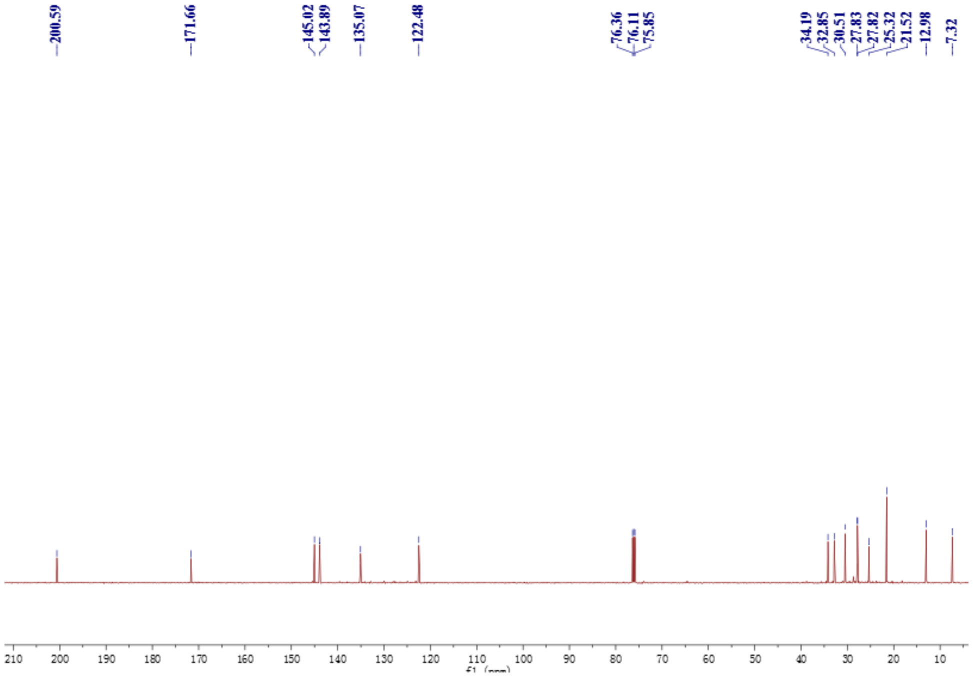


**Supplementary Figure 149.** $^{13}$C NMR spectrum for **7ag** in $CDCl_3$

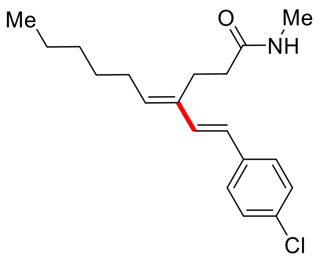


**(7ah)**

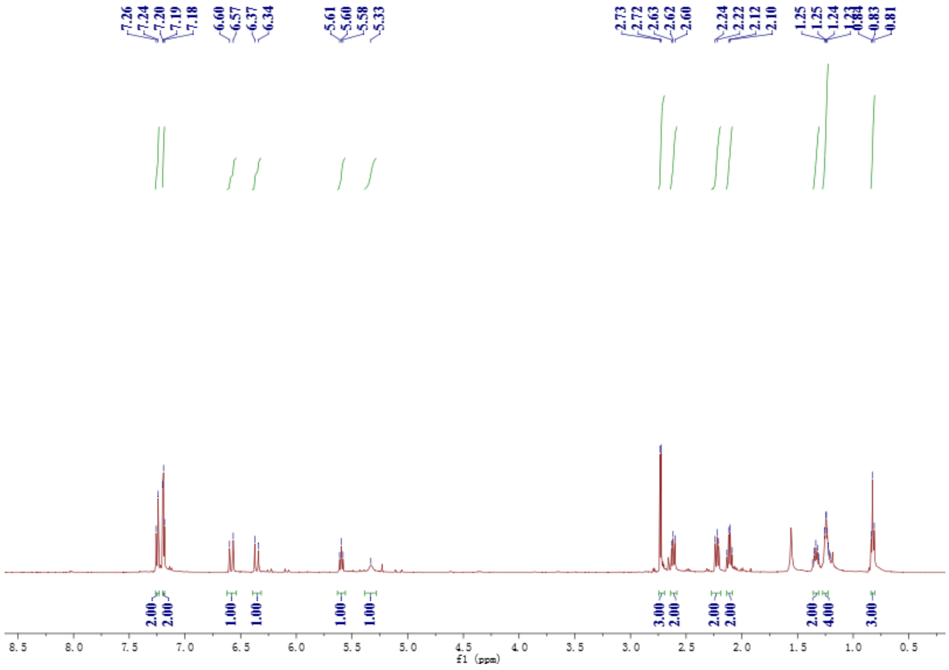


**Supplementary Figure 150.** $^{1}$H NMR spectrum for **7ah** in $CDCl_3$

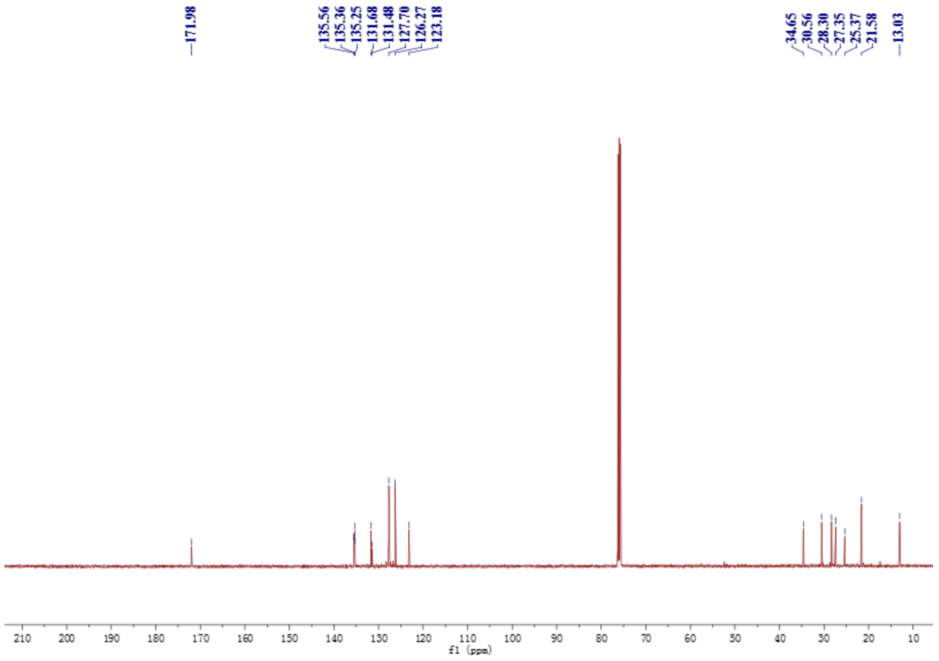


**Supplementary Figure 151.** $^{13}$C NMR spectrum for **7ah** in $CDCl_3$

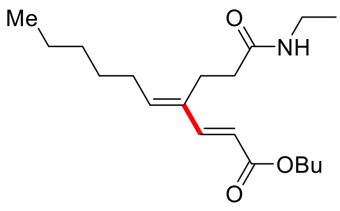


**(7ba)**

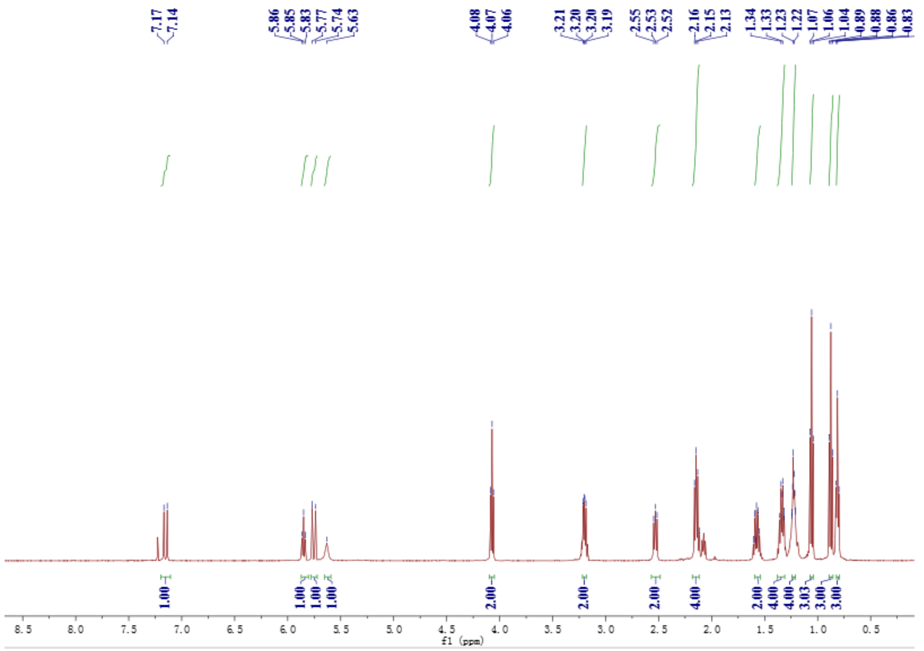

**Supplementary Figure 152.** $^{1}$H NMR spectrum for **7ba** in $CDCl_3$

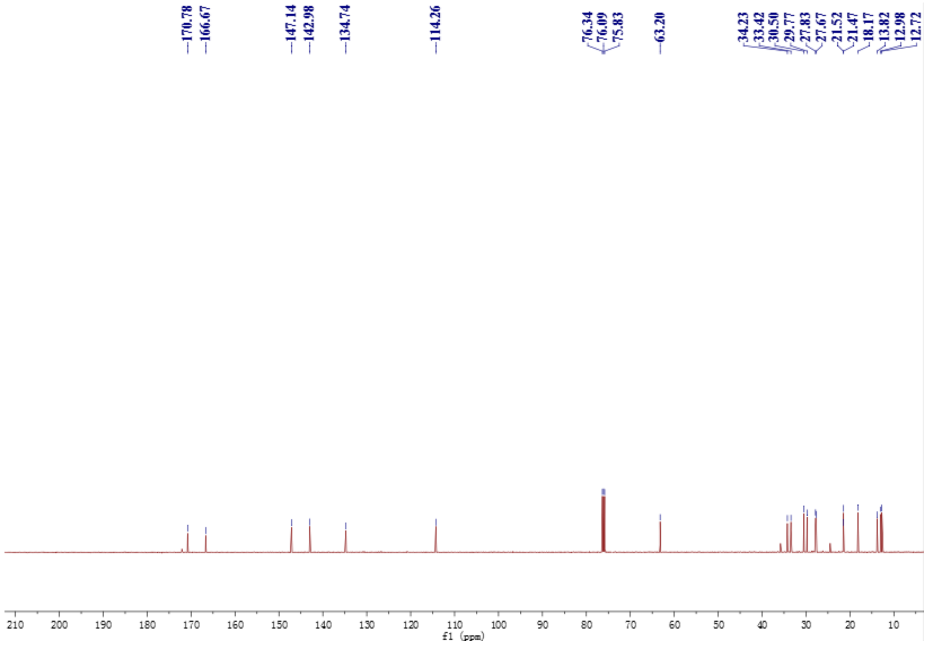

**Supplementary Figure 153.** $^{13}$C NMR spectrum for **7ba** in $CDCl_3$

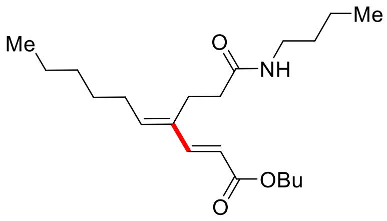


**(7ca)**

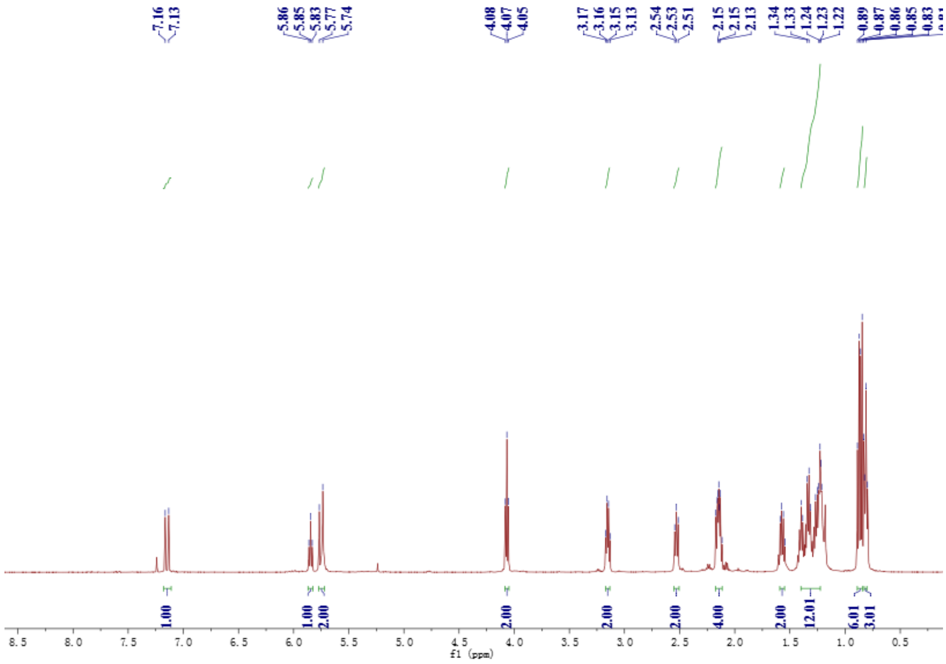


**Supplementary Figure 154.** $^{1}H$ NMR spectrum for **7ca** in $CDCl_3$

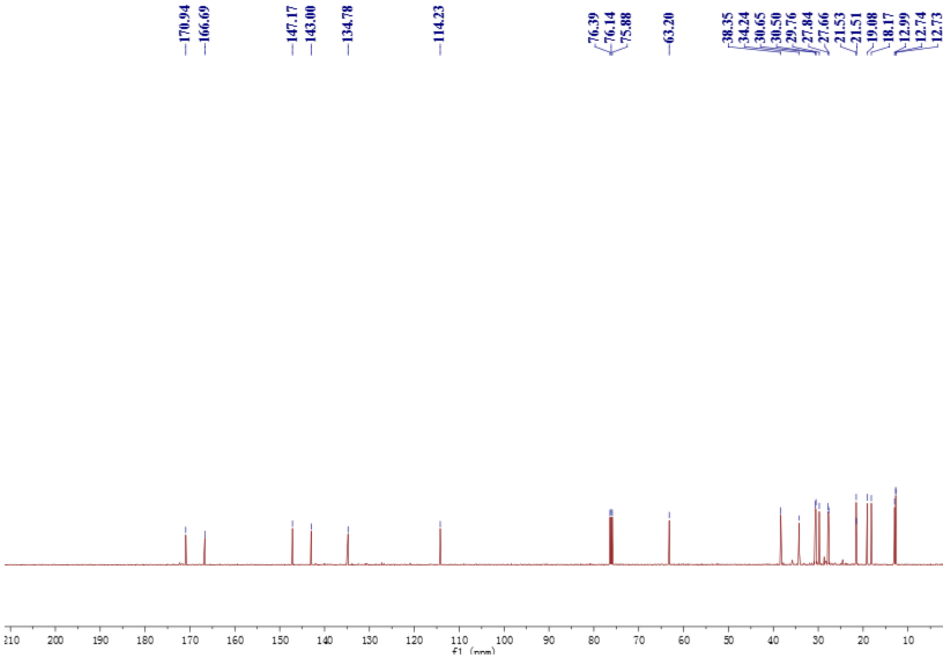


**Supplementary Figure 155.** $^{13}C$ NMR spectrum for **7ca** in $CDCl_3$

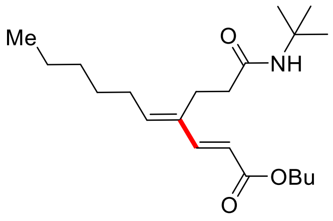


**(7da)**

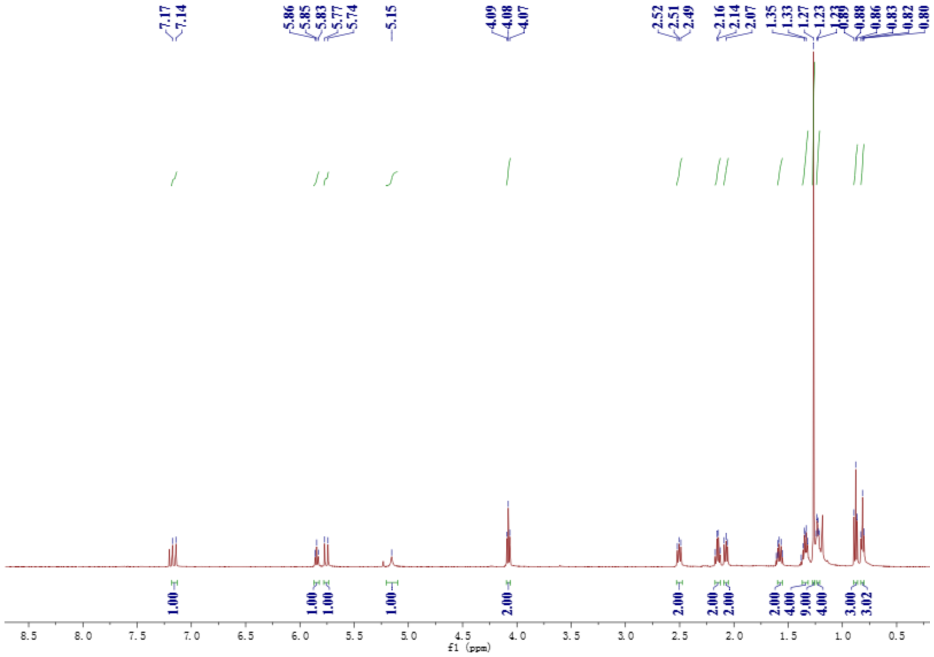


**Supplementary Figure 156.** $^{1}$H NMR spectrum for **7da** in $CDCl_3$

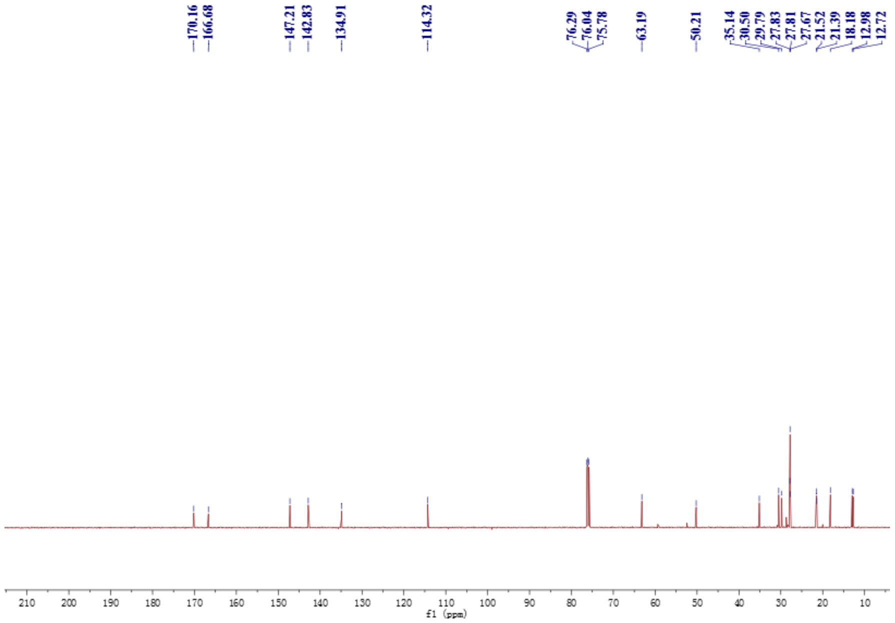


**Supplementary Figure 157.** $^{13}$C NMR spectrum for **7da** in $CDCl_3$

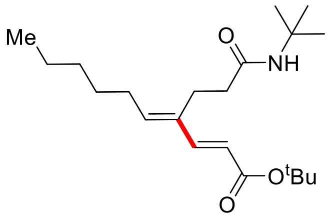


**(7dd)**

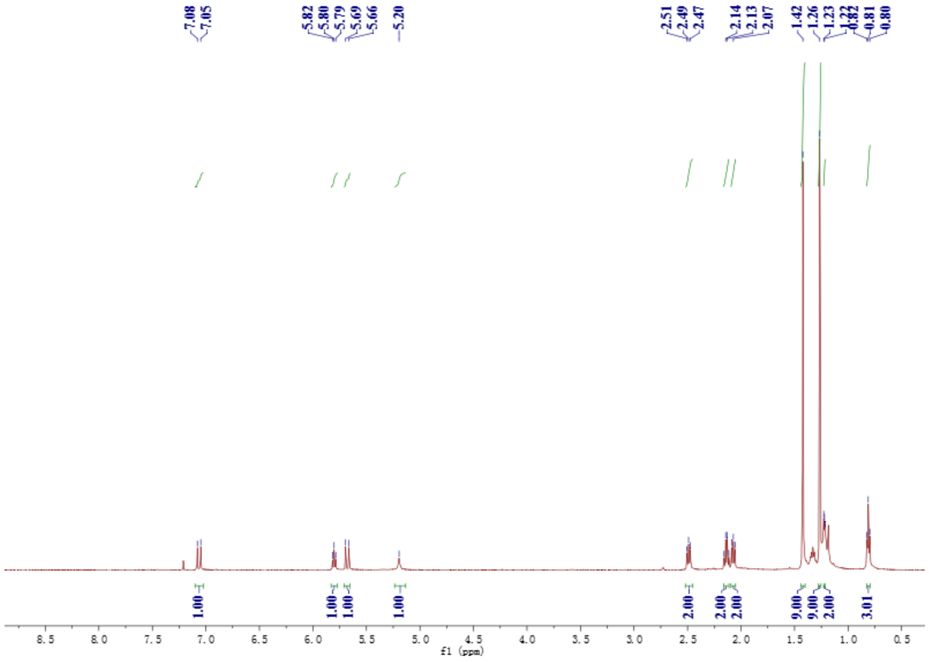

**Supplementary Figure 158.** $^{1}$H NMR spectrum for **7dd** in $CDCl_3$

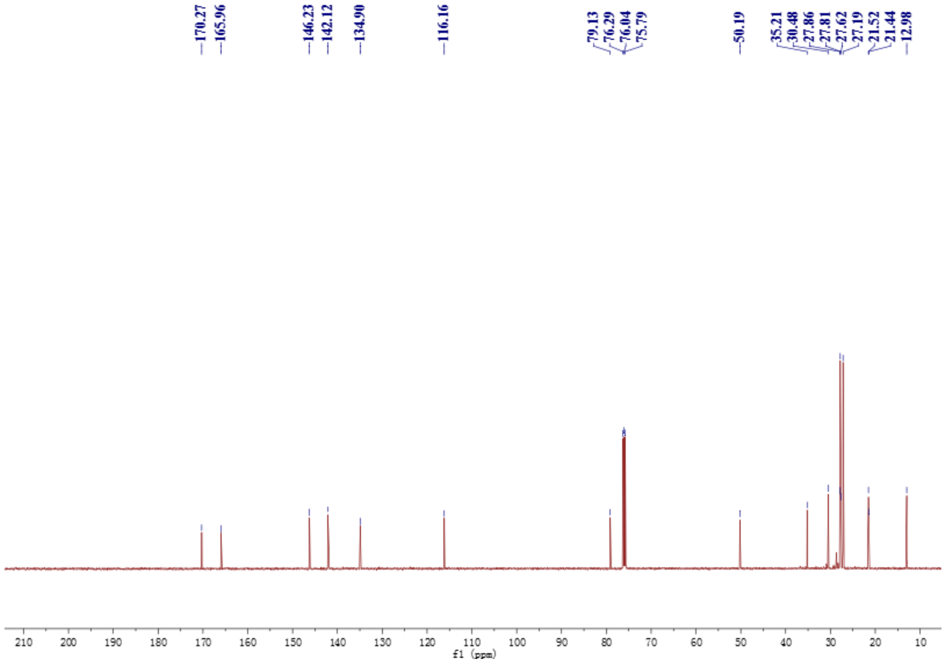

**Supplementary Figure 159.** $^{13}$C NMR spectrum for **7dd** in $CDCl_3$

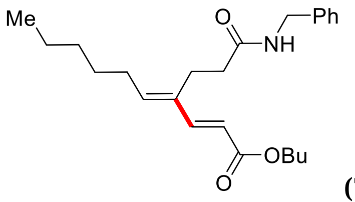


**(7ea)**

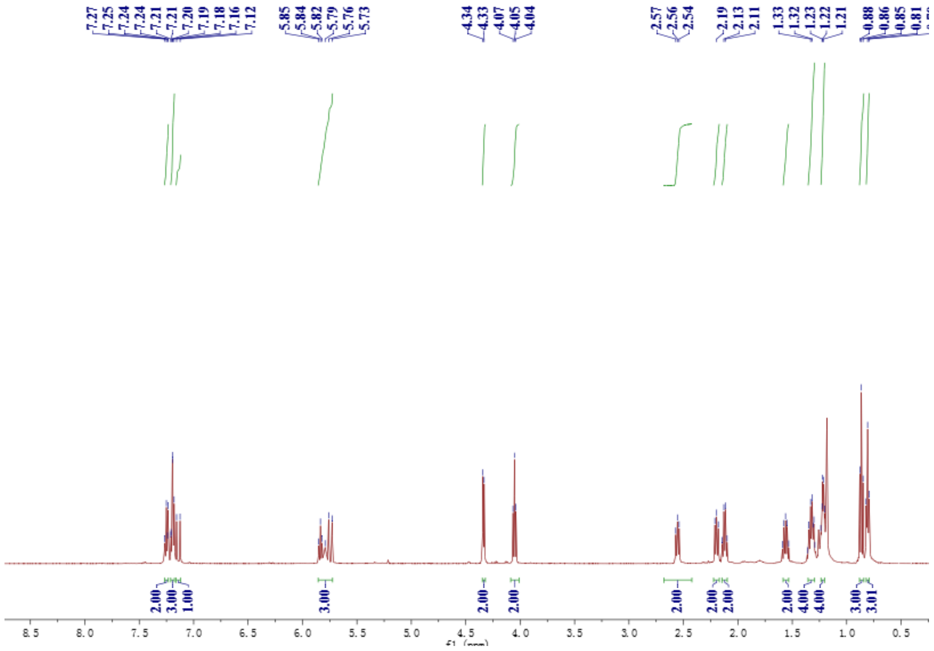

**Supplementary Figure 160.** $^{1}H$ NMR spectrum for **7ea** in $CDCl_3$

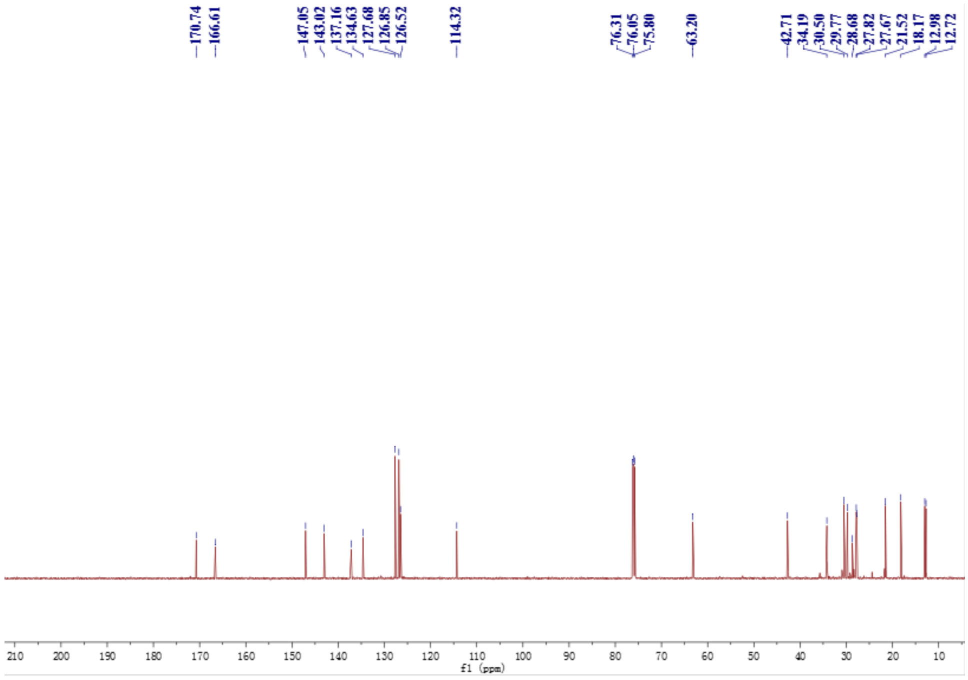

**Supplementary Figure 161.** $^{13}C$ NMR spectrum for **7ea** in $CDCl_3$

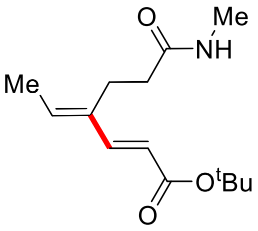


**(7fd)**

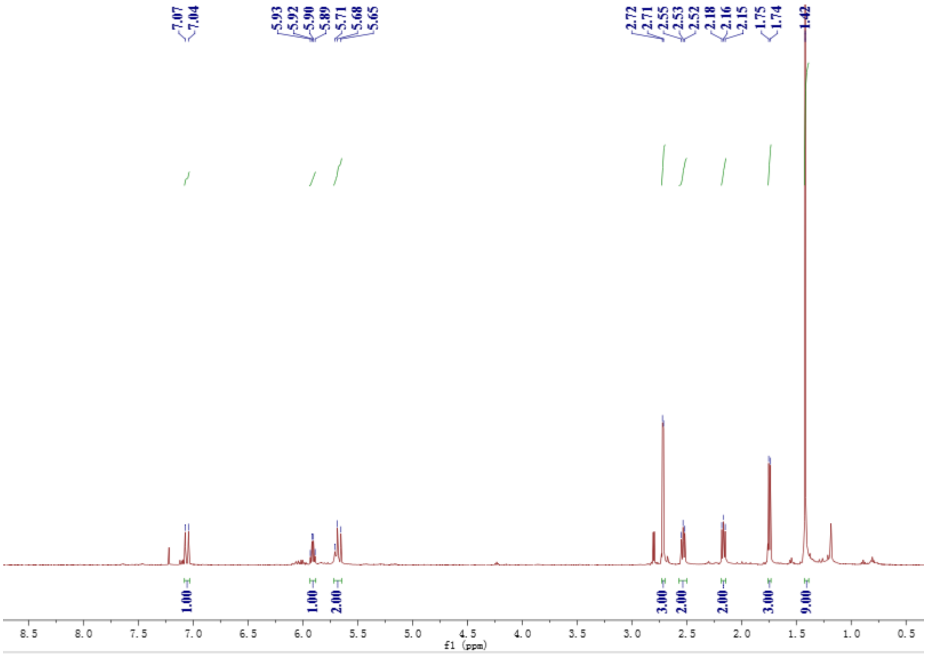


**Supplementary Figure 162.** $^1$H NMR spectrum for **7fd** in $CDCl_3$

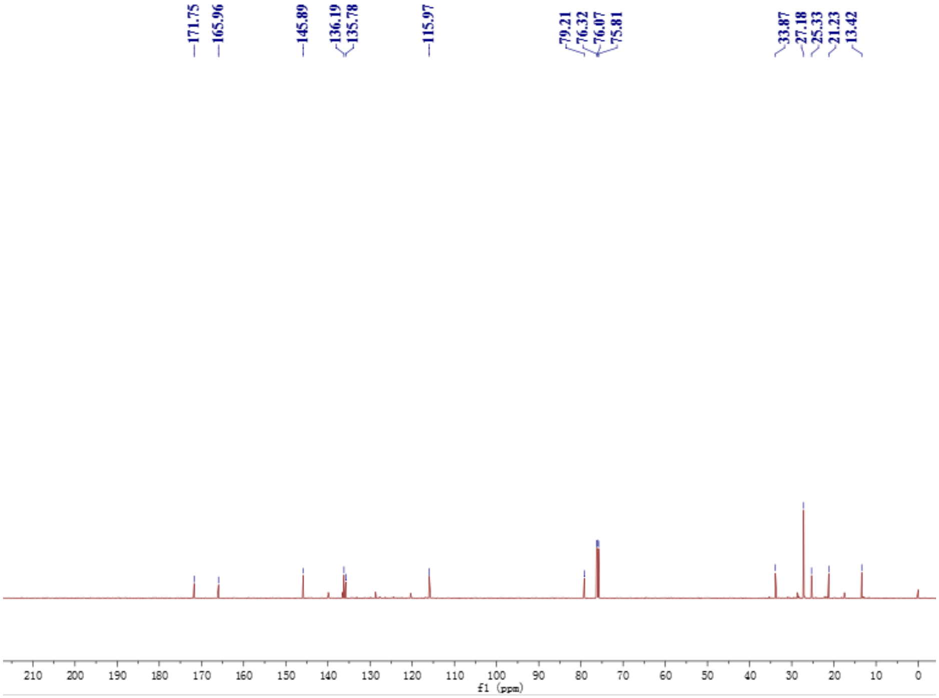


**Supplementary Figure 163.** $^{13}$C NMR spectrum for **7fd** in $CDCl_3$

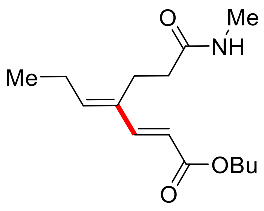


**(7ga)**

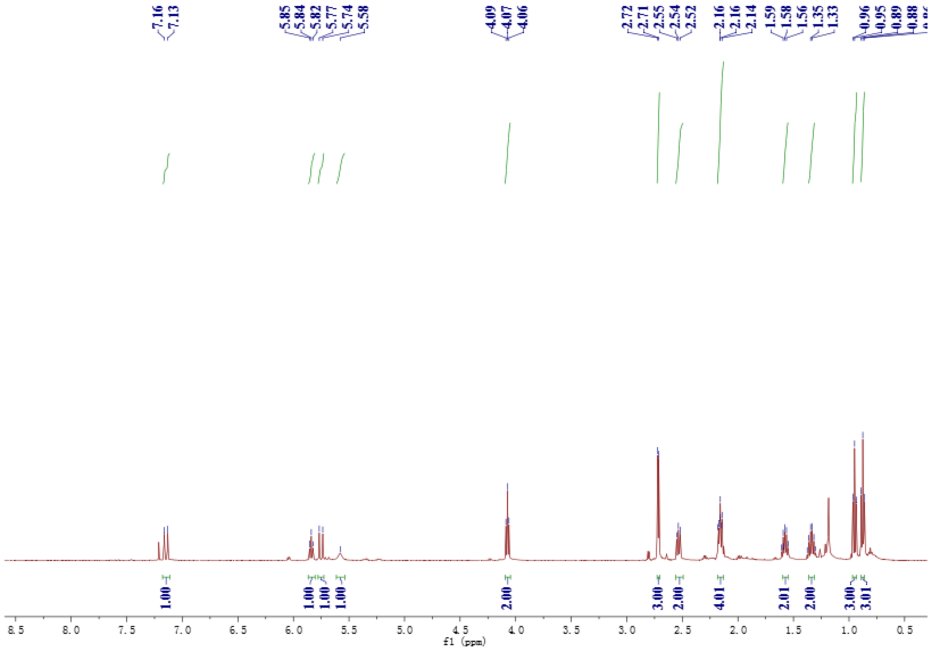


**Supplementary Figure 164.** $^{1}$H NMR spectrum for **7ga** in $CDCl_3$

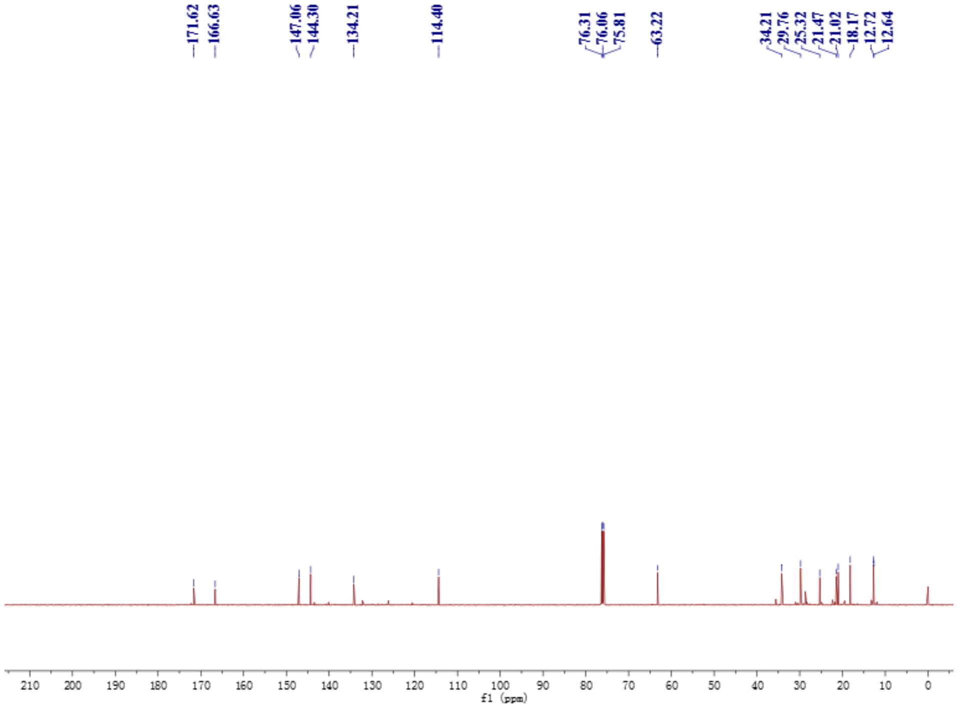


**Supplementary Figure 165.** $^{13}$C NMR spectrum for **7ga** in $CDCl_3$

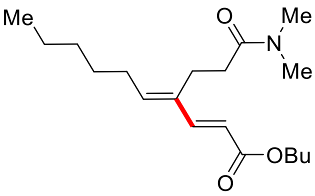


**(7ha)**

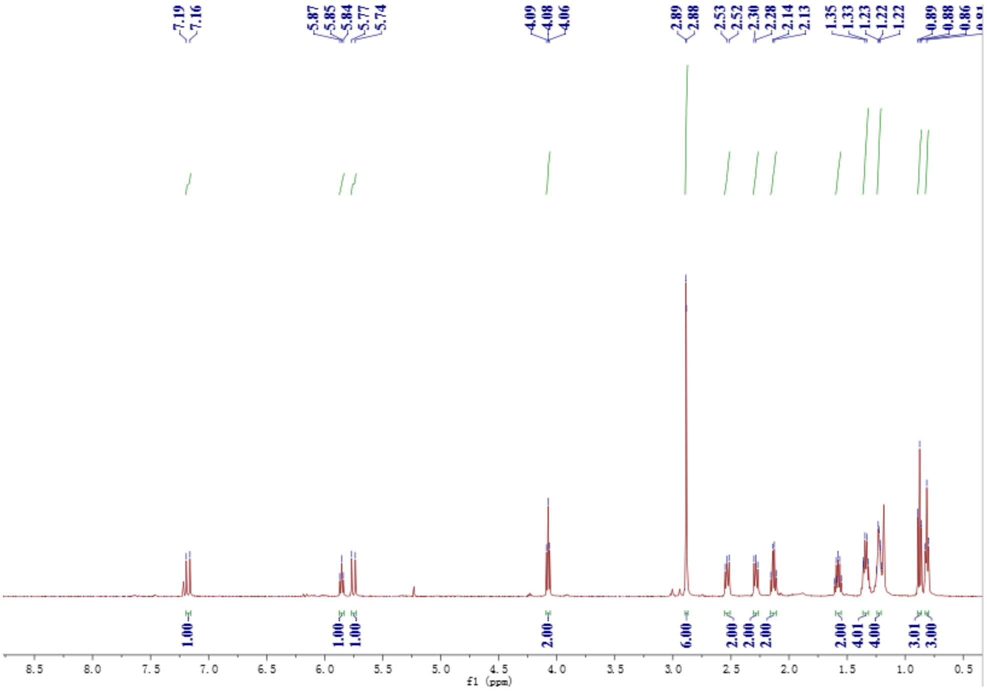


**Supplementary Figure 166.** $^{1}$H NMR spectrum for **7ha** in $CDCl_3$

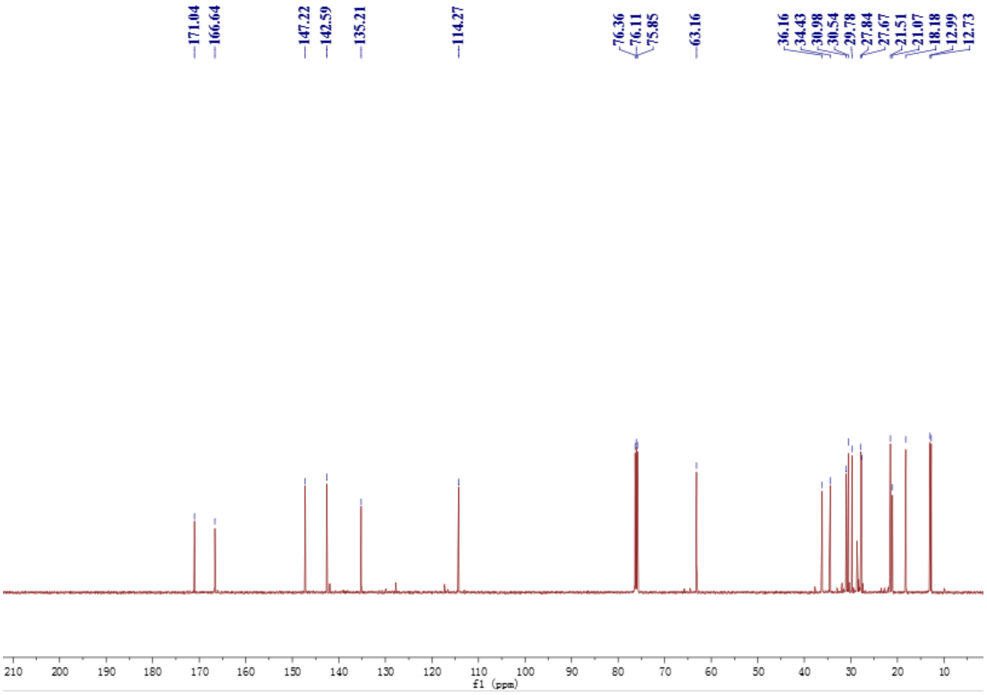


**Supplementary Figure 167.** $^{13}$C NMR spectrum for **7ha** in $CDCl_3$

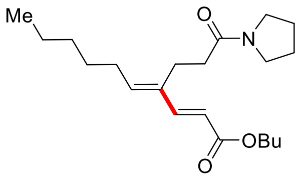


**(7ia)**

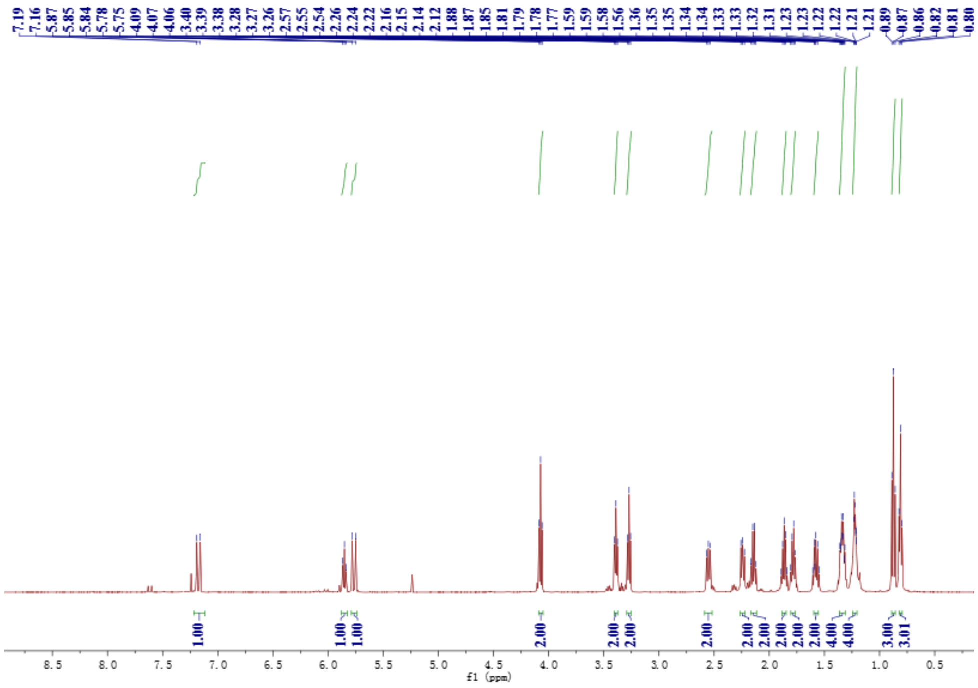


**Supplementary Figure 168.** $^{1}H$ NMR spectrum for **7ia** in $CDCl_3$

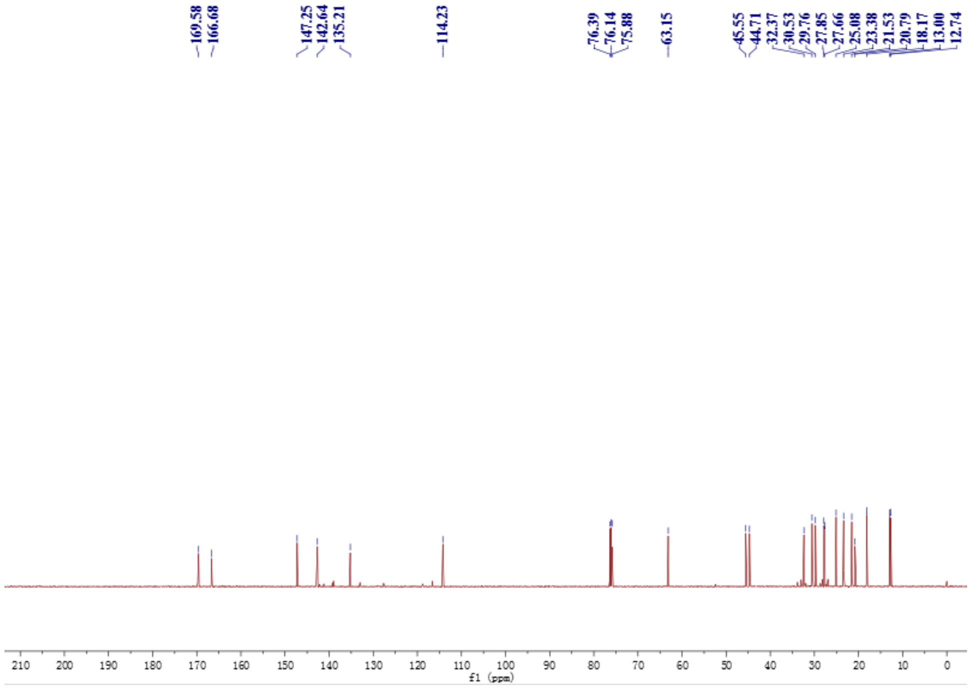


**Supplementary Figure 169.** $^{13}C$ NMR spectrum for **7ia** in $CDCl_3$

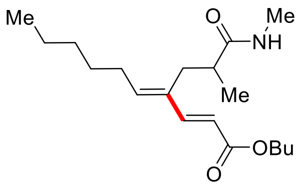


**(7ja)**

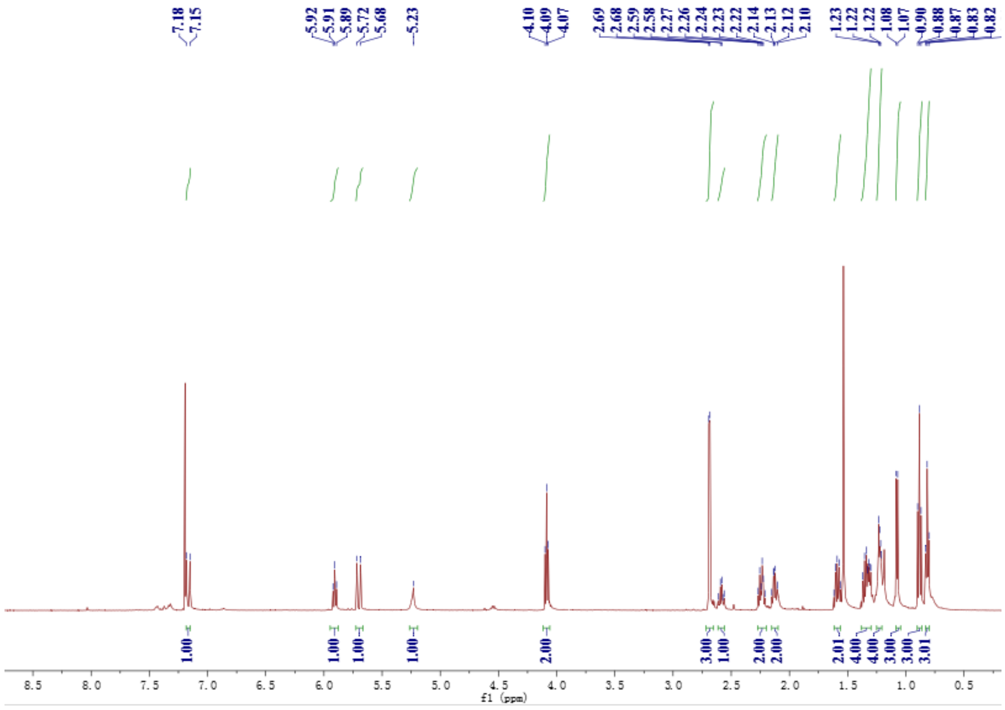


**Supplementary Figure 170.** $^{1}$H NMR spectrum for **7ja** in $CDCl_3$

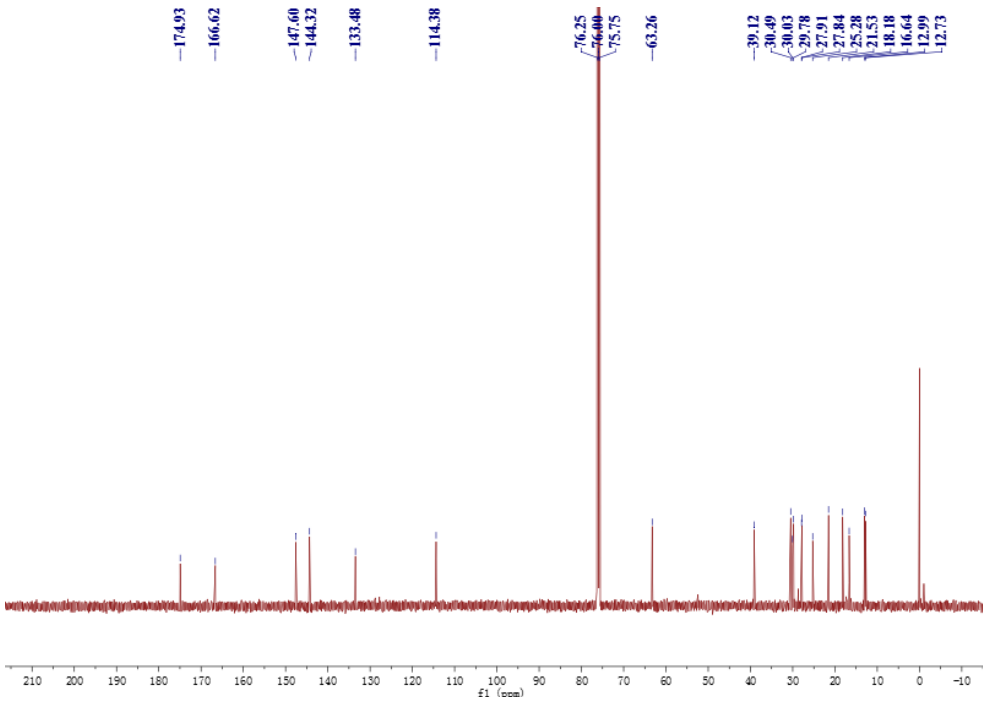


**Supplementary Figure 171.** $^{13}$C NMR spectrum for **7ja** in $CDCl_3$

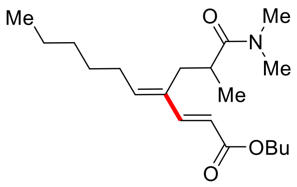


**(7ka)**

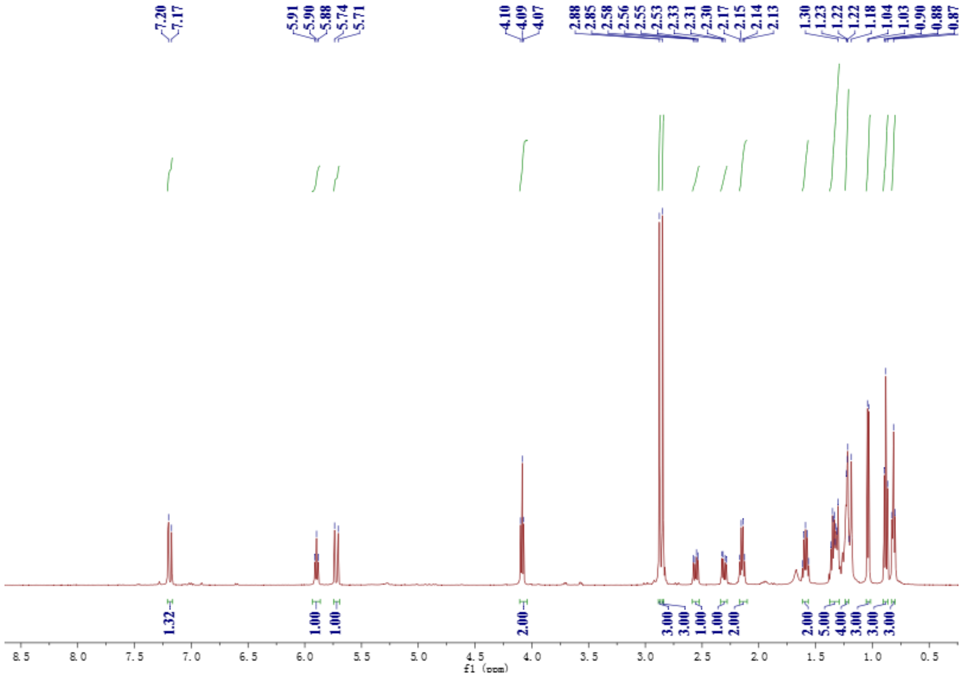


**Supplementary Figure 172.** $^{1}H$ NMR spectrum for **7ka** in $CDCl_3$

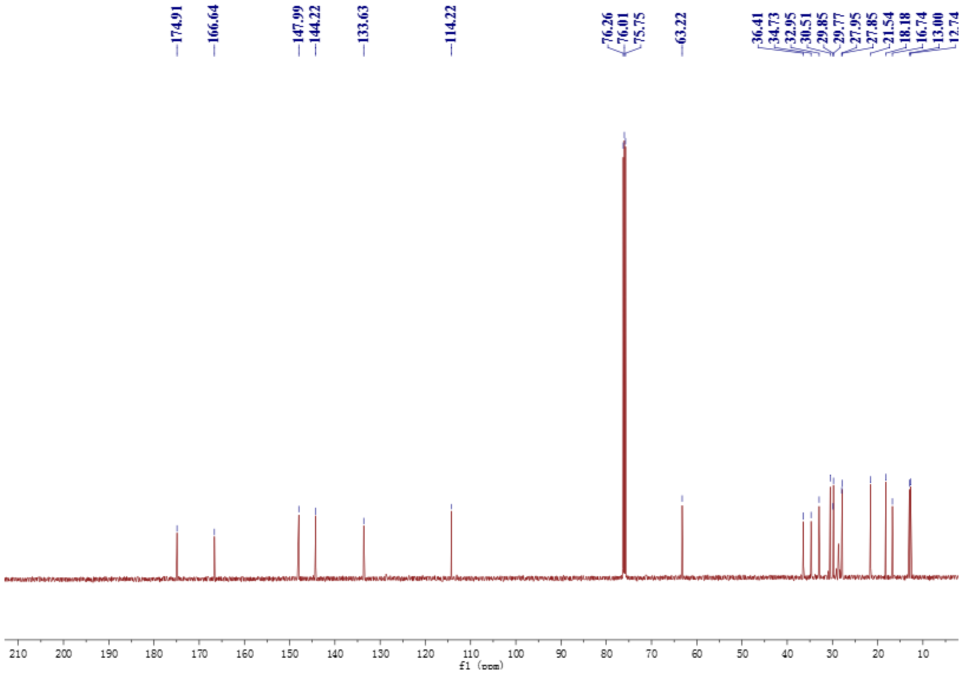


**Supplementary Figure 173.** $^{13}C$ NMR spectrum for **7ka** in $CDCl_3$

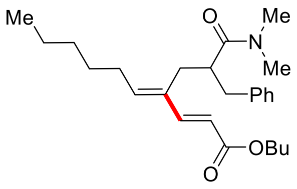


**(7la)**

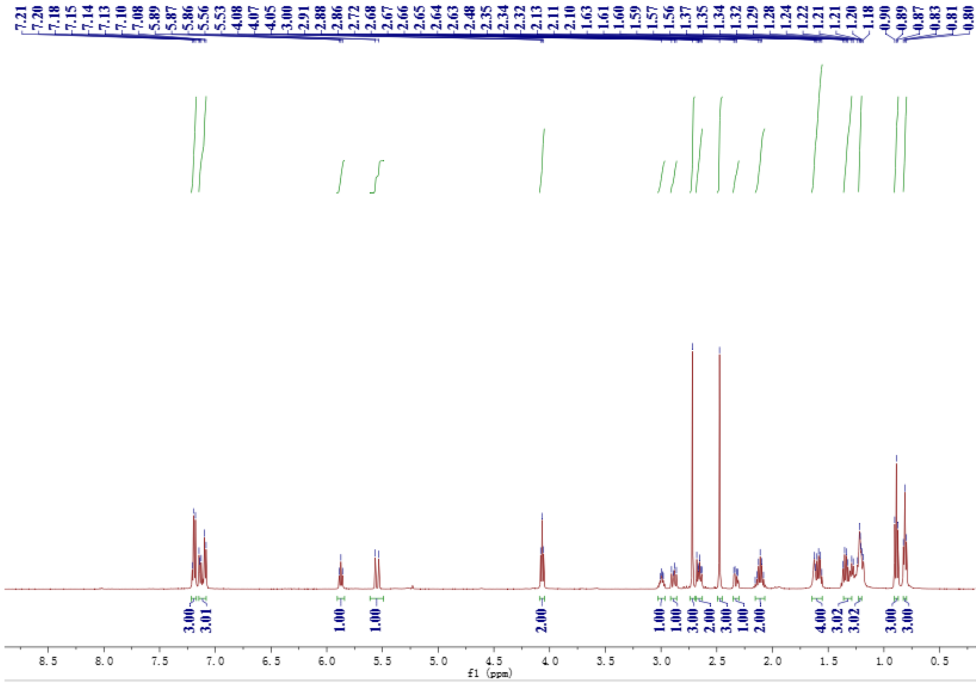


**Supplementary Figure 174.** $^{1}$H NMR spectrum for **7la** in $CDCl_3$

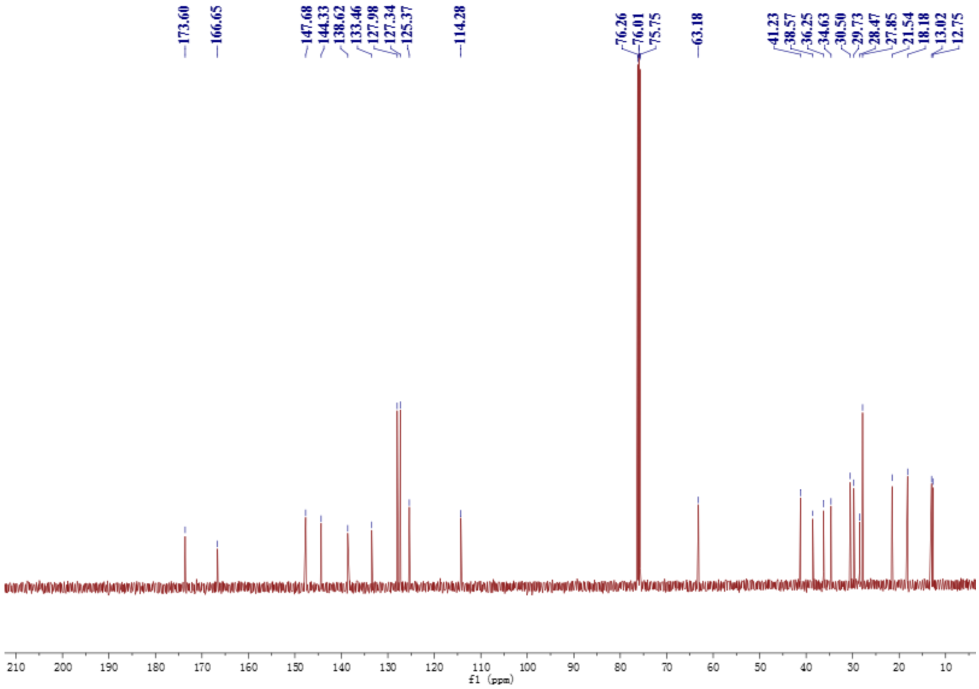


**Supplementary Figure 175.** $^{13}$C NMR spectrum for **7la** in $CDCl_3$

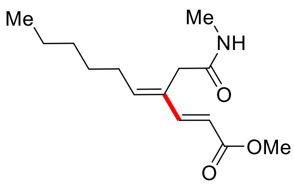


**(7ma)**

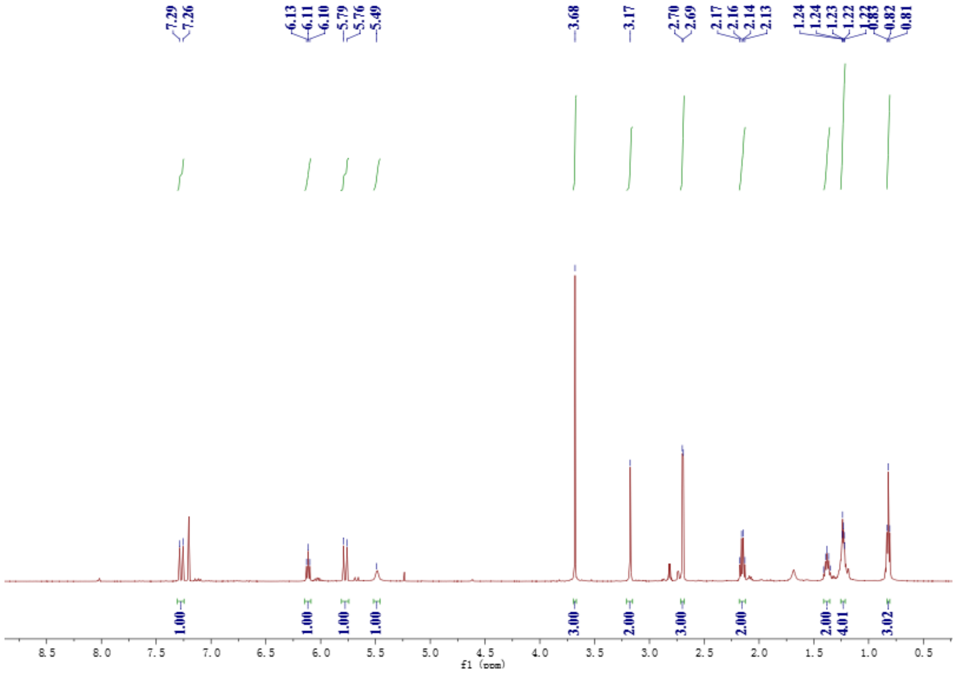


**Supplementary Figure 176.** $^{1}$H NMR spectrum for **7ma** in $CDCl_3$

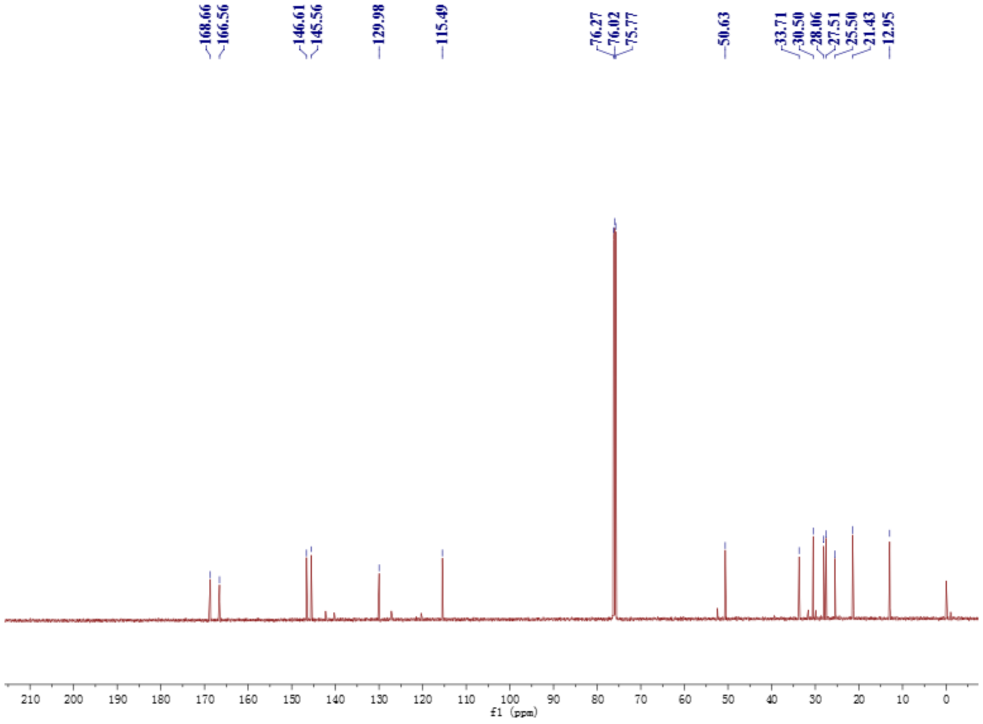


**Supplementary Figure 177.** $^{13}$C NMR spectrum for **7ma** in $CDCl_3$

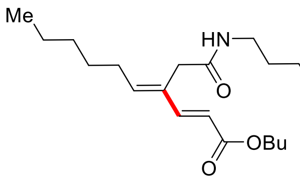


**(7na)**

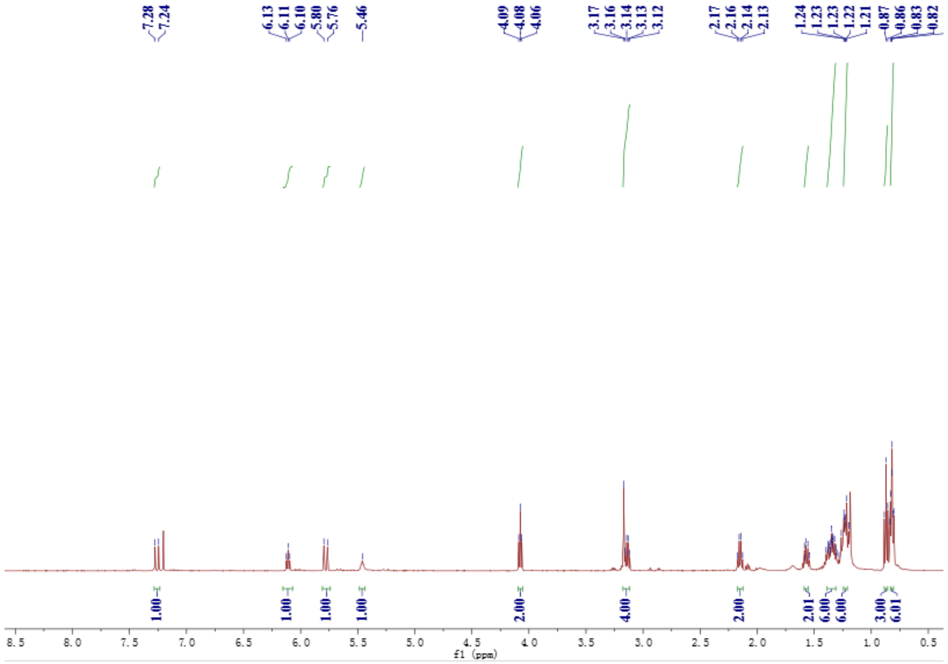


**Supplementary Figure 178.** $^{1}$H NMR spectrum for **7na** in $CDCl_3$

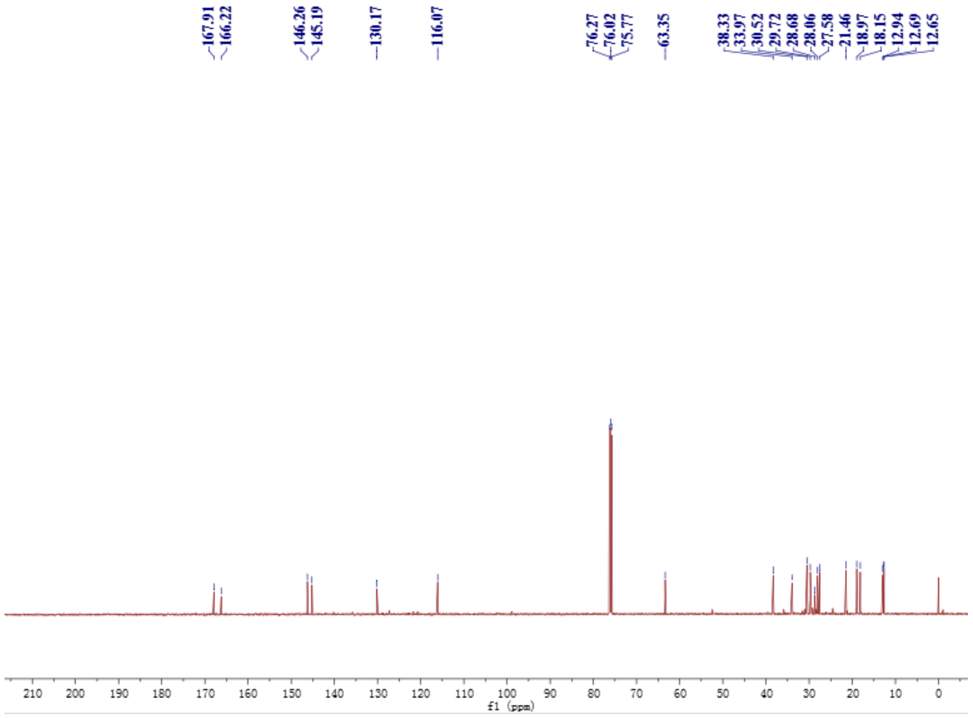


**Supplementary Figure 179.** $^{13}$C NMR spectrum for **7na** in $CDCl_3$

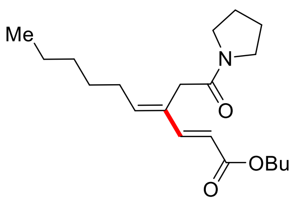


**(7oa)**

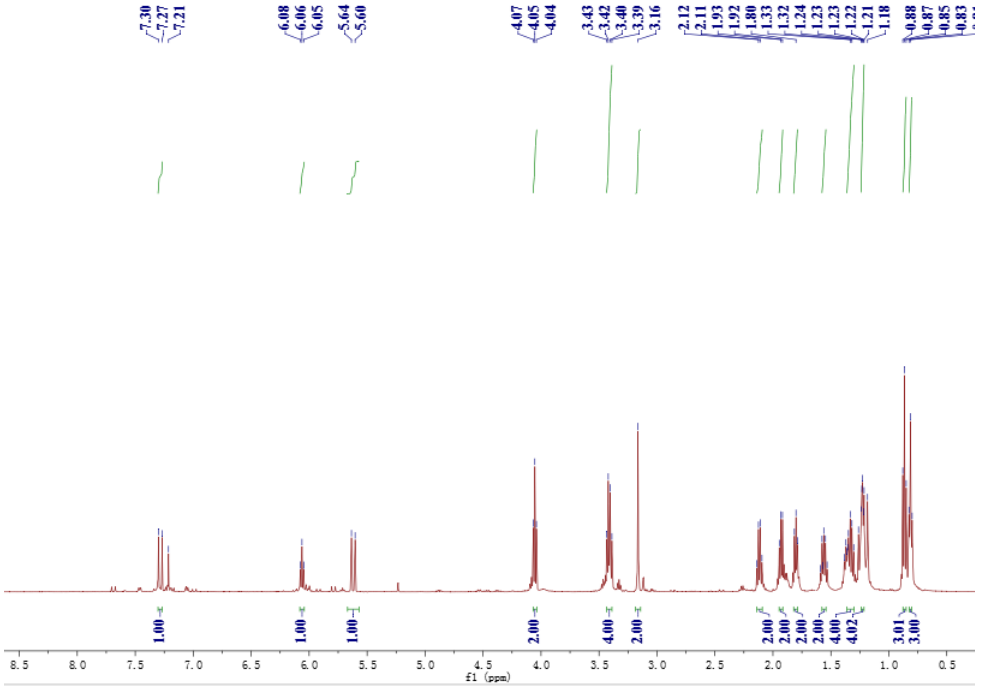


**Supplementary Figure 180.** $^{1}H$ NMR spectrum for **7oa** in $CDCl_3$

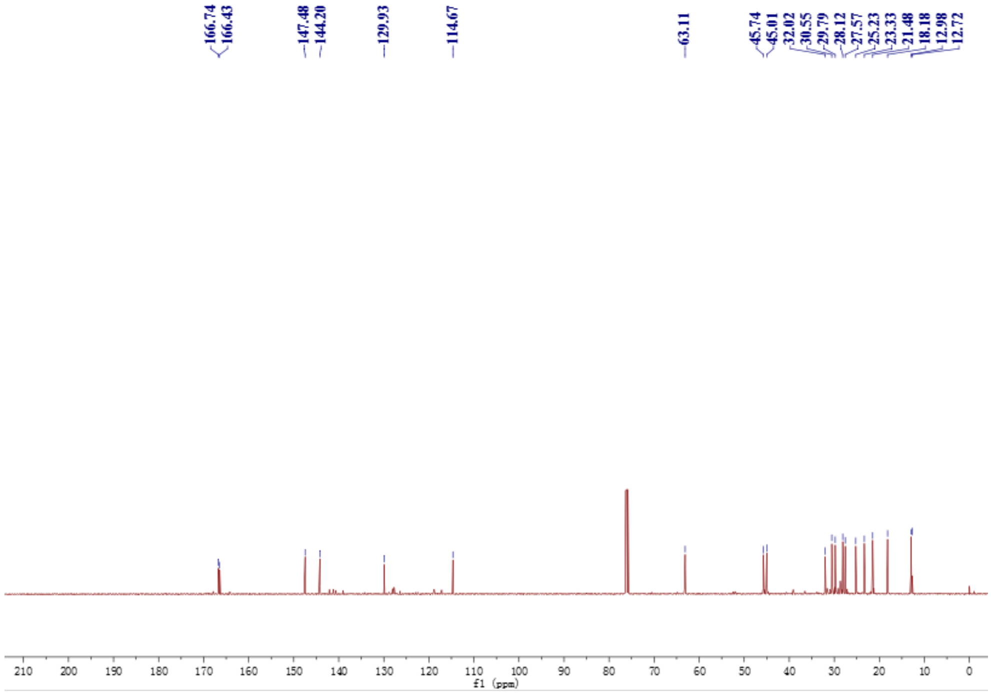


**Supplementary Figure 181.** $^{13}C$ NMR spectrum for **7oa** in $CDCl_3$

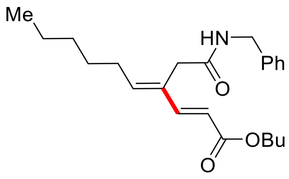


**(7pa)**

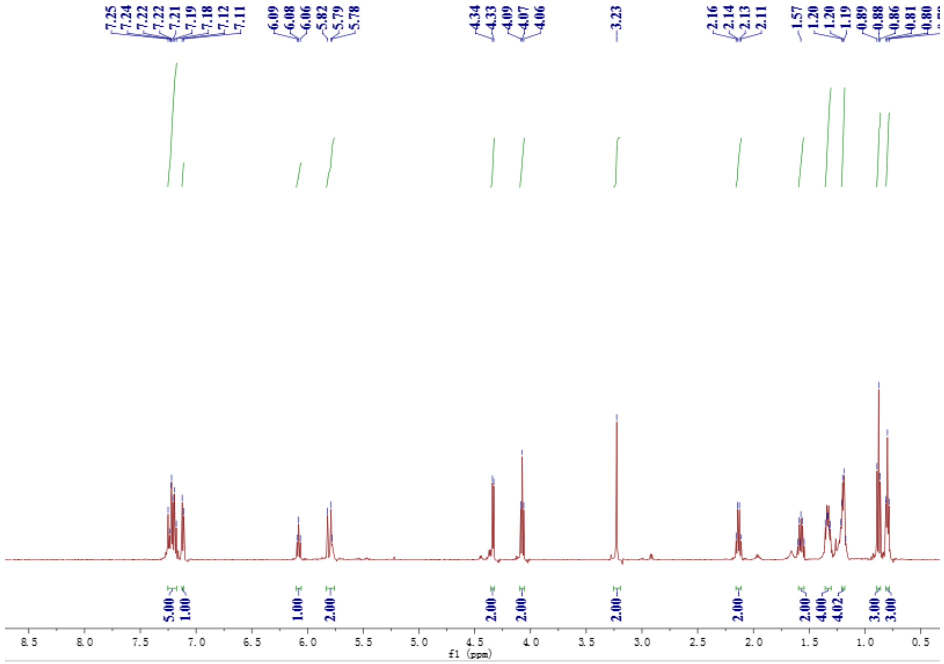


**Supplementary Figure 182.** $^{1}H$ NMR spectrum for **7pa** in $CDCl_3$

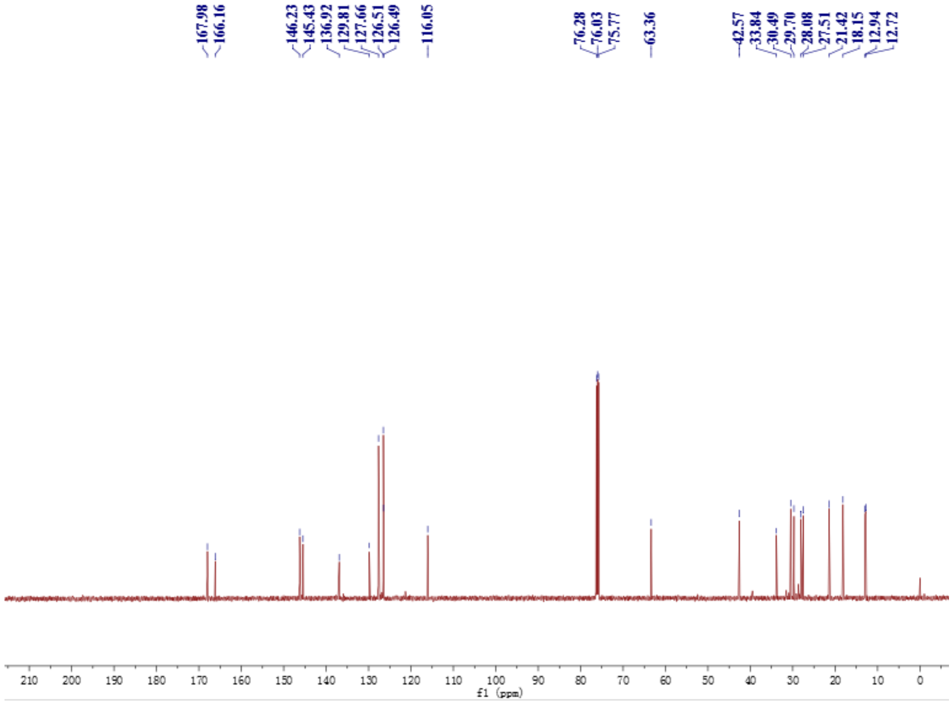


**Supplementary Figure 183.** $^{13}C$ NMR spectrum for **7pa** in $CDCl_3$

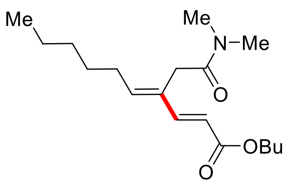


**(7qa)**

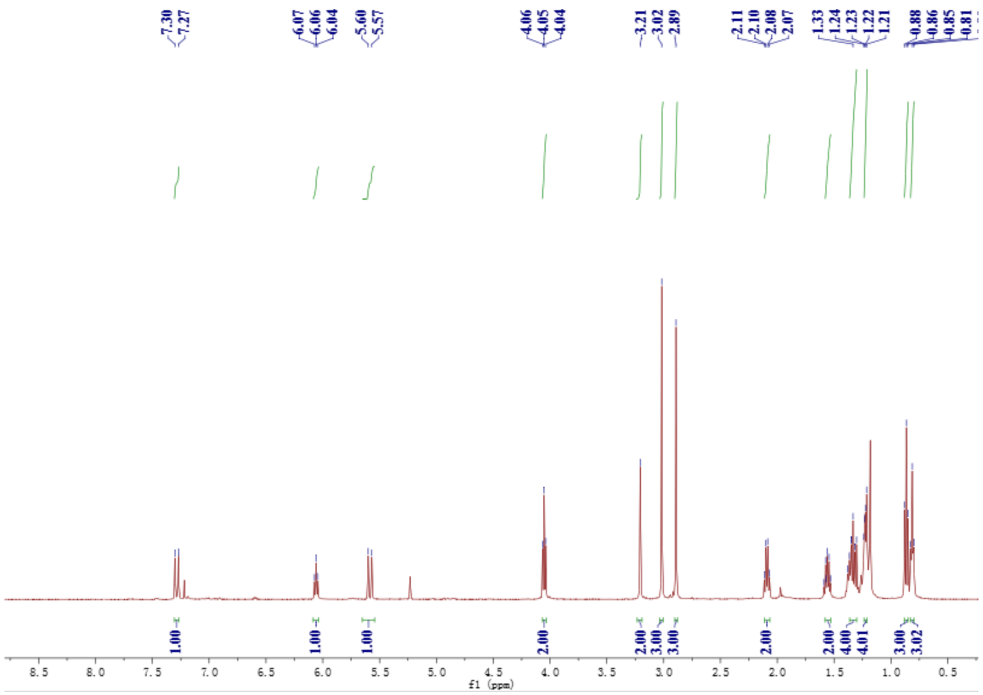

**Supplementary Figure 184.** $^{1}H$ NMR spectrum for **7qa** in $CDCl_3$

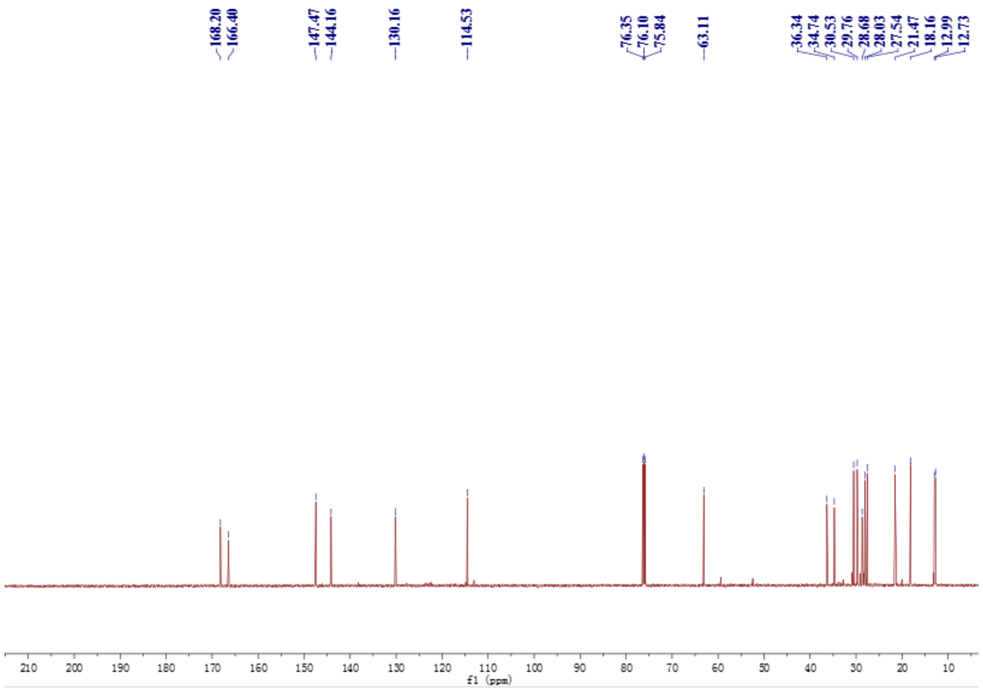

**Supplementary Figure 185.** $^{13}C$ NMR spectrum for **7qa** in $CDCl_3$

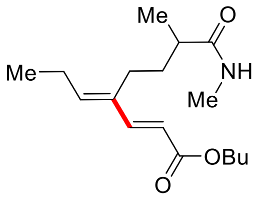


**(7ra)**

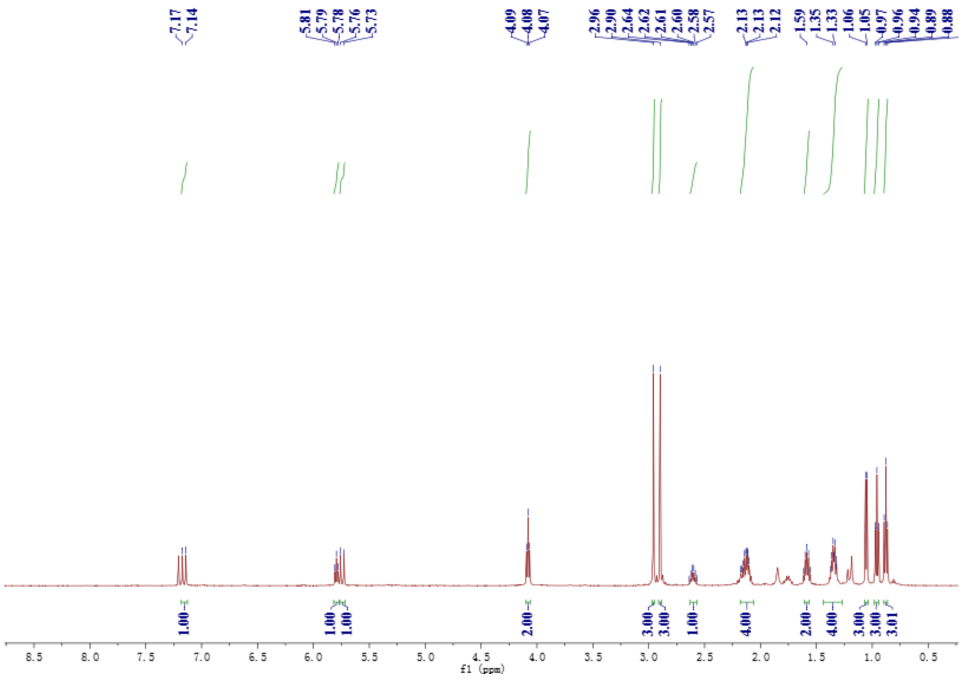

**Supplementary Figure 186.** $^{1}$H NMR spectrum for **7ra** in $CDCl_3$

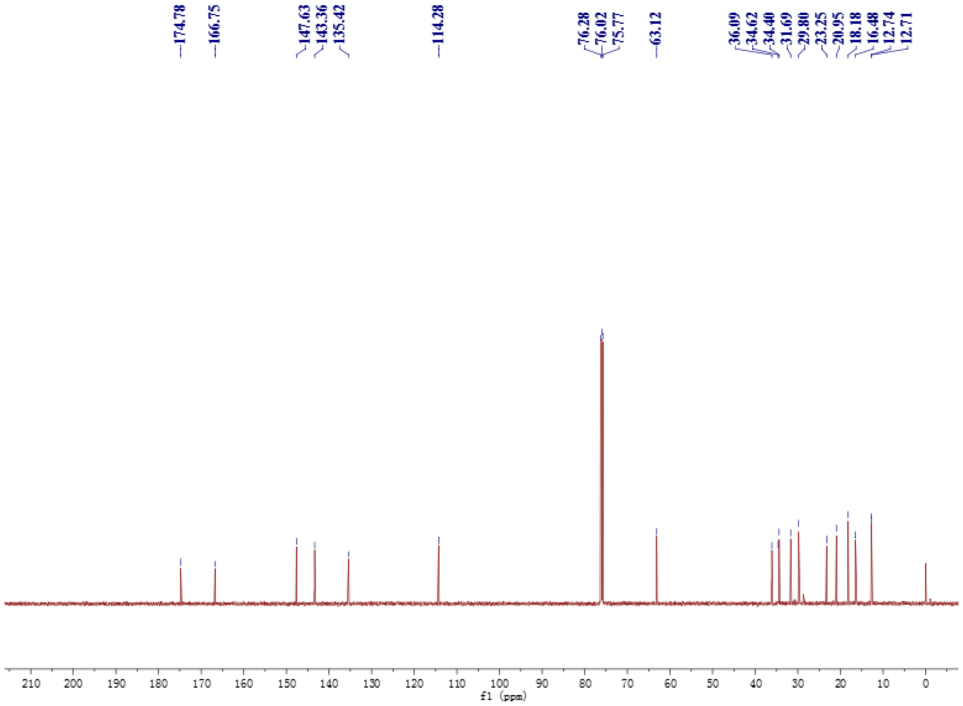

**Supplementary Figure 187.** $^{13}$C NMR spectrum for **7ra** in $CDCl_3$

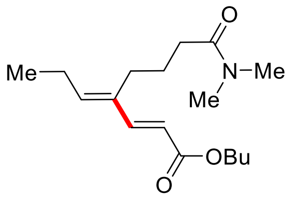


**(7sa)**

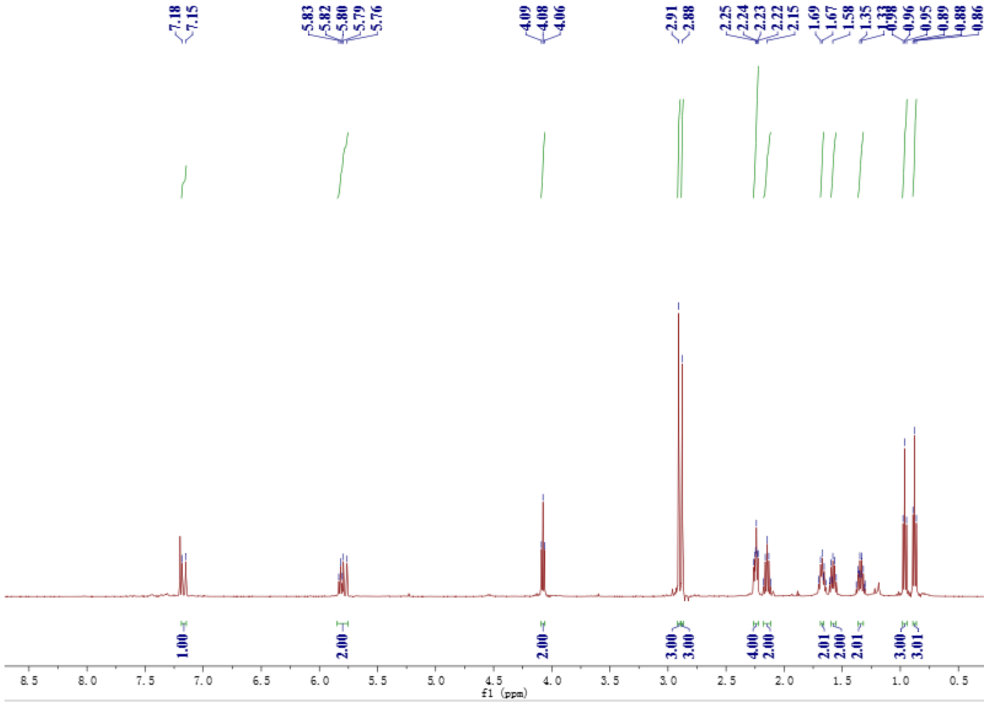

**Supplementary Figure 188.** $^{1}$H NMR spectrum for **7sa** in $CDCl_3$

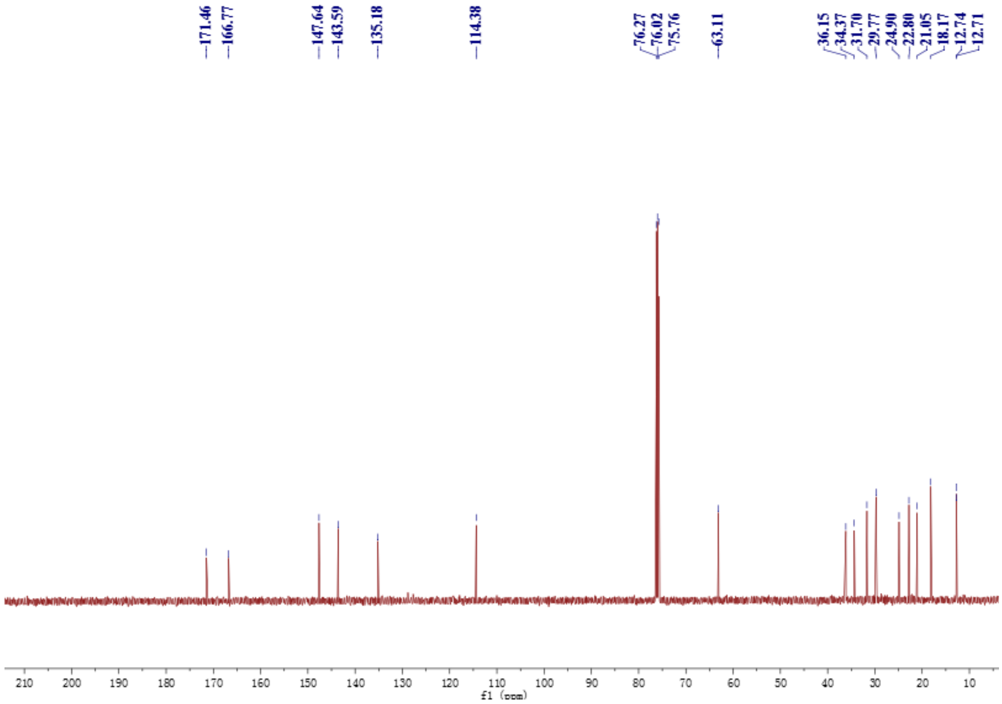

**Supplementary Figure 189.** $^{13}$C NMR spectrum for **7sa** in $CDCl_3$

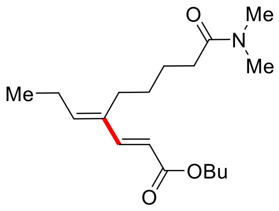


**(7ta)**

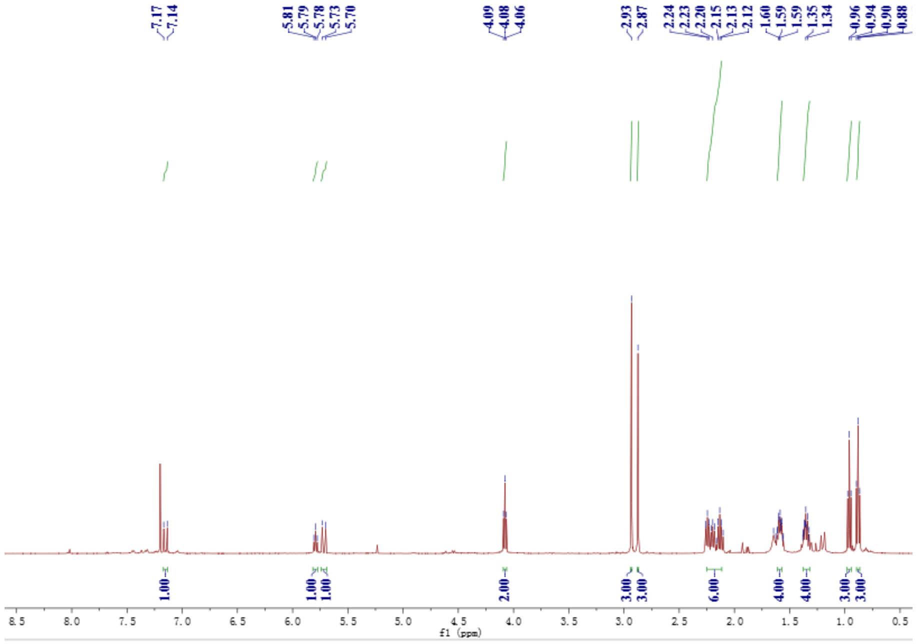


**Supplementary Figure 190.** $^{1}$H NMR spectrum for **7ta** in $CDCl_3$

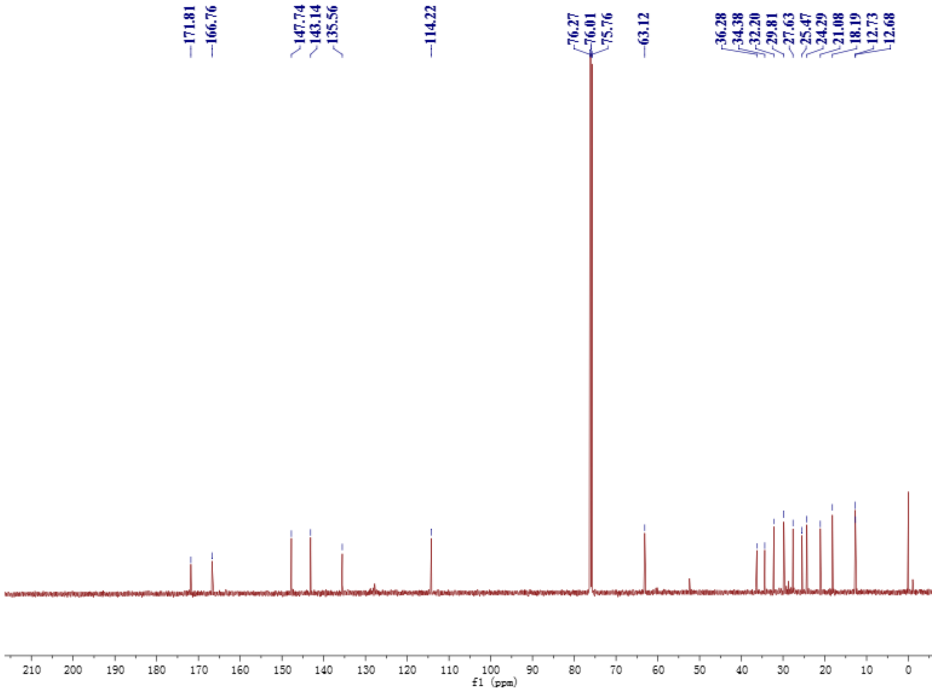


**Supplementary Figure 191.** $^{13}$C NMR spectrum for **7ta** in $CDCl_3$

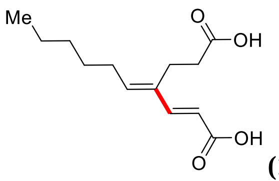


**(12)**

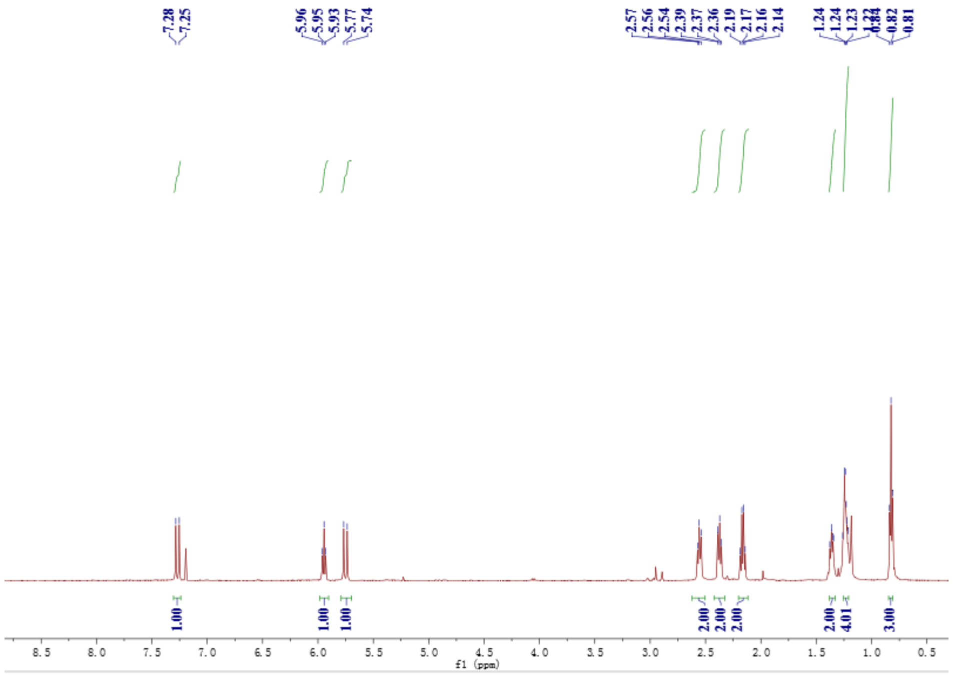


**Supplementary Figure 192.** $^{1}$H NMR spectrum for **12** in $CDCl_3$

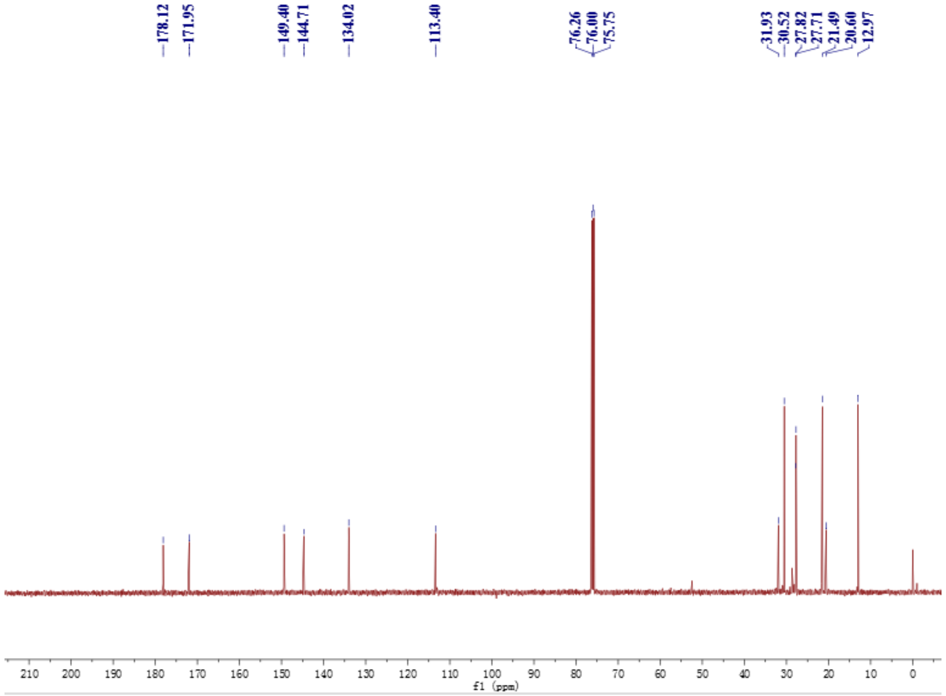


**Supplementary Figure 193.** $^{13}$C NMR spectrum for **12** in $CDCl_3$

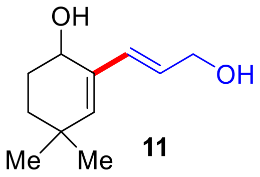


**11**

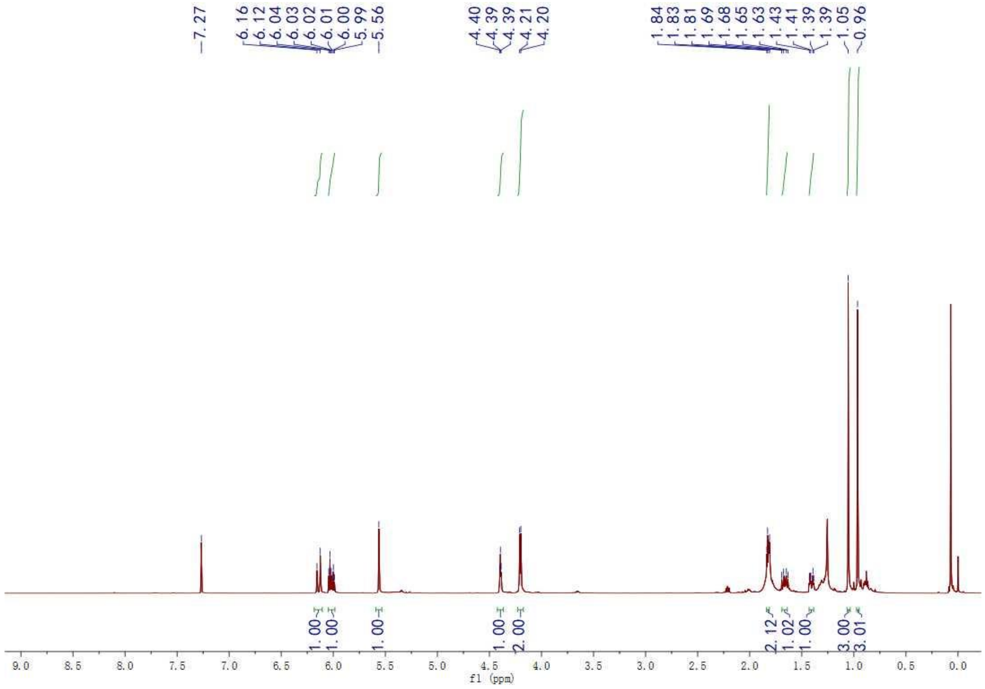


**Supplementary Figure 194.** $^{1}H$ NMR spectrum for **11** in $CDCl_3$

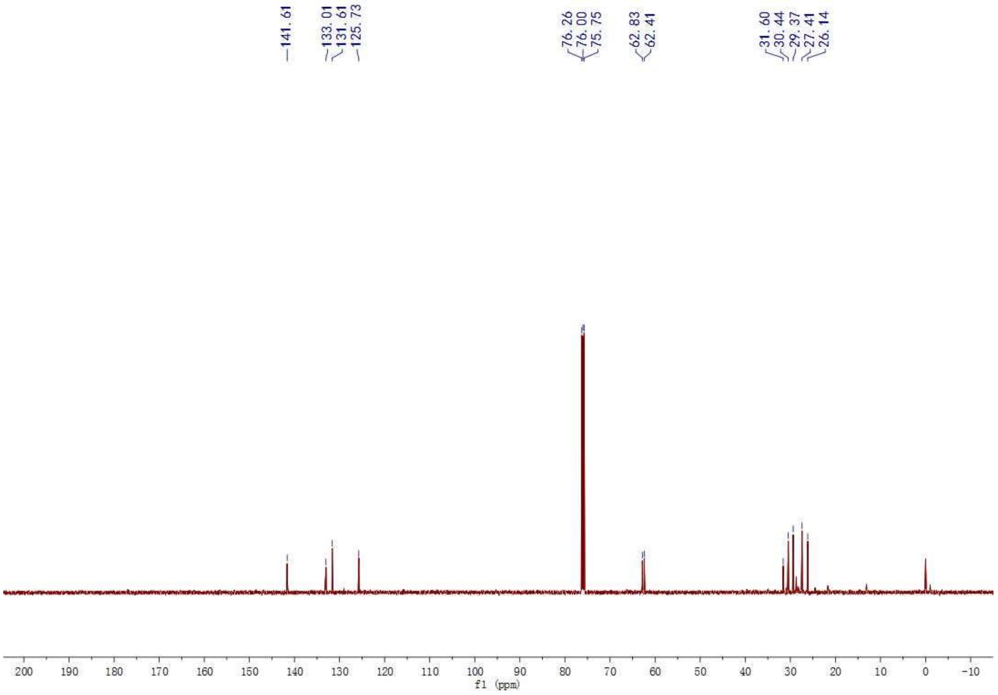


**Supplementary Figure 195.** $^{13}C$ NMR spectrum for **11** in $CDCl_3$

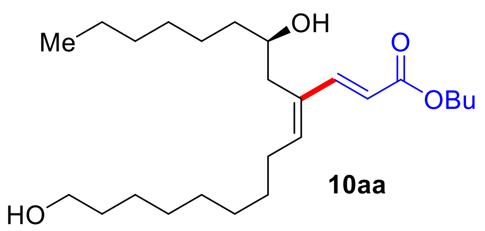


**10aa**

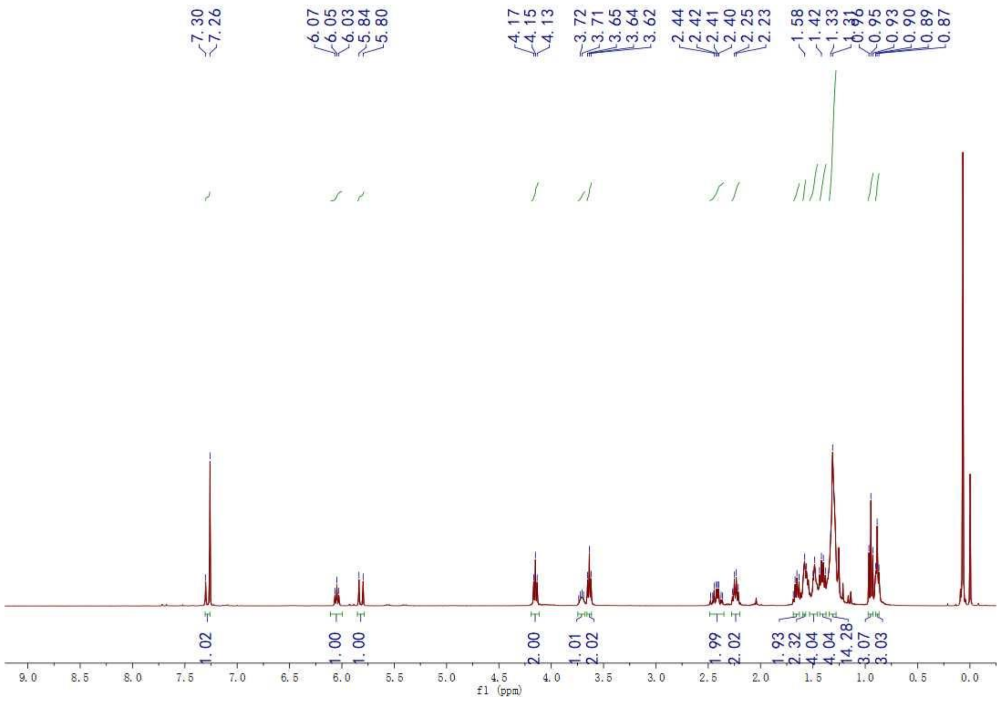


**Supplementary Figure 196.** $^{1}$H NMR spectrum for **10aa** in $CDCl_3$

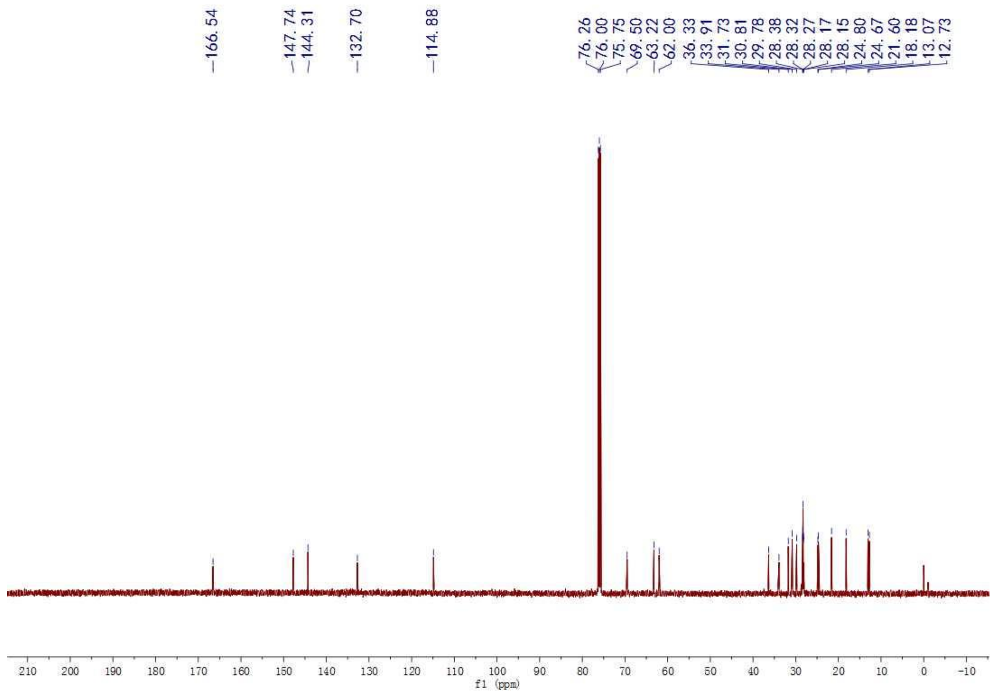


**Supplementary Figure 197.** $^{13}$C NMR spectrum for **10aa** in $CDCl_3$

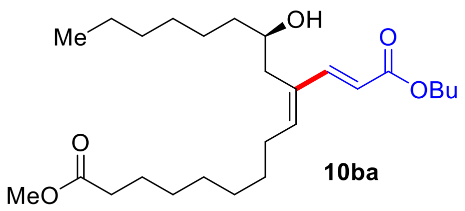


**10ba**

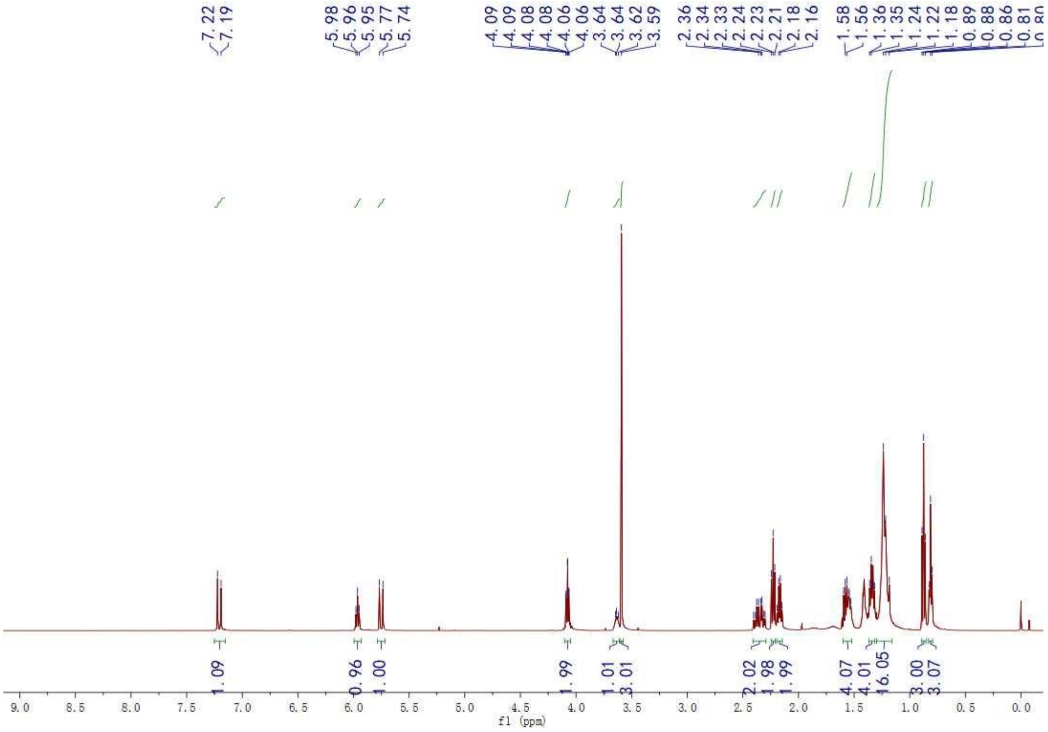


**Supplementary Figure 198.** $^{1}H$ NMR spectrum for **10ba** in $CDCl_3$

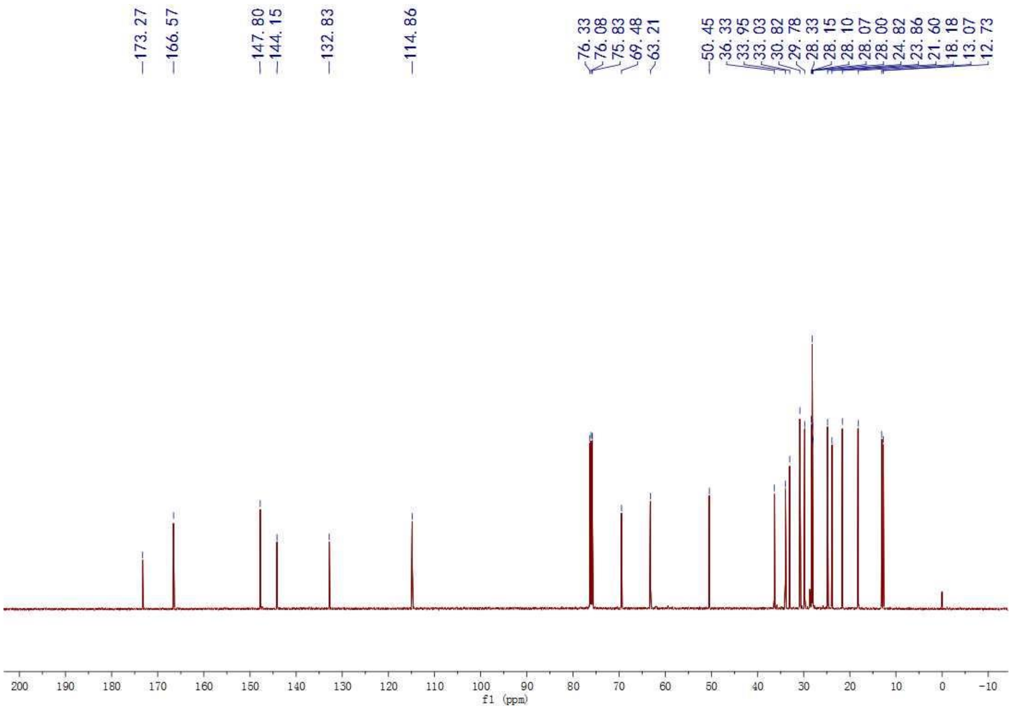


**Supplementary Figure 199.** $^{13}C$ NMR spectrum for **10ba** in $CDCl_3$

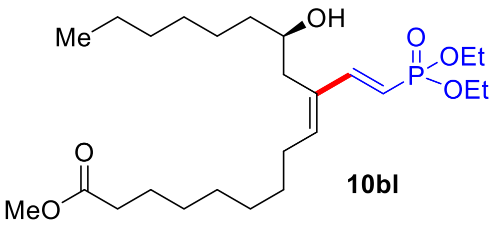


**10bl**

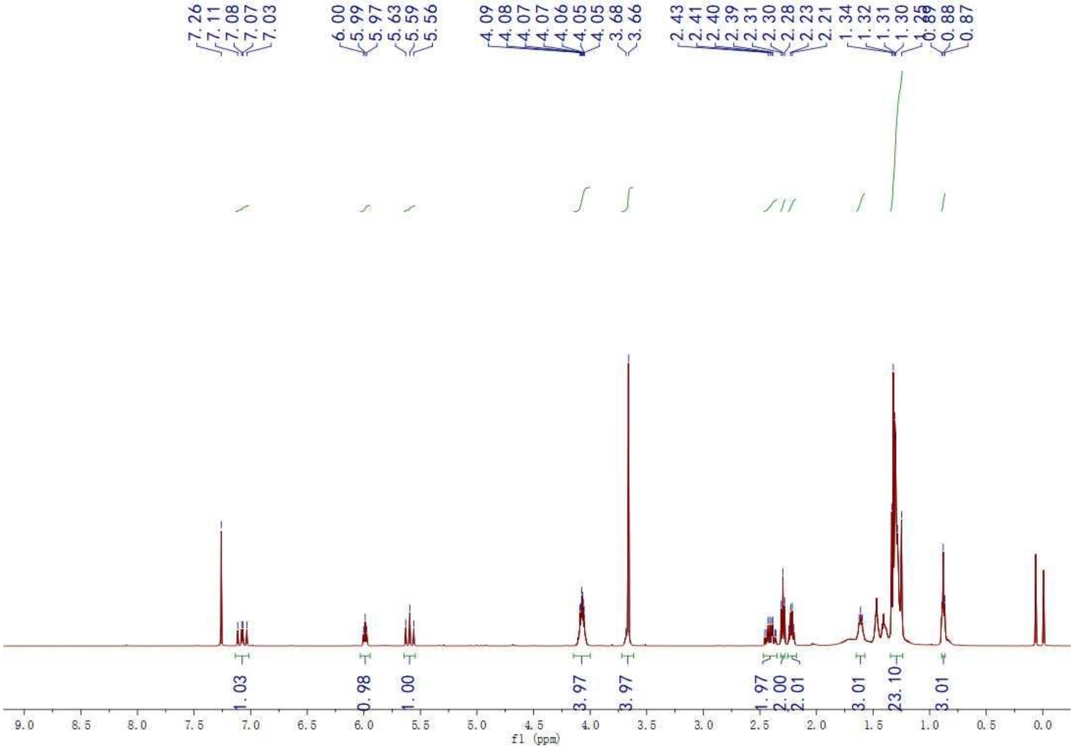

**Supplementary Figure 200.** $^{1}H$ NMR spectrum for **10bl** in $CDCl_3$

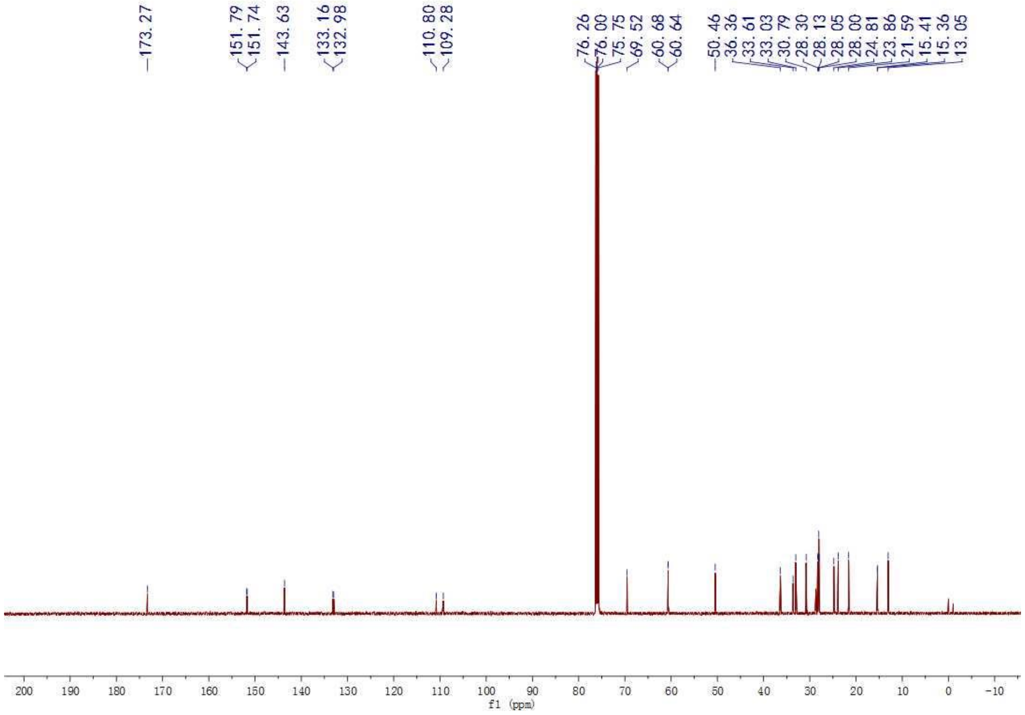

**Supplementary Figure 201.** $^{13}C$ NMR spectrum for **10bl** in $CDCl_3$

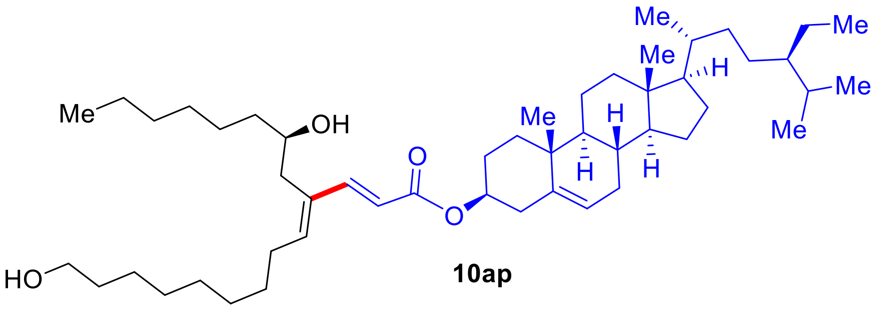

**10ap**

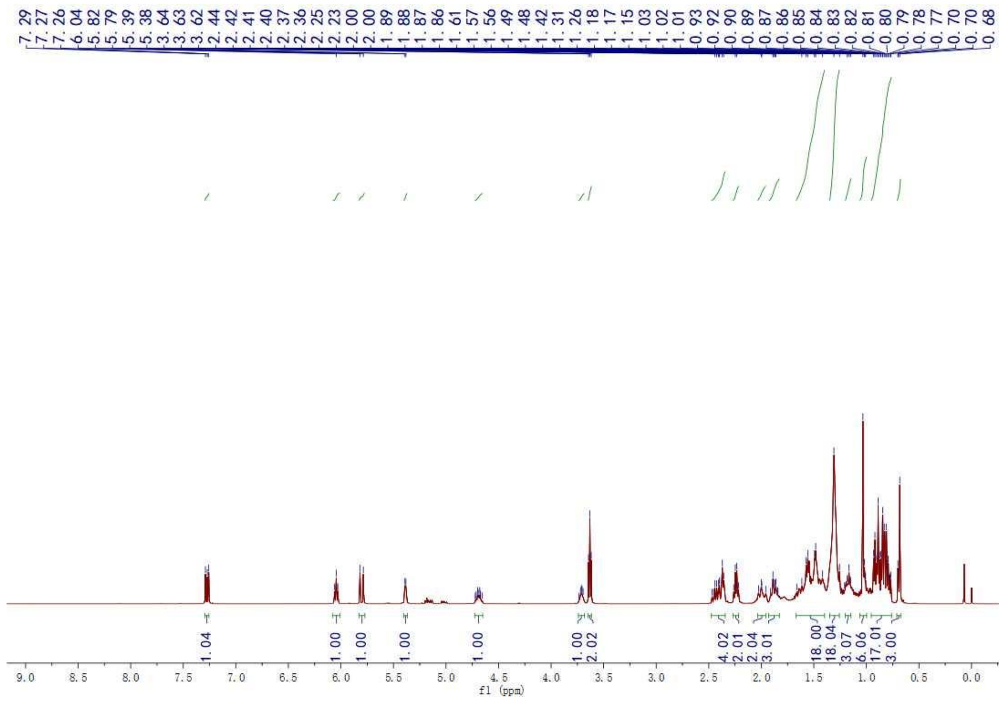

**Supplementary Figure 202.** $^{1}H$ NMR spectrum for **10ap** in $CDCl_3$

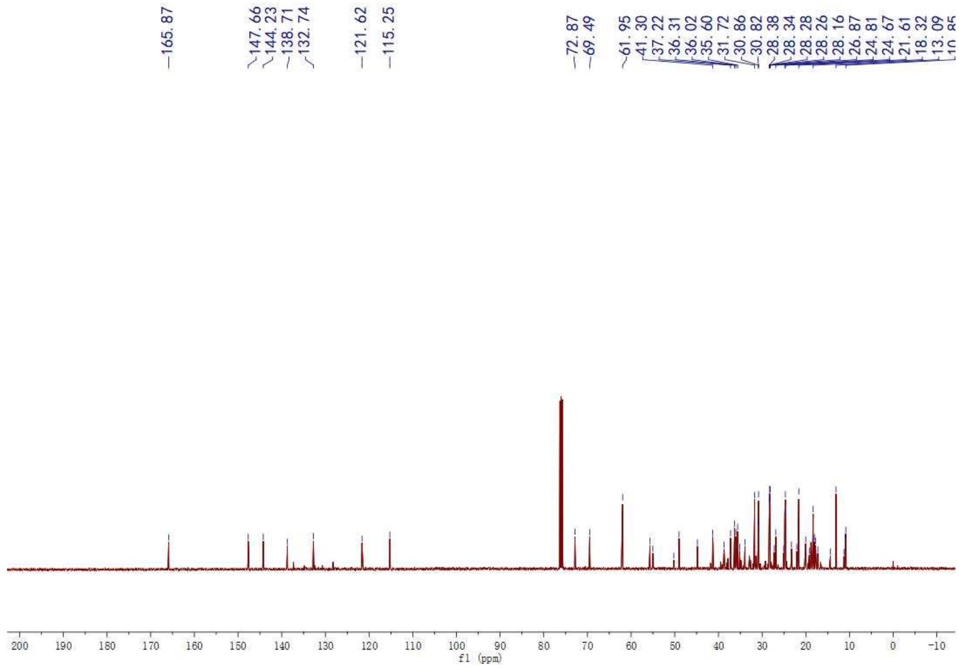

**Supplementary Figure 203.** $^{13}C$ NMR spectrum for **10ap** in $CDCl_3$

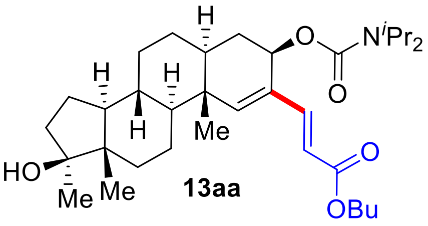


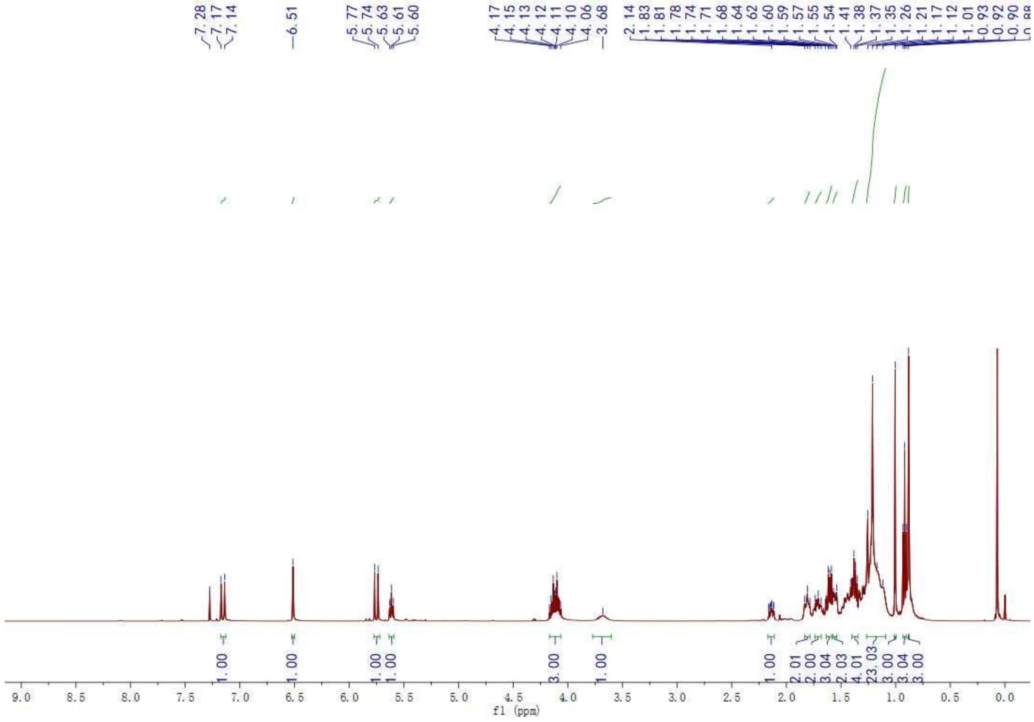


**Supplementary Figure 204.** $^{1}$H NMR spectrum for **13aa** in $CDCl_3$

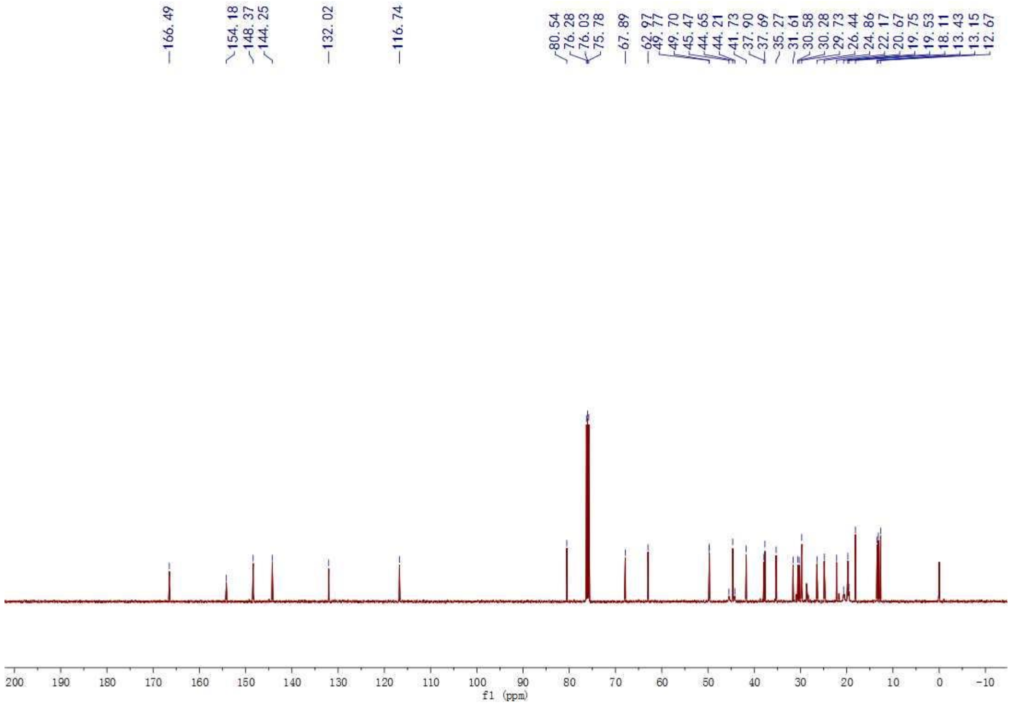


**Supplementary Figure 205.** $^{13}$C NMR spectrum for **13aa** in $CDCl_3$

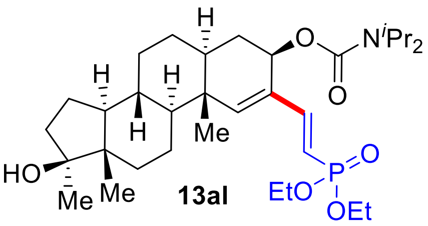


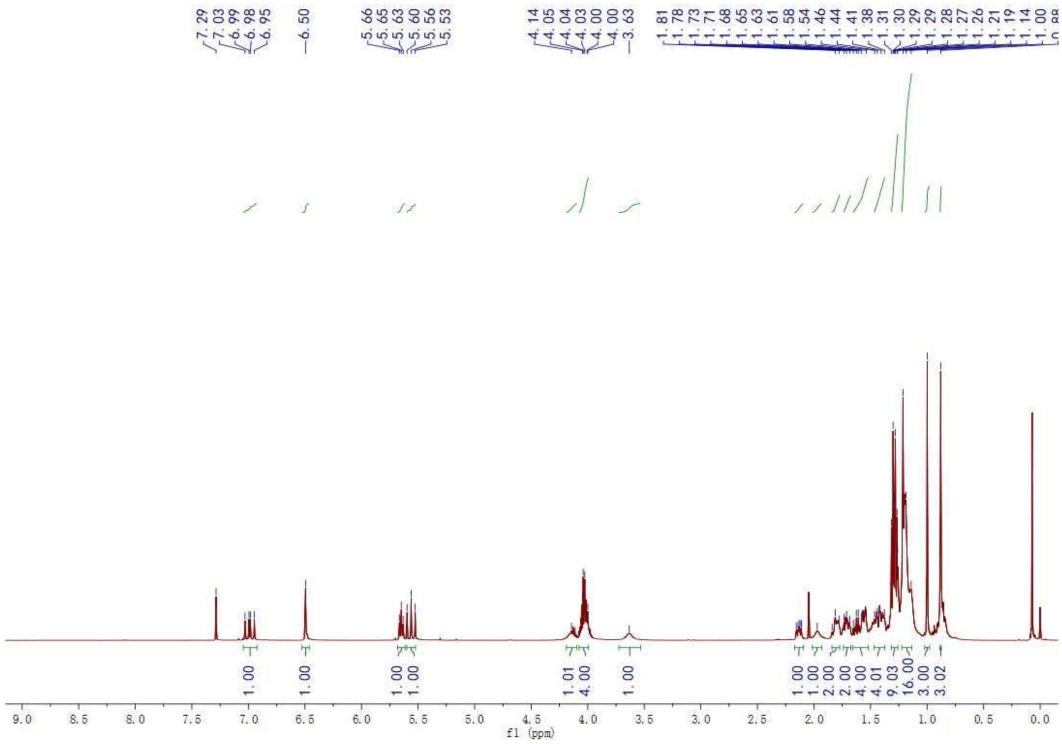


**Supplementary Figure 206.** $^1$H NMR spectrum for **13al** in $CDCl_3$

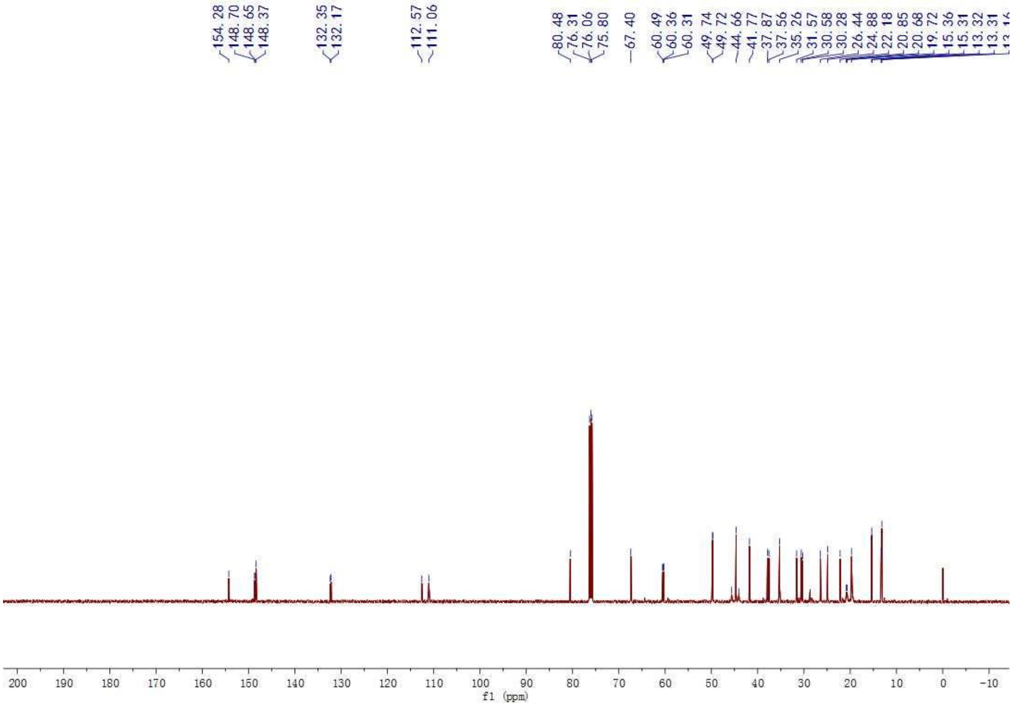


**Supplementary Figure 207.** $^{13}$C NMR spectrum for **13al** in $CDCl_3$

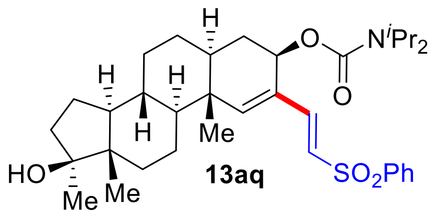


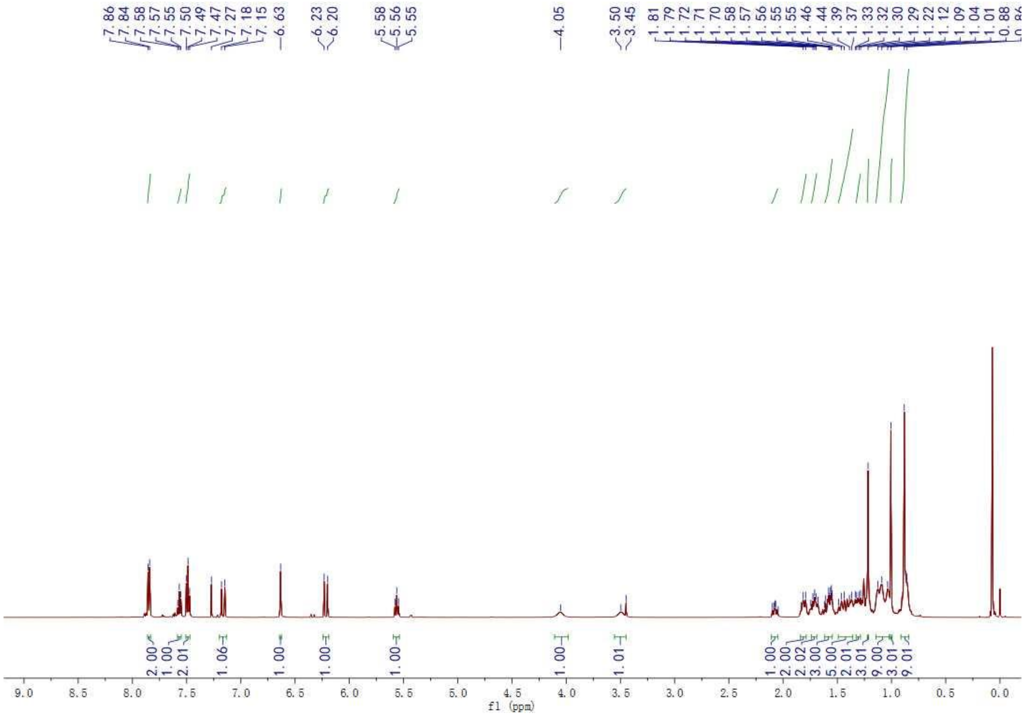

**Supplementary Figure 208.** $^{1}H$ NMR spectrum for **13aq** in $CDCl_3$

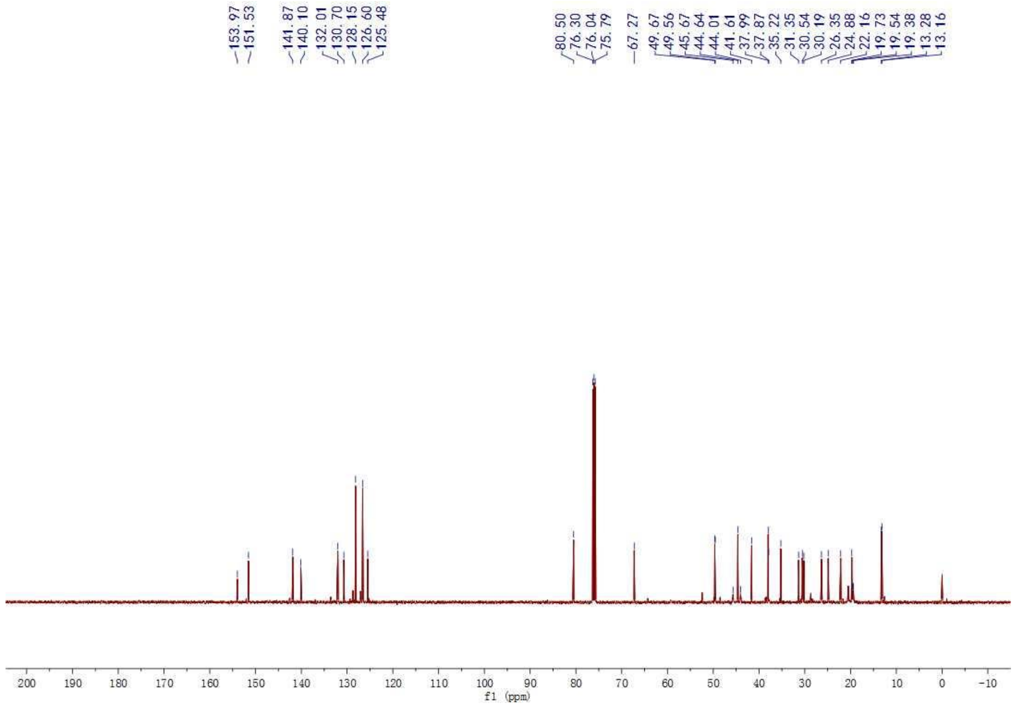

**Supplementary Figure 209.** $^{13}C$ NMR spectrum for **13aq** in $CDCl_3$

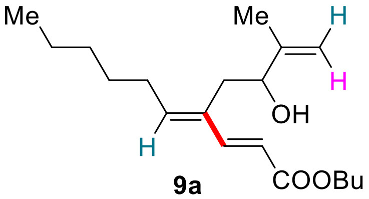


**9a**

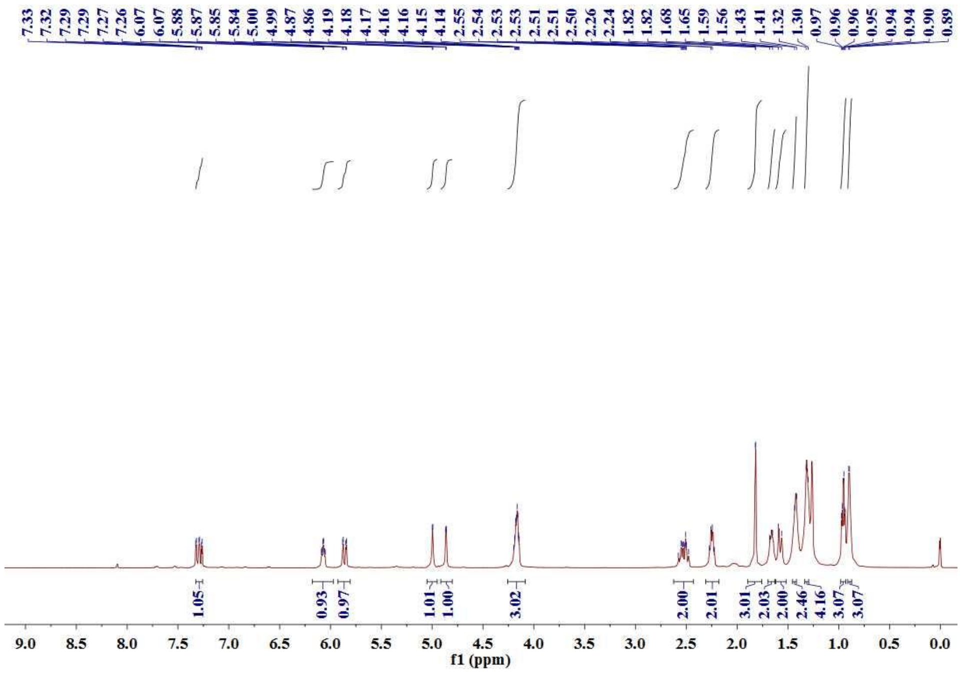


**Supplementary Figure 210.** $^{1}$H NMR spectrum for **9aa** in $CDCl_3$

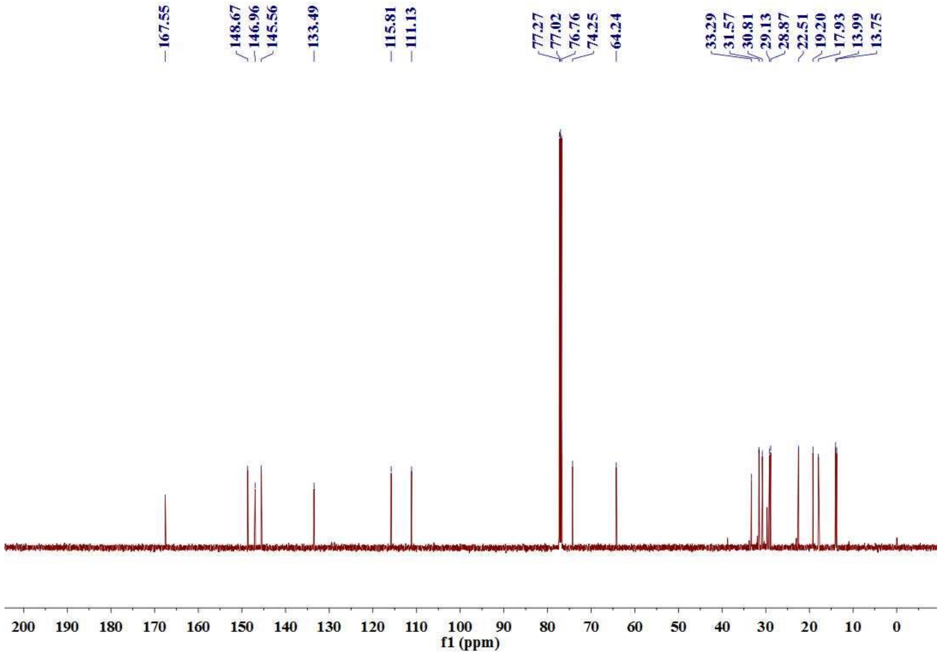


**Supplementary Figure 211.** $^{13}$C NMR spectrum for **9aa** in $CDCl_3$

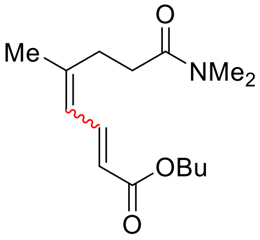


($Z$,$E$/$E$,$E$ = 65/35)

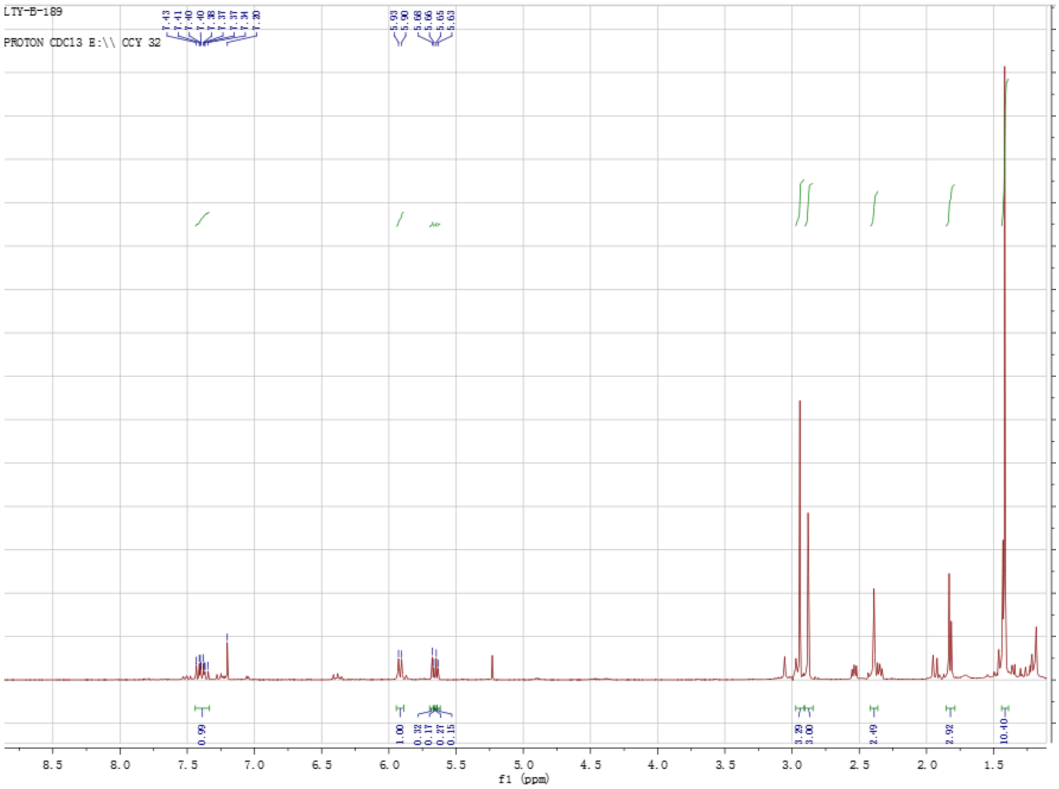


**Supplementary Figure 212.** $^1$H NMR spectrum for the product from 4-methyl-4-pentenamide

## Supplementary References


1. Hémery, T., Huenerbein, R., Fröhlich, R., Grimme, S. & Hoppe, D. Configurationally labile enantioenriched lithiated 3-arylprop-2-enyl carbamates: joint experimental and quantum chemical investigations on the equilibrium of epimers. *J. Org. Chem*. **75**, 5716-5720 (2010).
2. Kim, I. S., Dong, G. R. and Jung, Y. H. Palladium(II)-catalyzed isomerization of olefins with tributyltin hydride. *J. Org. Chem*. **72**, 5424-5426 (2007).
3. Lorber, K., Schieberle, P. & Buettner, A. Influence of the chemical structure on odor qualities and odor thresholds in homologous series of alka-1,5-dien-3-ones, alk-1-en-3-ones, alka-1,5-dien-3-ols, and alk-1-en-3-ols. *J. Agric. Food Chem*. **62**, 1025-1031 (2014).
4. Ohta, Y.; Yasuda, S.; Yokogawa, Y.; Kurokawa, K.; Mukai, C. Stereospecific and stereoselective rhodium(I)-catalyzed intramolecular [2+2+2] cycloaddition of allene-ene-ynes: construction of bicyclo[4.1.0]heptenes. *Angew. Chem., Int. Ed*. **54**, 1240-1244 (2015).
5. Walker, P. R.; Campbell, C. D.; Suleman, A.; Carr, G.; Anderson, E. A. Palladium- and ruthenium-catalyzed cycloisomerization of enynamides and enynhydrazides: a rapid approach to diverse azacyclic frameworks. *Angew. Chem., Int. Ed*. **52**, 9139-9143 (2013).
6. Zeng, X.; Miao, C.; Wang, S.; Xia, C.; Sun, W. Asymmetric 5-*endo* chloroetherification of homoallylic alcohols toward the synthesis of chiral $\beta$-chlorotetrahydrofurans. *Chem. Commun*. **49**, 2418-2420 (2013).
